# Sarcoma Meeting Stuttgart 2005

**DOI:** 10.1080/13577140500162409

**Published:** 2005


*Sarcoma,* March/June 2005; 9(1/2): 43–118

Taylor & Francis

Taylor & Francis Group

### Sarcoma Meeting Stuttgart 2005

### Key lectures

### KL01

**Adjuvant and neoadjuvant chemotherapy for osteosarcoma of the extremity: Experience at Istituti Ortopedici Rizzoli, in 1148 patients treated between 1972 and 1999, with a 5 to 27-year follow-up**

G. Bacci
*(Department of Musculoskeletal Oncology, Istituti Ortopedici Rizzoli, Bologna, Italy)*

1148 patients with non-metastatic osteosarcoma of the extremity were treated in a single institution between 1972 and 1999 with 4 protocols of adjuvant chemotherapy (248) and 5 protocol of neoadjuvant chemotherapy (900). The overall rate of limb-salvage was 71%, increasing from 20% of the first adjuvant studies to 96% obtained with the last neoadjuvant protocol.

5-year EFS and OS were 57% and 66% respectively, and they were significantly related to serum values of alkaline phosphatase, type of chemotherapy, and grade of histologic response to preoperative chemotherapy (in patients treated with neoadjuvant protocols).

The 61 local recurrences were significantly related to surgical margins. The 5-year post-relapse EFS was 17%, and it was significantly higher for patients relapsing with lung metastases than for those with local recurrence. It was also significantly related to the number of metastases and duration of the disease-free interval.

In our experience, the combination of aggressive chemotherapy and surgery can cure more than the 60% of patients with osteosarcoma of the extremity, preserving the limb in most patients.

### KL02

**Alveolar rhabdomyosarcoma: Molecular pathogenesis and therapeutic implications**

F.G. Barr, S.J. Xia
*(Department of Pathology and Laboratory Medicine, University of Pennsylvania, Philadelphia, USA)*

Alveolar rhabdomyosarcoma is an aggressive soft tissue cancer associated with the skeletal muscle lineage. These cancers typically have a characteristic 2;13 chromosomal translocation, which fuses the PAX3 and FKHR genes and results in a chimeric PAX3-FKHR protein. Our studies in NIH3T3 fibroblasts demonstrate that the fusion protein has transforming activity at low expression level, but suppresses growth at higher expression level. Using PAX3-FKHR mutants with inactivating mutations in the paired box or homeobox, we find that the homeobox is required for transformation whereas the paired box is required for growth suppression. Subsequently, we identified a vector that reduced PAX3-FKHR expression to a level that does not cause growth suppression. In this expression system, the paired box mutant demonstrates substantially enhanced transforming activity. This finding indicates that the paired box exerts an inhibitory effect on PAX3-FKHR oncogenic behavior, and thus we hypothesize that events that interfere with PAX3-FKHR paired box function, either by protein-protein interaction or direct mutation, will promote tumorigenesis by increasing oncogenic activity and decreasing growth suppressive activity. Therefore, another avenue for therapeutic strategies is to target these collaborating events that permit the cell to tolerate the growth suppressive activity of PAX3-FKHR or decrease its additional oncogenic potency.

### KL03

**Experimental systemic therapies in bone tumors**

M. Bernstein
*(Department of Pediatrics and Oncology, Hopital Ste. Justine, Montreal, Canada)*

The most common bone sarcomas are osteosarcoma and Ewing sarcoma, with 450 and 225 cases in North America each year in those younger than 20 years. Osteosarcoma occurs primarily in long bones, Ewing sarcoma also in the pelvis, spine or chest wall. Localized disease confers a 65% cure rate; initially metastatic disease, 25%. Management includes systemic chemotherapy and local control measures for primary and metastatic sites. Complete surgical excision is mandatory in osteosarcoma; in Ewing, radiation can also be used.

Chemotherapy for osteosarcoma is based on a combination of cisplatin, doxorubicin and high-dose methotrexate. Ifosfamide and etoposide, and alpha interferon, will be further examined in the Euramos (European and American Osteosarcoma) study. Ongoing investigations include the fas, fas ligand pathway and anti-folates.

Systemic therapy for Ewing includes vincristine, doxorubicin, cyclophosphamide, ifosfamide and etoposide. The current Children's Oncology Group (COG) protocol studies increased dose density. High-dose therapy with autologous stem cell reinfusion is being evaluated in patients with intermediate prognosis disease (Euro-EWING). The addition of the combination of vincristine, topotecan and cyclophosphamide to standard therapy will be assessed in the next COG study. Investigation of mechanisms encouraging tumor development or conferring resistance will lead to therapeutic innovation.

### KL04

**Radiotherapy and targeted radionuclide therapy in osteosarcoma**

Ø.S. Bruland
*(Department of Oncology, The Norwegian Radium Hospital, Oslo, Norway)*

The lecture will focus on some tumour- and radiobiological aspects of osteosarcoma (OS). It will present our clinical experiences employing external beam radiotherapy in primary inoperable or relapsing OS patients, and aspects on the physical and biological optimization will be discussed with inputs from members of the radiotherapy task group within EUROBOSS. One goal is find options to avoid conventional radiotherapy being used as an ultimate

ISSN 1357–714X print/ISSN 1369–1643 online © 2005 Taylor & Francis Group Ltd
DOI: 10.1080/13577140500162409

refugium, and rather define its indications and integration into the primary multimodal treatment. Cis-Pt based chemo-irradiation might be one such option. Experiences from a Scandinavian Sarcoma Group protocol (SSG XIII) where high-risk soft tissue sarcoma patients were treated post-operatively with a combination of doxorubicine and ifosfamide-based chemotherapy and risk-adapted accelerated/hyper-fractionated radiotherapy will be put forward for discussion. Lastly, results from a dose-escalating phase-I/II study using Samarium 153-EDTMP with stem cell support in 15 OS patients will be presented. The treatment was well tolerated and toxicity was mild. At injected activity levels below 3.0 mCi/kg, repeated treatments were feasible without the need for PBCS. The radiotherapy-boost from such bone-seeking targeted radionuclide therapy resulted in long-tern local control when given in combination with external beam irradiation. However, it was seemingly not efficient to prevent growth of chemo resistant micro metastases in the lungs.

### KL05

### Follow up

Sir A. Craft
*(Department of Child Health, Sir James Spence Institute, Royal Victoria Infirmary, Newcastle Upon Tyne, UK)*

Follow up is essential following treatment for sarcoma. There are several reasons for this. The most important is the early detection of recurrence so that further potentially curative treatment can be instituted. The other major reason is for the detection of late effects of treatment and if necessary institution of remedial treatment.

*Detection of recurrence:* Sarcomas have a particular pattern of recurrence and this may be local, at the site of original disease, or distant metastatic – usually to the lung. Detection of local recurrence depends on the initial local treatment. If this has involved amputation then it is much less likely to happen although stump metastases do occur. Regular clinical examination and imaging depending on the site will be important. U/S, CT and MRI can all have a part to play.

Metastatic disease will be detected by regular chest imaging either with plain chest xray or with CT.

Further treatment may need any of the modalities of surgery, radiotherapy or chemotherapy. The chances of long term survival/cure depend on number of metastases, surgical resectibility and the time of occurrence after the end of treatment.

*Detection of late effects:* Chemotherapy as well as radiotherapy and surgery can produce late effects and the patient should be given a detailed follow up plan depending on their particular risk of late effects. Many centres now have late effects clinics often led by nurses.

### KL06

### Pediatric soft tissue sarcoma – dose, volume, and timing of radiotherapy for local tumor control

S.S. Donaldson
*(Department of Radiation Oncology, Stanford University, Stanford, USA)*

*Purpose:* To address issues of local tumor control in rhabdomyosarcoma and non-rhabdomyosarcoma (NRSTS) group of tumors.

*Methods:* A review.

*Results/discussion:* For NRSTS, histologic grade (tumor biology) predicts outcome and patterns of failure. Surgical excision is important for local management. Indications for radiotherapy are a function of tumor grade, size, and adequacy of surgery. Radiotherapy following marginal excision for high-grade tumors improves local control. Dose, volume, timing and type of radiation must be individualized depending on clinical parameters, but usually involves high radiation doses administered by conformal techniques.

Radiotherapy is central to the multidisciplinary management of most patients with rhabdomyosarcoma, a high-grade tumor. Required doses and volumes vary with histologic subtype, group/stage, age and primary site. The estimated 5-year local control for IRSG IV patients with gross disease at study entry (Group III) is 86%. Features predicting local failure include: alveolar/undifferentiated histology; truncal, extremity, and parameningeal sites; T2, >5 cm, and N1 status. Systematic chemo-radiotherapy for patients with non-resected disease, as used in IRS IV, appears to provide improved event free and overall survival rates when compared with studies emphasizing chemotherapy but reducing local radiation therapy.

The goal in pediatric sarcomas is high cure rate, preserving form and function.

### KL07

### Sarcoma PET imaging

J.F. Eary
*(University of Washington, Seattle, Washington, USA)*

Sarcoma PET imaging has been explored by our group to understand the contribution this imaging technique can make to the diagnosis, treatment planning and follow-up of sarcoma patients. We have used the radiotracer [F-18]FDG to quantitate tumor metabolism in over 500 sarcoma patients. This imaging method, now used clinically throughout much of the world, can be used to determine sarcoma tumor grade. In a patient who is receiving neoadjuvant treatment, the FDG PET derived tumor metabolism data is a very sensitive means of determining treatment response. This information also can be used to predict patient outcome before, after, and during therapy. Newer radiotracers will likely make an even more significant contribution to sarcoma patient care by providing a means of quantitating tumor cell proliferation, cell population death, levels of regional tumor hypoxia, and activity of drug resistance mechanisms to chemotherapy agents. Many of these are being studied currently in sarcoma patient imaging protocols. In this symposium lecture, an overview with discussion of significant findings from PET imaging in sarcoma studies will be presented. Current clinically relevant findings as well as future research directions will be presented to provide background for sarcoma issues to be discussed at this symposium.

### KL08

### TNF-based isolated limb perfusion for locally advanced extremity STS

A.M.M. Eggermont
*(Department of Surgical Oncology, Erasmus University Medical Center, Daniel Den Hoed Cancer Center, Rotterdam, The Netherlands)*

Isolated Limb Perfusion (ILP) with melphalan has a successful track record as the treatment of choice of multiple (small) melanoma in-transit metastases. The application of Tumor Necrosis Factor-alpha (TNF) in ILP has made it a successful modality to treat locally advanced extremity soft tissue sarcomas and other large tumors, and achieve limb salvage. TNF was approved in

Europe based on the outcome of a multicenter trial European trial that showed that ILP with TNF + melphalan resulted in a 76% response rate and in limb salvage in 71% in patients with locally advanced soft tissue sarcomas, deemed irresectable by an independent review committee. Moreover efficacy of TNF + melphalan ILP was shown against various other limb threatening tumors such as skin cancers and drug resistant bone-sarcomas.

ILP models in the laboratory have provided a model system to elucidate our insights in mechanisms of action and develop new treatment modalities. These models have identified TNF-mediated vasculotoxic effects on the tumor vasculature and have shown that addition of TNF to the perfusate results in a 3–6 fold increase in melphalan or doxorubicin uptake in tumors. New (vaso-active) drugs and new mechanisms of action are being discovered.

### KL09

**EUROpean bone over 40 sarcoma study (EURO-B.O.S.S) A European treatment protocol for bone-sarcoma in patients older than 40 years**

S. Ferrari[1], S. Smeland[2], S. Bielack[3]
*([1]Italian Sarcoma Group, [2]Scandinavian Sarcoma Group, [3]Cooperative Osteosarcoma Study Group)*

The trial EUROBOSS is a multicentre prospective study for patients older than 40 years with highly malignant sarcoma of bone: Osteosarcoma, Fibrosarcoma, Malignant Fibrous Histiocytoma (MFH), Leiomyosarcoma, Dedifferentiated Chondrosarcoma.

The present EUROBOSS protocol is based on the experience of the participating intergroups in the treatment of spindle cell bone sarcomas and their past and present osteosarcoma protocols.

The study is a first step of a process to establish the standard chemotherapy treatment with the aim to improve outcome for patients with these rare tumours.

In this regard, the study aims to determine the feasibility of intensive chemotherapy in this age group, and/or separate efficacy analyses according to the different histologic categories and whether the number of patients recruited by the co-operating groups permits future randomised studies.

Primary aim is to evaluate clinical outcome and chemotherapy-related toxicity in patients 41–65 years old with high-grade bone sarcoma treated with a three drug chemotherapy regimen containing adriamycin (ADM), cisplatin (CDP) and ifosfamide (IFO), and the addition of methotrexate (MTX) to poor histologic responders

The study is open to collaboration with other Groups or Institutions, after agreement of all participating groups.

### KL10

**Treatment of adults according to pediatric protocols – The Milan experience**

A. Ferrari, M. Casanova, P. Collini, L. Gandola, C. Meazza, A. Gronchi, P.G. Casali, F.F. Fossati Bellani
*(Istituto Nazionale Tumori, Milan, Italy)*

*Purpose:* Published series on adult rhabdomyosarcoma (RMS) reported definitely worse results than in children, casting doubts as to whether RMS is the same disease in adults as in childhood patients. 171 RMS patients >18 years treated over a 25-year span were retrospectively analyzed. Patients were stratified according to how much they had been treated appropriately, in the light of current treatment criteria for childhood RMS.

*Results:* In the whole series, 5yr-EFS and OS were 28% and 40%. Considering only patients with embryonal, alveolar, or not-otherwise-specified RMS (n.110), 5yr-OS was 46%, but was 61% in those cases (39% of the total) who scored high for appropriate treatment.

*Conclusions:* Our series confirms a relatively poor outcome for RMS in adults. However, outcome was similar to childhood series for those patients whose treatment could actually reflect the criteria currently employed in children. In addition, the chemotherapy response-rate (85%) was super-imposable to that usually observed in children. These findings would suggest to treat adults with RMS with intense approach mutated from pediatric programs, but tailored for adults considering that these might tolerate to a lesser degree treatments which have been designated for children.

### KL11

**Tumor biology of bone sarcoma: Molecular pathogenesis and therapeutic implications**

R. Gorlick
*(The Children's Hospital at Montefiore and the Albert Einstein College of Medicine. Bronx, NY 10467, USA)*

All sarcomas can be characterized as those with recurrent consistent chromosomal translocations and relatively simple karyotypes and those with multiple tumor suppressor gene abnormalities with complex karyotypes. Osteosarcoma is an example of the latter category. Multiple germ line alterations result in predispositions to osteosarcoma including alterations in p53 in patients with Li-Fraumeni syndrome, Rb in patients with hereditary retinoblastoma, WRN in patients with Werner syndrome and RecQL4 in patients with Rothmund-Thomson syndrome. In addition, environmental exposures to ionizing radiation or increased bone turnover as observed in growing patients or with Paget's disease, predisposes to osteosarcoma. This may suggest osteosarcoma is the common endpoint of multiple genetic events but this is refuted by the rarity of the disease. The genetic complexity observed in tumors obtained from patients with sporadic osteosarcoma makes it unclear which features are centrally linked to tumor initiation and progression. Factors central in the molecular pathogenesis are likely to be prognostic and therapeutic targets. The identification of these events is of critical clinical importance and likely will need to include assessments of pathways and not just individual genes. Our laboratories and the Children's Oncology Groups efforts to define prognostic factors and therapeutic targets for osteosarcoma will be presented.

### KL12

**Local control in bone sarcomas**

R.J. Grimer
*(Royal Orthopaedic Hospital, Birmingham, UK)*

Limb salvage surgery is now the standard treatment for bone sarcomas. Amputation is still required for large tumours which involve the neurovascular bundle or at locations where complete resection of the tumour will lead to a worse result than limb salvage. Limb salvage is however associated with an increased risk of local recurrence and the factors associated with this and the significance of local recurrence are still not clear.

Margins of excision play a key role in determining the risk of local recurrence, but the effectiveness of neoadjuvant treatment is also highly relevant.

| | Total | Intraleioal | Marginal | Wide |
|---|---|---|---|---|
| Osteosarcoma | 16% | 38% | 26% | 8% |
| OS >90% necrosis | 4% | – | 18% | 2% |
| OS $\leq$90% necrosis | 19% | 51% | 24% | 13% |
| Ewing's >90% necrosis | 1% | 0 | 0 | 2% |
| Ewing's <90% necrosis | 17% | 16% | 28% | 12% |
| Chondrosarcoma | 24% | 35% | 26% | 17% |

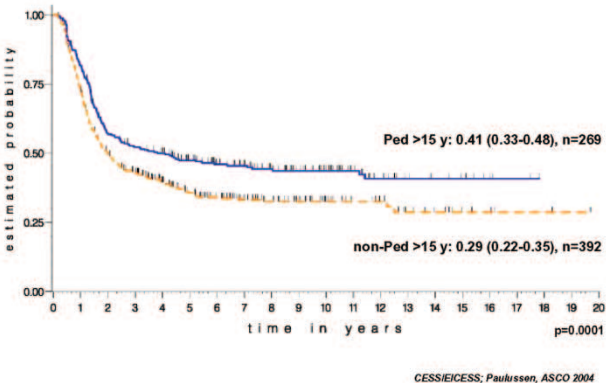

Figure. Survival after local recurrence – all bone tumours.

This table shows that even with wide margins but ineffective neoadjuvant the risk of LR is >10%. The definition of what is a wide margin varies greatly between institutions and attempts to clarify this are being made.

Once a patient has developed local recurrence, salvage treatment is related to the site of the LR and the presence or absence of disease elsewhere. In patients with metastases as well as LR the prognosis is appalling but in patients without metastases at the time of LR there is a 37% chance of surviving 5 years and a 26% chance of surviving 10 years.

Whether local recurrence poses an increased risk to overall survival is a difficult question to answer as the same factors which lead to LR – poor response to neoadjuvant – also lead to poor survival. A shared risk analysis will be presented to estimate the significance of LR on survival in bone sarcomas. Local recurrence is a devastating complication. Whether it is possible to predict patients at risk of LR who may have a better prognosis if they undergo primary amputation remains a subject of controversy!

### KL13

**Radiotherapy in the local control of soft tissue sarcomas**

J.-L. Habrand, O. Oberlin, C. Lepechoux
*(Institut Gustave Roussy, Villejuif, France)*

Soft tissue sarcomas are rare conditions both in adults and in children that represent approximately 1 and 5% respectively of malignancies. They also comprise a wide variety of pathological subgroups that are clearly different in both age groups, along with different clinical behavior, radio/chemosensitivity, and prognosis. On an other hand, tolerance of normal tissues appears quite different in both groups, with an exquisite sensitivity to radiations of immature soft and bone tissues. All these factors explain different treatment strategies, including divergences in the management of similar tumors by pediatric and adult oncological groups. Despite such a rarity and disparity, considerable efforts have been made by large institutions, as well as national and international groups in order to clarify their management. As far as radiotherapy, its crucial role has been evidenced relatively recently, mainly due to the false reputation of radioresistance of the adult subtypes. Nowadays, combined radio-surgical approaches have become standardized in adults and chemo-radiations in childhood. Evidence-based decisions become easier with the accumulation of large clinical series, including phases III. We will update projects that have evaluated the role of local treatment with emphasis on radiotherapy.

### KL14

**mTOR – A major determinant of biologic behavior in pediatric sarcomas**

X. Wan, C. Yeung, B. Midura, C. Khanna, L. Helman
*(Pediatric Oncology Branch, Center for Cancer Research, National Cancer Institute, Bethesda, Maryland, USA)*

We recently identified ezrin as a determinant of metastatic behavior in two independent mouse models of pediatric sarcomas. Subsequently, we found an association of ezrin with activation of mTOR signaling. Since mTOR is targeted by rapamycin and analogs that are currently undergoing clinical testing in patients with a variety of tumors, we have continued to explore the possible role of mTOR signaling in the biology of pediatric sarcomas. We have demonstrated that inhibition of mTOR signaling using rapamycin inhibits motility and invasion in vitro and decreases experimental metastases in vivo. This appears to be at least partially linked to inhibition of HIF1 and VEGF expression in tumor cells. We also have used reverse phase proteomic analysis of rhabdomyosarcoma tumors treated on Children's Oncology Group studies to analyze mTOR activation prior to therapy. We found a strong correlation between activation of mTOR pathways and poor outcome in both embryonal and alveolar Stage III RMS patients. We are currently attempting to validate these findings in an independent group of Stage III RMS tumors. These data strongly suggest that mTOR signaling is a significant determinant of aggressive biological behavior and that targeting this pathway may have significant therapeutic benefit in pediatric sarcomas.

### KL15

**Tumor biology of bone sarcoma: Pathology and genetic aspects**

Pancras C.W. Hogendoorn
*(Department of Pathology, Leiden University Medical Center, Leiden, The Netherlands)*

It has now been a well-recognised fact that a substantial number of sarcomas harbour a tumour specific translocation. This notion has been initially forwarded by the recognition of t(11;22) and its variants but now an array of tumours such as myxoid liposarcoma, infantile fibrosarcoma, aneurysmal bone cyst or desmoplastic small round cell tumour can be characterised diagnostically at the tumour genetic level. This conceptional recognition – histomorphic and genetic tumour specificity – has lead to the launching of the 2002 WHO classification of soft tissue and bone tumours. While the majority of patients develop their sarcoma de novo, a number clearly are part of a congenital and inherited syndrome such as Familial adenomatous polyposis, Beckwith-Wiedemann syndrome, McCune-Albright syndrome, Multiple osteochondromas, Li-Fraumeni syndrome, Retinoblastoma syndrome, Rothmund Thomson syndrome, or Werner syndrome to name a few. The recognition of these syndrome related sarcoma patient helps understanding their disease but most importantly alerts to the necessity to carefully examine family members and opens screenings and prevention discussion. Whether syndrome patients are having a different prognosis per se and the tumour phenotype is completely identical to their sporadic counterparts is matter of intense research.

### KL16

**Rational diagnostics: Pathology and molecular biology**

I. Leuschner[1], C. Poremba[2]
*(Institutes of Pathology, [1]Christian-Albrechts-University Kiel, [2]Heinrich-Heine-University Düsseldorf, Germany)*

Soft tissue sarcomas of childhood and adolescence include a wide spectrum of entities. The so-called small round blue cell tumors and spindle cell tumors are especially difficult to classify based solely on conventional histology. To identify different subtypes of tumors special histochemical (e.g. PAS, Gomori, etc.) and immunohistochemical techniques are necessary. Analysis of protein expression by immunohistochemistry provides a helpful tool to investigate the histogenesis of tumors. A basic spectrum of antibodies should be included to study these tumors: Desmin and myogenin (or MyoD1) for skeletal differentiation; S-100, NSE, CD56, and synaptophysin for neural/neuroendocrine differentiation; CD3, CD20, and CD 79 alpha for malignant lymphomas; CD 34, sm-actin, and $\beta$-catenin for spindle cell tumors; additional antigens, e.g. Ki-67 and p53, for estimation of proliferation and tumor suppressor gene malfunctions. Nevertheless, the molecular analysis of tumors is necessary for demonstration of specific translocations/gene defects to specify and prove a diagnosis. For this purpose, RT-PCR for RNA expression analysis of gene fusion transcripts and multi-color FISH for analysis of chromosomal rearrangements are used. Further investigations, using CGH or DNA microrrays may help to sub classify such tumors, with respect to prognosis or prediction of therapeutic response.

### KL17

**Systemic tumor control**

W.H. Meyer
*(Section of Hematology/Oncology, Department of Pediatrics, OUHSC, Oklahoma City, OK, USA)*

With modern treatment over 70% of children and adolescents with rhabdomyosarcoma are cured. Multidisciplinary therapy is necessary to maximize cure rates. Chemotherapy is required for all patients. Local control relies on complete surgical excision when possible; those whose tumors are not completely excised and those with alveolar histology tumors receive local irradiation in COG studies. In North America vincristine, actinomycin and cyclophosphamide are the standard chemotherapy agents. In 2004 and 2005, COG initiates new studies for all newly diagnosed risk groups. The low-risk patient subsets receive four VAC cycles, followed by VA therapy. The intermediate risk trial plans to test VAC vs. VAC + irinotecan/vincristine, based on results from the D9802 window trial that demonstrates activity for this combination. The new high-risk study will use irinotecan/vincristine followed by dose compression therapy with alternating cycles of vincristine/cyclophosphamide/doxorubicin and ifosfamide/etoposide. Both the intermediate and high studies will evaluate irinotecan radiosensitization. New advances in molecular characterization of tumors may identify new therapeutic targets that can be exploited by expanded preclinical drug discovery efforts, and hold the promise to revolutionize risk based therapies.

### KL18

**Improved outcome in soft tissue sarcoma monitored by the Scandinavian Sarcoma Group (SSG) register**

S. Smeland[1], T.A. Alvegård[2], H.C.F. Bauer[3]
*([1]Department of Medical Oncology, Norwegian Radium Hospital, Oslo, Norway, [2]Southern Sweden Tumour Registry, University Hospital, Lund, Sweden, [3]Department of Orthopedics, Karolinska Hospital, Stockholm, Sweden)*

*Purpose:* Quality assessment of soft tissue sarcoma (STS) treatment by the SSG registry
*Patients:* The SSG register contains data on 3634 patients with STS diagnosed from 1986 to 2003.
*Results:* During this period the rate of primary referral before surgery for deep lesions has improved from 73% to 93% and the use of adjuvant radiotherapy for deep-seated STS from 24% to 44%. In the same period the 3-year local control rate has increased from 73% to 88% whilst the percentage of adequate margins did not change and the amputation rate fell from 13% to 5%.

The preliminary survival rate for the SSG XIII study for high-risk soft tissue sarcoma shows a projected 5-year metastases-free survival of 57% (CI ±15%) as compared to 38% in a previous population based study from Southern Sweden. When including patients reported to the SSG registry not recruited to the study the 5-year projected metastases-free survival rate drops to 48% (CI ±12%).
*Discussion:* The changes in referral patterns and increase in adjuvant radiotherapy seem to contribute to the improvement in local control. Population based materials is important to assess the utilisation of the protocol and improvement in outcome for the patients at risk.

### KL19

**Systemic treatment of soft tissue sarcoma with an emphasis on local control**

M.C.G. Stevens
*(University of Bristol, Department of Oncology, Royal Hospital for Children, Bristol, UK)*

Multimodality therapy involving surgery, chemotherapy and radiotherapy is important for the treatment of rhabdomyosarcoma (RMS). Experience with this tumour type is predominant in childhood soft tissue sarcoma (STS) and whilst this may not always be appropriate for treatment of non RMS STS, experience derived from different approaches taken to the treatment of RMS provides some insight into the role of systemic therapy in local tumour control. There is a debate about the necessity for radiotherapy (RT) in patients who achieve complete remission with chemotherapy, with or without surgical resection. The data show that when local therapy is systematically delivered as part of primary treatment, local control is generally more secure and the risk of relapse less. However, the use of RT, particularly in very young children, raises concern about its long term consequences and there is experience to suggest that it may be possible to avoid or reduce the intensity of local therapy depending on response to surgery and primary chemotherapy. Unfortunately, it is not yet possible to reliably identify those patients who are most likely to be cured without RT after achieving CR with chemotherapy and conservative surgery but a synopsis of the current data will be reviewed.

**KL20**

**Tumor biology of soft tissue sarcoma: Pathology and genetic aspects**
T.J. Triche
*(USC Keck School of Medicine and Chair of Pathology, Childrens Hospital Los Angeles, USA)*

Diagnostic classification of soft tissue sarcomas in the young readily divides into two main groups- rhabdomyosarcoma and a diverse group of non-rhabdomyosarcomas, including synoviosarcoma, extraosseous Ewing's, malignant peripheral nerve sheath tumor, fibrosarcoma, and unclassified sarcomas. For therapeutic purposes, current COG protocols largely follow this dichotomous classification. We have been studying global gene expression patterns in IRS-IV sarcomas for the past five years and find that these tumors readily divide into these two main groups as well. However, the classical subdivision of rhabdomyosarcoma into embryonal and alveolar forms is not substantiated. Instead, we find that only PAX-FKHR translocated alveolar forms cluster together; histologically alveolar un-translocated tumors are indistinguishable from embryonal forms. Further, several cases of embryonal rhabdomyosarcoma are non-myogenic and indistinguishable from non-rhabdomyosarcoma sarcomas, while other non-rhabdomyosarcoma sarcomas are clearly myogenic and indistinguishable from embryonal rhabdomyosarcoma based on patterns of gene expression. Forty-eight selected prognostic genes predict highly favorable (94% 5-year survival), intermediate (75%), and unfavorable (22%) survival cohorts of patients; even a four-gene "sarcoma signature" performs nearly as well as current risk stratification schemes. Prognostic genes are highly enriched for MAP kinase signaling pathways, suggesting a mechanism for aggressive vs. indolent tumors and possible therapeutic targets for future drug development.

**KL21**

**Monitoring treatment and follow-up in bone and soft tissue sarcomas**
H.-J. Van der Woude
*(Department of Radiology, Onze Lieve Vrouwe Gasthuis, Amsterdam and Leiden University Medical Center, Leiden, The Netherlands)*

Modern imaging plays a pivotal role in the treatment strategy of patients with high-grade bone and soft tissue sarcoma. Various imaging modalities have been used to monitor the effects of neoadjuvant chemotherapy in osteogenic and Ewing's sarcoma and to evaluate isolated limb perfusion in high-grade STS. Currently, techniques that focus on therapy-induced changes in tumorvascularisation and metabolism are most successful in predicting histologic response. Dynamic contrast-enhanced magnetic resonance (MR) imaging in particular allows detection of and differentiation between residual viable tumor, granulation tissue, edema and necrosis. A benefit of fast gradient-echo sequences is that the entire tumor volume can be evaluated with high spatial and temporal resolution, opposed to single or double histopathologic section examination which serves as gold standard for final response. MRI as such aids in directing surgical strategy and adjuvant treatment. Native and static contrast-enhanced MR sequences are optimal for preoperative assessment of tumor dimensions, margins and relationship to viable structures and joints, but not for response determination. Only an increase of tumor volume after neoadjuvant therapy is indicative for poor histologic response. FDG-PET imaging with or without CT application has been found another promising tool with which to predict the outcomes of patients with high-grade extremity soft tissue sarcomas treated with chemotherapy.

**KL22**

**EURAMOS 1: A model for the future of clinical research in osteosarcoma.**
J. Whelan
*(UCLH, London, UK)*

Randomised studies with sufficient power to define improvements in survival or benefits of new treatments are difficult to conduct in rare cancers. Recent trials for osteosarcoma have been hampered by long accrual times and few new therapeutic interventions. EURAMOS 1 is an international randomised study for patients with resectable osteosarcoma which is the result of co-operation between 4 clinical study groups in Europe and the United States. This trial may prove to be a paradigm for future investigation in osteosarcoma and other rare cancers. The advantages of this joint venture are: rapid accrual times, harmonising understanding of osteosarcoma and its treatment and potentially rapid improvements in knowledge of osteosarcoma biology. Meeting current clinical trial regulatory requirements may be achieved more effectively by greater co-operation between study groups. Finally, as the costs of clinical trials soars, responsibility lies with investigators to ensure that only scientifically valid, viable trials are undertaken. However, recognition of both the importance of such studies and the difficulties associated with their execution is required by funding bodies and regulatory authorities to ensure that investigators are suitably supported.

**KL23**

**Surgery for soft tissue sarcoma**
H.C.F. Bauer
*(Department of Orthopedics, Karolinska, Stockholm, SE-171 76 Stockholm, Sweden)*

Surgery remains the most important part of the treatment of patients with soft tissue sarcoma. In approximately half of all soft tissue sarcoma patients control of the disease will be achieved by surgery. Adjuvant radiotherapy has been proven to improve the local control rate in combination with surgery. Although the adjuvant chemotherapy is increasingly used in soft tissue sarcoma the response rate is low and there is no real convincing evidence that adjuvant chemotherapy improves outcome to any large extent.

Limb sparing surgery can be achieved in most patients, and less then 10% have primary amputations, although the amputation rate can increase after local recurrence. The surgical classification system introduced by Enneking, where intralesional or marginal surgery is considered inadequate, and wide or compartmental margin is considered adequate, is in my opinion the most useful for soft tissue sarcoma of the trunk wall or extremities. Clearly, it is not applicable for intraabdominal or retroperitoneal sarcomas.

Maybe the most important factor for achieving good local control rates is to improve patient so that patients are referred to a sarcoma center before surgery. It is not enough to show good local control rates for patients treated at a center, it has to be shown for a whole population area. To highlight the effect of better management on outcome, we have assessed how changes in referral, surgical margins, and use of radiotherapy have influenced outcome in soft tissue sarcoma.

*Methods:* 869 adult patients with soft tissue sarcoma of extremities or trunk wall were referred 1986–2004. For comparison we have chosen three time periods: early (1986–90), middle (1991–97), and late (1998–2004).

*Results:* Referral of patients before open biopsy or excision improved from 64% to 66% and to 76% during the late period. A wide surgical margin was achieved in 54%, 68% and 61%, respectively. Radiotherapy was increasingly applied in the late period, from 29% to 42%. In assessment of outcome only patients without metastases and operated for primary tumor at the sarcoma center were considered. The overall 5 years local control rate (Kaplan-Meier) was 0.82, and for the three time periods 0.67, 0.83, and 0.88. The 5 years metastases-free survival rate was 0.69, and for the three time periods 0.58, 0.71, and 0.73.

*Conclusions:* These results show a striking improvement in local control and survival in soft tissue sarcoma. The improvement was mostly a result of improved referral, but also better margins and increased use of radiotherapy. Whether local recurrence leads to metastases, or is only an independent sign of malignant behavior, remains controversial. Clearly better local control at least coincided with better survival, and also leads to less surgery for recurrence and to less morbidity.

### KL24

**MRI in sarcoma: Essential and advanced imaging**

P. Winkler
*(Radiologisches Institut, Olgahospital, Stuttgart, Germany)*

MRI is the method of choice in imaging of primary, recurrent or treated tumors, combined with plain x-ray in malignant bone tumors. High resolution whole body imaging might become the standard for evaluation of metastatic disease and imaging of some complications of therapy in the future.

There are three essential principles of MRI in soft tissue sarcoma: 1. Fat suppression before and after contrast administration; 2. High physical and contrast in-plane resolution; 3. Lowest achievable volume averaging.

It is my opinion, that fat suppression is mandatory for all MR examinations in sarcoma. Fat has, however, been used as "contrast medium" (T1-weighted non-fat-suppressed sequence for estimation of bone marrow extension in osteosarcoma). Imaging of peripheral nerves, dynamic contrast studies, MR-guided biopsy and whole body MRI are examples of advanced MR-applications. Imaging of peripheral nerves is particularly relevant for surgical planning in soft tissue tumors or for MR-guided biopsy. Whole body MRI might have similar potential as PET-CT, but is considerably less expensive. MR-guided precision biopsy has proven to be an invaluable tool in primary tumors or tumor-like lesions and potential recurrent tumor, particularly in small, deeply situated sites. It might be also used in future treatment of local small metastases.

### KL25

**Going forward: The penalties of success and the tyranny of numbers in childhood sarcomas**

R. Womer
*(Division of Oncology, Children's Hospital of Philadelphia, PA, USA)*

Clinical research in pediatric sarcomas is in a difficult position. Patients are divided into two groups: those with localized rhabdomyosarcomas, osteosarcomas, and Ewing sarcomas, whose prognosis is fairly good; and those with desmoplastic small round cell tumors or disseminated sarcomas, whose prognosis remains bleak. The first group has a good supply of patients but few of the "events" that make clinical studies statistically feasible, while the second group has few patients, though most of them have events. Rapidly accumulating biologic insights may eventually lead to new treatments, but they also can make subclassification irresistible, and thus make therapeutic studies more difficult. There is a paucity of new therapeutic ideas, and even fewer that have been tested adequately in pilot studies. Thus there has been little therapeutic progress in either group in a decade. Changes in our attitudes toward clinical trials, as well as changes in the conception, design, and coordination of clinical and translational research may aid us in resuming therapeutic progress.

### KL26

**Local control of sarcomatous pulmonary metastases**

A.A. Vaporciyan[1], J.B. Putnam Jr[2]
*([1]MD Anderson Cancer Center, Houston, Texas, USA, [2]Vanderbilt University Medical Center, Nashville, Tennessee, USA)*

Isolated and resectable pulmonary metastases (PM) from sarcoma represent a unique biology between the patient and the tumor. However, resection alone for treatment of sarcomatous PM will fail in a significant number of patients. Various prognostic indicators (potential for complete resection, fewer numbers of metastases, adequate pulmonary reserve, etc.) and molecular characteristics may assist the clinician in selecting patients who will have the best potential for improved survival. These indicators may better define the biological nature of the metastases, and provide an estimate of post-resection survival. Use of neoadjuvant or adjuvant therapy strategies may allow for prolonged survival or cure

Other novel therapies such as regional delivery of therapeutic agents to the lung (isolated lung perfusion) may provide better and more directed therapy for PM. Image guided percutaneous radiofrequency ablation, although potentially less traumatic than thoracotomy, may fail to identify, or treat occult PM frequently found by manual palpation of the lung.

Today, the benefits of complete resection of sarcomatous pulmonary metastases represents a unique and serendipitous occurrence where the host biology, spread of tumor, response to chemotherapy, and resection, render the patient disease free. The best results of treatment of sarcomatous PM await improved biological and molecular therapies.

### Oral

### O01

**Co-ordinated oncogenic transformation and inhibition of host immune responses by the PAX3-FKHR fusion oncoprotein**

S. Nabarro[1,3], N. Himoudi[1,3], A. Papanastasiou[1,3], K. Gilmour[2], A. Thrasher[2], M. Blundell[2] M. Hubank[1], N. Sebire[3], H. Rees[1], G. Canderan[1], S. Gibson[3], J. Anderson[1]
*(Units of [1]Molecular Haematology and Cancer Biology, and [2]Molecular Immunology, Institute of Child Health, London*

*WC1N 1EH., [3]Department of Histopathology, Great Ormond Street Hospital for Children, London)*

Tumours have evolved elaborate mechanisms of evading immune detection; for example, production of immuno-inhibitory cytokines and down-regulation of MHC expression. We have studied PAX3-FKHR as an example of an oncogenic fusion protein associated with aggressive metastatic cancer. We have shown that PAX3-FKHR, as well as stimulating transcription from its own target promoters, interferes with normal interferon gamma signalling in tumour cells by means of specific interaction with the STAT3 transcription factor. This causes a dramatic reduction in tumour cells MHC expression and an altered expression of a number of cytokines and chemokines. For example PAX3-FKHR downregulates the pro-inflammatory cytokines CCL5 (RANTES) and IP10 that have been shown to be inhibited by STAT3, and it upregulates the known STAT3 targets CXCR4 and IL10. Conditioned media from cells expressing PAX3-FKHR has increased IL10 concentration which is reversed by a specific STAT3 blocker and inhibits dendritic cells maturation in vitro and in tumour xenografts, also in a STAT3 dependent manner. PAX3-FKHR also causes an increase in rate of growth of xenografted tumours in immunodeficient mice associated with decreased numbers of tumour infiltrating inflammatory cells, and despite no differences in growth characteristics in vitro between PAX3-FKHR expressing and non-expressing isogenic cell lines. Supported by Cancer Research UK and SPARKS)

### O02

**Significance of persistent rhabdomyoblasts in bladder prostate rhabdomyosarcoma: Results from the fourth intergroup rhabdomyosarcoma study**

C. Arndt[1], S. Hammond[2], D. Rodeberg[1], S. Qualman[2]
*([1]Departments of Pediatric and Adolescent Medicine and Pediatric Surgery, Mayo Clinic, Rochester Minnesota, USA, [2]Department of Laboratory Medicine, Children's Hospital, Columbus, Ohio, USA)*

*Purpose:* To determine the significance of residual rhabdomyoblasts(RmyoB) in bladder prostate rhabdomyosarcoma (BPRMS).
*Patients/methods:* 49 of 88 patients with BPRMS treated on IRS-IV had at least one "second look operation" (SLO) with tissue removal for evaluation (66 procedures in 49 patients). Slides from SLO's were examined for viable tumor (POS), no tumor (NEG), or RmyoB only. SLO's performed for documentation of clinical relapse were excluded. Results of slide review were compared to results obtained from chart review, and correlated with outcome.
*Results:* Slides were obtained from 41 patients (53 procedures). For 15 procedures, central review and institutional pathology differed. Of 16 patients POS at last evaluation, 7 failed (3 local, 1 distant, 3 second malignant neoplasm (1 each osteosarcoma and leiomyosarcoma in radiation field, 1 CNS glioma). Of 11 patients with RmyoB at last evaluation 1 failed (local and distant); 3 underwent cystectomy/cystoprostatectomy despite RmyoB only. Of 14 NEG 2 failed (1 toxic death, 1 local and distant).
*Conclusions:* RMyoB often occur at SLO for BPRMS. Persistence of RMyoB is not necessarily associated with tumor recurrence and is not an indication for radical surgery. Because RMyoB are difficult to discern from viable tumor, review by experienced pathologist is indicated.

### O03

**New biological markers predicting outcome in Ewing family of tumors**

S. Avigad[1,2], A. Ohali[1], I.J. Cohen[2], S. Ash[2], Y. Goshen[2], I. Meller[3], Y. Kollender[3], J. Issakov[3], R. Zaizov[1,2], I. Yaniv[1,2]
*([1]Molecular Oncology, Felsenstein Medical Research Center, [2]Pediatric Hematology Oncology, Schneider Children's Medical Center of Israel, Petah Tikva, Israel, [3]Sourasky Medical Center, Tel Aviv, Sackler Faculty of Medicine, Tel Aviv University, Tel Aviv, Israel)*

*Purpose:* In spite of advances in multimodal therapy, late relapses of Ewing's Sarcoma (ES) are frequent. Currently accepted prognostic factors fail to classify ES patients' risk to relapse at diagnosis. Our aim was to search for novel, reliable prognostic parameters.
*Methods:* Monitoring the chimeric transcripts and telomerase activity at diagnosis and during follow up in samples from 26 patients, and by gene expression profiling of 20 tumors from patients with localized disease.
*Results:* During follow up, but not at diagnosis, a highly significant correlation between the presence of the chimeric transcript, high telomerase activity and progression was detected ($p = 0.007$ and $p < 0.0001$, respectively). Molecular recurrences were identified prior to overt clinical recurrence.

We identified two distinct gene expression signatures distinguishing high risk ES patients that are likely to progress from low risk patients with long term progression free survival. The microarray-based classification was superior to currently used prognostic parameters.
*Conclusion:* The presence of tumor cells or high telomerase activity in BM/PBL are significant prognostic markers of progression – during follow up. Gene expression profile could identify high and low risk patients already at diagnosis.

This may be useful in designing better risk group stratified clinical protocols in ES.

### O04

**A single nucleotide polymorphism in the MDM2 promoter accelerates tumor formation in sporadic human soft tissue sarcomas**

P. Würl[1], H. Taubert[2], G. I. Bond[4], W. Hu[4], H. Robinson[3], G. Lozano[5], A. Levine[3], F. Bartel[2]
*([1]Surgical Clinic I, Faculty of Medicine, University of Ulm, Ulm, Germany, [2]Institute of Pathology, University of Halle-Wittenberg, Halle/Saale, Germany, [3]School of Natural Sciences, Institute for Advanced Study Princeton, New Jersey, USA, [4]Cancer Institute of New Jersey, University of Medicine and Dentistry of New Jersey, New Jersey, USA, [5]Section of Cancer Genetics M.D. Anderson Cancer Center, University of Texas, Houston, Texas, USA)*

In a subset of tumors – especially in soft tissue sarcomas (STS) – overexpression of MDM2 was found exclusive to p53 mutation connected with negative prognostic relevance, which could suggest that overexpression of MDM2 can substitute for inactivating p53 by mutation.

We have shown that a single nucleotide polymorphism (SNP309) is found in the MDM2 promoter. It increases the affinity of the transcriptional activator Sp1, resulting in higher levels of MDM2 RNA and protein and the subsequent attenuation of the p53 pathway.

105 patients who developed sporadic adult STS without hereditary cancer predisposition and no germline p53 mutation were studied. Interestingly, independent of the histological tumor type SNP309 associates with an earlier age of onset of STS. Specifically, those individuals homozygous for SNP309 were diagnosed on average 12 years earlier than those individuals without SNP309 ($p = 0.01$). The frequency of the SNP309 G allele was greatly increased in those individuals who developed STS at a young age. Those who developed STS below the age of 41 years old had an allele frequency for SNP309 of 50%, while the allele frequency is only 33% for the whole group ($p = 0.0262$). These data demonstrate that SNP309 is associated with earlier formation of sporadic STS in human adults.

### O05

**Improved outcome in soft tissue sarcoma through better management**

H.C.F. Bauer, O. Brosjö, R. Wedin, M. Szeps
*(Departments of Orthopedics and Oncology, Karolinska Hospital, Stockholm, Sweden)*

We have assessed how changes in referral, surgical margins and radiotherapy have influenced outcome in soft tissue sarcoma.

869 adult patients with soft tissue sarcoma of extremities or trunk wall were referred 1986–2004. For comparison we have chosen three time periods: Early (1986–90), middle (1991–1997), and late (1998–2004).

Referral of patients before open biopsy or excision has improved from 64% to 66% and to 76% during the late period. A wide surgical margin was achieved in 54%, 68% and 61%, respectively. Radiotherapy was increasingly applied in the late period, from 29% to 42%.

In assessment of outcome only patients without metastases and operated for primary tumor at the sarcoma center were considered. The overall 5 years local control rate (Kaplan-Meier) was 0.82, and for the three time periods 0.67, 0.83, and 0.88. The 5 years metastasis-free survival rate was 0.69, and for the three time periods 0.58, 0.71 and 0.73.

These results show a striking improvement in local control and survival in soft tissue sarcoma. The improvement was mostly a result of improved referral, but also better margins and increased use of radiotherapy.

### O06

**Major organ toxicities after sarcoma therapy according to the GPOH therapy trial protocols prospectively evaluated in 760 children and young adults**

J.D. Beck[1], M. Paulides[1], W. Stöhr[1], S. Bielack[2], H. Jürgens[3], J. Treuner[4], T. Langer[1]
*([1]LESS Centre, University Hospital for Children and Adolescents, Department of Paediatric Oncology, Erlangen, Germany, [2]Osteosarcoma Trial Centre, University Children's Hospital Muenster, Department of Paediatric Haematology and Oncology, Muenster, Germany, [3]Ewing's Sarcoma Trial Centre, University Children's Hospital Muenster, Department of Paediatric Haematology and Oncology, Muenster, Germany, [4]Cooperative Soft Tissue Sarcoma Study, Olgahospital, Department of Paediatric Oncology, Stuttgart, Germany)*

*Background:* Sarcoma treatment incurs a variety of organ-specific toxicities.
*Patients and methods:* The Late Effects Surveillance System (LESS) of the German Society for Paediatric Oncology and Haematology (GPOH) prospectively monitors late effects of antineoplastic treatment in patients treated for Ewing's, osteo- or soft tissue sarcoma within one of the therapy trials of the GPOH. The aftercare is carried out according to the published guidelines of LESS, using examinations that are sensitive for the detection of major toxicities and are efficient within the setting of a vertical network.
*Results:* The cumulative incidences of studied major toxicities in our relapse-free collective of paediatric sarcoma patients are: Anthracycline-induced clinical cardiomyopathy 2.0% (15/760), anthracycline-induced subclinical cardiomyopathy 2.2% (12/548), severe tubulopathy 6.2% (26/418), hearing impairment 52% (39/72).
*Discussion:* Nephrotoxicity and cardiotoxicity are moderate in our collective, while hearing loss in high frequencies (>4 kHz) is more common. Intervention studies are an important step to be taken, in order to develop strategies for the prevention and treatment of such major toxicities.

### O07

**Cooperative Osteosarcoma Study Group trial COSS-96 of intensive, risk-stratified chemotherapy for osteosarcoma**

S. Bielack[1], G. Delling[2], R. Kotz[3], S. Flege[1], B. Kempf-Bielack[1], W.E. Berdel[1], M. Dominkus[3], V. Ewerbeck[4], G.U. Exner[5], U. Göbel[6], N. Graf[7], U. Heise[8], K. Helmke[9], A. Hillmann[10], A.R. von Hochstetter[11], G. Jundt[12], H. Kabisch[13], S. Lang[14], R. Maas[15], P. Reichardt[16], R. Schwarz[17], J. Treuner[18], M. Werner[3], W. Winkelmann[19], A. Zoubek[20], H. Jürgens[1]
*([1]Cooperative Osteosarcoma Study Group (COSS). Departments of Pediatric/Medical Oncology, Universities Münster, [6]Düsseldorf [7]Homburg, [13]Hamburg, [16]Berlin, [18]Olgahospital Stuttgart, [20]St. Anna-Spital Vienna, [2]Institute for Osteopathology, University Hamburg, [3]Departments of Orthopedics, Universities Vienna, [5]Zürich, [4]Heidelberg, [19]Münster, [10]Klinikum Ingolstadt, [8]Orthopedic Practice Hamburg, [9]Departments of Pediatric Radiology, [17]Radiotherapy, [11]University Hamburg; Pathology Practice Zürich, [12]Departments of Pathology, Universities Basel, [14]Vienna, [15]Radiology Practice Hamburg. Germany, Austria, Switzerland)*

*Aims:* To evaluate risk-adapted osteosarcoma chemotherapy with doxorubicin (D), methotrexate (M), and ifosfamide/cisplatin (IP).
*Methods:* Patients <40 years with nonmetastatic high-grade extremity osteosarcoma eligible. Preoperative chemotherapy (1 D, 2 M, and 2 IP) identical for all. Risk groups stratified postoperatively by tumor volume and histologic response.
*Results:* 442 eligible patients, 393 of these risk-stratified. Median follow-up 3.6 years for all patients, 4.0 years for 373 survivors. Actuarial outcome at 4 years:

| Group | Postop chemo | *n* | Survival | Event-free |
|---|---|---|---|---|
| All patients | | 442 | 84% | 70% |
| *Not stratified* | | *49* | *71%* | *53%* |
| Stratified | | 393 | 85% | 71% |
| Lower risk | 1 D, 1 IP, 4 M | 65 | 87% | 77% |
| Standard risk | *Randomized comparison S1 vs. S2* | | | |
| *S1* | *3 D, 2 IP, 8 M* | *156* | *88%* | *72%** |
| *S2* | *2 D, 1 IP, 10 M* | *157* | *85%* | *71%** |
| High risk | 1 D, carboplatin + etoposide × 5 | 15 | 52% | 41% |

*Tendency towards late events beyond 4 years for S2 vs. S1: 5/62 vs. 1/56.

*Conclusions:* Four-drug chemotherapy is highly effective against osteosarcoma. A long treatment duration may be needed even for presumed low-risk patients. The outcome of high-risk patients remains poor despite salvage treatment. A longer follow-up is required to determine if S1 and S2 do equally well.

### O08

**The EpSSG RMS2005: A new stratification for the first pan-European protocol for localised rhabdomyosarcoma**

G. Bisogno, A. Mattke, E. Koscielniak, C. Bergeron, M. Jenney, R. Knietig, I. Brecht, S. Gallego, A. Schuck, H. Martelli, A. Kelsey, R. Alaggio, A. Rosolen, A. Ferrari, M. Carli, O. Oberlin, M. Stevens, J. Treuner
*(EpSSG)*

*Introduction:* The three Cooperative European Groups: The SIOP MMT Committee, the CWS and the AIEOP STSC recently decided to join together as the European paediatric Soft tissue sarcoma Study Group (EpSSG). As first step a common analysis has been performed on localized rhabdomyosarcoma to identify prognostic factors.
*Methods:* An analysis carried out by the CWS group using the CWS/RMS 96 data and validated with data from studies with longer follow up: The SIOP MMT-84 and 89, the German CWS-81, 86 and 91 and the Italian RMS-79 and RMS-88 studies identified the following significant prognostic factors:

- histology (alveolar vs. embryonal),
- post surgical status (IRS Group),
- tumour site,
- node involvement (N0 absent, N1 present),
- tumour size (> or $\leq$5 cm),
- patient's age (unfavourable if $\geq$10 years)

Combining these factors 8 subgroups of patients have been identified:
Subgroup A: eRMS, IRS I, Any site, N0, favourable age&size
Subgroup B: as subgroup A but with unfavourable age or size
Subgroup C: eRMS, IRS II/III, favourable site, N0, any age or size
Subgroup D: eRMS, IRS II/III, unfavourable site, N0, favourable age&size
Subgroup E: eRMS, IRS II/III, unfavourable site, N0, unfavourable age or size
Subgroup F: eRMS and N1
Subgroup G: aRMS, excluding N1
Subgroup H: aRMS and N1

Four risk groups have been identified with different estimated 3-yrs EFS:

- Low risk: Subgroup A (EFS 90%)
- Standard risk: Subgroup B, C, D (EFS 70–80%)
- High risk: Subgroup E, F, G (EFS 50–55%)
- Very high risk: Subgroup H (EFS 30–40%)

*Conclusions:* Using data from across Europe 4 risk categories for localized RMS have been identified. Objectives and treatment plans have been elaborated for each group in the EpSSG RMS 2005 protocol.

### O09

**Treatment of adult type sarcomas: The EORTC Soft Tissue and Bone Sarcoma Group (STBSG) experience**

J.-Y. Blay
*(Centre Leon Barard, Lyon, France)*

*Historic background:* The EORTC STBSG was founded in 1976. The founding members came from Italy, France, Germany, Belgium, Switzerland, UK and the Netherlands.
*Organization:* The objectives were to develop, stimulate and co-ordinate studies on all aspects of the treatment of soft tissue sarcomas (STS) as well as to organise congresses, symposia and conferences to promote these studies. The Group has developed very strict quality control procedures and played a major role in the development of the RECIST criteria. The quality assurance program involves a strict membership policy, central review of responses, central review of pathology, use of a systemic therapy checklist and on site monitoring visits. Today the STBSG has members from 40 institutions from 13 countries.
*Types of studies:* The activities have primarily been within the areas of standards for local and systemic treatment strategies especially in advanced STS. So far, more than 45 clinical trials have been conducted and more than 200 pts per year have been included. A unique database with over 2500 patients has been developed and based on this a number of important studies have been published on prognostic factors, long term survivors and new response criteria. Several new drugs have been tested in STS but doxorubicin and ifosfamide are still the most effective agents. Other drugs with some first line activity are dacarbazine, caelyx and possibly ET743. Imatinib is very effective in GIST where it is now the treatment of choice – a development in which the group has played a very important role. Several phase 3 trials have also been performed on both single agent and combination chemotherapy. The group has also participated in an osteosarcoma intergroup study. Recently a phase 3 study on adjuvant doxorubicin and ifosfamide in STS has been closed and awaits further analysis. Presently the group is performing studies on caelyx + ifosfamide, high dose ifosfamide + doxorubicin, hyperthermia, exatecan, brostallicin, and gefitinib (Iressa). The group also participates in the EE99 study of Ewing's/PNET. Finally studies on translational research have been initiated, including a study of the molecular biology of GISTs.
*Intergroup co-operations:* An increasing number of studies have recently been performed in collaboration with other groups such as SWOG, ECOG, NCIC, AGITG, FSG, ISG and SSG.

### O10

**Why are age and size the most important risk factors for further improvement of the risk stratification for high risk rhabdomyosarcomas in children and adolescents?**

I.B. Brecht[1], C. Int-Veen[1], R. Knietig[1], A. Mattke[1], S. Kirsch[1], T. Dantonello[1], A. Schuck[2], T. Klingebiel[3], G. Bisogno[4], M. Carli[4], E. Koscielniak[1], J. Treuner[1]
*([1]CWS study group, Olgahospital Stuttgart, Germany, [2]Radiotherapy Department, University Hospital Munster, Germany, [3]Department of Paediatric Oncology and Haematology, University Frankfurt, Germany, [4]Division of Pediatric Hematology and Oncology, Padua University, Italy)*

*Objectives:* To find possible reasons for the influence of age and size being found to be the most important factors for further improvement of the risk stratification for children and adolescents with RMS.
*Patients/methods:* 1140 patients (CWS 81, 86, 91, 96 studies) were stratified according to the CWS-96 study. 779 defined high risk patients were analysed by COX-regression. Age ($p<0.001$) and size ($p<0.001$) were identified as additional factors influencing prognosis significantly and were secondly correlated with tumour and patients characteristics.
*Results:* 5-yr SUR (EFS) was in patients <1 yr 62% (43%), 1–5 yrs 74% (65%), 6–10 yrs 75% (66%), 11–15 yrs 67% (52%), 16–21 yrs 52% (47%). More RMA, tumours >5 cm, and nodal involvement were found in patients >10 yrs. The high number of events in large tumours (>5 cm) is especially seen in patients >10 yrs (26% metastatic relapses), but also in patients <1 year old (24% progression). Patients <10 yrs are less radically operated, received lower doses of radiotherapy and preoperative chemotherapy was preferred. There is no better tumour response seen in younger patients (1–10 yrs) in comparison to patients >10 yrs.
*Conclusions:* Though older patients receive a more intensive treatment they show a worse outcome than younger patients having a second chance after relapse. As we see more alveolar histology, more nodal involvement, larger tumours and more metastatic relapse in older patients the reason may lie in a different tumour biology which expression is still not found.

### O11

**Pyridinoline cross-links: Markers for primary and secondary bone tumors**

J. Bruns[1], P. Behrens[2], K.P. Ullrich[1], Y. Açil[3]
*([1]Department of Orthopaedic Surgery, University of Hamburg, Germany, [2]Department of Orthopaedic Surgery, University of Schleswig-Holstein, Campus Luebeck, Germany, [3]Department of Macillo-Facial-Surgery, University of Schleswig-Holstein, Campus Kiel, Germany)*

*Introduction:* Determination of hydroxylysylpyridinolines (HP) and lysylpyridinolines (LP) in urine is a promising method to determine bone resorption. This method is independent from gender, diet, and kidney function (creatinine clearance >25 ml/min).
*Material & methods:* We evaluated the value of HP and LP in urine from adult tumor patients suffering from primary malignant bone tumors ($n=24$), bone metastases ($n=38$) and soft tissue sarcoma with additional osseous involvement ($n=13$). The values were compared with those obtained from 698 healthy controls (0.5 to 65 years old).
*Results:* Clearly exhibited a significant increase in HP values (57.75 nmol/mmol creatinine) in adult tumor patients (aged 15 to 65 years) of all three subgroups in comparison to the control group (22.23 nmol/mmol creatinine). Although LP is more specific for bone than HP, the values of LP from all subgroups of the adult tumor patients were less distinctly but significantly increased. In the HP:LP ratio patients exhibited a distinctly increased molar HP:LP ratio (12.0:1) in comparison to controls (6.6:1). Linear correlation analysis between HP and LP resulted in $r=0.98$ for controls and $r=0.8$ for tumor patients.
*Conclusion:* Determination of HP and LP in urine obtained from adult tumor patients suffering from malignant primary bone tumors, metastases to bone and osseous involvement in cases of soft tissue tumors is a useful marker for bone resorption and can be indicative for an osteolytic bone tumor. Further studies have to demonstrate whether determination of HP, LP and its ratio can be indicative for a relapse of the tumor.

### O12

**Improvement of local tumor control in retroperitoneal soft tissue sarcomas (RSTS) – radiotherapy**

W. Budach
*(Department of Radiation Oncology, University Hospital Düsseldorf, Germany)*

Most patients with RSTS are diagnosed with tumors larger than 10 cm. Complete resections are achieved in less than 50% of the cases. Local recurrences occur in 30–60% of cases even after complete resections. Distant metastasis are infrequent in liposarcomas, the most frequent histological subtype of RSTS. Therefore, many patients die from complications of the local tumor. External beam radiation therapy (EBRT) is an effective pre- and postoperative treatment in soft tissue sarcomas of the extremities. The vicinity of relatively radiation sensitive organs like small bowel and kidneys compromise the administration sufficient radiation doses in RSTS. However, retrospective data indicated some benefit from moderate dose EBRT. Since better sparing of sensitive tissues can be achieved, when the tumor is still in situ, preoperative RT appears advantages. The use of intensity modulated radiotherapy (IMRT) will markedly improve the ability to administer higher doses to RPSTS resulting in better local tumor control. Intraoperative electron beam radiotherapy (IOERT) allows to keep sensitive tissues outside the portals. A randomised trial comparing IOERT in RSTS as boost before EBRT with EBRT alone ($n=87$) showed a clear benefit of IOERT in terms of local tumor control. RSTS remain a interdisciplinary therapeutic challenge.

### O13

**ICS-CWS RMS 96 – Early results of the Italian and German Rhabdomyosarcoma (RMS) Cooperative Study Groups**

M. Carli[1], E. Koscielniak[2], G. Bisogno[1], A. Mattke[2], A. Ferrari[1], T. Dantonello[2], L. De Sio[1], I. Brecht[2], G. Cecchetto[1], G. Sotti[1] C. Int-Veen[2], V. Ninfo[1], J. Treuner[2]
*([1]AIEOP Soft Tissue Sarcoma Committee, Italy, [2]CWS Soft Tissue Sarcoma Study Group, Olgahospital, Stuttgart, Germany)*

*Purpose:* The ICS-CWS RMS 96, was addressed to children <18 yrs with localised RMS.
*Methods:* Treatment based on risk categories according to histology, tumor site, lymph node (N) involvement, IRS group (Gr). Chemotherapy (CT): "low risk"; "standard risk"; "high risk": VAIA randomized vs. CEVAIE. Delayed surgery after CT in Gr III. Radiotherapy: 32–45 Gy (pts in clinical CR or with residual disease or ARMS). Between 1996 and 2003, 797 eligible pts entered the study. Characteristics: sex: M 62%; age 74% <10 yr; site: orbit 10.7%, head-neck non PM 7,4%, PM 22%, GU BP 11.8%, GU non BP 19.6%, extremity 10.7%, other 17.7%. Histology: alveolar 20.3%. Tumor size: >5 cm 49%; N1 15%. IRS Group: I 16%, II 16%, III 68%. Risk group: Low 10%, standard 22%, high 68%.
*Results:* 3-yr PFS and OS are 68.3% and 80.9% respectively. According to risk group: Low risk 89.6% and 96.8%; standard risk 80.3% and 96.1%; high risk 61.4% and 73.9%.
*Conclusions:* Low risk pts: Very good OS with an alkylating-free regimen. Standard risk pts: Very good OS utilising a less aggressive treatment (9 vs. 12 cycles and no Doxo) in comparison CWS 91 and ICS 88. High risk pts: No improvement of OS and PFS.

### O14

**The orphan nuclear receptor DAX1 is expressed in Ewing tumours and upregulated by the EWS/FLI1 oncoprotein**

J. Carrillo[1], M. Mendiola[1], E. García[1], E. Lalli[2], E. de Alava[3], P. García-Miguel[4], F. López-Barea[5], S. Gallego[6], D.I. Segura[7], I. González[8], A. Navajas[9], A. Pestaña[1], J. Alonso[1]

*([1]OncoLabiib, Instituto de Investigaciones Biomédicas (CSIC-UAM), Madrid, Spain, [2]Institut de Pharmacologie Moléculaire e Cellulaire CNRS, Sophia Antipolis, Spain, [3]Laboratorio de Patología Molecular, Centro de Investigación del Cáncer (USAL-CSIC), Salamanca, Spain, [4]Unidad Oncohematología Infantil, Hospital Infantil La Paz, Madrid, Spain, [5]Dpto. Anatomía Patológica, Hospital La Paz, Madrid, Spain, [6]Unidad Oncología Pediátrica, Hospital Vall d'Hebron, Barcelona, Spain, [7]Dpto. Anatomía Patológica, Hospital Virgen del Rocio, Sevilla, Spain, [8]Dpto. Anatomía Patológica, Hospital Niño Jesús, Madrid, Spain, and [9]Unidad Oncología Pediátrica, Hospital de Cruces, Bilbao, Spain)*

Tumours of the Ewing family are sometimes difficult to classify because of the highly similar appearance in routine histology with other small round cell tumours, such as rhabdomyosarcoma. To identify new marker genes of these tumours, we analysed 22 Ewing's sarcomas (ES) and 10 rhabdomyosarcomas (RMS) by CodeLink oligonucleotides microarrays (Uniset I Human 20 K). Genes specifically expressed for each tumour type were identified by the statistical method Significance Analysis of Microarrays (SAM). A total of 56 ES and 67 RMS specific genes were identified. Amongst them, we focused on DAX-1 (NR0B1), an unusual orphan nuclear receptor involved in gonadal development, sex determination and steroidogenesis. Quantitative real time RT-PCR, western-blot analysis, and immunohistochemistry demonstrated that DAX1 was expressed in Ewing cell lines and tumours, but not in neuroblastoma or embryonal rhabdomyosarcoma. In agreement with the transcriptional repressor activity characteristic of DAX1, its expression was restricted to the nucleus of Ewing tumour cells. We also analysed if DAX1 expression was upregulated by the EWS/FLI1 fusion oncoprotein characteristic of Ewing tumours. Using two unrelated cell systems, we demonstrated that DAX1 mRNA and protein were upregulated by EWS/FLI1, but not by native FLI1. These data show that DAX1 could be a new target of the EWS/FLI1 oncoprotein and open the possibility of its use as a Ewing molecular marker in clinical routine diagnostic, at least in the differential diagnosis with neuroblastoma and embryonal rhabdomyosarcoma.

### O15

**The correlation between dose of follinic acid and neurotoxicity in children and adolescents treated for osteosarcoma with high dose methotrexate (HDMTX): A neurophysical and psychosocial study**

E. Boneda-Shkedi[1,2], D. Hoofien[2], M. Ben Arush-Weyl[3], C. Kaplinsky[4,5], S. Ash[1], Y. Goshen[1], Y. Yaniv[1,5], I.J. Cohen[1,5]

*([1]Department of Hematology Oncology Schneider Children's Medical Center of Israel, [2]Hebrew University of Jerusalem, Department of Psychology, Israel, [3]Myer Children's Hospital Rambam Hospital, Israel, [4]Tel Hashomer Hospital, Israel, [5]Sackler School of Medicine, Tel Aviv University, Israel)*

*Introduction:* Leukoencephalopathy occurs following HDMTX with low dose follinic acid but can neurotoxicity be prevented with adequate doses?
*Objectives:* To correlate follinic acid dose after MDMTX and psychological and psychosocial status. 23 osteosarcoma patients from three medical centers were evaluated 6 months – 20 years after therapy. 12 patients received 600–800 mg (one received 450 mg) follinic acid (HDFA) and 11 patients received 120–220 mg (LDFA).
*Methods:* 23 patients underwent extensive assessment including neuropsychological measures of attention, working memory, visual perception, verbal memory, visual memory, learning, executive functions, comprehension, psychosocial measures of psychiatric status, self-esteem, quality of life and social functioning. Both groups were compared using parametric and non-parametric tests in the relevant distributions and using the bonferroni method.
*Results:* 10 out of 18 equations in the neuropsychological assessment significantly favored the (HDFA) group. No psychosocial assessment showed a significance. Psychiatric status and the social functioning were insignificantly better in the (HDFA) group. Two measures of quality of life and a measure of self esteem were insignificantly higher in the (LDFA) group.
*Conclusions:* The (HDFA) group had a better neuropsychological status. These findings have wider relevance since the progressively lower follinic acid doses used in leukaemia protocols may increase neurotoxicity.

### O16

**PET scanning in the orthopaedic population**

E.U. Conrad III[1], H.D. Morgan[1], D. Hawkins[2], C. Vernon[3], J.F. Eary[3]

*([1]University of Washington Orthopaedics and Sports Medicine, Washington State, USA, [2]Children's Hospital and Regional Medical Center, Washington State, USA, [3]University of Washington, Nuclear Medicine, Washington State, USA)*

*Introduction:* Anatomic imaging modalities do not accurately assess response of soft-tissue sarcomas to preoperative radiotherapy and/or chemotherapy. [F-18]-fluorodeoxy-D-glucose (FDG) positron emission tomography (PET) predicts histopathologic response to therapy. We use FDG PET to correlate between change in FDG uptake and histopathologic response to preoperative doxorubicin-based chemotherapy, risk of recurrence and overall survival in patients with soft-tissue sarcomas. New pilot data will also be included for total-joint and allograft patients.
*Methods:* Retrospective analysis of thirty-eight patients with intermediate-or high-grade, localized soft-tissue sarcoma evaluated by FDG PET. Maximum standard uptake values (SUVmax) of tumors were measured prior to treatment with doxorubicin-based chemotherapy and prior to surgery. Resected specimens were examined for residual viable tumor using standard pathologic methods. Association between change in SUVmax and residual viable sarcoma was determined by Spearman rank correlation. Patients were followed at least annually for evidence of recurrence and survival. Kaplan-Meier analyses were performed to determine whether the change in SUVmax to chemotherapy correlated with risk of disease recurrence after surgery and survival.
*Results:* The change in SUVmax of FDG inversely correlates with the degree of residual viable tumor after preoperative chemotherapy. Patients whose tumors have 40% or greater decline in SUVmax to chemotherapy are at significantly lower risk of recurrent disease after complete resection, and show a trend toward improved overall survival.
*Discussion/conclusion:* FDG PET is a useful, non-invasive measure of chemotherapy sensitivity of intermediate and high-grade soft-tissue sarcomas. Soft-tissue sarcomas that show response to doxorubicin-based chemotherapy by FDG PET imaging have a lower risk of disease recurrence.

### O17

**Treatment results and prognostic factors in Ewing sarcoma family of tumors in a Brazilian institution**

C.M.L. Da Costa, A. Lopes, P.E. Novaes, B. De Camargo
*(Pediatric Oncology Department, Hospital do Cancer AC Camargo, São Paulo, Brazil)*

The Ewing sarcoma family of tumors (ESFT) comprises a group of neoplasms with aggressive behavior and definition of prognostic factors is necessary to risk adapted therapy. Prognostic factors in ESFT from a Brazilian institution were analyzed. 110 consecutive patients with ESFT treated from 1990–2001 were reviewed. 61% were male and 79% were white. Median age was 12 years (range 3 months–19 years). Pelvic area (25 cases), femur (21 cases) and chest wall (19 cases) were more often primary site. Thirty three patients had detectable metastases at time of diagnosis. There was a significant relationship between pelvic tumor and metastases ($p = 0.002$). Five-year event-free survival (EFS) was better for patients with age less than 12 years old (63% vs. 34% $p = 0.009$), without metastases (66% vs. 12% $p < 0.001$), LDH less than 300 U/L (60% vs. 38% $p = 0.032$), good clinical response (63% vs. 9% $p < 0.001$) and good histological response (70% vs. 29% $p < 0.001$). Metastases (HR = 4.7 CI:5.51–22.61), clinical response (HR = 11 CI:2.36–9.62) and LDH (DHL = 2 CI:1.01–3.93) were prognostic factors in multiple analysis (Cox). EFS was influenced by age, metastases, clinical response LDH, histological response. However only clinical response, LDH, metastases were independent prognostic factors.

### O18

**Initial patient characteristics can predict pattern and risk of relapse in localized rhabdomyosarcoma**

T. Dantonello[1], C. Int-Veen[1], S. Kirsch[1], I. Brecht[1], P. Winkler[1], I. Leuschner[2], A. Schuck[3], B. Schmidt[4], S. Bielack[5], B. Kazanowska[6], R. Ladenstein[7], T. Wiebe[8], T. Klingebiel[9], E. Koscielniak[1], J. Treuner[1]
*(The Cooperative Soft-Tissue Sarcoma Group (CWS): [1]Olgahospital, Stuttgart, Germany, [2]Institute for Paidopathology, University of Kiel, Germany, [3]Departments of Radiotherapy, University of Münster and [4]Katharinenhospital, Stuttgart, Germany, [5]Departments of Pediatric Oncology, Universities of Münster and [9]Frankfurt, Germany, [6]Department of Pediatric Oncology, University of Wroclaw, Poland, [7]St. Anna Kinderspital, Vienna, Austria, [8]Department of Pediatric Oncology, University of Lund, Sweden)*

*Purpose:* Evaluation of primary tumor-, treatment-, and patient-related factors predicting pattern and risk of relapse in patients with localized rhabdomyosarcoma (RMS).
*Methods:* 1164 patients with RMS and a median follow-up of 5 years achieved a complete remission after multimodal therapy in the trials CWS-81, -86, -91, and -96 between 1980 and 2002. 336 of these developed either local (LR) or metastatic (MR) relapse.
*Results:* Age, histology, tumor-size, -site ($p = 0.07$), IRS-group, and radiotherapy were identified in multivariate analysis as significant factors associated with increased relapse risk. Relapse rates did not differ between the CWS-trials. Median time to relapse was 1.43 (0.13–13.5) years. LR ($n = 217$) the most common type of failure. Only 3 of 119 MR occurred >3 years after diagnosis and all those were combined recurrences. Three patient subsets with rather consistent pattern and risk of relapse could be identified depending on the therapy-unrelated factors age, histology, and tumor-size.
*Conclusion:* Initial patient characteristics can predict pattern and risk of relapse. Screening for MR is not justified >3 years after diagnosis. A risk stratification for follow-up could be developed by means of three therapy-independent factors. These results will form the basis of a new risk-adapted follow-up for localized RMS in the CWS-trials.

### O19

**Role of radiotherapy in metastatic Ewing tumors**

J. Dunst[1], A. Schuck[2], S. Burdach[4], H. Jürgens[4], M. Paulussen[4]
*([1]Department of Radiotherapy, University of Halle, Germany, [2]Department of Radiotherapy, University of Münster, Germany, [3]Department of Pediatric Oncology, Technical University of Munich, Germany, [4]Department of Pediatric Oncology, University of Münster, Germany)*

*Purpose:* Primary metastatic disease is the main risk factor in patients with Ewing tumors. We propose the hypothesis that additional local treatment of metastatic sites (especially by radiotherapy) may improve outcome.
*Materials and results:* Bilateral lung irradiation (BLI) is effective in controlling microscopic lung metastases, but BLI was abandoned from treatment protocols with the development of more effective chemotherapy regimens. In the CESS-studies, BLI was a treatment option for patients with lung metastases at diagnosis who achieved a complete clinical response to chemotherapy. A retrospective analysis demonstrated a dose-dependency. In a multivariate analysis, lung irradiation was associated with improved survival in patients with primary lung metastases at diagnosis. Patients with bone metastases (± lung metastases) have a poor prognosis with standard chemotherapy and local therapy. In phase-II-trials, an aggressive approach including local irradiation to clinically involved sites and additional high-dose chemotherapy resulted in long-term remissions in about 40% of patients. Recent progress in diagnosis of metastases (e.g. by PET) and local radiotherapy (e.g. 3D-conformal or stereotactic radiotherapy) might further improve the results.
*Conclusions:* BLI is an effective approach and currently tested in the EURO-EWING-99-study. Local radiotherapy to metastatic bone lesions is an attractive approach and currently tested in ongoing trials.

### O20

**Topoisomerases I and II in high grade paediatric osteosarcomas**

N. Entz-Werle[1], E. Guerin[1], A.-C. Voegeli[1], P. Marec-Berard[2], C. Schmitt[3], M.-D. Tabone[4], C. Kalifa[5], Laurence Brugière[5], Pierre Oudet[1], Annie Babin[1], Marie-Pierre Gaub[1]
*([1]CHRU Hautepierre, Strasbourg, France, [2]Centre Léon Berard, Lyon, France, [3]CHRU Brabois, Nancy, France, [4]Hôpital Trousseau, Paris, France, [5]Institut Gustave Roussy, Villejuif, France)*

*Background:* In the French Society of Paediatric Oncology, patients with high grade osteosarcomas are treated with OS94 protocol, including usually topoisomerase II inhibitors. Antitopoisomerase I drugs were, only, used in phase I studies. Considering these therapeutic targets, our study was designed to investigate, firstly, the status at loci 3p24, 17q21 and 20q11 and, secondly, the candidate genes (TOP2B, TOP2A and TOP1, respectively).
*Patients and methods:* We included 74 children treated in OS94 protocol and systematically classified in good (GR) and poor responders (PR) to neoadjuvant chemotherapy. After DNA extraction, microsatellite analysis was performed in paired blood and biopsy samples at loci close to topoisomerase I, IIa and IIb genes and was followed by QPCR targeting TOP1 and TOP2A comparatively to two different reference genes.
*Results:* Our biopsies were highly rearranged at loci 20q11 (36.9%), 17q21 (47.6%) and 3p24 (69.7%). Since TOP1 was amplified in a half of the rearranged tumours, TOP2A was predominantly deleted (27/34 abnormal cases). Furthermore, all the TOP2A rearranged tumours were significantly correlated with the GR subgroup without any consequence on survival rate.
*Conclusion:* These results showed frequent rearrangements of TOP1 and TOP2A in paediatric osteosarcomas, which their role in oncogenesis should be studied.

### O21

### The role of surgical treatment in malignant vascular tumours in children: A report from the Polish Paediatric Rare Tumours Study

E. Bien, J. Godzinski, A. Balcerska, E. Izycka-Swieszewska, T. Stachowicz-Stencel, M. Rapala, W. Sulka, B. Kazanowska, A. Reich, W. Madziara, M. Perek-Polnik, P. Mankowski, J. Nurzynska-Flak, A. Kurylak, A. Rybczynska, B. Zalewska-Szewczyk, M. Dobruchowska, J. Bodalski, J. Bohosiewicz, A. Chybicka, K. Jaskiewicz, J.R. Kowalczyk, D. Perek, A. Prokurat, J. Wachowiak, M. Wysocki.
*(Oncological centres of the Polish Paediatric Solid Tumours Study Group from Gdansk, Wroclaw, Katowice, Warszawa, Poznan, Lublin, Bydgoszcz, Lodz)*

The Polish Paediatric Rare Tumours Study registered 32 children (1992–2002) with angiosarcoma (ASA-10), haemangioendothelioma (HE-7) and haemangiopericytoma (HP-16) of infantile (7 pts) and adult-type (9 pts).
*ASA:* In 7/10 pts disease was local/regional, 8/10 tumours infiltrated surrounding tissues. All 7 primary excisions (PE) were incomplete. All pts received CHT, 5-radiotherapy. 3/4 secondary resections were complete. 9/10 pts died of relapse/progression.
*HE:* In all disease was local. 2/5 PE were complete. 2 patients received radiotherapy, 4–CHT. In 2 patients secondary excisions were complete. 2/7 patients who had incomplete PE died of disease.
*Infantile HP:* in all disease was local, 3 patients underwent complete PE. 2/7 patients entered CR after CHT alone, 2/7 after post-CHT surgery. All are in CR.
*Adult-type HP:* in all disease was local. 5/6 PE were complete. 5 patients received radiotherapy, 6 CHT. 3 patients with macroscopic residues died of disease.
*Conclusions:* PE value in both HP subtypes and selected HE: 10/14 excisions were complete vs 0/7 in ASA. Post-CHT resections were complete in 7/9 cases improving the outcome in HE and HP, but not in ASA. More efficacious management of ASA should be searched for.

### O22

### Phase I – study: Exclusion of side-effects of the silver-coated tumour endoprosthesis in 20 patients with bone metastasis

G. Gosheger[1], J. Hardes[1], H. Ahrens[1], A. Streitburger[1], C. Gebert[1], A. Gunsel[2], H. Burger[3], F. Kemper[2], W. Winkelmann[1]
*([1]Department of Orthopaedics, University of Muenster, Muenster, Germany, [2]Environmental Specimen Bank for Human Tissues, University of Muenster, Germany, [3]Institute of Pathology, University of Muenster, Germany)*

The deep infection of tumour endoprosthesis is a serious complication (10–20%). The silver-coated tumour endoprosthesis proved their effectiveness in a rabbit model (7% infection versus 47% non-coated); toxicological side-effects could be excluded (Gosheger et al., Biomaterials 2004). A prospective study is conducted including 230 patients in 2 phases: Phase I to exclude side-effects (20 patients with bone metastasis) and secondly prove the effectiveness.

Phase I consists of patients undergoing resection of large metastasis with an impending ($n=7$) or a diagnosed fracture ($n=11$) or an infected osteosynthesis ($n=2$) with progressive metastatic disease. The age ranged from 32–82, the mean follow up was 16 months.

The preliminary results did not show any side-effects related to the silver-coating. Loosening, infection and material failure could not be observed. The laboratory parameters (GOT, GPT, creatinine, CK, etc.) did not show pathologic changes due to the silver-coated endoprosthesis. In two performed sections analysing the implant area no foreign body granuloma were found and the surface was stable.

In summary, Phase I could exclude any side-effect of the silver-coated endoprosthesis up to now so it can be recommended in difficult patients suffering from an infection, in an expected radiation therapy or in cases with problematic soft tissue coverage.

### O23

### Prognostic effect of re-excision in adult soft tissue sarcoma of the extremity

A. Gronchi[1], M. Fiore[1], P.G. Casali[2], L. Mariani[3], R. Miceli[3], P. Collini[4], L. Lozza[5], P. Olmi[5]
*([1]Department of Surgery, Istituto Nazionale per lo studio e la cura dei Tumori, Milano, Italy, [2]Department of Cancer Medicine, Istituto Nazionale per lo studio e la cura dei Tumori, Milano, Italy, [3]Department of Biostatistics, Istituto Nazionale per lo studio e la cura dei Tumori, Milano, Italy, [4]Department of Pathology, Istituto Nazionale per lo studio e la cura dei Tumori, Milano, Italy, [5]Department of Diagnostic Imaging and Radiotherapy, Istituto Nazionale per lo studio e la cura dei Tumori, Milano, Italy)*

*Objectives:* To explore the outcome of patients with primary adult soft tissue sarcoma (STS) of the extremities undergoing re-excision following previous unplanned surgery.
*Materials/methods:* A total of 597 consecutive adult patients with primary extremity STS were treated with conservative surgery at our institution over a 20-year time span. Patients referred after unplanned excisions were 318, while the remaining 279 were primarily resected at our center. The two groups significantly differed as far as size and depth were concerned. The assessed end-points were sarcoma-specific mortality, local recurrence and distant metastasis.

Univariable and multivariable analyses were performed in the competing risks framework.
*Results:* At multivariable analysis, the 10-year cumulative incidences in re-excised and primarily operated patients were, respectively: 18.7 vs. 16.4% ($P = 0.535$) for local relapse, 17.6 vs. 20.2% ($P = 0.541$) for metastatization, 20.4 vs. 22.4% ($P = 0.645$) for mortality. Amongst re-excised patients, evidence of microscopic residual disease on pathologic examination had a significant prognostic impact at multivariable analysis for distant metastases ($P = 0.002$), with a trend on survival detected as well.
*Conclusions:* At a referral center carrying out a liberal policy of re-excisions in adult, primary STS of extremities, the outcome of re-excised patients was similar to primarily resected patients. Evidence of microscopic residual disease at re-excision was a marker of clinical aggressiveness.

### O24

**Overall survival after limb sparing or radical surgery for malignant fibrous histiocytoma (MFH) of the extremity: Analysis of prognostic factors by using SEER data**

M.R. Hamre[1], P.J. Chuba[2], R. Severson[1], D. Lucas[3], M. Mott[1]
*([1]Department of Pediatrics and Orthopedic Surgery, Karmanos Cancer Institute, Wayne State University, Detroit, MI, USA, [2]Department of Radiation Oncology, St. John Health Systems, Detroit, MI, USA, [3]Department of Pathology, University of Michigan, Ann Arbor MI, USA)*

*Purpose:* Limb sparing surgery followed by radiation has emerged as standard management for extremity soft tissue sarcoma. We examined factors associated with overall survival in extremity MFH using NCI SEER program data.
*Patients and methods:* 1043 SEER cases of soft tissue MFH of the extremity (303 upper and 740 lower extremity) were analyzed with median follow-up 5.2 years. Survival analysis used Kaplan-Meier, log-rank test, and Cox proportional hazards models.
*Results/discussion:* By univariate analysis, tumor size, grade, age, SEER Stage, primary site, and type of surgery, were significantly associated with overall survival ($p < 0.0001$). Five year overall survival was 64.8% ($n = 588$) for local or wide resection and 56.9% for amputation ($n = 204$), but only 16.9% ($n = 60$) for cases having had biopsy only. Univariate significance was not achieved for the use of radiation therapy treatment ($p < 0.9$), or gender ($p < 0.4$). Race showed borderline significance ($p < 0.02$) with African Americans showing lower 5 year overall survival (45.2%; $n = 97$) compared with Caucasian (60.0%; $n = 795$). Multivariate analysis showed SEER stage ($p < 0.00001$), grade ($p < 0.00001$), size ($p < 0.0001$), and type of surgery ($p < 0.0039$) retained statistical significance. Overall urvival for limb sparing surgery was not different from amputation. The addition of radiation therapy to limb sparing surgery did not impact on overall survival.

### O25

**Postoperative hyperfractionated HDR-brachytherapy with iridium-192 combined with external beam radiotherapy for soft tissue sarcomas of the extremity or trunk**

T. Hehr[1], J. Classen[1], U. Lamprecht[1], M. Bamberg[1], W. Budach[2]
*([1]Department of Radiation Oncology, Tübingen University, Tübingen, Germany, [2]Department of Radiation Oncology, Düsseldorf University, Düsseldorf, Germany)*

*Aim:* A variety of radiotherapeutic approaches have been used in the adjuvant local management of soft tissue sarcoma. These include external beam irradiation, brachytherapy, and intraoperative radiation therapy. In appropriately selected patients, brachytherapy can be used as monotherapy or as a boost to external beam irradiation to treat possible microscopic residual disease at the tumor bed. We analysed practicability, toxicity, local tumor control and survival after hyperfractionated HDR-brachytherapy with iridium-192 as adjuvant boost combined with pre- or post-operative external beam irradiation for marginal resectable intermediate or high grade primary or recurrent soft tissue sarcomas of the extremity and trunk.
*Patients/methods:* Median diameter of 13 primary and 10 recurrent soft tissue sarcomas was 10 cm (range 3–20 cm), 17x extremity, grading GIII in 45%, 4x R0-resection (1–5 mm in sano), 13x R1-resection, and 5x R2-resection. Twenty patients received 23 courses of interstitial > HDR-brachytherapy with iridium-192 1.5 Gy BID, 15–30 Gy total dose, depending on resection margins. The median brachytherapy volume of the 1.5 Gy Isodose was 92 ccm. External beam irradiation was delivered to a median dose of 50 Gy (range 45–69 Gy, re-irradiation in 5 patients). Median follow up 27 months.
*Results:* Brachytherapy acute toxicity: 2*x* catheter dislocation, 1*x* subfebrile temperature, 1*x* dermatitis CTC°II. External beam irradiation acute toxicity: 1x dermatitis CTC°II, 3*x* lymphedema CTC°II. Actuarial 3-year survival 83%, disease free survival 51%; 3-year overall and extremity local tumor control was 78% and 87%, respectively.

### O26

**Improvement of local control in retroperitoneal disease: Surgical view**

P. Hohenberger
*(Division of Surgical Oncology and Thoracic Surgery, Klinikum Mannheim, University of Heidelberg, Germany)*

Retroperitoneal sarcoma, if treated with a primary surgical approach offer exactly one chance for cure by R0 resection. Liposarcoma of low grade as the type of tumor most often diagnosed usually can be resected completely with the retroperitoneal compartments providing surrounding tissue and organs serving as margins of safety. Unfortunately these characteristics are often neglected during shelling-out procedures.

As in extremity sarcoma, high grade tumors and those with extracompartmental extension should be treated with a combined modality approach. After removal of the sarcoma mass additional radiotherapy (RT) can applied only with an increased risk of radiation enteritis. This hold true also for completion-RT after IORT. According to presently available data, there is no place for adjuvant chemotherapy. Consequently, pretreatment biopsies (incisional or CT-guided) need to representatives specimen from those areas with different radiological appearance in Gd-enhanced MRT. If a G3 lesion is detected, neoadjuvant chemotherapy (alone or combined with hyperthermia in the EORTC 62961 study, or combined radiochemotherapy) or upfront radiotherapy (acc. ACOSOG trial) should be the first step.

Any operative strategy aiming for cure should be as radical as possible with resecting the total compartment if required (including colectomy, nephrectomy, partial pancreatic resection or even part of the aortic wall). The kidney itself uninvolved by tumor and resected for invasion of the ureter only can be reimplanted.

### O27

**Survival of rhabdomyosarcoma patients in Japan: 1991–2002**

H. Hosoi[1], S. Teramukai[2], Y. Matsumoto[1], K. Tsuchiya[1], T. Iehara[1], T. Sugimoto[1], T. Sawada[1]
*([1]Department of Pediatrics, Kyoto Prefectural University of Medicine, Japan, [2]Division of Clinical Trial Design and Management, Translational Research Center, Kyoto University Hospital, Japan)*

*Purpose:* To disclose characteristics of rhabdomyosarcoma in Japan and issues to discuss for future possible international cooperative studies.
*Patients:* Three hundred and twenty rhabdomyosarcoma patients treated from 1991 to 2002 in Japan.
*Methods:* Retrospective analysis for the outcomes of the patients based on IRS-V risk-grouping.
*Results:* The 5-year survival were 86.2, 80.7, 61.7 and 38.1 percent for the low-risk subgroup A, the low-risk subgroup B, the intermediate-risk group, and the high-risk group patients, respectively. In the high-risk group, the patient group that received high-dose chemotherapy (HDC) have better survival (58.2%) than the other patient group treated without HDC (18.4%) in significance ($p = 0.019$). There was no significant difference between the survival of embryonal (62.1%) and alveolar (58.5%) pathological subtypes.
*Discussion:* The worse outcomes for Japanese patients in the low-risk subgroup A and the low-risk subgroup B might be caused by some difficulty in the differential diagnosis between alveolar and embryonal subtypes. The better outcome for Japanese patients in the high-risk group might be due to the implementation of our original HDC regimen followed by auto-peripheral blood stem cell transplantation.

### O28

**Initial response and risk of early progression to regional HT (RHT) combined chemotherapy among patients with high-risk soft tissue sarcomas (HR-STS): A summary report**

L.H. Lindner[1,2], J. Braess[1], M. Schlemmer[1], S. Abdel-Rahman[1], R. Issels[1,2]
*([1]Klinikum Grosshadern, University of Munich, Germany, [2]GSF – National Research Center for Environment and Health, Neuherberg, Germany)*

*Introduction:* We analyzed the influence of grade (G) on initial response and the risk of early PD during RHT-combined chemotherapy.
*Patients and methods:* Doxorubicine based chemotherapy in combination with RHT was performed in a total of 319 STS patients. For risk assessment of early progression 213 patients were analyzed.
*Results:* For all patients with measurable disease ($n = 237/319$) the response rate (RR) was 33%. For G3, G2 and G1 STS the RR were 32%, 33% and 37%, respectively. RR was predictive for overall survival (OS) in patients with G3 and G2 STS (5 year OS was 59% vs. 26% and 60% vs. 24% for responders/non-responders, respectively). But in G1 STS 5 year OS was 85% for both. Early PD occurred in 33/213 pts (15%) within the first 4 cycles of thermo-chemotherapy. For the other 180 pts the 3-year local progression free rate (LPFR), local progression free survival (LPFS), and overall survival (OS) for E was 86.1%, 69.1% and 79.7% and for Non-E 48.0%, 38.1% and 56.7%, respectively.
*Conclusion:* Response is independent from grading but is predictive for OS in G2/G3 STS. The estimated probability for early PD is 15%. These pts do obviously not benefit from the preoperative treatment regimen.

### O29

**Chemotherapy for primary osteosarcoma by intra-arterial infusion**

N. Jaffe
*(Department of Pediatrics, University of Texas, M.D. Anderson Cancer Center, Houston, TX, USA)*

Therapeutic activity of an agent is related to mechanism of action and concentration at the target organ. Higher cytotoxic concentrations are achieved by the intra-arterial as opposed to the intravenous route. Intra-arterial chemotherapy for osteosarcoma has been administered with Mitomycin C, 5-Fluorourcil, Methotrexate, Adriamycin and Cisplatin. Cisplatin, 150 mg/m$^2$ administered over 2 hour at 2 to 3 week intervals (4–6 courses) achieved optimum results yielding tumor necrosis of 60–100%. Systemic Cisplatin concentrations attained by the intra-arterial route are similar to those attained by the intravenous route. Differences in the local as opposed to the peripheral vein concentrations, in the first 2 hours, are highly significant: highest single concentrations in the local draining vein at 60 to 90 minutes are 5–10 μg/ml with a steady state approaching 4 μg/ml in the systemic circulation ($p < 0.025$). Tissue concentration in the tumor and adjacent bone was 17 to 40 μg/gm with tumor destruction of 60–100%. Concentrations of 12 μg/gm or less yielded tumor destruction under 60% ($p < 0.025$). Major prognostic determinates of outcome were percentage of tumor necrosis and tumor size (burden). Response in the primary tumor to intra-arterial Cisplatin enhanced the safety of the surgical procedure for limb salvage.

### O30

**Is more better? Lessons from consecutive Ewing tumour studies**

H. Jürgens[1], C. Liebscher[2], M. Paulussen[1], A. Schuck[3], W. Winkelmann[4]
*([1]University Children's Hospital, Department of Paediatric Haematology and Oncology, Münster, Germany, [2]Coordinating Centre for Clinical Trials (KKS), University of Münster, Germany, [3]University Hospital, Department of Radiotherapy and Radiooncology, Münster, Germany, [4]University Hospital, Department of Orthopaedics, Münster, Germany)*

Chemotherapy and local control with surgery and/or radiation are essential in the treatment of Ewing tumours.

Results in patients below 25 years of age with biopsy-proven osseous Ewing tumour and no visible metastases were analysed with respect to the impact of trial (1981, 1986, 1992) and treatment strategy. A total of 400 patients (pts) were analysed (CESS 81, 77 pts; CESS 86, 130 pts; EICESS 92, 193 pts). The 5-year overall survival (OAS) for the whole group is 0.70 with a plateau of 64% at 13.8 years. 5-year OAS has increased from 0.60 (CESS 81) to 0.64 (CESS 86) and 0.79 (EICESS 92). This benefit was seen in both small (<100 ml) and large (>200 ml) tumours (small: 0.79, 0.68, 0.94; large: 0.31, 0.58, 0.69). A particular benefit was seen in good responders to chemotherapy (CESS 81, 0.79; EICESS 92, 0.95), in patients <15 years where OAS rose from 0.61 to 0.85, and in older patients (CESS 81, 0.33; EICESS 92, 0.62).

The benefit in prognosis is mainly related to increased chemotherapy intensity, i.e. substitution of cyclophosphamide by ifosfamide, and the extended use of surgery for local control (CESS 81, 64%; EICESS 92, 82%).

### O31

**Early risk adapted radiotherapy improves prognosis in children with primary nonresectable rhabdomyosarcoma: Report of the German cooperative soft tissue sarcoma studies**

E. Koscielniak[1], A. Schuck[2], B. Schmidt[3], T. Klingebiel[4], C. Int-Veen[1], J. Treuner[1]
*([1]Olgahospital, Oncol./Haematol., Stuttgart, Germany, [2]University of Muenster, Radiotherapy, Muenster, Germany, Katharinenhospital, Radiotherapy, Stuttgart, [3]University of Frankfurt/Main, Oncol./Haematol., Frankfurt/Main, Germany)*

The major questions concerning radiotherapy in pts with primary nonresectable rhabdomyosarcoma are: 1. Optimal time and dose 2. Succession of RT and surgery 3. When may RT be omitted? In the CWS-81 Study RT was stratified according to the results of SL surgery at week 16–20, given only to pts who still had microscopic (40 Gy) or macroscopic (50 Gy) residuals. In the CWS-86 Study the RT stratification has been changed – the RT was given in the majority of cases prior to SL surgery after one cycle of CT (7–10 weeks) and stratified by the degree of tumor volume reduction. The cumulative doses were: 32 Gy (favourable stratification criteria) or for patients with unfavorable features 54.4 Gy CWS-86, 48 Gy CWS-91, 45 Gy CWS-96. The single dose was reduced (1.6 Gy) and given twice a day (accelerated hyperfractionation) in the CWS-86, -91 and -96. This analysis included 429 pts with primary non resected localised RMS (Stage III). The proportion of irradiated pts was similar in all studies: 77% -79% -85%, -67%. The overall local tumor control (LTC) in irradiated pts was 56%, 81%, 81% and 70% in the CWS-81, -86, -91 and -96 respectively ($p = 0.01$). The median RT dose was 40 Gy vs. 32 Gy vs. 48 Gy vs. 43.6 in the CWS-81, -86 and -91 Study respectively. The prognosis improved dramatically in the CWS-86, -91 and -96 Study in the group of pts who were qualified as "good responders" after one cycle of the neoadjuvant CT and had been irradiated mostly prior to SL surgery (EFS-rate 69% vs. 46%, $p < 0.01$. The comparative dose of 40 Gy conventionally fractionated was given to 25 children in the CWS 81 Study (LTC = 48%) Early, risk adapted irradiation given preoperatively and concomitantly to chemotherapy improves prognosis in pts with primary nonresectable RMS.

### O32

**Impact of EWS-FLI1 expression on the p53 pathway in Ewing tumors**

J. Ban, M. Kreppel, C. Siligan, G. Jug, H. Kovar
*(Children's Cancer Research Institute, Vienna, Austria)*

P53 is a central regulator of cell cycle and death which is impaired in the majority of human cancers. In Ewing tumors (ESFT), however, p53 pathway alterations are rare. ESFT are characterized by rearrangements of *EWS* with the ETS transcription factor gene *FLI1* in 85% of cases. When introduced into primary cells of different origins, EWS-FLI1 induces p53 dependent cell cycle arrest or apoptosis while ESFT proliferation is dependent on sustained EWS-FLI1 expression independent of the presence of apparently fully functional p53. We tested the hypothesis that EWS-FLI1 may functionally interfere with p53 activity in the ESFT context. In reporter gene assays, EWS-FLI1 partially suppressed the p53 promoter, and suppression of endogenous EWS-FLI1 in ESFT cells by RNA interference slightly activated p53 promoter driven reporter gene activity. On the protein level, endogenous p53 was significantly induced upon EWS-FLI1 silencing in wildtype p53 ESFT cell lines. A similar p53 inducing effect was observed upon cell cycle inhibition or ectopic HEY1 expression in ESFT. In fact, *HEY1*, an established p53 regulator, was identified via chromatin immunoprecipitation as a gene directly suppressed by EWS-FLI1. Like EWS-FLI1 silencing, overexpression of HEY1 resulted in ESFT growth arrest. Our results imply that EWS-FLI1 suppresses basal p53 activity in ESFT.

### O33

**Comparison of treatment concepts for extraosseous Ewing Tumours (EET) within consecutive trials of two GPOH cooperative study groups**

R. Ladenstein[1], U. Pötschger[1], H. Jürgens[2], M. Paulussen[2], E. Koscielniak[3], J. Treuner[3]
*(The GPOH Cooperative Soft Tissue Sarcoma and Ewing Tumour Study Group, [1]St. Anna Kinderspital Wien, Austria, [2]Universitätskinderklinik Münster, Germany, [3]Olgahospital, Stuttgart, Germany)*

*Purpose:* Development of an effective strategy for patients (pts) with EPMD.
*Patients and Methods:* Of 192 (pts) registered, 141 pts have completed treatment. Median age is 15.8 yrs (0.4–49.29). Primary site was extremity in 57 pts and axial/other in 135 pts (40.6% in the pelvis). Tumour volume (TV) was ≥200 ml in 114 pts. Metastatic spread was bone marrow (BM) only in 17 pts, bone only in 94 pts and bone&BM in 79 pts. Additional metastatic sites were lungs (81 pts), liver (7 pts), CNS (7 pts) and lymph nodes (11 pts). 123 pts completed 6 VIDE induction cycles; reasons for <6VIDE were toxicity(7), patient choice(2), progression(6). Local treatments (surgery and/or radiotherapy) was documented in 86 pts. Recommended HDT was busulphan 600 mg/m$^2$ and melphalan 140 mg/m$^2$ with PSCR. Median follow up is 2 years (range 0.0–4.6).
*Results:* Partial remission or better was achieved in 42% by cycle 6. The 2 year overall survival (OS) for all 192 pts is 32.8%. Only 84/141 pts received BuMel-HDT and 10 pts other HDT. BuMel pts achieved an OS of 30%. Favourable OS factors at diagnosis are age <14 yrs ($p = 0.0069$), BM involvement only ($p = 0.0057$), single bone lesions only ($p = 0.0043$), TV <200 ml ($p = 0.0004$).
*Conclusions:* Further strategy refinement and validation of HDT appears necessary within randomised trials.

### O34

**Expression profiles of osteosarcoma that can predict metastatic potential**

C.C. Lau[1], C. Man[1], J. Gu[1], J. Visvanathan[1], J. Shen[1], L. Perlaky[1], J. Hicks[1], M. Johnson[1], N. Davino[1], J. Murray[2], L. Helman[3], W. Meyer[4], T. Triche[5], R. Gorlick[6], K.-K. Wong[1], M. Chintagumpala[1]
*([1]Baylor College of Medicine, Houston, TX, USA, [2]Cook Children's Hospital, Fort Worth, TX, USA, [3]National Cancer Institute, Bethesda, MD, USA, [4]University of Oklahoma Health Sciences Center, Oklahoma City, OK, USA, [5]Children's Hospital Los Angeles, Los Angeles, CA, USA, [6]Memorial Sloan Kettering Cancer Center, New York, NY, USA)*

Using cDNA microarrays, we have analyzed the expression profiles of 35 osteosarcoma cases consisting of 23 primary tumors and 12 metastatic lesions accrued from multiple institutions. The expression profiles of these samples were used to identify a set of significant predictor genes ($n = 45$), a metastatic signature, for predicting the metastatic potential of initial biopsies. This metastatic signature correctly classified 34 out of a total of 38 initial biopsy samples (correct classification rate = 89.5) and 22 of 26 initial biopsy samples in an independent validation set (84.6%). The model correctly predicted all 10 metastatic cases and 11 cases that initially presented with localized disease at the time of diagnosis, but developed metastatic disease later (sensitivity = 100%). Of the 17 cases that had only localized disease, four of them were predicted as metastatic (specificity = 76.5%) but two of these misclassified cases have relatively short follow-up (<2 years). In summary, we have used expression profiles to build a model that can predict metastatic potential of osteosarcomas at diagnosis. This model, together with our previous chemo-resistant prediction model, will help to refine the classification of osteosarcoma and ultimately impact the stratification of these patients in future clinical trials.

### O35

**Antitumor effect of EWS/Fli-1 junction point targeted antisense oligonucleotides by systemic injection in mice**

A. Maksimenko[1], P. Couvreur[4], V. Polard[1], J.R. Bertrand[2], M. Aboubakar[1], M. Gottikh[3], C. Malvy[2]
*([1]Bioalliance Pharma SA, Paris, France [2]CNRS UMR 8121, Institut G. Roussy, Villejuif, France, [3]Belozersky Institute of Physico-Chemical Biology, Lomonosov State University, Moscow, Russia, [4]CNRS UMR 8612, Faculty of Pharmacy Paris sud 11, Chatenay Malabry, France)*

The EWS/FlI-1 fusion gene, resulting from a t(11;22) translocation, plays a key role in the pathogenesis of Ewing's sarcoma and the mRNA fusion point constitutes a very specific target for therapeutic agents since it is only present in cancer cells. We have therefore designed an antisense oligonucleotide (ASO), made of 22 phosphodiester bonds and 8 phosphorothioate bonds, protected by its hairpin structure and complementary of type 1 fusion point in EWS/Fli-1 mRNA. It inhibits with specificity the expression of EWS/Fli-1 and cell growth when delivered by cationic lipids in cell culture. We have shown previously that this ASO inhibits with specificity the growth of a tumor expressing EWS/Fli-1 in nude mice when delivered intratumorally with bio-compatible polymeric nanospheres coated with a cationic derivative, CTAB. However CTAB could be toxic. We have now used chitozan for coating of the same nanospheres. Chitozan can be either reversibly or irreversibly bound to the nanospheres. In both cases these nanospheres are efficient by intratumoral injection as was CTAB. However when chitozan is irreversibly bound we obtain tumor growth inhibtion after intraveinous injection of the ASO-nanosphere complex.

### O36

**Conservative local treatment (surgery & brachytherapy) for boys with bladder-prostate rhabdomyosarcoma**

H. Martelli[1], C. Haie-Meder[2], O. Oberlin[2]
*([1]Hôpital Bicêtre, Le Kremlin-Bicêtre Cedex, France, [2]Institut Gustave Roussy, Villejuif, France)*

*Objectives:* to report the results of a conservative local treatment (surgery and brachytherapy) for boys with BP RMS, included in MMT 95 study.
*Patients:* From 1995 to 2003, 17 boys (14 were less than 6 years), were operated for a residual mass after chemotherapy and 1 for local relapse after chemotherapy alone. Patients underwent a partial prostatectomy or cystectomy or both, with urethra preservation. All but one patient had tumor cells in the resected specimen. Surgery was never microscopically complete at the level of the prostate. Brachytherapy was systematically performed after tumor resection. The technique consisted of 2 loops encompassing the prostate and the bladder neck area. A dose of 60 Gy was delivered with low dose rate.
*Results:* At a median follow-up of 4 years, 16 boys are alive in first complete remission. One boy died from pulmonary metastasis and another one from nodal and metastatic relapse. Results in terms of urinary continence seem very encouraging. Most of the boys are normally continent, sometimes after perineal physiotherapy and have erections.
*Conclusion:* Even if very long term sequelae of brachytherapy cannot be evaluated, this method should be considered in boys with bladder-prostate RMS.

### O37

**Detection and clinical significance of disseminated tumour cells at diagnosis in bone marrow of children with localised rhabdomyosarcoma**

H.P. McDowell[1], A. Donfrancesco[2], G.M. Milano[2], A. Clerico[3], O. Mannarino[2,3], P. Altavista[4], R. Boldrini[2], R. Cozza[2], A. Inserra[2] C. Dominici[2,3]
*([1]Division of Oncology, RLCH NHS Trust Alder Hey, Liverpool, UK, [2]Laboratory of Oncology & Division of Oncology, Bambino Gesù Children's Hospital, Rome; Italy, [3]Division of Oncology, Department of Paediatrics, La Sapienza University, Rome, Italy, [4]Section of Toxicology and Biomedical Sciences, ENEA Research Center Casaccia, Rome, Italy)*

Identification of poor prognosis patients with non-metastatic rhabdomyosarcoma (RMS) remains a clinical challenge. In an attempt to identify these patients a prospective analysis for the presence of disseminated RMS cells in bone marrow at diagnosis, using immunocytochemistry, with MyoD1 and myogenin as markers, was carried out. Thirty-seven patients treated on RMS88 and RMS96 Italian protocols underwent staging investigations, and in addition marrow examination for occult tumour cells. All patients had negative marrow involvement using cytomorphology, but 10/37 were positive with immunostaining. With a median follow-up of 30 months (range, 3–103), 7 patients are dead, 2 alive with disease and 28 disease-free. Marrow involvement was found to be associated with a significantly higher risk of distant recurrence ($p = 0.014$) rather than local and/or regional recurrence. Overall survival probability was 91% in patients with no marrow infiltration, 31% with marrow infiltration ($p = 0.001$); event-free survival probability was 88% in the former and 48% in the latter ($p = 0.01$). Disseminated tumour cells are indicative of disease spread and are significantly linked to recurrence at distant sites and poorer outcome. Marrow examination at diagnosis using immunocytochemistry, a relatively low cost investigation, may be an additional tool to modulate treatment.

### O38

**Children's Kaposi's sarcoma in an endemic area within the ongoing AIDS endemic**

B. Nkegoum[1], A. Gessain[2], P. Ndoumbé[3]
*([1]Pathology Service, University Teaching Hospital-Yaoundé-Cameroon, [2]Unit of Viral Oncology, Pasteur Institute-Paris, France, [3]Department of Pediatric Oncology-Faculty of Medicine-Yaoundé-Cameroon)*

*Background:* Cameroon (Central Africa) was known to be an endemic area of Kaposi's sarcoma even before the onset of AIDS epidemic. Jensen and al reported 16 cases of Kaposi's sarcoma in Cameroonian children between 1968 and 1974. The aim of this study was to show the anatomoclinical aspects of Kaposi's sarcoma within the ongoing AIDS epidemic in Cameroon.
*Materials and methods:* we carried out this retrospective study over a period of 18 years (from 1987 to 2004). The registers of the most important pathological laboratories in Cameroon were retrieved.
*Results:* Among the 556 cases noted, 4% were aged 0–15 years. The youngest patient was aged 3 months and was HIV positive. The male to female ratio was 3/1. The most common site of involvement was cervical lymph nodes. All the children presented with fever and weight loss. Skin and lymph nodes were simultaneously involved in 2 cases. Only 4 cases of HIV positive serology were observed in this series of children and the mother. The pathological aspect most frequently observed was advanced stage Kaposi's sarcoma. Less than 10 children received full dose of chemotherapy made up of cyclophosphamide, vincristine and prednisone. Only one child survived 9 years after chemotherapy.

### O39

**Ifosfamide vs. cyclophosphamide: Long term gonadal effects in male survivors of childhood cancer**

O. Fawaz[1], V. Ridola[1], C. Schmitt[2], J.C. Gentet[3], F. Aubier[1], D. Orbach[4], C. Bergeron[5], O. Oberlin[1]
*(SFCE (Société Française des Cancers de l'Enfant): [1]Institut Gustave Roussy, Villejuif, France, [2]Hôpital Brabois, Nancy; France, [3]Hôpital La Timone, Marseille; France, [4]Institut Curie, Paris; France, [5]Centre Léon Bérard, France)*

*Purpose:* To compare late effects of ifosfamide vs. cyclophosphamide on fertility in males survivors of childhood cancers.
*Patients:* 99 males were evaluated after treatment of a soft tissue sarcoma (37), osteoasarcoma (25), Ewing (6), lymphoma (28), other (3). Forty patients received ifosfamide as unique alkylating agent and the other 59 received cyclophosphamide as unique alkylating agent. Median interval after diagnosis was 9 years (4.5–19 yrs), median age at evaluation was 20 years (17.5–30.6 yrs), similar in the two groups. Median dose of ifosfamide was 54 g/m$^2$ (30–96), median dose of cyclophosphamide was 8.3 g/m$^2$ (4.6–22).
*Methods:* Evaluation was based on basal FSH measurement known for its correlation with spermatogenesis. LH and testosterone were also measured in most of the patients.
*Results:* All males but one (17.5 yrs) had normal testosterone levels. FSH was above laboratories upper limit in 28 of the 59 males treated with cyclophospahmide and was within the normal range in all the 40 pts treated with ifosfamide.
*Conclusions:* These results show a low risk of gonadal dysfunction in men exposed to ifosfamide (median dose 54 g/m$^2$) by contrast with the results for males treated with cyclophosphamide. Additional patients are under evaluation and will be presented.

### O40

**Cancer incidence in families of children with embryonal and alveolar rhabdomyosarcoma included in the Manchester children tumour registry**

D. Pang, R. Alston, A. Kelsey, J.M. Birch
*(University of Manchester & Cancer Research UK, Manchester, UK)*

*Purpose:* Histopathological subtypes of rhabdomyosarcoma (RMS) in children including embryonal RMS (eRMS) and alveolar RMS (aRMS) show distinct clinical features. However the comparative cancer patterns among families of patients have not been studied due to the rarity and difficulties of differential diagnoses. We now compare cancer incidences in relatives of children with these subtypes of RMS.
*Patients and methods:* All children under 15 years old included in the Manchester Children Tumour Registry between 1 January 1954 and 31 December 2001 with histologically confirmed RMS were eligible. All cases were re-reviewed including immunohistochemistry and molecular analysis. Families of cases were interviewed. Reported cancers in relatives were verified from medical records. Standardised incidence ratios (SIR) were estimated from the age, sex, and calendar year specific national cancer incidence rates. Results. For relatives of children with eRMS, there were excesses of soft tissue sarcoma (SIR = 5.6, $p = 0.006$), breast cancer (SIR = 1.8, $p = 0.03$), and cervix cancer (SIR = 3.4, $p = 0.009$). For aRMS families, there were excesses of soft tissue sarcoma (SIR = 4.2, $p = 0.04$), stomach cancer (SIR = 2.5, $p = 0.04$), and lung cancer (SIR = 1.8, $p = 0.02$).
*Conclusions:* For the first time we have demonstrated distinct differences in cancer patterns between eRMS families and aRMS. The role of germline mutations in genes other than TP53 should be explored.

### O41

**DNA methylation abnormalities in rhabdomyosarcomas**

D.M. Parham[1], B. Chen[2], C. Cooney[1], R.T. Kurmasheva[3], C.A. Peterson[1]
*([1]Departments of Pathology, Pediatrics, Biochemistry, Physiology, and Geriatrics, Arkansas Children's Hospital, University of Arkansas for Medical Sciences and Central Arkansas Veterans Health Care System, Little Rock, AR, USA, [2]Division of Laboratory Systems, Centers for Disease Control and Prevention, Atlanta, GA, USA, [3]Department of Molecular Pharmacology, St. Jude Children's Research Hospital, Memphis, TN, USA)*

DNA methylation has been implicated in a broad array of physiologic and pathologic processes. Because of its association with genomic imprinting and epigenetics, abnormal methylation holds great interest as a tumorigenic mechanism in the origin and maintenance of rhabdomyosarcoma (RMS). We have investigated DNA methylation in a series of experiments that demonstrate differences in methylation status among embryonal rhabdomyosarcomas (ERMS), alveolar rhabdomyosarcomas (ARMS), and normal tissues. Using methylation-sensitive Southern blotting, our results indicate that ERMS shows partial upstream MyoD gene methylation, similar to normal embryonic muscle and distinct from unmethylated MyoD in ARMS and totally methylated MyoD of postnatal tissues. Similarly, by methylation-sensitive PCR, the upstream region of the PAX3 gene appears to be hypermethylated in most ERMS and hypomethylated in most ARMS and normal muscle. In contrast, the cell cycle suppressor gene p16WAF shows hypermethylation in a subset of both ARMS and ERMS. Analysis of global methylation shows a trend towards global hypomethylation in a small number of RMS, whereas semi-quantitative RT-PCR reveals that DNA methyltransferase shows increased expression in RMS, particularly ERMS, suggesting a misregulation of enzyme activity. Our experiments demonstrate an array of methylation abnormalities in RMS that correlate with the known imprinting abnormalities of these neoplasms.

**O42**

**Systemic therapy for refractory soft-tissue sarcomas**

S.R. Patel
*(Department of Sarcoma Medical Oncology, MD Anderson Cancer Center, Houston, Texas, USA)*

A limited number of systemic therapeutic agents have some established activity in soft-tissue sarcomas. Several histologies are considered primarily refractory since no systemic therapy has been found to be effective for these subsets, e.g. alveolar soft parts sarcomas and clear cell sarcomas. For most of the other soft-tissue sarcomas, conventional/standard agents include doxorubicin, ifosfamide and dacarbazine. Patients with disease that has progressed despite these agents would be considered "refractory". With the rare exceptions of long-term disease control after surgical resections (mostly lung metastases), these patients are very likely incurable. The primary therapeutic intent in these patients therefore, is either palliation or occasionally, extension of life. Therapeutic options for these patients are also very limited and in many instances, histology-specific, e.g. high-dose ifosfamide for synovial sarcomas, taxanes for angiosarcomas, topoisomerase 1 inhibitors for rhabdomyosarcomas/Ewing's sarcomas and gemcitabine/gemcitabine-taxotere for leiomyosarcomas. Amongst agents still undergoing investigations, ecteinascidin seems to have some promising activity and is being pursued in liposarcomas and leiomyosarcomas. The activity of imatinib mesylate in GISTs and DFSP has fuelled the interest in several other "targeted" therapeutic agents, which are currently under investigation. It is hoped that ongoing clinical trials will help identify new agents with a better therapeutic ratio for these patients with refractory soft-tissue sarcomas. Continued efforts at development of new agents, and enrolment of patients on clinical trials is strongly recommended.

**O43**

**Should adolescents with Ewing tumors be treated in pediatric or non-pediatric oncology units? Data from the GPOH CESS/EICESS studies**

M. Paulussen, C. Liebscher, A. Hunold, D. Manner, H. Juergens
*(Department of Pediatric Hematology/Oncology, University Children's Hospital, Muenster, Germany)*

*Background:* Adolescents with cancer have lower survival rates than children. Ewing tumor patients treated according to the same protocol in all age groups in Germany were analyzed regarding age, treating institution, and outcome.
*Patients:* Data from 1426 patients treated on protocols CESS81, CESS86, and EICESS92 were analyzed. The treatment prescribed was indifferent of age. Patients were registered in pediatric (73%), or non-pediatric, usually (21%) medical oncology, institutions. Ten years event-free survival (EFS) was analyzed.
*Results:* Patients aged $\leq$15 years achieved an EFS of 0.50, EFS was 0.35 for patients >15 years, $p = 0.0001$. EFS was 0.49 for patients in pediatric, and 0.29 in non-pediatric units, $p = 0.0001$. Patients aged >15 years achieved an EFS of 0.41 in pediatric and of 0.29 in non-pediatric institutions, $p = 0.001$. Patients aged 15–20 years achieved EFS rates of 0.43 in pediatric and 0.24 in non-pediatric institutions, $p = 0.0002$. In stepwise multivariate analyses, older patient age lost significance, when the type of treating institution was introduced into the model. Treatment in non-pediatric institutions was an independent risk factor in this model (RR1.43, $p = 0.0006$).
*Conclusions:* Adolescents with Ewing tumors should best be treated in pediatric oncology institutions. Differences in patient care between types of institutions should be analyzed.

**O44**

**Improving survival in Ewing tumors: A surgeon's contribution**

A. Puri[1], M.G. Agarwal[1], C.N. Nair[2], P.A. Kurkure[2], S. Banavalli[2], M.A. Muckaden[3], S. Laskar[3], N.A. Jambhekar[4]
*([1]Department of Orthopaedic Oncology, [2]Department of Medical Oncology, [3]Department of Radiotherapy, [4]Department of Pathology, Tata Memorial Hospital, Mumbai, India)*

Our aim was to evaluate the role of adequate surgical excision as part of the multidisciplinary management of Ewing's tumors.

From January 2000 to August 2004 a total of 104 biopsy proven localised Ewing's/PNET tumors were surgically excised. Eighty five lesions arose in bone & 19 in soft tissues. Surgery followed induction chemotherapy. Wide excision with appropriate bone and soft tissue reconstruction as required was attempted. Limb salvage was accomplished in 92 cases and ablative surgery in 12 cases. Postoperatively the specimen was evaluated for surgical margins and response to chemotherapy. Patients showing residual viable tumor & those with positive resection margins received postoperative radiotherapy. All patients continued maintenance chemotherapy.

Eighty four patients with a minimum follow up of 12 months (12–58 months) were available for evaluation. Sixty six patients are alive without evidence of disease, 8 are alive with disease. Ten patients died due to progressive disease. Both cases with intralesional margins, 1 of 7 cases with involved margins & 5 of 75 cases with uninvolved margins had local recurrence. Of the 9 cases with inadequate margins 3 died and 2 have progressive disease.

Improving local control by adequate surgical excision could ultimately contribute to increased long term survival in Ewing's tumors.

**O45**

**Fusion gene dependent dissemination patterns of alveolar rhabdomyosarcoma in the nude rat model**

R. LeGallo[1], A. Burger[1], F.G. Barr[2], J.M. Gastier-Foster[1], R.B. Fraser[3], W.E. Shiels[1], S.J. Qualman[1]
*([1]Department of Laboratory Medicine, Columbus Children's Hospital, Columbus, OH, USA, [2]Department of Pathology and Laboratory Medicine, University of Pennsylvania, Philadelphia, PA, USA, [3]Department of Pathology, IWK Health Centre, Halifax, Nova Scotia, Canada)*

The direct influence of PAX-FKHR fusion genes on metastatic behavior of alveolar rhabdomyosarcoma (ARMS) in vivo has not been tested. We hypothesize that fusion gene presence is sufficient to alter the biologic properties of an embryonal RMS (ERMS) cell line into that of an ARMS-like pattern. The nude rat model was manipulated using a human ERMS cell line transfected with a construct containing the PAX3- or PAX7-FKHR fusion gene. Three levels of gene expression (low, medium, high) were studied. The ERMS cell line transfected with an empty vector served as control. All animals received intraarterial (intracardiac) injections of tumor cells (107). The controls showed a similar pattern of tumor dissemination as the native ERMS cell line, involving lungs and adrenal glands. The PAX3-FKHR transfected rats showed an increase of bone involvement at all levels of expression ($p < 0.05$). Distant and disparate sites including the brain, intraocular cavity, kidneys and lymph nodes were involved only in the high expression group ($p < 0.05$), suggesting gene dose dependency. The PAX7-FKHR transfected lines replicated the osteophilic deposition of the PAX3-FKHR transfections. The PAX-FKHR fusion genes determine the biologic dissemination patterns of ARMS (versus ERMS) in the nude rat reflective of RMS behavior in the human patient.

**O46**

**Results of second-look operations in the intergroup rhabdomyo-sarcoma study-IV, 1991–1997**

B. Raney, J. Stoner, J. Anderson, C. Arndt, K. Brown, W. Crist, H. Maurer, S. Qualman, M. Wharam, E. Wiener, W. Meyer
*(Children's Oncology Group: Arcadia, CA. Division of Pediatrics, UT MD Anderson Cancer Center, Houston, TX, USA)*

*Purpose:* To describe results of second-look operations [SLO] and relate findings to outcome.
*Patients/methods:* 79 patients [pts] with gross residual rhabdomyosarcoma had SLO after chemotherapy, with [$N=65$] or without [$N=14$] radiotherapy. Primary tumor sites were bladder/prostate [$N=27$], orbit/head/neck [$N=24$], extremity/trunk [$N=16$], and retroperitoneum/pelvis [$N=12$]. SLO was performed in 49 pts within 36 weeks after starting chemotherapy. Pts were grouped by preoperative tumor response before SLO and pathology results on SLO tissue.
*Results:* 15/18 pts [83%] without residual mass ["complete response"] had no viable tumor at SLO. 26/61 pts [43%] with residual mass had no viable tumor at SLO. The SLO patients were compared with 328 similar patients without SLO. 5-year failure-free survival [FFS] rates in those having or not having SLO were 68% and 74% respectively [$P=0.4$]. The 5-year FFS rate in those without viable tumor was 83% compared to 53% in those with viable tumor [$P < 0.001$].
*Conclusions:* Clinical/radiographic disappearance of tumor usually correlated with no viable tumor at SLO. Clinical/radiographic examinations detected residual masses, but were non-specific regarding tumor viability. Nearly half of the patients with residual masses had no viable tumor. Patients without viable tumor had a much better outlook for FFS than did those with persistent disease.

**O47**

**Current status of systemic treatment of GIST**

P. Reichardt
*(Department of Hematology and Oncology, Charité Campus Virchow-Klinikum, Berlin, Germany)*

Gastrointestinal stromal tumors (GIST) are the most frequent mesenchymal tumors originating from the GI tract. GISTs are strongly and uniformly positive for CD117 (c-kit), a type III receptor-tyrosine kinase. Kit mutations, leading to ligand independent constitutive activation play a major role in the pathogenesis of GIST. Until recently no active systemic treatment was available. Imatinib, a specific inhibitor of the c-kit tyrosine kinase has revolutionized the treatment of GIST with response rates around 60% and progression arrest in 80 to 90% of patients. Results from a large European trial show that imatinib 800 mg/day yields a 5 months advantage over 400 mg/day in terms of progression-free survival, although overall survival data are not yet mature. Once started, treatment with Imatinib should not be terminated, even in complete remission or after resection of residual disease. The role of adjuvant treatment after potentially curative resection of GIST is currently evaluated in ongoing clinical trials. Patients with progressive disease while under treatment with Imatinib should be enrolled in studies testing novel treatment strategies as RAD001, PKC412, AMN107, SU11248 or AMG706.

**O48**

**Molecular imaging in the genomic age: The next big thing**

D. Rosenbach
*(Tampa General Hospital, Affiliate Assistant Professor, Department of Radiology, University of South Florida, Florida, USA)*

Traditional radiologic evaluation of malignancy focuses by technological necessity on questions of anatomy and morphology. Recent advances in the understanding of genomics and proteomics have directed interest in imaging, instead, to oncology's underlying biological behavior. Molecular imaging (MI) represents an emerging discipline that aims to characterize and quantify the in vivo actions of cellular and molecular events involved in normal and pathologic processes, particularly malignancy. This new paradigm heralds not only a growing insight into human disease but also a shift from the anatomic and morphologic determinism of traditional radiology. MI probes, contrast agents in the broadest sense, interact with specific target genes and proteins and manifest as radioactivity, paramagnetism, fluorescence and acoustic impedance that can be detected by appropriate scanning modalities. By identifying the exact cellular events responsible for malignancy, molecular imaging transcends the demonstration of location and size and addresses the questions of tissue differentiation, tumor expression, metastatic disease, early response to treatment and development and validation of novel therapies.

**O49**

**Role of chimeric ETV6-NTRK3 transcript in diagnosis and prognosis of congenital infantile fibrosarcoma**

F. Sartori, R. Alaggio, P. Dall'Igna, B. Famengo, V. Ninfo, M. Carli, A. Rosolen
*(Clinica di Oncoematologia Pediatrica, Clinica di Chirurgia Pediatrica and Istituto di Anatomia Patologica, Azienda Ospedaliera-Università di Padova, Padova, Italy)*

Congenital Infantile Fibrosarcoma (CIFS) is a rare myofibroblastic lesion, morphologically indistinguishable from Adult Fibrosarcoma (AF) and Infantile fibromatosis (IF). Until recently age was the only criterion to distinguish CIFS from AF.

We investigated the diagnostic role of ETV6-NTRK3 transcript and its prognostic impact.

Hematoxylin-eosin stained slides and clinical reports from 10 patients with CIFS were reviewed. Total RNA was obtained from fresh or frozen samples and studied for the chimeric transcript ETV6-NTRK3 by RT-PCR.

Patient median age was 4 months. Median follow-up was 52 months. All cases were characterized by high cellularity, high mitotic rate and features of immature mesenchymal cells with focally infiltrative margins. Immunostains confirmed the myofibroblastic nature of cells (vimentin and smooth muscle actin positive, desmin and S100 negative). Four cases were positive for the ETV6-NTRK3 transcript and were morphologically undistinguishable from the 6 negative cases.

All ETV6-NTRK3 positive patients are alive and disease free. Transcript negative cases had an aggressive clinical course with multiple local relapses (6/6) and distant metastases (3/6). One patient died of disease after two years, the remaining are alive with disease.

Detection of ETV6-NTRK3 transcript is a useful diagnostic tool and may identify different prognostic groups within CIFS.

### O50

**Expression profiling of rhabdomyosarcoma: Increasing reliability of target gene selection by combining expression data generated from different platforms**

G.J. Schaaf[1], S. Gallego[2], P.D. Moerland[3], D. Zwijnenburg[1], A. Llort[2], R. Versteeg[1], M. Kool[1]
*([1]Department of Human Genetics, [3]Bioinformatics Laboratory, Academic Medical Centre, University of Amsterdam, Amsterdam, The Netherlands, [2]Unitat d'Oncologia Pediatrica, Hospital Universitari Vall d'Hebron, Barcelona, Spain)*

Rhabdomyosarcoma (RMS) is the most frequent soft tissue sarcoma in children. Heterogeneity in response to treatment still exists, in particular, the alveolar subtype portends a very poor prognosis. We have employed expression profiling analysis to select genes that are differentially regulated in RMS as compared to normal muscle, as well as genes that profoundly reflect the difference between alveolar (ARMS) and embryonal RMS (ERMS), the major histological subtypes. To increase the robustness of the candidate gene selection procedure, we only select genes that show consistent differential expression in the analysis of 3 different profiling platforms, including serial analysis of gene expression (SAGE), cDNA and oligonucleotide microarrays. For the comparison of RMS with normal muscle a large proportion of the genes downregulated in RMS were found to encode muscle-specific proteins (e.g. titin, troponin). Genes consistently upregulated in RMS are committed to cell cycle control. The comparison of ARMS to ERMS identified several genes that are involved in disease progression and metastatic disease. The relevance of identified genes will be further analyzed using tissue arrays that are generated in our departments. This will allow us to combine gene expression data with clinical parameters (e.g. age, histology, site, survival).

### O51

**Pharmacogenomic study on EWS-gene rearranged sarcoma**

K.-L. Schaefer[1], K. Brachwitz[1], Y. Braun[1], D. Wai[2], H.E. Gabbert[1], C. Poremba[1]
*([1]Institute of Pathology, Heinrich-Heine-University, Duesseldorf, Germany, [2]Children's Hospital Los Angeles, Los Angeles, CA, USA)*

The Ewing family of tumors (EFT) and clear cell sarcoma of soft tissue (CCSST) both belong to the malignancies of the musculoskeletal system which are characterized by a chromosomal translocation involving the EWS gene on chromosome 22. While for EFT neoadjuvant chemotherapy is well established as an indispensable component in the corresponding treatment protocols, for CCSST wide chirurgical resection is the mainstay of tumor therapy.

To elucidate the molecular similarities and varieties between these two tumor groups we sought to correlate the susceptibility of EFT and CCSST to standard cytostatic drugs with their respective gene expression pattern.

Seven CCSST and nine EFT cell lines were treated with a panel of common chemotherapeutic agents and the resulting LD50-values were correlated with their gene expression pattern as measured by using Affymetrix HG U133A microarrays.

In general, EFT are more sensitive to the most cytostatic drugs in comparison to CCSST. However in both tumor entities we also observed subsets of cell lines showing exceptional patterns of drug resistance or drug susceptibility respectively. These cell lines could be characterised by a specific expression pattern of marker genes. We therefore conclude that the functional analysis of these marker genes may be helpful to understand the individual differences in chemosensitivity in both EFT and CCSST.

### O52

**Single-center experience with more than 800 soft tissue sarcoma patients treated between 1993 and 2004: Outcome and prognostic factors**

P.M. Schlag, P.-U. Tunn, S. Koswig, P. Reichardt
*(Clinic for Surgery and Surgical Oncology, Charité Campus Buch, Berlin, Germany)*

*Purpose:* We report about our experiences of multimodal therapy of more than 800 patients in a single institution over a time-period of 12 years.
*Patients and methods:* 815 patients with soft tissue sarcoma underwent surgical treatment between 1992 and 2004. The average age was 51.5 years (10–82 years). 73.1% were located at the extremities, 12.2% at the trunk and 14.7 in the retroperitoneum. The most frequent sarcoma was liposarcoma (25.4%). High malignant sarcomas were predominant (G3 42%). 60% percent of sarcoma were greater than 5 cm, and 74% were located extracompartmental. A multimodal therapy was applied in 52.4% of the patients.
*Results:* R0 resection was achieved in 85%. Extremity conserving surgery was performed in 85.3%. 5-year local recurrence rate is 18.8%. Neoadjuvant therapy achieved in 41% a partial and in 11.1% a complete remission (isolated limb perfusion, radiation, chemotherapy). The 5 year overall survival rate is 66.9%. Patients with malignant peripheral nerve sheath tumor had a significantly worse overall survival (5-year SR: 49.9%). In contrast, patients with liposarcoma (82%) and synovial sarcoma (84.5%) had the best prognosis.
*Discussion:* Prognosis is dependent on R0 resection, grading, size of the tumor, metastasis and tumor localization. Additive therapy options within multimodal therapy concepts are promising.

### O53

**Radiotherapy in parameningeal rhabdomyosarcoma: Results of the CWS trials**

A. Schuck[1], C. Int-Veen[2], S. Kirsch[2], T. Dantonello[2], I. Brecht[2], B. Schmidt[3], E. Koscielniak[2], J. Treuner[2]
*([1]Radiotherapy Department, University Hospital Muenster, Muenster, Germany, [2]Department of Pediatric Oncology/Hematology, Olgahospital Stuttgart, Stuttgart, Germany,*

[3]*Radiotherapy Department, Katharinenhospital, Stuttgart, Germany)*

*Purpose:* The role of radiotherapy in patients with localized parameningeal RMS registered in the CWS trials was analyzed.
*Methods:* 304 study patients were treated between 4/1980 and 12/2002. Treatment consisted of systemic therapy and local therapy. The median follow up of surviving patients was 73 months.
*Results:* There was 1 patient in group I, 18 patients in group II and 285 patients in group III. EFS was 56% at 5 years overall. In group II, local relapse free survival (LRFS) at 5 years was 72%. One local relapse occurred in13 patients receiving radiotherapy and 3 in 3 patients without RT. In group III, LRFS at 5 years was 76%. 255 of 285 patients received radiotherapy. Median time from the start of treatment to onset of radiotherapy was 104 days in group III. No difference in local control was observed between patients with onset of RT < or >104 days. In IRS group III, 8 of 42 patients (19%) relapsed locally following 28–36 Gy and 32 of 204 patients (15%) after >36 Gy.
*Discussion:* Radiotherapy is essential in parameningeal RMS. No clear correlation between onset of RT and local control could be established.

### O54

**Prognostic significance of preoperative [18-F] fluorodeoxyglucose (FDG) positron emission tomography (PET) imaging in patients with resectable soft tissue sarcomas**

M.H.M. Schwarzbach[1], U. Hinz[2], A. Dimitrakopoulou-Strauss[3], F. Willeke[1] S. Cardona[1], G. Mechtersheimer[5], T. Lehnert[6], L. G. Strauss[3], C. Herfarth[1], M.W. Büchler[1]
*([1]Department of Surgery, [6]Division of Surgical Oncology, [2]Unit for Documentation and Statistics, [3]Department of Surgery, Clinical Cooperation Unit Nuclear Medicine, German Cancer Research Center, [5]Institute of Pathology, University of Heidelberg, Germany)*

*Purpose:* To evaluate the prognostic value of preoperative PET using FDG calculating the mean Standardized Uptake Values (SUV) in resectable soft tissue sarcomas (STS).
*Patients/methods:* 74 patients with STS underwent preoperative FDG-PET with SUV calculation. Data were analyzed for an association with the outcome. First and third quartiles of the SUV distribution function were used as cut-off values (1.59 and 3.6). Survival was estimated by the Kaplan-Meier method. Uni- and multivariate analyses were performed (log-rank test and Cox proportional hazards regression model).
*Results:* In 55 cases, STS were completely resected (follow-up 40 months): 5-year recurrence-free survival in patients with SUV <1.59, 1.59–<3.6, and ≥3.6 was 66%, 24%, and 11% ($P = 0.0034$). SUV was a predictor for survival (5-year rates: 84% [SUV <1.59], 45% [SUV 1.59–<3.6], and 38% [SUV ≥ 3.6]; $P = 0.057$) and local control (5-year rates: 93% [SUV <1.59], 43% [SUV 1.59–<3.6], and 15% [SUV ≥3.6]; $P = 0.0017$). By multivariate analysis, SUV predicted recurrence-free survival. The differences were associated with grade ($P = 0.002$).
*Conclusion:* The FDG uptake, as measured by the mean SUV in resectable STS, is a useful prognostic parameter. SUV with cut-off values at the first and the third quartiles predicted overall survival, recurrence-free survival, and local control. Therefore, it can be used to improve the preoperative assessment of resectable STS.

### O55

**Antiangiogenesis as an option for the treatment of bone and soft tissue sarcoma?**

L. Schweigerer
*(Department of Pediatrics I, Children's Hospital, University of Göttingen, Germany)*

Angiogenesis, the formation of new vessels, is indispensable for tissue growth and expansion. On the molecular level, relative tissue hypoxia or other situations generate angiogenic triggers, including the activation of humoral antiogenic stimulators and/or the suppression of antiangiogenic stummuli and/or the corresponding signal transduction systems in adjacent vascular endothelial cells. Signalling results in endothelial cell proliferation and the subsequent invasion of the cells into adjacent tissues, eventually generating functional new capillaries. Within the last decade, several angiogenic stimulators, inhibitors and their receptors have been identified including "vascular endothelial growth factor" (VEGF), the best-known angiogenic stimulator.

Angiogenesis is seen under various physiological conditions including pre- and postnatal growth. It is also seen during regeneration, for example during uterine growth and subsequent to cardiac infarction. Angiogenesis is also casually involved in the progression of solid pediatric malignancies. In contrast to malignancies prevalent in adults, those presenting in children are extremely well vascularized. Therefore, the inhibition of angiogenesis by endogenous or synthetic antiogenesis inhibitors could provide a means to improve conventional tumor therapy. We have identified several molecules able to inhibit experimental angiogenesis, including 2-methoxyestradiol, genistein and arginine deiminase. These could have the potential for clinical application, the most likely setting being additive to conventional treatment modalities.

Antiangiogenesis is still in its infancy. However, recent data have demonstrated its clinical potential in adult tumors, suggesting that antiangiogenesis could be feasible to improve outcome of children with solid malignancies.

### O56

**Visualization of xenotransplanted human rhabdomyosarcoma following transfection with red fluorescent protein**

G. Seitz[1], S.W. Warmann[1], R.M. Hoffman[2], H. Heitmann[1], J. Mahrt[3], J.T. Wessels[3], J. Fuchs[1]
*([1]Department of Pediatric Surgery, University Children's Hospital, Tübingen, [2]AntiCancer Inc., San Diego, California, USA, [3]Centre for Internal Medicine, Department of Nephrology and Rheumatology, Göttingen, Germany)*

*Purpose:* Discosoma sp. red fluorescent protein (DsRed2) is a novel marker for in vivo labelling studies in different biological systems. The aim of this study was to establish a DsRed2-transfected human rhabdomyosarcoma cell line and to perform xenotransplantation into nude mice for in vivo imaging.
*Materials and methods:* The rhabdomyosarcoma cell line (Rh30) was transfected with the pDsRed-N1 vector by lipofection. DsRed2-positive cells were sorted by FACS 96 h post transfection and selected in culture with G418. After vector transfection, a pure and stable DsRed2-positive clonal cell line was established. Expression of DsRed2-mRNA was assessed using single-cell rT-PCR. $1 \times 10^6$ RFP cells were injected subcutaneous into nude mice. Using a filter setup it was possible to detect tumour growth through the mouse skin even at a very early stage.
*Results/conclusion:* Successful vector transfection was achieved. After FACS followed by G418 selection and monoclonal selection, a pure DsRed2-positive cell line was established. rT-PCR revealed constant expression of DsRed2-mRNA. After xenotransplantation of these human cells, tumours could be detected and observed in non-invasively through the mouse skin. This is a useful tool for further investigations of the *in vivo* biology, angiogenesis and metastasis of human rhabdomyosarcoma.

### O57

**E-cadherin mediates cell-cell adhesion and suppression of anoikis during anchorage independent growth of Ewing tumor cells**

P.H.B. Sorensen[1,2], H.-G. Kang[1], J. Zhang[1], N. Keshelava[1], C.P. Reynolds[1], T.J. Triche[1]
*([1]Childrens Hospital Los Angeles, University of Southern California, Los Angeles, California, USA, [2]University of British Columbia, Vancouver, BC, Canada)*

Ability to grow under anchorage independent conditions is a hallmark of transformed cells. A key aspect of this process is the suppression of anoikis, defined as programmed cell death induced by detachment from the extracellular matrix. To model this phenomenon, we analyzed Ewing family tumor (EFT) cell lines growing as anchorage independent multicellular spheroids lacking attachment to underlying matrix. Not only do EFT spheroids show reduced basal levels of apoptosis compared with adherent cultures, but also demonstrate profound chemoresistance to a wide range of agents. We therefore hypothesized that cell-cell adhesions activate unique survival pathways in EFT spheroids, and analyzed which proteins might underlie this process. We found that while EFT monolayers utilize integrin-mediated adhesion to plastic, multicellular spheroid formation is dependent on cell-cell adhesions mediated by E-cadherin. Anti-E-cadherin antibodies blocked spheroid formation and led to massive cell apoptosis. Moreover, expression of dominant negative E-cadherin reduced spheroid formation, restored their chemosensitivity, and reduced cellular AKT activation. Spheroid cells demonstrated increased activation of the Rac1 and Cdc42 Rho family GTPases, and dominant negative Rac1 or Cdc42 blocked spheroid cell survival. These results suggest that anchorage independent Ewing tumor cells suppress anoikis through E-cadherin cell-cell adhesion and activation of Rho family GTPases.

### O58

**Results of osteosarcoma therapy without HD-MTX between 1986 and 1992: A twelve year follow-up of 53 patients**

J. Jakob[1], P. Reichardt[2], P.-U. Tunn[1]
*([1]Clinic for Surgery and Surgical Oncology, Robert-Rössle-Klinik, Charité-Campus Buch, Berlin, [2]Clinic for Hematology, Oncology and Tumor Immunology, Robert-Rössle-Klinik, Charité-Campus Buch, Berlin, Germany)*

*Background:* We report about results of systemic chemotherapy of osteosarcoma without HC-MTX.
*Patients and methods:* 53 patients with osteosarcoma of the extremity, stage 2B, were treated between 1986 and 1992 (age 6–36 years, median 17 years). All patients received a pre- and postoperative chemotherapy without HD-MTX. 60.4% of the patients had a tumor volume less than 150ml. 71.7% of the patients were treated by limb-sparing therapy, 20.8 by amputation and 7.5% by rotation plasty.
*Results:* The overall survival rate after 10 years was 67.2%. The rate of local relapse was 11.3%. Without local recurrence the 10 year survival was 74.2% ($p = 0.002$). Pulmonary metastasis deteriorates the progtnosis, the mean five year survival was 31.6%, a ten year survival was not achieved. If the tumor volume was less than 150 ml a ten year survival of 71.1% was observed, more than 150 ml led to 61.1% ($p = 0.445$) of 10 year overall survival. Overall response to preoperative chemotherapy was 45.3%. The 10 year survival rate was 73.5% and 62.1% for non responder.
*Conclusion:* The most important prognostic factors are local and systemic progression of the tumor. Comparison with the COSS protocol shows that therapy results without HC-MTX are identical.

### O59

**Successful high resolution animal positron emission tomography of human Ewing tumors and their metastases in a murine xenograft model**

C. Franzius[1], M. Hotfilder[2], C. Poremba[3], S. Hermann[1], K. Schäfers[1], H.E. Gabbert[3], H. Jürgens[2], O. Schober[1], M. Schäfers[1], J. Vormoor[2]
*([1]Department of Nuclear Medicine, University Hospital Muenster, Muenster, Germany, [2]Department of Pediatric Hematology and Oncology, University Hospital Muenster, Muenster Germany, [3]Institute of Pathology, Heinrich-Heine-University, Duesseldorf, Germany)*

*Purpose:* A major advantage of small animal PET is its capability to study molecular pathways in vivo non-invasively and serially. As primary osseous metastasis is the main adverse prognostic factor in patients with Ewing tumors, a NOD/scid mouse model for human Ewing tumor metastases has been established. The aim of this study was to evaluate the feasibility of diagnostic molecular imaging in this mouse model for human Ewing tumor metastases.
*Methods:* Human Ewing tumor cells (VH-64, CADO-ES1, TC-71) were transplanted into immune-deficient mice via s.c. injection ($n = 17$) or via i.v. injection ($n = 17$). To assess glucose utilization and bone metabolism, mice were scanned after injection of 2-[18F]Fluoro-2-deoxy-D-glucose (FDG) or [18F]Fluoride, respectively, using a submillimeter resolution animal PET scanner (quadHIDAC, Oxford Positron Systems Ltd, Oxford, UK).
*Results:* Subcutaneously transplantated Ewing tumors and metastases (lung, bone and soft-tissue) demonstrate a moderately increased glucose uptake. Osseous metastases are additionally visible on [18F]Fluoride PET by demonstrating a decreased [18F]Fluoride uptake (osteolysis). Hypermetabolic (FDG) and hypometabolic ([18F]Fluoride) metastases smaller than $10\,mm^3$ were depicted. Metastases were confirmed immunohistologically.
*Conclusion:* Diagnostic molecular imaging of Ewing tumors and their small metastases in an in vivo NOD/scid mouse model is feasible using a submillimeter resolution PET scanner.

### O60

**Subgroup classification of rhabdomyosarcoma by immunohistochemistry**

M. Wachtel[1], S. Stegmaier[2], I. Leuschner[3], B. Odermatt[4], T. Runge[1], E. Koscielniak[2], J. Treuner[2], F.K. Niggli[1], B.W. Schäfer[1]

*([1]Department of Oncology, University Children's Hospital Zurich, Switzerland, [2]Department of Pediatric Oncology/ Hematology, Olga Hospital Stuttgart, Germany, [3]Institute of Pathology, Universitätsklinikum Schleswig-Holstein, Kiel, Germany, [4]Department of Pathology, University Hospital Zurich, Switzerland)*

Rhabdomyosarcoma (RMS) is the most common soft tissue sarcoma in childhood. Based on histology, two different RMS subgroups are distinguished, the embryonal (ERMS) and the alveolar (ARMS) form. Due to a higher rate of metastasis formation, the outcome of the ARMS subgroup is much worse in comparison to the ERMS, and a correct classification therefore has clinical significance. However, classification solely based on histology is not sufficient in many cases and requires additional methods.

In the last decade, classification could be improved by the finding that 80% of the ARMS carry specific chromosomal translocations leading to the fusion of PAX3 or PAX7 to FKHR or NCOA1. However detection of the fusion-transcript needs high quality RNA, which is not always available, as the complete biopsy material is often needed for the initial histological analysis.

Performing a tissue array on 252 RMS tumors we demonstrate here that two translocation-dependent marker proteins, AP2beta and P-Cadherin, allow indirect detection of translocation-positive RMS by immunohistochemistry with high sensitivity and specificity. Another marker, the EGFR, shows high ERMS-specificity and supplements the classification.

Our data therefore simplify diagnostic classification of RMS on the level of immunohistochemistry.

**O61**

**Role of plastic and reconstructive surgery in sarcomas**

M. Greulich
*(Centre for Plastic Surgery, Marienhospital, Stuttgart, Germany)*

*Introduction:* Local tumour control is a major problem in sarcomas. Surgery therefore plays a key role in multimodal therapy. But mutilating and radical surgery should be avoided, especially in children and adolescents with respect to quality of life and late effects.
*Methods:* Examples for resection by means of plastic-reconstructive surgery and cosmetic and functional outcome are presented. A short summary of the applied techniques for coverage of major soft tissue defect is given as well (e.g. transplantation, microvasular coverage, flaps, vascular and neural reconstruction, microvascular bone replacement, muscle- and tendon transfers, etc.). Plastic surgery can be useful in reexision too. The difficulties in determining the optimal timing of radiotherapy and reconstructive surgery are considered. Radiotherapy can be avoided in some patients by means of plastic surgery.
*Conclusion:* Plastic and reconstructive surgery offers the possibility of resection without mutilation, but also allows complete tumor resections that would not have been possible with conventional surgery. Its use should be an essential part of multidisciplinary and multimodal therapy of sarcomas. Plastic-reconstructive surgery does not only improve outcome and quality of life, it can help to minimize the burden of therapy in sarcoma as well.

### Posters

### P001

**Inflammatory Myofibroblastic Tumors (IMTs) in children: Their relationship with HHV8 infection and prognostic role of histology.**

R. Alaggio, L. Calabrò, M. Barbierato, S. Bitetti, G. Bisogno, E.S.G. d'Amore, P. Dall'Igna, G. Cecchetto
*(Department of Oncology and Surgical Sciences, Pathology and Oncology Section, University of Padua, Italy)*

*Introduction:* IMTs can be neoplastic lesions, with clonal rearrangements of ALK1, or infection-related.
*Aim:* To identify prognostic histological parameters and investigate the role of human herpesvirus 8 (HHV8) in pediatric IMTs.
*Study design:* 25 children (mean age 8 years) affected by IMTs of lung (9), abdomen/pelvis (14), liver (1), spleen (1) were analysed. Cellularity, flogosis, myxoid component, necrosis, mitosis and cytologic atypia were evaluated, as well as smooth muscle actin (SMA), desmin, ALK1 and clusterin (Clu) expression.

Ten cases were analyzed for the presence of HHV8 sequences by nested-PCR using ORF25 and ORF26-specific primers, and 20 for LANA1/HHV8 immunohistochemical expression.
*Results:* All cases were Vimentin/SMA+, Desmin/CD21/CD23−, 7 ALK1+, and 12 Clu+. Among lung IMTs, 1 was ALK1+, and 6 Clu+. Among abdominal cases, 6 (1 liver) were ALK1+, and 6 Clu+. HHV8 sequences and LANA1 expression were detected in 1 pulmonary IMT. Abdominal involvement, mostly myxoid component, atypias, poor inflammation and ALK1 expression were found in 6/25 IMTs (2 AWD, 1 DOD).
*Conclusions:* Abdominal site, ALK1+, myxoid component, atypias might be unfavourable prognostic markers. Lung IMTs might be infection-associated, as suggested by the frequent Clu expression, involved in tissue remodelling. However, HHV8 detection is only occasional.

### P002

**Microsatellite instability is a rare event in Ewing's sarcoma**

I. Alldinger, K.L. Schaefer, D. Gödde, W.T. Knoefel, H.E. Gabbert, C. Poremba
*(Department of Pathology, University of Düsseldorf; Department of Surgery, University of Düsseldorf)*

We investigated paraffin-embedded tumor specimen from 63 patients with Ewing's sarcoma. For microsatellite analysis, we extracted DNA and performed PCR for 7 microsatellites. The PCR product was analysed in a Genetic Analyzer. 8 patients (14.5%) were MSI-L; one showed instability in D17S 250, 3 in BAT-25 and 4 in D5S 346. No instability was detected in BAT-26, D2S 123, BAX and TGFBR2. LOH was detected in 9 patients (16.4%), 6 in D17S 250, 3 in D2S 123 and 2 in D5S 346. One patient had LOH in D17S 250 and in D5S 346. The patient instable in BAT-25 had LOH in D2S 123. We analysed protein expression of the mismatch repair proteins MSH2, MSH6 and MLH1 by immunohistochemistry. In 54 of 59 patients (91.5%) we found a nuclear expression. In three patients expression of none of these proteins could be detected, with positive external control. Three reactions could not be assessed due to technical problems. One of the patients with no detected expression was the patient with high-grade MSI. We conclude that MSI is a rare event in Ewing's Sarcoma, and, unlike in HNPCC, MSI is not associated with lost expression of MSH2, MSH6 and MLH1.

### P003

**En-bloc chest wall and vertebral resection for sarcoma**

M. Incarbone[1], M. Alloisio[1], S. Luzzati[2] M. Infante[1], U. Cariboni[1], A. Testori[1], G. Ravasi[1]
*([1]Thoracic Surgery Department, Istituto Clinico Humanitas, Rozzano, Italy, [2]Orthopedics Department, Ospedale di Cremona, Italy)*

Improvement of surgical techniques has resulted in successful chest wall resection and simultaneous reconstruction. We reviewed our experience with chest wall sarcoma involving the spine, and verified 5-year survival and feasibility of this aggressive surgery.

Thirteen patients underwent surgical en-bloc resection for chest wall primary sarcoma involving the spine. Concurrent pulmonary resection was performed in twelve cases. A single hemi-vertebrectomy was performed in two patients, a triple hemi-vertebrectomy in two, a complete vertebrectomy in four, a triple complete vertebrectomy in five.

Surgery includes three main steps: chest wall and lung resection in posterolateral thoracotomy, spine section in median posterior approach, en-bloc resection and reconstruction.

Significative morbidity occurred in one patient who had lower limbs paralysis (9%). Mortality occurred in two patients (15.4%): One because of operative hemorrhage and one because of ARDS. The overall 5-year survival was 30.8%. Excluding the two perioperative deaths, the 5-year survival resulted 36.4%.

In spite of the limited number of patients, morbility and mortality outcome and 5-year survival lead us to think that surgery is the main therapy for primary sarcomas involving the spine: en-bloc chest wall, lung and vertebral resection is a safe and effective treatment.

### P004

**PKC412 induces cell death in alveolar rhabdomyosarcoma cells**

R.A. Amstutz, M. Wachtel. B.W. Schäfer
*(University Children's Hospital, Division of Oncology, Zurich, Switzerland)*

Rhabdomyosarcoma (RMS) accounts for about 5% of all childhood malignancies. Alveolar RMS (aRMS) is more metastatic than embryonal RMS (eRMS) and is therefore poorly prognosed. ARMS is characterised by two translocations t(2;13)(q35;q14) and t(1;13)(p36;q14) encoding for the transcription factors PAX3-FKHR and PAX7-FKHR. These chimeric proteins activate transcription at PAX binding sites more efficicently than its wild type forms both contributing to the malignant phenotype of aRMS cells including a decreased apoptosis and an increased proliferation rate. As shown earlier, the specific downregulation of PAX3-FKHR by antisense oligonucleotides reduces the cellular viability of aRMS cells. In this study, we show that N-benzoyl- staurosporine (PKC412), which was originallly identified as a Protein Kinase C inhibitor, also induces cell death in aRMS but not in eRMS cells. Furthermore, we show that PKC412 inhibits the transcriptional activity of PAX3-FKHR in 293T cells.

Treatment of PAX3-FKHR expressing 293T cells with PKC412 significantly reduced expression levels of several target genes of PAX3-FKHR compared to untreated cells. These results suggest targeting of PAX3-FKHR by PKC412 which in turn breaks down the anti-apoptotic pathways normally favoured by this transcription factor. Therefore, PKC412 is a promising agent for the treatment of alveolar rhabdomyosarcoma.

### P005

**Comparison of results of a pilot study of alternating vincristine/doxorubicin/cyclophosphamide and etoposide/ifosfamide with the fourth intergroup rhabdomyosarcoma study for intermediate risk rhabdomyosarcoma**

C. Arndt[1], J. Anderson[2], W. Meyer[3], D. Hawkins[4], S. Sencer[5], J. Neglia[6]
*([1]Department of Pediatric and Adolescent Medicine, Mayo Clinic, Rochester MN, USA, [2]Preventive and Societal Medicine, University of Nebraska, Omaha, NE, USA, [3]Department of Pediatrics, University of Oklahoma, Oklahoma City, OK; USA, [4]Department of Hematology/Oncology, Children's Hospital and Regional Medical Center, Seattle, WA, USA, [5]Children's Hospital and Clinics, Minneapolis-St. Paul, MN, USA, [6]Division of Pediatric Hematology Oncology, University of Minnesota, Minneapolis MN, USA)*

*Purpose:* To compare the outcome of patients with intermediate risk rhabdomyosarcoma (IRRMS) treated on a doxorubicin containing pilot study (pilot) utilizing alternating cycles of vincristine (V), doxorubicin (D), cyclophosphamide(C) and etoposide(E), ifosfamide(I) (Eur J Cancer 34:1224–29, 1998) with those of IRS-IV using three drug therapy (VActinomycin(A)C, VIE, VAI).
*Patients/methods:* 46 patients with IRRMS were treated on the pilot with doxorubicin. Their outcome was compared to a similar subset of patients treated on IRS-IV without doxorubicin. Because 22/46 pilot patients had parameningeal primaries (PM), these were analyzed separately.
*Results:* The 22 PM pilot patients and the 203 PM IRS-IV comparison subset had comparable patient and disease characteristics. Five year event free survival (EFS) and overall survival (OS) for PM subset were: Pilot: 82% and 82%; IRS-IV: 72% and 76% ($p = 0.42$). When the 24 pilot patients with other primary sites were compared to similar IRS-IV patients, there was no statistically significant difference in failure risk ($p = 0.23$). The estimated failure relative risk for the pilot compared to IRS-IV was 0.54 (95% CI: 0.20, 1.47).
*Conclusions:* This non-randomized comparison of a small ($N = 46$) pilot doxorubicin containing study of VDC/IE to a similar group of VAC/VAI/VIE patients treated on IRS-IV without doxorubicin showed no statistically significant difference in outcome.

### P006

**Anti-antiogenic scheduling of gemcitabine vinblastine carboplatin for relapsed and metastatic osteosarcoma: A new therapeutic option**

S. Ash[1], L. Kornrich[2], Y. Goshen[1], I.J. Cohen[1]
*([1]Department of Pediatric Hematology Oncology, Schneider Children's Medical Center of Israel, [2]Department of Imaging, Schneider Children's Medical Center of Israel, Petah-Tikva, Sackler School of Medicine, Tel Aviv University)*

*Objective:* To report development of a protocol for metastatic and relapsed osteosarcoma using an anti-angiogenic schedule with Gemcitabine (known to give palliation in osteosarcoma), vinblastine and carboplatin (known antiangiogenic drugs) Y.O. a 10 y old boy received the protocol after a local femoral relapse and lung metastasis with a pleural effusion. He was first treated with weekly Gemcitabine 1 gram/m$^2$ with a progressive improvement including pleural effusion disappearance, decrease in size of lung metastasis, negation of oxygen dependence and improved well-being. 4 months later the lung metastasis grew and 2 new lesions appeared. Additional vinblastine of 3 mg/m$^2$ resulted in mild improvement and after 2 months additional carboplatin 50 mg/m$^2$ (gemcitabine 500 mg/m$^2$) improved his condition for a short period of time. He died of disease after 10 months of weekly Gemeitabine.

Another adolescent remained asymptomatic on this protocol for 15 months after an almost complete resection of a lung/chest wall relapse until anasarca and acute renal failure developed. Spontaneous improvement occurred but 5 weeks later she developed 2 brain metastases and became moribund. Therapy was resumed and she became asymptomatic again and returned to full activity for 3 months until abdominal progression occurred. Another child received treatment for 3 months with only pain improvement.

### P007

**Prognostic factors in Ewing's sarcoma family of tumors**

S. Ash[1], T. Perri[1], S. Mor[2,★], G. Horev[3,7], I. Meller[4,7], D. Loven[5,7], J. Isakov[6,7], Y. Kollender[4], Y. Yaniv[1,7], R. Zaizov[1,7], I.J. Cohen[1,7]
*([1]Department of Hematology/Oncology, [3]Department of Radiology, Schneider Children's Medical Center of Israel, [2]Department of Pathology, [5]Department of Oncology, Rabin Medical Center, Petah Tiqva, Israel, [4]National Unit of Orthopedic Oncology, Israel, [6]Unit of Bone and Soft Tissue Pathology Sourasky Medical Center, Tel Aviv, Israel, [7]Sackler Faculty of Medicine, Tel Aviv University, Israel ★deceased)*

The study aim was to identify pre-treatment factors that predict survival and to assess prognosis with a more aggressive treatment protocol consisting of 4 courses of IV-VACA (ifosfamide, etoposide, vincristine, cyclofosphamide, actinomycin, adriamycin) radiation with 4000 cGY before and 2000 cGY after surgery, followed by 2 courses of IV-VAC (without adriamycin). 75 patients with Ewing Sarcoma admitted between 1972 and 1998 were reviewed.
*Result:* Metastatic disease, histologic subtype (PNET) and higher I.DH levels at diagnosis worsen prognosis. Response to treatment ($\geq$40% reduction in tumor size on imaging and $\leq$10% viable tumor at surgery after 2–3 chemotherapy courses) was a powerful predictor of survival ($p < 0.05$). Surprisingly, location of tumor at diagnosis (for localized disease only) had no prognostic significance (pelvic disease had the same prognosis as limb disease). 42 of the 60 patients with localized disease at diagnosis were treated with VACA or IV-VACA protocols 15 (35.7%) survived with no progression of the disease. Another 16 were treated with the new modified IV-VACA protocol with a mean follow-up of 73 months, this protocol yielded a better prognosis than the two previous protocols ($P = 0.03$ on multivariate analysis). 11 (68.7%) are alive with no disease progression.

**P008**

**Alternative Lengthening of Telomeres (ALT) as a predominant mechanism of telomere maintenance in embryonal rhabdomyosarcoma**

A. Ohali[1,2], S. Avigad[1,2], Y. Goshen[2], S. Ash[2], R. Zaizov[1,2], I. Yaniv[1,2].
*([1]Molecular Oncology, Felsenstein Medical Research Center, [2]Pediatric Hematology Oncology, Schneider Children's Medical Center of Israel, Petah Tikva, Sackler Faculty of Medicine, Tel Aviv University, Tel Aviv, Israel)*

*Purpose:* The two major types of rhabdomyosarcoma (RMS) are characterized by loss of heterozygosity (LOH) at the 11p15 locus (embryonal) and translocations between FKHR and PAX3 or PAX7 genes (alveolar). Telomeres, the repetitive sequences at the end of eukaryotic chromosomes, shorten with each cell division. In cancer cells, telomeres are maintained by activation of the telomerase enzyme or by the ALT mechanism (alternative lengthening of telomeres). Fifteen RMS tumors: 7 embryonal and 8 alveolar, were tested for telomere length (TRF) and telomerase activity (TA).
*Methods:* TA was evaluated by the expression of hTERT, using quantitative real time PCR. TRF was estimated using Southern blotting, and the TRF of each tumor sample was compared with matched peripheral blood (PBL) from the same patient.
*Results:* 75% alveolar RMS expressed hTERT, and heterogeneous telomere lengths: 4 tumors exhibited shorter telomeres and 4 tumors longer telomeres. In embryonal RMS, only 43% tumors expressed hTERT, and 80% presented long telomeres.
*Conclusions:* These results suggest that ALT might be the predominant mechanism of telomere maintenance in embryonal RMS, while in alveolar RMS, both telomerase dependent mechanism and ALT occur. The association between presence of ALT and better prognosis in embryonal RMS is currently being explored.

**P009**

**Oncosurgical and reconstructive therapeutic concepts in the treatment of soft tissue sarcomas of the extremities**

H. Bannasch, G. Felmerer, A. Momeni, M. Föhn, S.M. Ryu, G.B. Stark
*(Department of Plastic and Hand Surgery, University Medical School Freiburg, Germany)*

Between 1994 and 2004 59 patients with soft tissue sarcoma of the extremities underwent surgical treatment in our department. The therapy of each patient was sanctified by the interdisciplinary tumor conference of the university medical school. The localisation showed typical distribution patterns (upper extremity: $n=24$ (41%) and lower extremity: $n=35$ (59%). The most common technique used for oncosurgical resection was wide excision (70%), followed by compartment resection (22%); whereas 3 patients (5%) had to be amputated primarily (4 patients had to amputated secondarily due to local relapse). All patients underwent single step reconstruction of the defect according to the principles of the so called "plastic reconstructive ladder". Beginning with primary closure (20%) and skin grafts (24%), which were suitable after wide excision of epifascial sarcomas, local fasciocutaneous (8%) and myocutaneous (20%) flaps were used for safe coverage of exposed neurovascular structures. Free microvascular tissue transfer (fasciocutaneous, myocutaneous and osteocutaneous flaps) was performed in another 12 patients (20%) due to defects which could not be closed by local techniques. In 5 cases (8%) with significant functional loss due to compartment resection, a single step local muscle transfer was performed. The surgical therapy should include the armamentarium of complex reconstruction to be able to achieve radical resection and functional extremity preservation.

**P010**

**The bioexpandalbe prosthesis: A new perspective after resection of malignant bone tumors in children**

R. Baumgart, P. Thaller, S. Hinterwimmer, D. Bürklein, W. Mutschler
*(University of Munich, Dept of Surgery, Germany)*

Treatment of malignant bone tumors with prostheses in skeletally immature patients is difficult because of reduced stability and loss of growth plate in most cases. How can stability be improved and limb-length discrepancy be prevented?

Conventional expandable prosthetic devices cause an increasing disproportion between bone and prosthesis. To prevent such lack of correspondence of bone and prosthesis, a bioexpandable prosthesis has been developed which makes the bone to grow and not the prosthesis. The stem of the modular implant consists of an intramedullary nail with a smooth surface. When lengthening is necessary, an osteotomy of the remaining bone will be performed and the stem will be replaced by a fully implantable motorized distraction nail. This construction allows limb lengthening by callus distraction. External components are not necessary. The first prototypes of such a bioexpandable prosthesis have been clinically used following a 12 cm femoral resection in a 8 year old boy with osteosarcoma and removal of a 13 cm tibial segment in a 4 year old girl with Ewing sarcoma. The postoperative clinical courses were without complications.

Prosthetic joint replacement in combination with a fully implantable motorized distraction nail makes it possible to lengthen the remaining bone and increase stability.

**P011**

**Non-metastatic osteosarcoma in children: Treatment results and prognostic factors**

Z. Bekic[1], V. Svesko[1], B. Sbutega[2], B. Dimitrijevic[2], N. Lujic[2], M. Atanackovic[3]
*([1]Institute for Oncology and Radiology of Serbia, Belgrade, Serbia & Montenegro, [2]Institute of Orthopedic Surgery "Banjica", Belgrade, Serbia & Montenegro, [3]Institute of Pathology, Belgrade, Serbia & Montenegro)*

*Objective:* The aim of our study was to evaluate results of treatment and relevance of prognostic factors in patients with nonmetastatic osteosarcoma.
*Patients and methods:* From 1988–2000 we treated 92 patients with osteosarcoma, median age 15 years (range 3–18). 76 pts had large tumors volume over 150 ml.

Adjuvant chemotherapy after amputation was administered in 29 pts and neoadjuvant in 63 (intravenous/ intraarterial in 47 and 16 pts, respectively), followed by surgery (amputation in 32 pts, limb salvage in 27 pts, resection in 3 pts) and postoperative chemotherapy. Two-drug regimen (Adr-CDDP) was administered in 43 pts, while 49 pts received chemotherapy by other protocols.
*Results:* During the 38–186 months follow-up period (Me = 86) overall survival rate was 57% and disease-free survival rate was 55.8%. Prognostic factors significantly related to survival were tumor necrosis ($p=0.02$), tumor volume ($p=0.013$), duration of symptoms <6 months ($p=0.03$), and value of laboratory parameters: alkaline phosphatase ($p = 0.036$) and lactic dexogrenase ($p = 0.028$).

There were no significant differences in survival in relation to histopathological subtype, age, regimen or mode of chemotherapy applied (two-drug/multi-drug, i.v./i.a.).

*Conclusion:* Tumor load and responsiveness to chemotherapy are two major prognostic factors. The effects of Adr-CDDP regimen are similar to other more complex and toxic regimens.

### P012

**Phase II study of irinotecan in children with refractory or recurrent sarcomas**

G. Bisogno, R. Riccardi, A. Ruggiero, G. Surico, A. Prete, M. Provenzi, P. Infolfi, L. Casula, M. Carli
*(The AIEOP Soft Tissue Sarcoma Committee (STSC), Italy)*

*Background:* Irinotecan is a novel antineoplastic agent that works by inhibiting topoisomerase I. To evaluate its activity against pediatric soft tissue sarcomas (STS) the Italian STSC performed a multi-institutional phase II study.

*Methods:* Between 2002 and 2004, 32 heavily pre-treated patients were treated with a 60-minute infusion of 20 mg/m$^2$/day irinotecan for 5 days per week for two consecutive weeks. The courses were repeated every 4 weeks for at least 2 courses, unless there were some toxicity or disease progression. Overall 30 patients (12 rhabdomyosarcoma, 13 PNET, 3 Desmoplastic small round cell tumor and 2 other STS) were evaluable for response.

*Results:* In all, 79 cycles were delivered. Major regimen-related toxicity was diarrhea present in 58% of cycles with 11% graded as 3–4. Grade 3–4 neutropenia was reported in 10% of cycles. The response rate was 23% (2 CR + 5 PR) being 16% for rhabdomyosarcoma and 38% for PNET. In addition 4 minor responses were noted.

*Conclusions:* Irinotecan used as single agent in the treatment of recurrent/refractory STS showed an interesting response rate in a population of heavily pre-treated patients, especially in the subset of patients with PNET. Its toxicity profile warrants further investigations in association with myelotoxic agents.

### P013

**Parameningeal rhabdomyosarcoma: Improved survival in the protocols coordinated by the Italian Soft Tissue Sarcoma Committee (STSC)**

G. Bisogno, C. De Rossi, Y. Gamboa, G. Scarzello, E. Basso, S. Dallorso, C. Carollo, A. Scagnellato, A. Ferrari, A. Prete, M. Carli

*Purpose:* We report a series of patients with non-metastatic Parameningeal Rhabdomyosarcoma (PM-RMS), treated between 1979 and 2002, to analyse survival and prognostic factors.

*Methods:* 109 patients were enrolled in RMS-79, RMS-88 and RMS-96 studies. Biopsy or conservative surgery was recommended as initial approach. Chemotherapy was delivered according to the ongoing protocol: vincristine, actinomycin-D/adryamicin, cyclophoshamide (VAC/CAV) in the RMS-79 study (plus intrathecal therapy in case of intracranial extension). Ifosfamide replaced cyclophosphamide in the VAIA/IVA regimens used in the RMS-88 study. VAIA randomized vs. CEVAIE in the ongoing RMS-96 protocol. Radiotherapy (RT) was recommended at the beginning of treatment in RMS-79 (dose: 55 Gy), but was delayed to the 9th week in RMS-88 and RMS-96 (doses: 45–55 Gy), adopting the accelerated/hyperfractionated technique.

*Results:* For the whole population the 5-year overall survival (OS) was 64%. The OS for RMS-79, RMS-88 and RMS-96 was 40%, 72% and 70%, respectively. Protocol type was the most important prognostic factor. The different parameningeal sites, tumor size, invasiveness and histotype, and patient' age resulted no significant as well as known risk factors such as nerve palsy, skull base erosion and intracranial extension.

*Conclusions:* Despite RT being delayed and doses reduced, the outcome for PM-RMS patients has improved over years. With the improvement of the treatment the known prognostic factors have lost their impact.

### P014

**Malignant fibrous histiocytoma of bone after irradiation for a myxoid liposarcoma in a 16 year old patient**

B. Bode[1], U. Exner[2], S. Frigerio[1], J. Hodler[3], B. Lombriser[4], H.P. Honegger[5]
*([1]Institute of Clinical Pathology, University Hospital, Zurich, Switzerland, [2]Clinic of Orthopeadics, Balgrist Hospital, Zurich, Switzerland, [3]Radiology, Balgrist Hospital, Zurich, Switzerland, [4]Radioocology, Triemli Hospital, Zurich, Switzerland, [5]Oncology, Triemli Hospital, Zurich, Switzerland*

Radiation associated sarcomas are rare complication of adjuvant treatment. We present an unusual case of a short interval between development of an intraosseous malignant fibrous histiocytoma following irradiation for a mariginally resected myxoid liposarcoma with supportive molecular data.

16 year old male patient presented with a slowly growing mass on the medial aspect of the knee. Radiologically, a deep seated, partially intraarticularly growing tumor was detected. Biopsy specimen showed a myxoid liposarcoma, but the RT-PCR for the diagnostic translocation was negative. The mariginal resection followed with histologic confirmation of the myxoid liposarcoma. 70 Gy irradiation was applied to the operation field. In the follow up MRI an intraosseous signal alteration of the distal femur was detected 1.5 years later. At further follow up the change was progressive and a biopsy was performed. Pleomorphic malignant tumor without osteoid production, consistent with malignant fibrous histiocytoma was diagnosed histologically. The resection of the distal femur after neoadjuvant therapy was performed. The tumor was histologically confined to the bone. Repeated RT-PCR on the myxoid liposarcoma using own designed primers revealed the diagnostic translocation t(12;22) with a unique breaking point, which was absent in all examined specinems of morphologically different bone sarcoma.

Close follow up of the soft tissue sarcoma patient allowed diagnosis of a unexpected bone sarcoma in the irradiation field in an early stage.

### P015

**Evaluating the chemosensitivity of clear cell sarcoma of soft tissue by an in vitro-assay**

K. Brachwitz, K.L. Schaefer, Y. Braun, H.E. Gabbert, C. Poremba
*(Department of Pathology, Heinrich-Heine-University, Duesseldorf, Germany)*

Clear cell sarcoma of soft tissue (CCSST) represents a rare, slowly growing tumor usually affecting adolescents and young adults. CCSST demonstrates a fully malignant neoplasm with tendency to metastasize, local recurrences and 5-year survival rates of ~50%. Retrospective data often reveal few advantage of an adjuvant chemotherapy since this tumor entity often shows resistance against the majority of therapy schedules. In vitro cytotoxicity-assays are not published for CCSST. Therefore we evaluated the effectiveness of agents like adriamycine, ifosfamide, etoposide, dactinomycine, irinotecan's metabolite SN-38 and vincristine in CCSST cell lines compared to Ewing's tumor cell lines which are assumed to be chemotherapy-sensitive. The growth inhibition was measured by an MTT-assay. 6/7 cell lines were sensitive against adriamycine, SN-38 and dactinomycine, and partly sensitive against ifosfamide. Only 2/7 cell lines were sensitive against etoposide, but all were resistant against vincristine.

In an ongoing project we are about to generate expression profiles using DNA microarrays (Affymetrix U133A) to identify critical differentially-expressed genes in chemo-sensitive and chemo-resistant CCSST tumor cell predicting characteristics for response to treatment.

**P016**

**Combination telomerase inhibition therapy in Ewing sarcoma cell lines**

Y. Braun[1], A. Truck[2], I. Duran-Seuberth[2] C. Lanvers-Kaminsky[2], A. Schuck[3], H.E. Gabbert[1], C. Poremba[1]
*([1]Institute of Pathology, Heinrich-Heine-University, Duesseldorf; Germany, [2]University Children's Hospital, Department of Pediatric Hematology and Oncology, Muenster; Germany, [3]Department of Radiotherapy, University Children's Hospital Muenster, Germany)*

Telomeres (the tips of chromosomes) shorten gradually with each cell cycle due to the end–replication–problem leading to growth arrest and apoptosis. Telomerase is a ribonucleoprotein enzyme responsible for telomere maintenance. Inhibition of telomerase is a promising therapeutic target, since it is required for the long-term proliferation of most cancer cells but not present in most somatic cells.

Telomerase inhibitors by themselves will result in telomere shortening, but it may take many cell replications to achieve the benefit of such therapies. Combining conventional therapies with a telomerase inhibitor may accelerate the onset of proliferative deficiencies in cancer cells.

To explore this hypothesis we inhibited telomerase activity in two Ewing sarcoma cell lines, RM–82 and STAET–1, by stable expression of a dominant-negative mutant (DN) of the telomerase catalytic unit (hTERT, human telomerase reverse transcritpase) and subsequently treated these cells with several cytotoxic agents (vincristine, doxorubicin and etoposide, respectively).

After transfection (up to three passages) hTERT inactivation so far did not reveal an synergistic effect on growth inhibition by treatment with doxorubicin, vincristine, or etoposide in STA-ET-1 cells. Analysis of RM-82 and long-term inhibition experiments for both cell lines are ongoing.

**P017**

**Localized peripheral primitive neuroectodermal tumors (pPNET) and extraosseus Ewing's Sarcomas (EES) in children and adolescents: Results of the prospective studies CWS 81, 86, 91 and 96 of the German cooperative soft tissue sarcoma study group**

I.B. Brecht[1], C. Int-Veen[1], T. Dantonello[1], S. Kirsch[1], I. Leuschner[2], A. Schuck[3], H. Jürgens[4], S. Bielack[4], R. Ladenstein[5], G. Ljungman[6], B. Kasanowska[7], T. Klingebiel[8], E. Koscielniak[1], J. Treuner[1]
*([1]Department of Pediatric Oncology/Hematology, Olgahospital Stuttgart, Germany, [2]Department of Paidopathology, University of Kiel, Germany, [3]Department of Radiotherapy, University of Münster, Germany, [4]Department of Pediatric Oncology/Hematology, University of Münster, Germany, [5]Department of Pediatric Oncology/Hematology, St. Anna Kinderspital Vienna, Austria, [6]Department of Pediatric Hematology/Oncology, Uppsala, Sweden, [7]Department of Children Oncology/Hematology, Wroclaw, Poland, [8]Department of Pediatric Oncology/Hematology, University Frankfurt, Germany)*

*Objectives:* To show the results of the prospective CWS 81–96 studies for EES/pPNET, to evaluate prognostic factors and to find a prognostic favourable subgroup in order to adapt the therapy regime.
*Patients/methods:* 224 children and adolescents with localized pPNET ($n = 152$) and EES ($n = 72$) diagnosed between 1981 and 2000 and registered with the CWS 81 ($n = 13$), CWS 86 ($n = 43$), CWS 91 ($n = 77$) and CWS 96 ($n = 91$) studies were analysed for risk factors and outcome. The median age was 11 years, most tumours occurred in extremities (28%), thorax (19%), trunk (16%) and small pelvis (10%). Median follow-up was 54.9 months. All patients received chemotherapy, 162 received radiotherapy and 60 patients not. Primary complete resection was performed, if possible (22 IRS I, 49 IRS II, 151 IRS III).
*Results:* 5-year EFS was 58.0% (EES 65.8%, pPNET 53.8%) and SUR 67.9% (EES 79.9%, pPNET 61.7%). 12.9% patients showed local relapses, 12.5% metastatic relapses, 14% combined relapses and 4.9% showed initial tumour progression. Risk factors showing significant influence on the outcome are invasiveness ($p = 0.005$), size ($p < 0.001$), IRS group (SUR IRS I 85.8%, IRS II 79.6%, IRS III 61.6% $p = 0.01$). Prognosis in small and not invasive tumours is excellent (EFS 89.6%, SUR 96.3%), but still bad for the large group of T2b tumours (EFS 48.5%, SUR 59.6%). Response to chemotherapy ($p = 0.003$) and radiotherapy in IRS group II ($p < 0.001$) have significant influence on prognosis.
*Conclusions:* Prognosis in children and adolescents with EES/pPNET is still not satisfactory, but an especially favourable subgroup of patients with small and non-invasive could be found, which might need less therapy. Complete resection, response to chemotherapy and radiotherapy are important factors for prognosis.

**P018**

**Leiomyosarcoma in children and adolescents: A retrospective analysis of the German and Italian pediatric soft tissue sarcoma groups on prognostic factors and outcome**

I.B. Brecht[1], C. Int-Veen[1], A. Ferrari[2], G. Bisogno[3], S. Kirsch[1], T. Dantonello[1], A. Schuck[4], M. Casanova[2], T. Klingebiel[5], E. Koscielniak[1], M. Carli[3], J. Treuner[1]
*([1]CWS study group, Olgahospital Stuttgart, Germany, [2]Paediatric Oncology Unit, Istituto Nazionale Tumori Milano, Italy, [3]Devision of Pediatric Hematology and Oncology, Padova University, Italy, [4]Radiotherapy Department, University Hospital Munster, Germany, [5]Department of Paediatric Oncology and Hematology, University Frankfurt, Germany)*

*Objectives:* To evaluate prognostic factors and outcome of children and adolescents with leiomyosarcoma treated with different therapy regimes.

*Patients/methods:* 62 patients with leiomyosarcoma registered within the CWS and ICG studies between 1980 and 2003. Median follow up was 7.15 years. Male: female ratio was 1.4, median age 8.8 years. Primary sites were: other site (trunk, thorax, abdomen: 30) extremity (20), head and neck (10), parameningeal (2). 14% were lymph node positive, 39% showed invasive growth and tumour size was >5 cm in 39%. IRS group was IRS I in 34, IRS II in 10, IRS III in 13 and IRS IV in 5 patients. The patients had different adjuvant therapy (23 pts chemotherapy, 1 pt radiotherapy, 10 pts chemo- and radiotherapy).
*Results:* Overall SUR was 80.7% and EFS 72.2% for all patients. 5yr SUR (EFS) was 91.8% (84.5%) in IRS group I, 76.1% (68.5%) in IRS group II, 74.1% (66.6%) in IRS group III and 40% (20%) in IRS group IV. 13% of all patients showed local relapse, 8% metastatic relapse and 3% initial progression. Age (≤10 yrs, >10 yrs) showed significance in the $\chi^2$-test, no other risk factors could be found. Abdominal localisation seems to be favourable. The influence of radiotherapy could not been proven. Good response to chemotherapy was seen in 43%.
*Conclusions:* Prognosis in children and adolescents with leiomyosarcoma is very good, especially when completely resected. Prognostically favourable is age less than 10 years and abdominal localisation.

### P019

### Cardiac sarcoma in children and adolescents: Two cases registered with the German pediatric soft tissue sarcoma study

I.B. Brecht[1], I. Leuschner[2], V. Debus[3], H. Jürgens[4], T. Spieker[5], S. Kirsch[1], T. Dantonello[1], C. Int-Veen[1], A. Schuck[3], S. Bielack[4], T. Klingebiel[5], E. Koscielniak[1], J. Treuner[1]
*([1]CWS study group, Olgahospital Stuttgart, Germany, [2]Department of Paidopathology, University of Kiel, Germany, [3]Department of Pediatric Cardiology, University Children's Hospital Muenster, Germany, [4]Department of Ped. Oncology/Hematology, University Children's Hospital Muenster, Germany, [5]Gerhard-Domagk-Institut of Pathology, University Munster, Germany)*

*Objectives:* To show the outcome of two cases of the extremely rare cardiac sarcomas in children and adolescents registered with the CWS-study in 25 years.
*Patients:* A 16 year old girl with cardiac leiomyosarcoma (G2) in the right atrium, 10 cm, N0, M0 (patient 1) and an 8 year old boy with cardiac malignant mesenchymoma (G3) in the left ventricle, >5 cm, N0, M0 (patient 2) are shown.
*Results:* The leiomyosarcoma of patient 1 was completely resected and chemotherapy (vincristine, adriamycin, actinomycin and ifosfamide) was given. The girl is still alive in complete remission after 7 years. The cardiac sarcoma of patient 2 was first resected in 2003, the histology showed a liposarcoma (G1). After rapid progression the patient received a heart transplantation in 2004. The histology showed now a malignant mesenchymoma (G3) with infiltration of the whole left ventricle. The patient died in the same year of transplantation.
*Conclusions:* Cardiac sarcomas are very rare in children and adolescents. Though prognosis is known to be extremely bad we can show a long term survivor with cardiac leiomyosarcoma.

### P020

### Adherence to treatment protocols in Ewing's sarcoma and osteosarcoma in patients treated at the departments of pediatric oncology and medical oncology

P. Brons, J. Boekhorst, Q. van Hoesel, H. Schreuder, P. Hoogerbrugge
*(Departments of Pediatric Oncology (PB, JB, PH), Medical Oncology (QH) and Orthopaedics (HS), Radboud University Nijmegen Medical Centre, Nijmegen, The Netherlands)*

Children treated for sarcoma have a better outcome as compared to adults. Possible explanations for this include intrinsic differences of tumor biology, clinical characteristics or protocol adherence.

We compared the protocol adherence of patients with a Ewing sarcoma (ES $n = 27$) and osteosarcoma (OS $n = 42$) treated at the dept. of Pediatric Oncology (PO) and Medical Oncology (MO), diagnosed between 1992 and 2002. Patients treated at the dept. of PO had a signifcant shorter delay between time of diagnostic biopsy and start of treatment. All patients but 3 were included in international trials (EICESS 92; EORTC 80931 and Euro Ewing 99). We found no difference in delay of surgery or neoadjuvant chemotherapy and survival between patients treated at the dept. of PO and MO. Protocol non-adherence was noticed in 16/33 patients treated at the dept. of MO and 7/34 patients treated at the dept. of PO. Reasons for protocol non-adherence were: Unknown (20% and 0%), lack of response (80% and 46%), patient decision (0% and 23%) and toxicity (0% and 30%) for patients treated at the the dept. of PO and MO, respectively.
*Conclusion:* Protocol adherence was less at the dept. of MO due to toxicity and patient decision.

### P021

### Fatal diagnosis: Dedifferentiated chondrosarcoma – report on 13 patients

J. Bruns[1], W. Fiedler[2], M. Werner[3], G. Delling[3]
*([1]Department of Orthopaedic Surgery, University of Hamburg, Germany, [2]Department of Hematology & Oncology, University of Hamburg, Germany, [3]Institute for Osteopathology, University of Hamburg, Germany)*

Dedifferentiated Chondrosarcoma (DDCS) are one of the skeletal tumors with the worst prognosis. It was the aim of our study to get any hints regarding therapeutic improvements.
*Methods:* Retrospectively, we analysed the data of patients suffering from DDCS which were treated surgically in the last 15 years. For staging the MSTS-Enneking system was used; determination of the survival rate was done by the Kaplan-Meier analysis.
*Results:* Between 1990 and 2003 13 patients suffering from a DDCS were treated.

The mean age was 59.8 yrs, (6 female, 7 male). The location of the tumor was the pelvis 4X, proximal femur 3X, distal femur 4X, proximal humerus 2X. 9 patients were staged IIB and 4 patients staged III. In all the diagnosis was histologically confirmed either by biopsy or complete histological analysis of the tumor. In all but one the treatment was surgically. In 6 cases surgery was accompanied by chemotherapy using holoxan and adriablastin. In 11 cases surgery was performed, in one patient with a pelvic DDCS surgery was renounced, one patient died during the preop. chemotherapy; in ten cases curative resection was planned, in one patient a palliative intralesional resection was done; in 6/10 cases with a curative goal we achieved a wide resection, a marginal resection in 3/10 and an intralesional resection in 3 cases. The mean time of survival was 9.6 months (2–26 mo.) Local recurrence was observed in 1 case. All dead patients died due to metastasis to the lung. No obvious differences in the time of survival were detectable depending on the use of chemotherapy.

**P022**

**Saving limbs with soft tissue sarcomas requires integration of plastic-reconstructive surgery into the interdisciplinary treatment concept**

Th. R. Bund, L.U. Lahoda, C. Choi, P.M. Vogt
*(Klinik für Plastische, Hand- und Wiederherstellungschirurgie der Medizinischen Hochschule Hannover, Klinikum Hannover Oststadt, Podbielskistraße 380, Hannover, Germany)*

Soft tissue sarcoma of the extremities is a rare disease in adults with an incidence of 2–3 per 100 000 inhabitants. Wide tumor excision (R0) is the most important factor for local disease control. Limb sparing surgery combined with adjuvant forms of therapy represents a preferable alternative to amputation even in advanced sarcomas if limb function can be preserved. Plastic surgical and reconstructive procedures permit coverage of even major defects after wide R0 excision and allow restoration of function even if large bony defects and extensive loss of neuro-muscular units occur. We present interdisciplinary concepts for limb preserving therapy with soft tissue sarcomas formerly being amputated. Due to the rareness of these soft tissue sarcomas and the intricacy of therapy, patients should be referred to centres of "excellence" offering interdisciplinary, multimodular treatment options. Plastic and reconstructive surgery is an integral part of these options. As such plastic surgery needs to be involved from the start when patients with soft tissue sarcomas are to be treated emphasizing restoration of form and function.

**P023**

**Immunohistochemical characteristics of uterine smooth muscle tumors**

G.M. Burkadze, G.A. Turashvili
*(Department of Pathological Anatomy, Tbilisi State Medical University, Tbilisi, Georgia)*

*Purpose:* To explore more sensitive immunohistochemical markers in differential diagnosis of uterine leiomyosarcoma (LMS) and the specific subtypes of leiomyoma.
*Patients:* 14 cases of usual leiomyomas (UL), 16 cases of cellular myomas (CL), 14 cases of bizzare leiomyomas (BL), 15 cases of smooth muscle tumors of uncertain malignant potential (STUMP), and 13 cases of LMS were investigated.
*Methods:* Formalin-fixed paraffin-embedded surgical specimens were immunostained for Ki-67, p53, Bcl-2, smooth muscle actin (SMA), estrogen (ER) and progesterone receptors (PR), and cathepsin D (CD) using avidin-biotin-peroxidase method.
*Results/discussion:* The expression of Ki-67 was useful in distinguishing CL from UL, STUMP from CL, and LMS from STUMP. p53 expression was only seen in leiomyosarcomas. Bcl-2 was expressed more frequently in UL compared with LMS and STUMP indicating good prognosis. The expression rates of SMA, ER and PR in LMS were lower compared with CL and BL. CD expression differed between LMS and STUMP, LMS and UL, but not between STUMP and UL indicating that CD expression might be a marker for invasion or metastasis in LMS, and the expression of CD and ER/PR may be an especially useful immunohistochemical parameter to distinguish smooth muscle tumors in which histological features are ambiguous or borderline.

**P024**

**Tumors of the family of sarcomas Ewing's/PNET. 1990–2002**

A.I. Caballero, A.Y. Sánchez, C.J. Reno, S.D. García, T.M. Pérez, R. Ropero
*(Oncopediatric Service of Cuban National Oncology Institute)*

A total 30 patients (between 0 and 15 years of age) of the pediatric Service of the Cuban National Oncologist Institute were studied, with the diagnosis of Ewing/PNET between 1990–2002. 22 patients (73.3%) had typical Ewing's Sarcoma, 4 patients with PNET (13.3%), and 4 (13.4%) Askin's Tumors.

We intended to describe the clinical characteristics and the main factors presage found in this series, besides evaluating the results of the used different treatment modalities. It prevailed the male (1.5:1), the white race (98%) and the bony localizations. The main consultation reason was the pain and the palpable mass. The size tumor was one of the most significant factor worse presage (8 cm). The answer to the chemotherapy was also a factor predictor for the survival. In this time the service has been used 3 protocols (VACA-High Risk; VACA-Low Risk; P-6). The disease-free survival (DFS) to 3 years change in each one: 73%, 57%, 89% respectively. The radical surgeries prevailed to the conservative treatment being better the survival for these last ones. The global survival and the DFS to 5 and 2 years respectively were of 49% and 76%.

**P025**

**Quality of Life (QoL) in pediatric sarcoma patients: What do we know?**

G. Calaminus
*(Department of Pediatric Oncology, Hematology and Immunology, University Children's Hospital, Duesseldorf, Germany)*

*Background:* Sarcoma cover a wide range of disease (Ewing-, osteo- and soft-tissue sarcoma) with a specific treatment according to site and stage. Additionally the age of the affected patients within the pediatric population varies widely. Therefore factors influencing Quality of Life of the different disease groups are frequent.
*Questions are:* Are there disease specific QoL domains affected? What is the role of stage, age, gender and treatment ? How is long term QoL of these patients?
*Osteo-and Ewing sarcoma*: In the last year QoL research in this group of patients has been started more extensively. Investigators identified parameters such as treatment, mobility, age, mutilation, control of pain, educational status as factors influencing QoL after the end of treatment.
*Soft tissue sarcoma*: Published series are very limited and are mainly related to older children. Mutilation, pain, affection of body image and treatment related (irradiation) long term effects are described as factors influencing QoL in the long run.
*Standardized and prospective evaluation within disease specific trials are needed*: To determine the specific QoL areas affected and to identify patients and families of risk who need a more extensive psychosocial support and a more specific mental and physical rehabilitation.

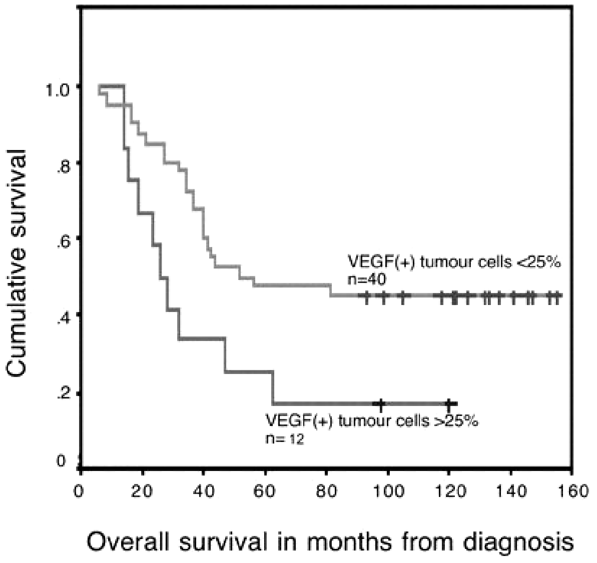

**P026**

**Is the biopsy really the best choice in initially localized unresectable rhabdomyosarcoma (RMS)? results of the Italian studies**

G. Cecchetto[1], F. De Corti[1], G. Bisogno[2], A. Ferrari[3], P. Dall'Igna[1], G. Scarzello[4], R. Alaggio[5], I. Zanetti[2], C. Boglino[6], M. Carli[2] for the Italian Cooperative Group
*([1]Department of Paediatrics, Division of Pediatric Surgery, University of Padua, Italy, [2]Department of Paediatrics, Division of Oncology, University of Padua, Italy, [3]Pediatric Oncology Unit, Istituto Tumori of Milan, Milan, Italy, [4]Division of Radiation Therapy, General Hospital of Padua, Italy, [5]Department of Oncologic Sciences, Pathology Unit, University of Padua, Italy, [6]Pediatric Surgery Department, Bambino Gesu, Rome, Italy)*

*Purpose:* Analysis of impact on survival of initial biopsy vs. macroscopic-residual resection (Debulking-Operation = DO) in IRS-Gr.III RMS, enrolled in the Italian studies (1979–2003).
*Patients/methods:* Surgical guidelines recommended biopsy and discouraged DO. Treatment after biopsy/DO was the same. 394 patients were evaluable. 323 underwent biopsy. Site: 34 Orbit, 31 HNnPM, 99 HNPM, 49 GUBP, 11 GUnBP, 27 extremities, 72 other. Histology: 84 Alveolar, 239 non-Alveolar. Tumor-size: 124 ≤5 cm, 199 >5 cm. 71 underwent DO. Site: 20 Orbit, 11 HNnPM, 9 HNPM, 7 GUBP, 1 GUnBP, 4 Extremities, 19 Other. Histology: 22 Alveolar, 49 non-Alveolar. Tumor-size: 36 ≤5 cm, 35 >5 cm.
*Results:* The 5-year OS is 68.7% after biopsy, 71.3% after DO ($p = 0.59$); PFS 57.5% after biopsy, 61.9% after DO ($p = 0.56$).

Patients NED after biopsy and after DO respectively: SITE. Orbit 32/34 (94%) and 15/20 (75%); HNnPM 23/31 (74%) and 9/11 (82%); HNPM 69/90 (70%) and 40/9 (44%); GUBP 38/49 (77%) and 7/7 (100%); GUnBP 11/11 (100%) and 1/1 (100%); Extremities 16/27 (59%) and 3/4 (75%); Other 36/72 (50%) and 10/19 (53%).
HISTOLOGY. Alveolar: 53/84 (63%) and 14/22 (64%); non-Alveolar: 172/239 (72%) and 35/49 (71%).
TUMOR-SIZE. ≤5 cm: 105/124 (85%) and 39/36 (83%); >5 cm: 121/199 (61%) and 19/35 (54%).
*Remarks:* No significant differences in outcome comparing patients who had biopsy or DO. Since the biopsy is less aggressive and dangerous, we believe it is preferable as initial approach for Gr.III RMS.

**P027**

**Ewing's sarcoma in children and adolescent: A report from a single institution in a developing country - 1970–1999**

A. Chandra, T.G. Sagar
*(Department of Medical Oncology, Cancer Institute (WIA), Chennai, India)*

*Background:* Treatment outcome of malignant bone sarcomas have changed over last three decades. Few studies have been reported from less developed countries.
*Material and methods:* We report here our experience of 171 evaluable cases in last 30 years(1970–1999) at Cancer Institute (WIA), a regional cancer centre in India. Event free survival rates(EFS) and 95% confidence interval were estimated by Kaplan Meier analysi and prognostic factors by logrank statistics and logistic regression method.
*Results:* Prior to 1985 suboptimal drugs were available and EFS was 6.3% at 5 years. During 1985–1999 with the advent of neoadjuvant chemotherapy and local radiotherapy/surgery, the survival improved to 53% in those who completed therapy. Metastatic disease at presentation and incomplete therapy are poor prognostic factors ($p = <0.05$).
*Conclusion:* Integrated multimodal care and achievement of survival figures are a challenges in a developing country.

**P028**

**Vascular endothelial growth factor and matrix metalloproteinase-9 expression by tumour cells influence patient survival in osteosarcoma**

R.M. Charity, A.F. Foukas, N.S. Deshmukh, R.J. Grimer, D.C. Mangham, S. Taylor
*(Royal Orthopeadic Hospital, Birmingham, UK)*

We investigate whether vascular endothelial growth factor (VEGF) and matrix metalloproteinase-9 (MMP-9) expression in osteosarcoma influence patient survival.

Fifty-five patients with stage II-B osteosarcoma of the distal femur/proximal tibia were followed-up for a minimum of 92 months. All had chemotherapy plus resection of tumour. Tissue from resected tumours was stained for VEGF/MMP-9 using immunohistochemical methods and the percentage of tumour cells expressing VEGF/MMP-9 was assessed. The relationship between VEGF/MMP-9 expression and survival was assessed using Kaplan-Meier survival curves.

At follow-up 33/55 (60%) patients were dead, all from metastatic disease. 25/52 (50%) tumours expressed VEGF and 37/51 (72%) expressed MMP-9. Patients with tumours expressing VEGF in more than 25% of their cells had significantly shorter overall ($p = 0.019$) and disease free survival ($p = 0.009$). Patients with tumours expressing MMP-9 also had significantly shorter overall ($p = 0.0042$) and disease free survival ($p = 0.0004$). There was an association between VEGF and MMP-9 expression ($p = 0.021$). Poor post-chemotherapy tumour necrosis did not significantly influence survival and was a factor independent of VEGF/MMP-9 expression.

VEGF and MMP-9 have roles in tumour neoangiogenesis, invasion, and metastasis. We have shown that they influence prognosis in osteosarcoma. New therapies targeting their actions warrant further study.

**P029**

**A model for drug resistance in osteosarcoma**

A. Marie Cleton-Jansen[1], C. Gomes[1,2], J. Anninga[3], N.N. Henriquez[4], G. van der Pluijm[4], E. Pauwels[2], P.C.W. Hogendoorn[1]
*([1]Departments of Pathology, [2]Nuclear Medicine, [3]Pediatrics, [4]Endocrinology, LUMC, Leiden, The Netherlands)*

*Purpose:* Resistance to chemotherapy is a problem in failing treatment for osteosarcoma. Studies in other tumours have identified multidrug resistance (MDR) genes, encoding ATP driven cellular pumps as the cause of failing chemotherapy. Due to pre-operative adjuvant chemotherapy protocols and because this tumour is relatively rare the role of these genes is unclear in osteosarcoma. To establish the role of known mechanisms for multidrug resistance and to identify new genes involved in osteosarcoma adjuvant therapy response we have developed an in vitro/vivo model.
*Methods:* A panel of osteosarcoma cell lines has been characterized for sensitivity to cytostatics. Dose dependent cell death induced by cytostatics is determined by FACS analysis. Expression of multidrug resistance (candidate) genes is determined by quantitative PCR. The biological activity of the MDR pumps is determined by radiopharmaceuticals (99mTc-Mibi and 99mTc-tetrofosmin) and by fluorescent reporters.
*Results:* The osteosarcoma cell lines have different sensitivity for chemotherapy. Cells can form tumours in nude mice and tumor progression and metastasis can be identified by a luciferase reporter gene using whole body bioluminescent reporter imaging (BLI) and in vivo MDR activity can be monitored by radiopharmaceuticals.
*Conclusion:* We have developed an in vivo model to study drug resistance in osteosarcoma.

**P030**

**Clinical and radiographic outcome of large segment prosthetic replacement for tumors of the distal femur and proximal tibia**

H.D. Morgan[1], S.S. Leopold[1], D. Hawkins[2], V. Bolejack[3], E. Conrad III[1]
*([1]University of Washington Orthopaedics and Sports Medicine, [2]Children's Hospital and Regional Medical Center, [1]University of Washington, Washington State, USA)*

*Purpose:* Our purpose was to achieve clinical and radiographic follow-up of patients who had modular distal femoral or proximal tibial prostheses inserted following the resection of a primary bone tumor around the knee.
*Materials/methods:* We retrospectively reviewed ninety-six patients who had distal femoral or proximal tibial megaprostheses implanted between 1985 and 2002. Surviving patients were followed for a minimum of two years; mean follow-up of all study patients was 60 months (range 0–226 months). At each follow-up, patients underwent clinical examination, Musculoskeletal Tumor Society (MSTS) rating system evaluation, an SF-36 questionnaire, and appropriate AP and lateral radiographs.
*Results:* The 96 patients ranged in age from 9–85 years, with a mean of 33 years. Fifty-three patients had a diagnosis of osteosarcoma, 32 had a non-osteosarcoma malignancy, and 11 had a benign aggressive tumor. Sixty-nine patients received a distal femoral prosthesis and 29 had a proximal tibial replacement. 79 of the prostheses were Howmedica Modular Oncology Prostheses. The overall patient survival was 84% at two years' follow-up, 72% at five years, and 66% at ten years, with younger patients having significantly better survival rates than patients over 18 years of age. Implant survival was 79% at two years, 66% at five years, and 51% at ten years, with proximal tibial implants faring better than distal femoral implants. Gender, length of resection, diagnosis (osteosarcoma vs. other), and the use of allograft prior to prosthetic implant did not significantly affect implant survival. There were 80 total complications, with aseptic loosening being the most common.
*Conclusion:* Modular prosthetic implants are a viable option for limb salvage following tumor resection; however, Implant survival at five and ten years may be lower than previously described.

**P031**

**Endoprosthetic vs. "Condyle-Sparing" intercalary allografts for distal femoral osteosarcoma: A comparison of long-term follow-up**

T.B. Rapp[1], M. Zimmel[2], E. U. Conrad III[3]
*([1]Loyola University Medical Center, [2]University of Chicago Medical School, [3]University of Washington Orthopaedics and Sports Medicine)*

*Introduction:* Tumor resection and limb salvage reconstruction are commonly performed for distal femoral osteosarcoma. No consensus exists regarding the optimal method of reconstruction. Further, there is little published data directly comparing different reconstructive techniques. This study directly compares the long term oncologic and functional results of two limb salvage techniques after resection of distal femoral osteosarcoma.
*Methods:* A retrospective chart and radiographic review was performed on patients treated for distal femoral osteosarcoma at a single institution by one senior surgeon.
*Results:* Between 1989–2000, 45 patients were treated with chemotherapy and limb salvage reconstruction for high grade distal femoral osteosarcoma. 27 patients received an intercalary "condyle-sparing" allograft, 18 received primary endoprosthetic replacement. Average follow-up was 6.5 years. The overall survival rate for the combined groups was 84%. There were two local recurrences in the allograft group, one in the endoprosthetic group. Ten patients (37%) in the allograft group required revision to an endoprosthesis. There was an average of 3.3 revision procedures per patient performed in the allograft group compared to 1.2 in the endoprosthetic group. There were no significant functional differences between the groups.
*Discussion/conclusion:* Limb salvage with a "condyle-sparing" intercalary allograft has the potential advantage of preserving the knee joint. In our series there was no significant difference in oncologic outcome when allografts were compared to endoprosthetic reconstruction. However 37% of the allograft patients required eventual conversion to an oncologic prosthesis and allograft patients had nearly 3× the number of revision surgeries. There were no functional differences discernable between the two groups.

**P032**

**The impact of surgery on outcome in pediatric non-rabdomyosarcoma soft tissue**

L.H.T. Sakamoto, C.M.L. da Costa, B. De Camargo, C.B.G. Antoneli
*(Pediatric Oncology Department, Hospital do Câncer AC Camargo, São Paulo, Brazil)*

Nonrhabdomyosarcoma Soft Tissue Sarcoma (NRSTS) comprises a heterogeneous class of tumours which biology is still not understood. Records of 32 NRSTS patients from a single institution were reviewed from January 1995 to December 2000, regarding demographic data, TNM staging, histological type and site of primary tumor.

Treatment were consisted of surgery, radiotherapy and chemotherapy (ifosfamide, actinomicyn D, vincristine, cyclophosphamide, doxorubicin, etoposide). Median age at diagnosis was 10.7 years (range: 1.9–220.4 months). Primary site was extremities (37.5%), head and neck (28%), followed by pelvic area (12.5%). The most common histological subtypes were fibrosarcoma (47%), alveolar sarcoma (12.5%), leiomyosarcoma (9.5%). Complete resection was performed in 65.5% of patients as first treatment. Chemotherapy was done in 31.3% and 21.9% were irradiated. Five-year overall and event-free survival were 80.3% and 76.5%, respectively. Patients submitted only to surgery overall survival was statistically higher than children treated with multimodal therapy (88.9% and 63.6%; $p = 0.03$). The high survival of patients submitted to complete tumour resection reveals the importance of surgery for management of NRSTS. Chemotherapy and radiotherapy had no influence in improving surgery resection and in overall survival.

### P033

**Intensive chemotherapy is not better than VAC regimen for rhabdomyosarcoma in children and adolescents**

L.H.T Sakamoto, C.M.L. Da Costa, B. De Camargo, C.B.G. Antoneli
*(Pediatric Oncology Department, Hospital do Câncer AC Camargo, São Paulo, Brazil)*

Several studies have reported the efficacy of different combinations of chemotherapy agents in the systemic disease control. Intensive chemotherapy has been suggested to improve prognosis.

To determine if intensive aggressive chemotherapy scheme is better than pioneer regimen VAC records from 38 children and adolescents with rhabdomyosarcoma admitted in our institution during the period 1995–2000 were reviewed. The median age at diagnosis was 7.6 years. Local site was 8 cases head and neck, 7 parameningeal, 7 genitourinary, 7 extremity and 9 others. Histology was alveolar in 15.5% of cases. Excluding 8 patients stage IV, 15 (50%) received VAC (vincristine, actinomycin, cyclophosphamide) and 15 (50%) received intensive chemotherapy (VAC plus ifosfamide, doxorubicin, etoposide). Clinical and tumor characteristics were similar among the both groups. ($p > 0.05$). Five-year overall survival and event-free survival were 55.5% and 54.1%, respectively. Age more than 10 years, alveolar histology and TNM stage constituted prognostic factors for overall survival ($p < 0.05$). Five-year overall survival was not different between patients who received VAC vs. who received intensive chemotherapy (72.7% vs. 58.3%, respectively; $p = 0.42$). Despite choice of treatment was not randomized VAC regimen suggests as good as intensive chemotherapy with less toxicity and significant less cost.

### P034

**Is early diagnosis an important prognostic factor in osteosarcoma?**

K.S. Rodrigues, C.M.L da Costa, M.R. Latorre, B. de Camargo
*(Pediatric Oncology Department, Hospital do Cancer AC Camargo, São Paulo, Brazil)*

Early diagnosis has been suggested as a factor that could improve survival of children with cancer. One hundred and sixty five children and adolescents with osteosarcoma were referred to the Pediatric Department, Hospital do Cancer, Sao Paulo, Brazil during 1991–2000. The M:F ratio was 1.2, mean age was 13.3. Interval between symptom onset and diagnosis (lag time) vary from 7.5 days to 48 months (ms) mean of 3.5 ms. First symptoms were bone pain, tumor mass, fracture with a mean lag time of 3.1, 5.0, 4.5 ms respectively ($p = 0.064$). Age and bone pain as first symptom was significantly correlated with lag time. When bone pain was the first symptom there was a reduction in lag time of 1.7 ms ($p = 0.031$). The older the patient the longer was the lag time. Lag time increased 1.9 ms each year of life ($p = 0.024$). Patients with localized disease had a mean lag time of 3.2 ms while metastatic disease 3.9 ms. ($p = 0.303$). There were no difference between 5-year overall survival in patients with a shorter and longer lag time (<3 ms: 46.1% vs. >3 ms: 47.5; $p = 0.45$). The relationship between lag time and prognosis is complex and many factors play important roles on survival.

### P035

**Osteosarcoma and Ewing's sarcoma of the forearm and hand**

W. Daecke[1], A.-K. Martini[1], H. Jürgens[2], S. Bielack[2], L. Bernd[1]
*([1]Orthopädische Universitätsklinik Heidelberg1, Schlierbacher Landstraße 200 A, 69118 Heidelberg, Germany, [2]Pädiatrische Haematologie und Onkologie, Westfälische Wilhelms Universität, Münster, Germany)*

*Introduction:* Knowledge and prognosis of osteosarcoma (os) and ewing sarcoma (ews) of hand and forearm is limited.

*Method:* On basis of the Cooperative-Osteosarcom-Study-Group (COSS) and the Ewing-Sarcoma-Study-Group (EICESS) 39 patients with os and 33 patients with ews of hand and forearm were assessed. Epidemiological, clinical, histological data and the survival rates were evaluated. For patients with os therapy consisted of surgical tumour resection and (neo)-adjuvant chemotherapy. Patients with low grade os received local resection only. All patients with ews had neoadjuvant chemotherapy. Local therapy in patients with ews was surgery in 7, radiation in 7 and a combination of both 19 subjects.

*Results:* For 33 patients with high-grade central os 5-year overall/event free survival was $86.2 \pm 6.4\%/65.4 \pm 9.6\%$, respectively. 5 of 8 patients with secondary metastasis were in remission. 4 patients died of disease and two patients died of chemotherapy related complications. In 33 patients with ews 5-year overall/event free survival was $84.1 \pm 6.5\%/71.3 \pm 8.1\%$. Out of 7 patients with secondary ews metastasis 6 died. Altogether 8 of 33 patients with ews died of disease.

*Discussion:* By utilising current therapeutic protocols in patients with os or ews of the hand and forearm survival-rates are remarkably high.

### P036

**Epithelioid sarcoma with SYT-SSX1 fusion gene expression. Molecular and cytogenetic analysis**

C. de Torres[1], T.M. Cardesa[2], V. Cusí[3], S. Rodríguez-Perales[4], J.C. Cigudosa[4], J. Mora[2]
*([1]Laboratori de biologia molecular dels tumors del desenvolupament, [2]Oncologia Pediàtrica, [3]Anatomia Patològica, Hospital Sant Joan de Déu, Barcelona, Spain, [4]Centro Nacional de Investigaciones Oncológicas, Madrid, Spain)*

Epithelioid sarcoma (ES) is a rare malignant soft tissue tumor of unknown histogenesis, characterized by an epithelioid morphology of tumor cells, and co-expression of epithelial and mesenchymal lineage proteins.

Three neoplasms showing mesenchymal and epithelial differentiation of tumor cells have similar 18q11 breakpoints: mesothelioma t (3;18)(p14;q11); t (5;18)(p15;q11), synovial sarcoma (SS) t (X;18)(p11.2;q11.2) and ES 18q11 breakpoint. Expression of SYT-SSX fusion genes has been previously investigated in ES with negative results.

A 14-year-old male presented with a painful, slowly growing mass on the anterior aspect of his right forearm. Histopathologic evaluation of the excised lesion demonstrated the presence of epithelioid cells exhibiting slight nuclear atypia and a nodular growth pattern of the tumor. Immunohistochemical staining was consistent with the diagnosis of ES (positive for vimentine, cytokeratins, EMA and CD34).

Expression of SYT-SSX fusion genes was analyzed by conventional and real-time RT-PCR analysis. Both showed a low level of SYT-SSX1 amplification in ES, when compared to the positive control (synovial sarcoma). FISH analysis was performed on paraffin sections of the tumor and confirmed the presence of the SYT-SSX1 fusion gene in a small proportion of tumor cells. This finding supports a likely common histogenetic background between SS and ES.

### P037

**Experiences with the "clavicula pro humero" operation in malignant bone tumors of the proximal humerus in adolescents and adults**

P.M. de Zwart, F. Maurer, K. Weise
*(BG-Unfallklinik Tübingen, Germany)*

Malignant tumors in the proximal part of the humerus can nowadays be treated by a limb saving procedure in many cases, because of improved regimen of chemotherapy, radiotherapy and because of better reconstruction of the defect by arthroplasty or by using bone transplants, such as the clavicula pro humero operation, firstly presented by Winkelmann.

An 18-year-old young man presented with shoulder pain. The diagnosis was osteosarcoma of the left proximal humerus. After neoadjuvant chemotherapy a wide resection of the sarcomatous proximal humerus was done. The defect was bridged by a clavicula pro humero operation. Internal fixation from the clavicle to the distal humerus was made with an AO plate and screws. No complications were observed during the postoperative period. A good bone healing and even an enlargement of the clavicula could be demonstrated by X-rays. Range of motion of the shoulder was 80 degrees flexion and abduction, 30 degrees external rotation, and 80 degrees internal rotation. A comparison is made between the functional advantages of this operation and other forms of reconstruction like arthroplasty.

### P038

**The Ewing Tumour (ET)-antigen ETAA 16: Insights in ET-specificity and functional evidence**

U. Dirksen[1], A. Borowski[2], S. Borkens[1], C. Bury[1], U. Göbel[1]
*([1]Kinderonkologie, Hämatologie und Immunologie, Universitätsklinikum Düsseldorf; Germany, [2]Anatomisches Institut, Universität Bonn, Germany)*

ETAA16 is a 60–95 kD protein expressed on the majority of Ewing-tumour-cells (ETC). Non-ETC obtained from healthy tissues or tumour were tested negative for ETAA16 surface expression. Nothing is known about the biological role of ETAA16 in ETC. An anti-ETAA16 antibody is available. ETC have been shown to release Interleukin (IL) 8, vascular endothelial growth factor (VEGF), IL6 and monocyte chemotactic protein (MCP)-1. In order to test the influence of AK16 on cytokine release in human ETC, RDES- and VH64-celllines were cultured in the presence or absence of different concentrations of AK16. Cytokine release was tested by ELISA and cell death by propidium iodine staining and binding of Annexin-V at 24, 48, 56 and 72 hours of culture. Our results indicate, that anti-ETAA16 antibody inhibits the release of VEGF, IL8, MCP-1 and IL6 after 48 h of culture. Apoptosis occured within 56–72 hours of culture. As controls non-ET-celllines were treated equally.
*Conclusion:* ETAA16 is a ET-specific antigen. Binding of AK16 to the ETAA16 antigen results in an impaired function and earlys cell death in ET-celllines. Although the apoptosis-specific signal appeared later than the down-regulation of cytokine release, we can not exclude that the impaired cytokine release is a secondary effect.

### P039

**Alveolar rhabdomyosarcoma with bone and bone marrow involvement: The experience with diagnosis and treatment**

E. Drahokoupilova[1], D. Sumerauer[1], E. Kabickova[1], O. Belohlavek[2], R. Kodet[3], L. Krskova[3], J. Stary[1]
*([1]Department of Ped. Hematology and Oncology Charles University and University Hospital Motol, Prague, Czech Republic, [2]Department of Nuclear Medicine, Na Homolce Hospital, Prague, Czech Republic, [3]Department of Pathology and Molecular Medicine, 2nd Medical School Charles University Prague, Czech Republic)*

*Background:* The bone marrow infiltration and bone metastases are very unfavourable prognostic factors in the treatment and survival of children with alveolar rhabdomyosarcoma. The treatment is very difficult and the chance for achieving the long-term remission is very small.
*Patients/Methods:* We report three patients (age >10 years) with alveolar rhabdomyosarcoma (diagnosis was confirmed by imunohistochemical analysis of MYOD1 and PAX-FKHR transcript by RT-PCR) and with diffuse bone marrow involvement by neoplastic cells and bone metastases (1 patient with and 2 without the evidence of primary tumour by the using of FDG-PET and radiological methods). Patients were treated with 9–11 cycles of chemotherapy: topotecan, cyclophosphamide in alteration with VAC (vincristine, actinomycin D, cyclophosphamide), followed by local radiotherapy and with maintenance therapy by vinorelbine.
*Results:* Two patients achieved complete remission and 1 child achieved very good partial remission, median follow-up was 15.3 months and median time to disease progression was 11.6 months (range 10–13 months). The toxicity of this chemotherapy regimen was acceptable.
*Conclusions:* The regimen topotecan, cyclophosphamide alterated with VAC is effective and good tolerated combination for the treatment of poor-risk alveolar rhabdomyosarcoma patients.

### P040

**Microarray analysis to identify target genes of PAX3-FKHR in rhabdomyosarcoma after siRNA mediated down-regulation**

M. Ebauer, M. Wachtel, B.W. Schäfer
*(University Children's Hospital Zürich, Division of Oncology, Zürich, Switzerland)*

Rhabdomyosarcoma is an aggressive childhood cancer derived from skeletal muscle cells with two major histological subgroups, the embryonal (eRMS) and the alveolar (aRMS) form. ARMS is associated with the chromosomal translocation t(2;13) or t(1;13) which generates the chimaeric transcription factor PAX3/FKHR. Direct transcriptional targets mediating the oncogenic effects of this fusion protein are largely unknown.

In the present work, we analyzed the effect of PAX3/FKHR downregulation by siRNA to identify novel targets and characterize effects on cellular viability. Transfection of small interfering RNA (siRNA) for PAX3/FKHR reduced its expression on mRNA and protein levels and resulted in decreased cellular viability.

To identify PAX3/FKHR target genes in RMS, the alveolar line Rh4 was chosen as a model and microarray analyses were performed after downregulation of PAX3/FKHR. Numerous genes involved in muscle function such as troponin C2 were induced by low levels of PAX3/FKHR, suggesting that PAX3/FKHR was repressing their expression. Several genes were downregulated, among them are genes that play a role in apoptosis and cell growth such as TFAP2beta and cannabinoid receptor 1.

The RNAi technique appears to be a promising tool for analysis of PAX3/FKHR gene function and could also be used to identify novel therapeutic targets for aRMS.

### P041

**Treatment of non-metastatic rhabdomyosarcoma and other non-rhabdomyosarcoma soft tissue tumors of childhood and adolescence**

S. El-Badawy, H. Hussein, G. Attia
*(National Cancer Institute, Cairo, Egypt)*

*Background:* At NCI Egypt, soft tissue sarcoma represent 3.75% of total malignancies & 27.6% of these occur in pediatric age group.
*Methods:* 56 children and adolescents, treated by upfront chemotherapy and local treatment at week 9. Fifty four patients received the high risk regimen of chemotherapy consisted of alternating 6 drugs.
*Results:* Most common primary sites were the extremity (28.6%), followed by orbit, trunk, parameningeal sites (12.5% each), head & neck 10.7%, bladder & prostate 7.1%, retroperitoneal 7.1%. Tumor size more than 5 cm was encountered in 72.5%. Sixty two percent had embryonal RMS and 27% were alveolar subtype. Forty three percent of patients showed CR, 46% good PR, 7% PR, while 2% SD. The 2-year OAS & DFS for the whole group was 73 ± 9% & 53 ± 9% respectively. Sixteen patients relapsed (7 locoregional, 6 distant & 3 both). DFS was significantly affected by tumor size in favour of those with tumors <5 cm ($P = 0.03$). Local failure free survival was 61 ± 13%. On multivariate analysis radiotherapy showed statistical significance ($P = 0.04$). Only one patient died of fungal pneumonia.
*Conclusion:* Chemotherapy used in this study was well tolerated. Radiation therapy is an effective modality should be refined to reduce late sequlae by the use of IMRT & brachytherapy.

### P042

**The significance of isolated limb perfusion with TNF-alpha and melphalan in adults and children**

S. Elezkurtay, P.-U. Tunn, P. Hohenberger, C. Kettelhack, P.M. Schlag
*(Clinic for Surgery and Surgical Oncology, Charité Campus Buch, Berlin, Germany)*

*Purpose:* We report our 12-year experience with isolated limb perfusion (ILP) for limb salvage therapy of locally advanced soft tissue sarcoma.
*Patients and methods:* Between 1993 and 2004 105 patients with soft tissue sarcoma (13% of all patients) received isolated limb perfusion. Age was between 10 and 80 years (Median 54 years) with a predominance of female individuals (1.3:1). 3.8% of patients were children. ILP was carried out most commonly in the lower limbs (84%). Resection of the tumor was performed after a six week period in 97% of patients.
*Results:* Limb salvage could be achieved in 78% of patients after ILP. The response rate was 69%. Local recurrence was seen in 11% of cases. The 5 year overall survival was 68% in accordance with the survival of all patients treated with soft tissue sarcoma ($n = 815$). Secondary amputation was most frequently indicated for synovial sarcoma of the foot. There was no case of amputation, local recurrence or therapy related complications in childhood age.
*Discussion:* Isolated limb perfusion is the most effective neoadjuvant limb sparing modality for locally advanced soft tissue sarcoma in newly diagnosed as well as recurrent lesions. First experiences in childhood conditions are encouraging.

### P043

**Dose intensity of chemotherapy in osteosarcoma in the Cooperative Osteosarcoma Study Group (COSS) trials**

M. Eselgrim[1], H. Grunert[1], M. Kevric[1], H. Bürger[2], H. Jürgens[1], T. Kühne[4], R. Kotz[5], S. Lang[6], R. Mayer-Steinacker[7], A. Zoubek[8], S. Bielack[1]
*([1]Universitätsklinikum Münster, Pädiatrische Hämatologie und Onkologie, [2]Institut für Pathologie, [3]Allgemeine Orthopädie, [4]Universitätskinderspital beider Basel, Switzerland, [5]Orthopädische Universitätsklinik, [6]Klinisches Institut für Pathologie, Universitätsklinik Wien, [7]Universitätsklinikum Ulm, [8]St. Anna-Spital, Wien, Austria)*

*Background:* The prognostic relevance of chemotherapy dose intensity in osteosarcoma is still under discussion. This analysis investigated whether higher dose intensities correlate with better outcomes.
*Procedure:* Ninehundredseventeen COSS patients <40 years with primary, high-grade-central, non-metastatic extremity osteosarcoma were evaluated for potential correlations between chemotherapy dose intensity during the first 200 days of treatment (DI200) and overall and event-free survival. Scheduled therapy consisted of intensive mutidrug chemotherapy and surgery. The analysis focused on methotrexate, doxorubicin, cisplatin, and ifosfamide, which were given to the majority of patients. Multivariate analyses including well-known prognostic factors were added to complete this investigation.
*Results:* Until day 200, patients had received $80684 \pm 26073\,mg/m^2$ methotrexate, $242 \pm 69.2\,mg/m^2$ doxorubicin, $324 \pm 133\,mg/m^2$ cisplatin and $13880 \pm 9802\,mg/m^2$ ifosfamide (mean ± SD). After a median follow-up of 6.6 (0.02–22.1) years, actuarial overall-suvival (from day 200) was 77%, with an event-free survival rate of 66%. There was no correlation between a higher dose intensity of any particular agent or of overall treatment and better outcomes in either uni- or multivariate analyses.
*Conclusions:* In an overall setting of intensive multidrug treatment for osteosarcoma, we could not prove that higher dose intensities correlate with better outcomes.

### P044

**Localized pelvic embryonal rhabdomyosarcoma with bone invasion in a 14 y old girl. Ten year disease free follow-up after multimodal treatment in the CWS 91 study**

G.U. Exner, A.R. von Hochstetter, H.P. Honegger, N. Lombriser, J. Streuli, E. Wight
*(Uniklinik Balgrist, University of Zürich, and Stadtspital Triemli, Zürich, Switzerland)*

*Rationale:* Rhabdomyosarcoma of the pelvis has a very poor prognosis. This case is presented to encourage multimodal treatment and further evaluate the contribution of aggressive extralesional surgery for such patients.
*Patient and methods:* At age 14 years the patient presented with inguinal pain of 6 months duration considered tendinopathy from horseback riding. At presentation she had a large inguinal mass and a swollen right leg. Standard X-ray showed destruction of the pubic and fracture of the ischiac bone. The tumor volume was about 750 ml from MRI studies. Biopsy revealed a partially myxoid embryonal rhabdomyosarcoma.

The patient was enrolled in the EVAIA branch of the CWS 91 study. Following 30.1 Gy of percutaneous radiotherapy an extralesional resection of the affected pubic and ischiac bones including the anterior part of the acetabulum was achieved with min. margins of 6 mm. Islands of vital tumor cells were found in the largely necrotic specimen. Postoperatively the patient completed the full course of the CWS 91 regime and radiotherapy was added to 52.8 Gy.

At present at 10 y f/u the patient is free of tumor. She has lymph edema of the right leg, but otherwise is able to carry out her daily activities; she continues horseback riding including jumping despite complete loss of adductor muscle function. She has no pain and has normal bladder function. She is working as laboratory technician and is presently pregnant in the 28th week of a so far uneventful gestation. According to the patient she is highly satisfied with the result and her quality of life.

### P045

**Adult-type soft tissue sarcomas in the pediatric age: The experience of the Istituto Nazionale Tumori, Milan, Italy**

A. Ferrari, M. Casanova, P. Collini, C. Meazza, R. Luksch, M. Massimino, G. Cefalo, M. Terenziani, F. Spreafico, D. Polastri, M. Podda, A. Rognone, D. Catania, E. Zaffignani, O. Sironi, L. Gandola, A. Gronchi
*(Istituto Nazionale per lo Studio e la Cura dei Tumori, Milan, Italy)*

*Introduction:* In the heterogeneous group of non-rhabdomyosarcoma soft tissue sarcomas (NRSTS), it is important to identify groups as homogeneous as possible; we define "adult-type" NRSTS those histotypes typical of adulthood, definitely malignant, with morphological features resembling differentiated/mature tissues (excluding infantile fibrosarcoma, borderline tumors, small-round-cell-tumors, i.e. RMS, extraosseus pPNET/Ewing and DSRCT). Therefore, this definition includes adult-type fibrosarcoma, MPNST, epithelioid-sarcoma, leiomyosarcoma, clear-cell-sarcoma, liposarcoma, alveolar-soft-part-sarcoma, malignant-fibrous-histiocytoma, malignant-hemangiopericytoma, angiosarcoma, mesenchymal-chondrosarcoma, dermatofibrosarcoma.
*Methods:* We report on 179 patients <18 yrs treated over a 25-year period. Surgery was the mainstay of treatment; 73 patients received radiotherapy, 114 chemotherapy (70 adjuvant). Results: 5 yr-OS was 88% in IRS Group I, 79% in Group II, 52% in Group III, 17% in Group IV. Outcome was unsatisfactory in patients with large and high-grade tumors even after gross resection (MFS 36%) and adjuvant chemotherapy seemed to improve the.
*Results*. In the group of initially-unresected cases (EFS 45%), patients who responded well to chemotherapy and underwent complete resection had EFS ~70%. The chemotherapy response rate was 58% considering also minor responses.
*Conclusion:* The identification of prognostic variables should enable risk-adapted therapies to be planned. Patients with initially-unresectable disease and those with resected large and high-grade tumors are at high-risk of metastases and might benefit of intensive chemotherapy.

### P046

**Synovial sarcoma in adolescents and young adults. The experience of the Istituto Nazionale Tumori, Milan, Italy**

A. Ferrari, A. Gronchi, M. Casanova, C. Meazza, L. Gandola, P. Collini, M. Massimino, R. Luksch, G. Cefalo, M. Terenziani, F. Spreafico, D. Polastri, M. Podda, R. Bertulli, P.G. Casali
*(Istituto Nazionale Tumori, Milan, Italy)*

*Purpose:* We previously reported on 271 SS patients of all ages, treated at our institution over a 30-year period (*Ferrari et al., Cancer, 2004*). Herein, we focus on the 83 AYA (age 17–30yrs), comparing clinical findings and treatment results to those of younger and older patients.
*Patients:* Among the 83 AYA, 60 received initial macroscopic resection, 14 had locally-advanced disease and 3 had metastases at onset. Tumor size was >5 cm in 60% of cases (49% in children and 73% in older adults). Among the AYA who had initial gross surgery, 45% received radiotherapy (58% in children and 49% in older adults); adjuvant chemotherapy was administered to 20% (78% in children and 14% in older adults).
*Results:* For initially-resected AYA, 5 yr-EFS was 40% (66% for children, 31% older adults). For AYA with advanced disease, 5 yr-EFS was 55% and chemotherapy-response rate was 60%.
*Conclusions:* Older age correlates with a worse prognosis and this may be partially due to the greater use of chemotherapy in the younger patients (treated by pediatricians as rhabdomyosarcoma). Chemotherapy may play a more important part in SS than in other adult sarcomas. AYA could probably receive the better treatment within experienced institutions that enrol patients into clinical trails.

### P047

**Epithelioid sarcoma in children and adolescents: A report from the soft tissue sarcoma Italian cooperative group**

M. Casanova[1], A. Ferrari[1], G. Bisogno[2], P. Collini[1], R. Alaggio[2], A. Gronchi[1], C. Meazza[1], G. Cecchetto[2], A. Garaventa[3], A. Di Cataldo[4], P. Indolfi[5], M. Carli[2]
*([1]Italian Cooperative Group on Soft Tissue Sarcoma, Milano, [2]Padova, [3]Genova, [4]Catania, [5]Napoli, Italy)*

*Objectives:* To contribute further information to natural history and management of epithelioid sarcoma in children and adolescents.

*Methods:* We analyzed 24 patients (3–18yrs) treated between 1988–2002. Primary site was extremity in 83%, size ranged between 1–10 cm (median 3); 2 patients had nodal involvement, 1 lung metastases; 14 patients were IRSgroup I, 4 group II, 5 group III, 1 group IV.
*Results:* Radiotherapy was delivered to 6 patients, chemotherapy to 7 (N1 and group III–IV patients). Response to chemotherapy was achieved in 3/6 evaluable pts (2 pathological complete remission). Five pts underwent delayed complete resection. Five-year EFS and OS were 65.3% and 95.8% (median follow-up 51 mos). Nine patients relapsed (3–124 mos, median 12): 7 local (all alive after salvage treatment), 1 local + metastases, 1 metastases (the 2 pts who developed metastases died of disease). EFS correlated with age (81.8% ≤10 yrs, 52.7% >10 yrs), site (70.0% extremity, 37.5% other), T-status (71.8% T1, 50.0% T2), but not with size.
*Conclusions:* Despite the high rate of local relapse, the prognosis was good. Chemotherapy may achieve a response in unresectable disease and enhance surgery. Unlike other pediatric soft tissue sarcomas, size seems to be an unreliable predictor of prognosis. Pathological review will be presented.

### P048

### The upcoming European protocol on pediatric non-rhabdomyosarcoma soft tissue sarcomas

A. Ferrari, M. Casanova, I.B. Brecht, E. Koscielniak, B. Brennan, N. Francotte, M. Van Noesel, D. Orbach, G. Ljungman, A. Schuck, H. Martelli, A. Kelsey, R. Alaggio, A. Rosolen, H. Brissè, K. McHugh, G.L. De Salvo, C. Bergeron, G. Bisogno, C. Int-Veen, M. Carli, O. Oberlin, M. Stevens, J. Treuner
*(European Pediatric Soft Tissue Sarcoma Study Group (EpSSG) Pediatric Oncology Unit, Istituto Nazionale Tumori, Milan, Italy)*

*Purpose:* The three cooperative European groups (SIOP-MMT, CWS and AIEOP-STSC) recently joined together as the European paediatric Soft tissue sarcoma Study Group (EpSSG). As concern Non-Rhabdomyosarcoma-Soft-Tissue-Sarcoma (NRSTS), the EpSSG is developing the first European study to focus specifically on pediatric NRSTS.
*Methods.* The study includes two prospective clinical trials, one on synovial sarcoma and one on "adult-type" NRSTS. Only general guidelines will be given for the so-called "other histotypes". First objective of the study is to make uniform the treatment of NRSTS patients in Europe. Main objectives of the prospective trails are the investigation of the role of an ifosfamide-doxorubicin regimen in improving the response rate in patients with unresectable tumors, the impact of the omission of adjuvant chemotherapy in patients with low-risk synovial sarcoma (IRS group I, tumour <5 cm), the role of adjuvant chemotherapy in IRS group I–II, G3, size >5 cm adult-type NRSTS patients in improving the MFS and the OS, the prospective evaluation of clinical/pathological prognostic factors (in particular the tumour grade).
*Conclusions:* Increasing European collaboration should allow the exploration of better treatment strategies for children with NRSTS, for which in the past treatments were generally adapted from those drawn up for rhabdomyosarcoma.

### P049

### Could adjuvant chemotherapy have a role in surgically-resected adult-type soft tissue sarcomas of children and adolescents?

A. Ferrari[1], I.B. Brecht[2], E. Koscielniak[2], M. Casanova[1], A. Scagnellato[3], G. Bisogno[3], R. Alaggio[3], G. Cecchetto[3], C. Meazza[1], C. Int-Veen[2], S. Kirsch[2], T. Dantonello[2], M. Carli[3], J. Treuner[2]
*([1]Italian Cooperative Group on Soft Tissue Sarcoma, Milan, [2]Padua, Italy, [3]The German Soft Tissue Sarcoma Study Group (CWS), Olgahospital, Stuttgart, Germany)*

*Purpose:* This analysis aims to evaluate whether adjuvant chemotherapy can be recommended for high-risk, surgically-resected, adult-type non-rhabdomyosarcoma soft tissue sarcomas (NRSTS). The Italian and German Cooperative Groups reviewed their databases, analyzing patients classified as IRS group I–II, with high grade tumor (G3) larger than 5 cm in size.
*Methods:* The analysis included 36 patients (39% MPNST), and compared the clinical features and outcome of the group of 21 patients who received chemotherapy versus the group of 15 patients treated with local therapies only.
*Results:* For the series as a whole, 5yr-EFS, MFS and OS were 26.2%, 34.0% and 37.5%, respectively. In patients treated with chemotherapy, MFS and OS were 49.5% and 41.5% (median time to relapse: 13 months). In patients who did not receive chemotherapy, MFS and OS were 0% and 23.8% (median time to relapse: 3 months).
*Conclusion:* The role of adjuvant chemotherapy in NRSTS is still uncertain, however the current analysis showed that: (1) despite the globally good prognosis of grossly-resected cases (5yr-EFS = 72%, OS = 86% [Ferrari et al., J Clin Oncol 2005]), patients with G3 and large-size have a high risk of metastatic spread, (2) MFS would seem better in patients who had chemotherapy. Given these results, and in accordance with some recent suggestions coming from the literature on adult sarcomas, the new European pediatric Soft Tissue Sarcoma Study Group (EpSSG) NRSTS protocol will recommend adjuvant chemotherapy in high-risk surgically-resected patients.

### P050

### Co-stimulation through CD137 enhances interleukin-2 driven immune response against Ewing family tumor cells

J. Foell, C. Kuehnoel, O. Diwan, G. Horneff, S. Martin
*(Martin-Luther-University Halle-Wittenberg, Department of Pediatrics, Halle, Germany)*

CD137 (4-1BB) is an inducible T cell costimulatory receptor and a member of the tumor necrosis factor receptor (TNFR) super family. It is mainly expressed on activated T cells and activated natural killer (NK) cells. Binding of CD137 to the natural ligand (4-1BBL) or to agonistic monoclonal antibodies induces T cell co-stimulation and leads to enhanced proliferation and cytokine production. We report here that co-stimulation with an monoclonal antibody against CD137 or transfection of tumor cells with human 4-1BBL enhances the interleukin-2 (IL-2) induced immune response against Ewing family tumor (EFT) cells in vitro. Additionally, the incubation of circulating T cells from a patient with progressively growing EFT with autologous tumor cells in the presence of anti-CD137 mAb and IL-2 enhanced the cytolytic T cell response against these tumor cells. The stimulation of the CD137/4-1BBL pathway led to an expansion and activation of CD8+ and CD4+ T cells and

NK cells. Under these conditions, growth of autologous tumor cells was nearly completely suppressed. These data suggest that in vitro stimulation of T and NK cells through CD137 in combination with IL-2 followed by adoptive transfer of activated T and NK cells might be an interesting option for the treatment of patients with advanced metastatic cancer including EFT.

**P051**

**Topotecan monotherapy and Topotecan/Thiotepa combination therapy with and without stem cell rescue in patients with advanced malignant peripheral neuroectodermal tumors (PNET): A pilot study of toxicity and efficacy**

S. Hess[1], C. Kuehnoel[1], J. Föll[1], A. Wawer[2], S. Burdach[1]
*([1]Martin-Luther University of Halle-Wittenberg, Division of Pediatric Hematology/Oncology, Department of Pediatrics, 06097 Halle, Germany, [2]Children's Hospital, Medical Center, Munich University of Technology, Munich, Germany)*

A pilot study was designed to determine the toxicity of topotecan (TPT) and to evaluate the efficacy of TPT in combination with thiotepa in patients with advanced malignant PNET.

Three patients were treated with TPT by interpatient dose escalation with 1.2, 1.5 and 2 mg/m$^2$ for 5 days as continuous intravenous infusion (CIVI). All patients received granulocyte-colony stimulating factor, one patient received aPBSC. Thereafter five cycles of TPT 2 mg/m$^2$ on days −7 to −3 and thiotepa 100 mg/m$^2$ on days −7 to −3 followed by aPBSC rescue on day 0 were given to three patients.

Nonhematologic toxicity was mild to moderate, myelosuppression was the main toxicity. Grade 4 neutropenia was seen in all patients. One of three patients with monotherapy had no response, one became free of pain for 14 days and the third showed stable disease (SD). One patient received combination with thiotepa achieved partial remission, one of three showed complete remission. The third patient showed SD after first but progression after 2 cycle.

TPT monotherapy is a palliative regimen with acceptable nonhematologic and manageable hematologic toxicity. TPT/ thiotepa appears to be a tolerable myeloablative regimen with antitumor activity. Hematologic toxicity is reduced by use of cytokines and aPBSC rescue.

**P052**

**Role of primary lung metastases in RMS-like tumors in children and adolescents: Results of the CWS-study group**

G. Friedel[1], S. Veit[1], S. Kirsch[2], T. Dantonello[2], I.B. Brecht[2], C. Int-Veen[2], A. Schuck[3], P. Winkler[2], I. Leuschner[4], S. Bielack[5], T. Klingebiel[6], E. Koscielniak[2], J. Treuner[2], H. Toomes[1]
*(CWS Study Group: [1]Department of Thoracic Surgery, Klinik Schillerhoehe, Gerlingen, Germany, [2]Department of Pediatric Haematology and Oncology, Olgahospital, Stuttgart, Germany, [3]Department of Radiotherapy, University of Münster, Münster, Germany, [4]Department of Paidopathology, University of Kiel, Kiel, Germany, [5]Department of Pediatric Haematology and Oncology, University of Münster, Münster, Germany, [6]Department of Pediatric Haematology and Oncology, University of Frankfurt, Frankfurt, Germany)*

*Purpose:* Analysis of data from patients with RMS-like tumors and primary metastases confined to the lung treated within the CWS 81–96 trials.

*Methods:* Evaluation of 75 completely documented patients treated between 3/1981 and 10/2002. Median follow up was 58 months (survivors). Treatment of the primary tumor consisted of local and systemic therapy according to the trial guidelines.

*Results:* The collective is composed of 38 patients with RME, 6 with RMA, 2 RMS, 3 EES, 14 pPNET and 12 SySa patients with different localisation of the primary tumor. Patients with pPNET had significantly more pulmonary metastases than RME patients ($p = 0.02$). 26 patients are alive in complete remission, 2 showed partial remission, 2 progressive disease, 1 relapsed and 42 died of disease.

The 5-yrs OS and EFS improved significantly from CWS-81 to CWS-96 (23% vs. 60% and 8% vs. 39%). Compared to all CWS-96 stage IV-patients (OS 31%, EFS 25%) they showed better results approximately to those in the RMS-like High Risk group (OS 68%, EFS 57%). Younger patients or those with small tumors had a significantly better outcome ($p = 0.01$ and 0.03). The impact of lung irradiation and resection of pulmonary metastases will be discussed.

*Discussion:* Results of patients with RMS-like tumors and with primary metastases only in the lung are comparable to those in the High Risk group rather than to stage IV-patients. Favourable risk factors and adequate local and systemic control seem more responsible for prognosis than the metastatic situation itself.

**P053**

**The use of a textile implant as artificial ligament to prevent dislocation in THA due to pelvic resections**

P.T. Funovics, M. Dominkus, C. Toma, F. Abdolvahab, R. Kotz
*(Department of Orthopaedics, Vienna Medical University, Vienna, Austria)*

*Purpose:* The risk of dislocation of THA after resection of pelvic tumours is fairly high due to resulting soft tissue loss. We report a procedure using a textile implant (LARS®) to prevent dislocation of pedestrial cup implants.

*Patients/methods:* In 7 consecutive patients (1 male; 6 female; average age 51 years) with skeletal malignancies (osteosarcoma: 2; chondrosarcoma: 1; metastasis: 4;) in whom resection of the acetabular pelvic bone was indicated, reconstruction of the hip was performed by use of a pedestrial cup (Schöllner®) and a rectangular femoral stem (Zweymüller®). Either primary (in 6) or secondary (in 1) to this a textile LARS®-ligament was attached in a sleeve-wise technique around the prosthetic femoral neck and periacetabular tissue as a recontruction of joint ligaments. Post-operative cast immobilization of the hip was performed for 6 to 12 weeks.

*Results:* Patients could be mobilized partially weight-bearing starting at the fifth post-operative day, and remained so according to subjective ability. There were no cases of dislocation and ability to walk was rated satisfying by all patients. At latest follow-up 5 to 14 months post-operatively (average: 8.3 months) range of motion averaged 84° in flexion (60° to 120°).

*Conclusion:* The use of a textile implant in THA after pelvic resections has shown to be a reliable instrument to prevent dislocation due to large soft tissue defects with satisfying functional results.

**P054**

**A pilot trial of tumour-lysate loaded dendritic cell vaccines in patients with primary and secondary skeletal malignancies**

P.-T. Funovics[1], M. Dominkus[1], R. Kotz[1], T. Felzmann[2]
*([1]Department of Orthopaedics, Vienna Medical University, Austria, [2]Childrens' Cancer Research Institute, Vienna, Austria)*

*Purpose:* This study presents preliminary data of tumour-lysate loaded dendritic cell-vaccination (DC) for recurrent or metastatic skeletal malignancies.
*Patients/methods:* In 7 patients suffering osteosarcoma (1), chondrosarcoma (1), Ewing's Sarcoma (2), soft tissue sarcoma (1) and metastasis of renal cell carcinoma (2), DC-vaccination was administered additional to standard treatment. DC precursor cells were generated from peripheral blood mononuclear cells by leukocyte apheresis and charged with lysed autologuous tumour cells obtained by surgery. Each patient received 6 vaccinations of $1 \times 106$ cells (=0.5 ml) intranodally under sonographic guidance in weekly intervals. Delayed Type Hypersensitivity (DTH) testing for immune response against tumour antigens and standard clinical and radiological examinations were performed before and after treatment.
*Results:* No adverse side effects were observed in any patient throughout treatment. Three patients died of disease, 2 patients showed progressive disease, 2 revealed stable disease for 15 and 21 weeks. Helper as well as cytotoxic T-lymphocytes of 3 patients showed in vitro reactivity in terms of CD4 expression.
*Conclusion:* The absence of adverse effects and uncomplicated therapeutic regimen together with promising immunological responses, that can be correlated to clinical outcome suggest that the use of DC-vaccination should be investigated in earlier stages of disease to improve results.

**P055**

**Clinical characteristics of aggressive fibromatosis in children**

J. Godzinski[1], K. Bronowicki[2], C.C. Flohil[3], F.W.J. ten Kate[2], D.C. Aronson[2], H.A. Heij[2], E. Bien[1], W. Madziara[1], D. Perek[1], A. Prokurat[1], M. Rapala[1], W. Sulka[1], W. Wozniak[1], M. Wysocki[1]
*([1]Polish Paediatric Solid Tumours Study Group, [2]Emma Children's Hospital AMC, [3]Vrije Universiteit Medical Center, Amsterdam, The Netherlands)*

*Background:* Aggressive fibromatosis (AF) has different characteristics in childhood and in adulthood. Its adequate management is unclear.
*Aim:* To analyse AF behaviour and outcome after various methods of treatment.
*Patients:* 45 patients (9 centres in The Netherlands and Poland: 1988–2004). 29 boys (64%), 16 girls; age 0–17 years; locations: limbs/26 (58%), trunk & abdominal wall/11 (24%), head & neck/8 (18%).
*Results:* 40/45 (89%) underwent surgery. 22 resections (55%) were incomplete/marginal (15 relapsed 68%), 18 wide/compartmental and complete (2 relapsed 11%). 24 underwent surgery only (10 relapsed 42%). Surgery was supplemented with CHT in 10 (5 relapsed), RTX in 3 (no relapse), RTX + CHT in 3 (2 relapsed). RTX & CHT only were used in 1 (relapsed), CHT in 4 (2 CR, 2 progression-free).
*Summary:* In contrast to adults, AF of childhood is located predominantly in the limbs and has a higher incidence in boys. Wide/compartmental complete resection (16/18 CR 89%) has good predictive value for outcome; marginal/incomplete resections imply a high risk of relapse (7/22 CR 32%). RTX seems of value (3/3 CR); good responders to CHT occur (4/4 progression-free). 40% relapse-rate indicates that the problem requires broader studies.

**P056**

**Extraskeletal osteosarcoma treated like conventional osteosarcoma**

S.Y. Goldstein-Jackson[1,2], G. Gosheger[3], G. Delling[4], W.E. Berdel[5], G.U. Exner[6], G. Jundt[7], J.N. Machatschek[1], A. Zoubek[8], H. Jürgens[1], S. Bielack[1]
*([1]Universitätsklinikum Münster, Pädiatrische Hämatologie und Onkologie, Münster, Germany, [2]University of Wales College of Medicine, Heath Park, Cardiff, Wales UK, [3]Universitätsklinikum Münster, Allgemeine Orthopädie, Münster, Germany, [4]Universitätsklinikum Hamburg-Eppendorf,, Institut für Osteopathologie, Hamburg, Germany, [5]Universitätsklinikum Münster, Medizinische Klinik und Poliklinik A, Germany, [6]Orthopädische Universitätsklinik Balgrist, Zürich, Switzerland, [7]Universitätsspital Basel, Knochentumor-Referenzzentrum am Institut für Pathologie; Switzerland, [8]St. Anna Kinderspital, Vienna, Austria)*

*Purpose:* The aims of this analysis were to investigate the clinical features of extraskeletal osteosarcoma (ESOS) and examine the outcome after multimodal therapy.
*Methods:* The Cooperative Osteosarcoma Study Group database was searched for patients with extraskeletal osteosarcoma. Eligible patients were included in a retrospective analysis of patient, tumour, and treatment related variables and outcome. Scheduled treatment included surgery and multi-agent chemotherapy as for conventional osteosarcoma.
*Results:* 17 eligible patients identified, median age 44 years (3–65). The thigh was the commonest tumour site. Two patients had a history of previous malignancies and two had primary metastases. Median follow up was 3.2 years (range: 0.6–7.4 years) and at last follow up, eleven patients were alive in complete remission, three patients were alive with disease and three patients had died of disease. Actuarial 3-year overall and event-free survival rates were 77% and 56%, respectively. Patients with macroscopically complete surgical remission had an improved overall survival ($p = 0.0004$).
*Conclusions:* The patients in this retrospective study had a surprisingly good survival rate. This may be due to the combination of multi-agent chemotherapy with surgery, and we recommend this approach in the treatment of ESOS.

**P057**

**Late effects of treatment in rhabdomyosarcoma (RMS)**

Y. Goshen, I.J. Cohen, S. Ash, J. Stein, R. Zaizov, I. Yaniv
*(Department of Pediatric Hematology/Oncology, Schneider Children's Medical Center of Israel, Petah Tikva, Sackler Faculty of Medicine, Tel Aviv University, Israel)*

89 RMS patients treated between 1974–2003. 66 are survivors. Median follow-up 163 months (14–372). Treatment included surgery, chemotherapy (VAC, VACA, VACAIE, IRS III, V) and radiotherapy (30–60 Gy). Five survivors died, 4 of second cancer, 1 committed suicide. Radiotherapy related: 9/10 orbital tumors received x-rays, 4 developed hypoplastic orbit and severe retinopathy, 7 ptosis, 7 cataract, and 6 keratoconjuctivitis sicca. In parameningeal RMS 8/11 developed face disfiguration, 4 hypoplastic jaws and teeth, 2 hypoplastic orbit with retinopathy, 2 hypoplastic pharynx/larynx with speech/eating disorders and 2 GH deficiency. Recurrent hemorrhagic cystitis occurred in 2/5 bladder/prostate tumors. 7–13cm leg shortening was observed in 3/10 extremities tumors irradiated at age <5 years. Chemotherapy related. Cardiac: 48 survivors received doxorubicin, 21 received 350–450 mg/m$^2$, six developed cardiomyopathy, 4 with CHF. None at doses < 350 mg/m$^2$. Fertility: 14/24 males > 21 years examined were azoospermic, (cyclophosphamide doses were 10.2–24 gr/m$^2$, some received ifosfamide), 8 were pre-pubertal at treatment. 4/13 with paratesticular RMS, developed ejaculation disorder (surgery related). 4/15 females, developed ovarian failure, 3 after irradiation. Second tumors developed in 6, two AML and one T-ALL (14, 46, 30 months from RMS). One who developed osteosarcoma 7 years post RMS, belonged to a cancer family (L-F), BCC in one and thyroid adenomas in another.
*Conclusions:* 1. RMS survivors require prolonged follow-up. 2. New protocols must relate to previous late effects.

### P058

**Status of surgical margins and prognosis in adult soft tissue sarcomas of the extremities: A series of 911 consecutive patients treated at a single institution**

A. Gronchi[1], M. Fiore[1], P.G. Casali[2], L. Mariani[3], R. Miceli[3], P. Collini[4], L. Lozza[5] P. Olmi[5]
*([1]Department of Surgery, Istituto Nazionale per lo studio e la cura dei Tumori, Milano, Italy, [2]Department of Cancer Medicine, Istituto Nazionale per lo studio e la cura dei Tumori, Milano, Italy, [3]Department of Biostatistics, Istituto Nazionale per lo studio e la cura dei Tumori, Milano, Italy, [4]Department of Pathology, Istituto Nazionale per lo studio e la cura dei Tumori, Milano, Italy, [5]Department of Diagnostic Imaging and Radiotherapy, Istituto Nazionale per lo studio e la cura dei Tumori, Milano, Italy)*

*Purpose:* To explore the prognostic effect of microscopic marginal status after surgery for extremity soft tissue sarcomas.
*Patients/methods:* 911 consecutive patients surgically treated over a 20-year span at a single referral center were analyzed. Six hundred and forty-two were first seen with a primary, and 269 with a locally recurrent tumor. All patients underwent macroscopically complete resection. Microscopic marginal status was negative (tumor >1 mm) in 748 patients and positive (≤1 mm) in 163. Median follow-up was 107 months.
*Results:* Patients with primary disease had a lower disease-specific mortality in comparison to those first viewed for recurrence (25% vs. 37% at 10 years). Size, malignancy grade, depth, histotype and local recurrence had a statistically significant prognostic effect at multivariable analysis, while microscopically positive surgical margins had not, though a trend in favour of negative margins was observed. However, an extra risk was observed for patients with positive margins after 3–5 years (HR = 1.8 after 5 years versus 0.8 before 5 years). In patients treated for a local recurrence, the prognostic impact of positive margins was higher (HR = 1.6).
*Conclusion:* Positive surgical margins had a weak adverse prognostic effect, which was more pronounced for those patients escaping an early relapse. This would seem to justify a policy of surgical adequacy in adult soft tissue sarcomas, though clinical decision-making in borderline presentations for conservative surgery might be reasonably flexible and shared with the patient. Once a local relapse has occurred, the impact of local treatments seems more critical.

### P059

**Targeting of pediatric embryonal tumors**

K. Hajdin, H. Witt, F. Niggli, B.W. Schäfer, M. Bernasconi
*(University Children's Hospital Zurich, Division of Oncology, Zurich, Switzerland)*

Rhabdomyosarcoma (RMS) is a highly malignant tumor in childhood. Existing chemotherapies can be improved by delivering drugs specifically to the tumor or its vasculature. For identification of a specific surface target on RMS tumor cells we used in vitro phage display with libraries coding for cyclic random peptides. A 80-fold enrichment was observed after five rounds of panning as compared to the nonrecombinant phage. Sequencing of the enriched phage population identified phages displaying the peptides CQQSNRGDRKRC and CMGNKRSAKRPC. Phages carrying these sequences were tested on RMS, neuroblastoma cells and fibroblasts. Both peptides showed an over 400 fold increase in binding to RD cells and bound with lower affinity to normal fibroblasts.

The RGD containing peptide confirmed the efficacy of our screening: This motif mediates the interaction between fibronectin and integrins αvβ5 and αvβ6, which are up-regulated in tumor cells. The second peptide contains a motif that was previously identified in an in vivo screening for breast tumor targeting peptides and showed specificity for tumor lymphatics. These results demonstrate that surface markers on tumor cells are shared between different tumor types. Furthermore, specific targets for RMS can be identified, examined and used to improve targeting and efficiency of small anti-tumor molecules.

### P060

**The linear body growth in height and body weight in children with sarcoma during the two years after initiating oncological therapy**

T.F. Hajnzic, G. Stipancic, M. Cuk, M. Mataija
*(Department of Pediatrics, Hematology and Oncology Unit, University Hospital Sestre Milosrdnice, Zagreb, Croatia)*

We wanted to estimate the incidence of delay in linear body growth and body growth velocity in children with sarcoma during initial, aggressive therapy. We evaluated the effects of therapy on body height (BH) and body weight (BW) in sarcoma-patients during the first six months of therapy and eighteen months after initiating therapy.

3–6 month after initiating oncological therapy (protocol CWS-96 P-Treuner, Koscielniak 1995), we found a decrease of growth velocity and stagnation of BH in 79% (15/19) pts. 2/3 (9/15) pts with total growth failure had significant catch up and equalize linear BH. The some quantitative changes were found in the pace of BW changes. After discontinuation of therapy 90% of pts (17/19) had no further loss in BW.

The problem of late sequeles of cancer and chemotherapy during childhood is increasing. Acute toxic effects of therapy are mostly expressed during aggressive multi-agent induction of chemotherapy and radiotherapy. Our results show that large decrease in linear BH and BW during first months of treatment in most cases is temporary. It is less expressed during less toxic maintenance therapy and it disappears after discontinuation of the therapy.

### P061

### 3480 Cases of Dermatofibrosarcoma Protruberans

P.J. Chuba[2], M.R. Hamre[1], M. Mott[1], R. Severson[1], D. Lucas[3]
*([1]Department of Radiation Oncology, St. John Health Systems, Detroit, MI, [2]Departments of Pediatrics and Orthopedic Surgery, Wayne State University, Detroit, MI, [3]Department of Pathology, University of Michigan, Ann Arbor, MI)*

*Purpose:* Dermatofibrosarcoma protruberans (DMFP) has low metastatic potential. We reviewed cases accessioned by the NCI SEER program 1973–1999.
*Patients/methods:* 3480 cases of DMFP were identified with median follow-up of 7.25 years. Tumors were head and neck ($n = 532$; 15.3%), upper extremity ($n = 791$; 22.7%), lower extremity ($n = 654$; 18.8%), and truncal ($n = 1457$; 41.9%).
*Results/discussion:* Overall survival for localized DMFP was 95%. Head and neck tumors were more common in males, Caucasians, and the elderly. Distant metastasis was rare ($n = 15$; 0.5%) with 5 year survival of 56.4%. Tumor size ($p = 0.007$), and tumor site ($p = 0.0009$) were both statistically significantly associated with overall survival. Head and neck tumors showed 91.4% five year overall survival compared with 96.2%, 96.7%, and 95.1% of cases affecting the upper extremity, lower extremity, and trunk, respectively.
*Conclusion:* These data provide an epidemiologic basis for the conservative management of DMFP with wide local excision. Head and neck and larger tumors may have decreased overall survival.

### P062

### Late relapses in patients with osteosarcoma: Study of clinico-pathological parameters

E.I. Hauben[1,8], S. Bielack[2], R. Grimer[3], G. Jundt[4], P. Reichardt[5], M. Sydes[6], A. H. M. Taminiau[7], P.C.W. Hogendoorn[1]
*([1]Department of Pathology, Leiden University Medical Center, Leiden, The Netherlands, [2]University Chidren's Hospital, Department of Pediatric Hematology and Oncology, Munster, Germany, [3]Royal Orthopaedic Hospital, Birmingham, United Kingdom, [4]Institute of Pathology, Bone Tumor Reference Center, Basel, Switzerland, [5]Charite Campus Berlin-Buch, Robert-Rössle Klinik, Berlin, Germany, [6]MRC Clinical Trials Unit, 222 Euston Road, London NW1 2DA, UK, [7]Department of Orthopaedic Surgery, Leiden University Medical Center, Leiden, The Netherlands, [8]Laboratory for Pathology, Stichting Pamm, Eindhoven, Netherlands)*

*Purpose:* To identify characteristic clinico-pathological features of osteosarcoma patients with late relapse.
*Materials/methods:* Late relapse was defined as first local recurrence or metastasis 5 years or more after diagnosis. Patient data on age, gender, location of the tumour and histological subtype were retrieved from 557 patients from the European Osteosarcoma Intergroup, 1136 patients from the Cooperative OsteoSarcoma Study and 550 patients of one of the authors (RG). Patients without relapse after 5 years acted as a control.
*Results:* Thirty-three patients had late relapse. Age and sex distribution did not differ from the control. Eighteen (54%) patients had their osteosarcoma located in the tibia or the fibula versus 170 patients (31%) in the control group. Thirteen (39%) patients had an osteosarcoma subtype other than conventional versus 158 (29%) in the control group. This was largely due to the higher incidence of chondroblastic osteosarcoma subtype in the late relapses patient group.
*Conclusion:* The occurrence of late relapse does not appear to be associated with age or gender. Patients with a chondroblastic subtype of osteosarcoma or a location of osteosarcoma in the tibia or fibula are at higher risk to have a late relapse.

### P063

### High prevalence of Simian virus 40 in human osteosarcoma of hungarian origin

S. Heinsohn[1], M. Szendroi[2], U. zurStadt[1], H. Kabisch[1]
*([1]Centre for Women's and Children's Health, Clinic and Policlinic for Peadiatric Hematology and Oncology University Hospital, Hamburg, Germany, [2]Department of Orthopedics, Faculty of Medicine, Semmelweis University Budapest, Hungary)*

Recent studies have detected Simian virus 40 (SV40) DNA in specific human tumors albeit with significant discrepancies in frequency.

The evidence that SV40 might play a role in some malignancies is mounting. The major SV40 oncoprotein is the large tumor antigen (L-Tag) due to its transforming activity and the capability to bind and to inactivate p53 and pRB or exerting an oncogenic potential by another mechanism. We analysed 130 paraffin embedded osteosarcoma (OS) from Hungary by a recently established real time quantitative (RQ-) PCR assay and 42 OS from Germany by different methods. Peripheral blood samples from healthy people of both countries served as a control group.

The RQ-PCR assay allowed the linear amplification of 101 to 105 copies in 500 ng of genomic DNA. More than 102 SV40 copies were detectable in 75% of the Hungarian OS compared to less than 5% of the German OS samples as shown in our recently published data.

We found a weak SV40 positivity (less than 101) in 32/166 (19%) and 2/149 (1.3%) peripheral blood samples from Hungarian or German healthy volunteers, respectively.

Our data demonstrate remarkable differences in the incidence of SV40 sequences in OS of different geographic origin with an overall low frequency in samples from peripheral blood of healthy volunteers.

### P064

### Is proliferative activity a useful diagnostic means for low-grade chondrosarcoma?

A. Helfenstein[1], S.Frahm[2], M. Krams[2], W. Drescher[1], R. Parwaresch[2], J. Hassenpflug[1]
*([1]Department of Orthopedic Surgery, University of Kiel, Kiel, Germany, [2]Department of Pathology and Lymph Node Registry, University of Kiel, Kiel, Germany)*

Distinguishing enchondromas from highly differentiated chondrosarcomas is a common problem in bone pathology. Even though proliferation activity is known to be a prognostic factor in chondrosarcomas, it is not a major grading criterion as in other tumors. One reason may be the large number of grade 1 tumors and their extremely low proliferation rates. Proliferating cells are too rare and often cannot be detected by the established markers.

The present study focused on a new and very sensible marker of proliferation, a monoclonal antibody to the nuclear minichromosome maintenance protein 6, named Ki-MCM6. We studied 17 chondrosarcomas (12 grade 1) and compared them with 14 enchondromas immunohistochemically by using antibodies to MCM6, Ki-67 and Repp86. Ki-MCM6 proved most effective at identifying proliferative activity in grade 1 chondrosarcomas. The MCM6 labeling index correlated with tumor grade and was significantly increased in grade 1 chondrosarcomas compared with enchondromas. The 5 cases of progressive chondrosarcoma also had a significantly higher MCM6 labeling index than the nonprogressive cases. Furthermore, by means of the MCM6 labeling index, many cases of progressive disease were recognized among those of uncertain malignant potential, justifying their classification as low-grade chondrosarcomas.

**P065**

**Topotecan and cyclophosphamide for Ewing tumour relapse**

A. Hunold[1], N. Boldering[1], C. Liebscher[2], M. Paulussen[1], H. Jürgens[1]
*([1]University Children's Hospital, Department of Paediatric. Haematology and Oncology, Münster, Germany, [2]Coordinating Centre for Clinical Trials (KKS), University of Münster, Germany)*

*Background:* Every third Ewing tumour patient relapses. Prognosis in relapse is poor. Standard chemotherapy regimens for this situation are lacking.
*Patients:* Fifty-seven patients aged 3.2–49.5 (median 16.9) years were treated with the combination of topotecan (0.75 mg/m$^2$/day, days 1–5) and cyclophosphamide (250 mg/m$^2$/day, days 1–5) following first (42) or second (7) relapse or progression under first-line therapy (8).
*Results:* A median of 3 (range 1–14) topotecan/cyclophosphamide courses were given. Twenty-three patients (40.4%) showed complete (6) or partial (17) response, 13/57 patients (22.8%) had stable disease, 15/57 (26.3%) progressed. Two (3.5%) patients showed a mixed response. At completion of relapse therapy, 26/57 patients were in complete (20) or partial (6) remission. One had stable disease, 26 showed progression, information was unavailable in 4 patients. Of the 19 relapse patients achieving CR, 11 maintained remission (57.9%). At the time of evaluation, after a median follow-up for survivors of 23.1 (range 7.8–59.8) months from the event prompting topotecan/cyclophosphamide treatment, 14/57 patients (24.6%) were in continous complete (13) or partial (1) remission. Overall survival after 1 year was 59% (95% CI 48.1–73.7%), after 2 years was 31.7% (95% CI 18.1–45.3%).
*Conclusion:* Topotecan/cyclophosphamide is active in relapsed Ewing tumours and warrants further evaluation.

**P066**

**Vacuum-assisted biopsy of soft tissue tumors: A minimal-invasive alternative to incision biopsy**

J. Jakob[1], M. Hünerbein[1], S. Gretschel[1], P.-U. Tunn[1], W. Schneider[2], P.M. Schlag[1]
*([1]Clinic for Surgery and Surgical Oncology, Charité-Campus Buch, Berlin, Germany, [2]Institut for Pathology, Helios-Klinikum Berlin-Buch, Berlin, Germany)*

*Purpose:* To assess feasibility and accuracy of vacuum-assisted biopsy of soft tissue tumors and to compare the results with surgical biopsies.
*Patients and methods:* Vacuum-assisted biopsy (8G) was performed in 39 Patients with soft tissue tumors in local anaesthesia between April 2004 and January 2005. As a control group we reviewed all patients who underwent incision biopsy for soft tissue tumors in general anaesthesia between 2003 and 2004 ($n = 42$). Tumor localisation, size and histology were similar in both groups.
*Results:* Vacuum-assisted biopsy was technically sucessful in all patients and provided high quality specimen. All specimen were adequate to make a diagnosis including malignancy, tumor subtype and grade. Sensitivity and specificity for malignancy were 100% and accuracy of diagnosis was 96.7%. Incision biopsy provided adequate specimen in all cases and sensitivity and specificity for malignancy also were 100%. Accurate histologic diagnosis was obtained in 97.5%. No relevant complications occurred related to either biopsy. Regarding the effectiveness of both techniques there were no differences.
*Conclusion:* Vacuum-assisted biopsy provides accurate specimens for the diagnosis of soft tissue tumors and may be considered as minimal-invasive alternative to incisional biopsy.

**P067**

**Osteosarcoma in children in Slovakia 1995–2004: Diagnostics, treatment and results**

I. Jenčo[1], V. Žilinek[2], P. Bician[3], I. Oravkinová[1], E. Bubánska[3], E. Kaiserová[2]
*([1]Department of Children's Oncology, Kosice, Slovakia, [2]Clinic of Children's Oncology Bratislava, Slovakia, [3]Department of Children's Oncology, Banska Bystrica, Slovakia)*

The authors present a review of 66 patients with the diagnosis of osteosarcoma at the age of up to 18 years, treated in the Slovak Republic in three children's oncological centres (Bratislava, Banská Bystrica and Košice). They analyse the epidemiology and incidence of osteosarcoma in this age group, they show the summary of used chemotherapeutic regimes and the ways of local treatment, showing EFS and OS results of the mentioned group of patients.

**P068**

**Reconstruction of a foot by free musculocutaneus Latissimus dorsi flap and free tendon transfer after resection of a large congenital fibro sarcoma in a 15-week-old infant. A case report**

A. Jester[1], K.-L. Waag[2], B. Selle[3], G. Germann[1]
*([1]Klinik für Rekonstruktive, Plastische- und Handchirurgie, -Schwerbrandverletztenzentrum, BG-Unfallklinik Ludwigshafen, Plastische und Handchirurgie der Universität Heidelberg, Germany, [2]Klinik für Kinderchirurgie, Klinikum der Stadt Mannheim GmbH, Mannheim, Germany, [3]Klinik für Kinder und Jugendmedizin, St. Annastiftskrankenhaus Ludwigshafen, Germany)*

*Purpose:* To present the case of a 15-week-old infant after microsurgical reconstruction of a large defect of the lower leg due to fibro sarcoma.
*Patient/method:* After birth a 5.5 × 4 cm large tumor was observed on the dorsum of the right foot. Biopsy showed a congenital fibro sarcoma. After four cycles of chemotherapy a radical excision of the tumor at the age of 14 weeks followed. The defect was temporarily covered. Histology showed a complete excision of the tumor. To cover the defect a musculocutaneus latissimus dorsi flap was taken, the cutaneus part being large enough to cover the defect. Extensor tendons were reconstructed with free tendon transplants.
*Discussion:* Being confronted with a soft tissue tumor in extremities, oncologists and surgeons have to decide, what kind of strategy to follow. Radical excision usually involves amputation or mutilation, or on the other hand, with the aim of preserving extremities compromises oncological principles of surgical radicality. Microsurgical techniques are rarely applied to infants. This case shows that reconstruction is rewarding. Plastic surgery adds an important factor to pediatric oncology and surgery and should be routinely integrated in oncological concepts.

**P069**

**Cell surface charge modification by lipopolysaccharides from rhodobacter capsulatus**

D. Kabanov, S. Prokhorenko, I. Prokhorenko
*(Molecular Biomedicine Laboratory, Institute of Basic Biological Problems RAS, Pushchino, Russia)*

The antineoplastic activities of bacterial lipopolysaccharides (LPS) have been severely limited by the systemic toxicity. LPS from Rhodobacter capsulatus (LPSRb.cap) is nontoxic. The LPS intercalation into a cell membrane via lipid A part is capable to disturb tumor homeostasis, leading to tumor accessibility of immunological defence mechanisms. Since malignant cells carry a higher density of negatively fixed charges on their surfaces we have chosen human erythrocytes (HE) as a model for our investigation. We compare the influence of LPSRb.cap and LPS from Escherichia coli O55:B5 (S-LPSE.coli) intercalation into the erythrocyte membrane on HE electrophoretic mobility (HEEM) at the saturation thereshold. HE ($5 \times 106$/ml) were incubated with corresponding LPS in PBS solution (pH 7.4) at 37°C for 1 h. The HEEM was measured with a "Parmoquant-2" device. The incubation with LPSRb.cap and LPSE.coli (35 μg/ml) decreased the HEEM by 5% ($-1.23 \pm 0.01$; $p < 0.001$) and ($-1.22 \pm 0.01$; $p < 0.001$) respectively, as compared to the control ($-1.29 \pm 0.01$). The HEEM decreased by 11% ($-1.15 \pm 0.02$; $p < 0.001$) at the LPSE.coli concentration of 70 μg/ml, whereas the same LPSRb.cap concentration decreased HEEM only by 5%. These results indicate that the effect of LPS on the HEEM depends on the lipid A intercalation capacity and the total charge of the LPS molecule. We suppose to test LPSRb.cap for tumoricidal activity in tumor models in vitro and in vivo.

**P070**

**In vivo proton magnetic resonance spectroscopic (PMRS) evaluation and its histopathological correlation for characterisation of soft tissue sarcomas**

Kar-Madhabananda[1], S.V.S. Deo[1], N.K. Shukla[1], N.R. Jagannathan[2], U. Sharma[2], V. Kumar[2], S. Datta Gupta[3], Dr. V. Deo[3], S. Thulkar[4]
*([1]Department of Surgical Oncology, [2]IRCH, N.M.R, [3]Department of Pathology, [4]Department of Radiology, All India Institute of Medical Sciences, New Delhi, India)*

*Purpose:* To correlate in vivo proton MRS findings with histopathological features of various soft tissue sarcomas.
*Patients/methods:* PMRS Study was performed at 1.5T with a surface coil appropriate for the location of the lesions in 16 patients. Single-voxel (SVS) study has been done in 10 cases and chemical shift imaging (CSI) study characterised the heterogeneity of the tumor in 6 cases by using point-resolved spectroscopic sequence (PRESS) with echo time TR = 2000/TE = 30 & 270 msec. The choline peak was identified at 3.2 ppm in spectra. MRS results and histopathologic findings were correlated and $P < 0.001$, considered being significant.
*Results/discussion:* Choline peak was found in 11 out of 11 patients with malignant soft tissue tumours where as one patient with benign and four treated malignant soft tissue tumour patients with no residual disease did not show any choline. In vivo spectroscopy here shows sensitivity, specificity, ($p < 0.001$), positive predictive value, negative predictive value and accuracy of 100% each. In vivo PMRS study can differentiate the malignant from benign soft tissue tumor, tumour heterogeneity (especially CSI) and tumour activity in recurrent and/or residual soft tissue sarcoma.

**P071**

**Genitourinary soft tissue sarcomas located outside bladder and prostate in children treated according to the CWS-96 Protocol: Report from the Polish Paediatric Solid Tumours Study Group**

E. Bien, T. Stachowicz-Stencel, B Kazanowska, A. Balcerska, W. Balwierz, A. Chybicka, A. Dluzniewska, E. Drozynska, A. Kurylak, M. Matysiak, M. Krawczuk-Rybak, M. Rychlowska, E. Solarz, B. Sopylo, D. Stencel, J. Wachowiak, M. Wieczorek, W. Wozniak, M. Wysocki
*(Oncological Centres of the Polish Paediatric Solid Tumours Study Group: Gdansk, Wroclaw, Krakow, Bydgoszcz, Warszawa, Bialystok, Poznan, Chorzow)*

*Aim:* Analysis of therapy efficacy in non-bladder/prostate genitourinary sarcomas in children treated from I'1997 to VI'2003 with CWS-96 protocol in Poland.
*Material and methods*: 19 children (M/F: 15/4, age: 3mo–17.5y; median 7.2y). Histopathology: RMS – 15 pts (RME – 13), non-RMS – 4.
*Results:* Primary site: Testes – 9 pts, paratesticular region – 6, uterus – 2, vagina and ovary – 1 each. 63% presented with low stage neoplasm (I – 7, II – 5). Primary tumour exceeded 5 cm and/or invaded surrounding tissues in 7 pts (37%). 3 pts had regional, 2 pts – distant lymph nodes metastases. Primary excision: complete in 7 pts, incomplete – 12 (microscopically – 5, macroscopically – 7). 10 pts received chemotherapy for high risk group, 5 – standard, 4 – low. 6/7 pts with macroscopic tumour residues responded to chemotherapy (CR – 4, GR – 2). 1 pt (stage III triton tumour of uterus) did not, entering CR after mutilating delayed surgery. No other patient required delayed tumour resection. Radiotherapy (23.5–54 Gy) was given to 8 pts. 3 pts developed local relapse (non-RMS histology and incomplete primary excision in 2). 3 pts died (16%): 2 due to neoplasm progression, 1 of neutropenia-related sepsis. 16 pts are alive (84%) with mean follow-up 48months. The only permanent complications result from surgery (uterus and bladder removal – 1 NR, bladder resection after relapse – 1). Chemotherapy and radiotherapy were accompanied by severe but transient myelosuppression in HR group.

**P072**

**Soft tissue sarcomas of bladder and prostate in children treated according to the CWS-96 protocol: Report from the Polish Paediatric Solid Tumours Study Group**

T. Stachowicz-Stencel, E. Bien, B. Kazanowska, A. Balcerska, W. Balwierz, A. Chybicka, A. Dluzniewska, E. Drozynska, K. Katski, J. Kowalczyk, A. Kurylak, J. Peregud-Pogorzelski, D. Stencel, B. Zalewska-Szewczyk, J. Wachowiak, M. Wysocki
*(Oncological Centres of the Polish Paediatric Solid Tumours Study Group from Gdansk, Wroclaw, Krakow, Lublin, Bydgoszcz, Szczecin, Poznan, Lodz, Poland)*

*Aim:* Analysis of course, outcome and therapy complications in bladder/prostate soft tissue sarcomas in children treated from I'1997 to II'2003 according to CWS-96 protocol in Poland.

*Material/methods:* 22 children (M/F: 17/5, age: 8mo–17y2mo; median 5.3y). Histopathology: RMS–20 pts (RME–14), non-RMS – 2 pts. Results: 96% presented with advanced neoplasm (III – 14, IV – 7). In 18pts (82%) primary tumour exceeded 5 cm; in 17 (77%) extended beyond site of origin. Lymph nodes metastases were stated in 6 (27%), distant metastases in 7 pts (32%). No patient underwent primary complete tumour excision. All received chemotherapy for high risk group. Response to chemotherapy after 3 cycles: CR – 2 pts, GR – 6, PR – 3, OR – 2, PD – 4, missing data – 5. Delayed resections were performed in 11 pts (mutilating – 7) proving complete in only 5. Radiotherapy (32–50.4 Gy) was given to 16 pts, mainly after delayed incomplete surgery. 5 pts developed local relapse, 4 – continual PD. None of these 9 pts had ever complete tumour resection. 9 pts died: 7 due to neoplasm progression, 2 of therapy complications (septic shock, ARDS). 13 pts are alive (59%) with mean follow-up of 42 months. 8 had bladder removed with continent ileal pouch formed in 4. Chemotherapy and radiotherapy were accompanied by severe but transient myelosuppression and infections.

**P073**

**Young patients with metastatic soft tissue sarcomas: Retrospective multicenter study of the polish pediatric solid tumors group**

B. Kazanowska[1], A. Reich[2], A. Balcerska[3], W. Balwierz[4], J. Bodalski[5], A. Dłużniewska[4], E. Drożyńska[3], K. Kątski[6], J. Kowalczyk[6], A. Kurylak[7], M. Matysiak[8], J. Peregoud-Pogorzelski[12], M. Rychłowska[9], B. Sopyło[8], D. Stencel[10], B. Szewczyk[5], J. Wachowiak[10], M. Wieczorek[11], W. Woźniak[9], M. Wysocki[7], A. Chybicka[1]
*([1]Department of Pediatric Hematology, Oncology and Bone Marrow Transplantation, Wroclaw Medical University, Poland, [2]Department of Dermatology, Venereology and Allergology, University of Medicine, Wroclaw, Poland, [3]Department of Pediatrics, Hematology, Oncology and Ensocrinology, Medical University of Gdańsk, Poland, [4]Department of Pediatric Oncology and Hematology, Polish-American Institute of Pediatrics, Jagiellonian University Medical College, Kraków, Poland, [5]Oncohematology Unit, Institute of Pediatrics, Medical University of Łódź, Poland, [6]Department of Pediatric Hematology and Oncology, Medical University, Lublin, Poland, [7]Department of Pediatric Hematology and Oncology, The Ludwik Rydygier Medical University, Bydgoszcz, Poland, [8]Department of Pediatrics, Hematology and Oncology, Medical University of Warsaw, Poland, [9]Department of Oncological Surgery in Children and Youth, Institute of Mother and Child, Warszawa, Poland, [10]Department of Oncology, Hematology and Pediatric Transplantology, University of Medical Sciences, Poznań, Poland, [11]Department of Pediatric Oncology, Chorzów, Poland, [12]Department of Pediatric Oncology, University of Medicine, Szczecin, Poland)*

*Objectives:* The aim of this study was to present the 10-years experiences of the Polish Paediatric Solid Tumors Group in the treatment of children suffering from metastatic soft tissue sarcomas.
*Material and method:* All included patients were treated between 1991 and 2002 in 11 centres belonging to the PPSTG. The children were treated according to SIOP-MMT-91 protocol (23 patients), and according to CWS-96 protocol (35 patients). Their age ranged between 1 and 217 months. In 4 patients undifferentiated rhabdomyosarcoma, in 23 embryonal rhabdomyosarcoma, in 18 alveolar rhabdomyosarcoma, in 9 PNET, in 1 EES, and in 3 SS was diagnosed.
*Results:* Estimated 5-years event free survival for all patients was 0.26 and estimated 5-years overall survival was 0.33. Complete remission achieved 37 patients (63.8%). The comparison of EFS between the patients treated with different protocols revealed significantly better results of therapy in the group treated according CWS-96 protocol ($p < 0.005$). The diagnosis, patient's age, tumour's localisation and number of metastases significantly influenced the prognosis when considered together ($p = 0.03$).
*Conclusions:* A good stratification system for disseminated soft tissue sarcomas is still lacking. Patients with very poor prognosis need a new therapy strategies as a currently employed regimens are totally uneffective.

**P074**

**Second recurrence of osteosarcoma: An analysis of 249 COSS patients**

B. Kempf-Bielack[1], S. Bielack[1], D. Branscheid[4], Th. Kühne[2], A. Zoubek[3], H. Jürgens[1]
*([1]Departments of Pediatric Hematology and Oncology, University Children's Hospital, Muenster, Germany, [2]University Hospital Basel, Switzerland, [3]St. Anna-Kinderspital, Vienna, Austria, [4]Thoraxklinik Grosshansdorf, Germany)*

*Background:* Information about the presentation and outcome of second and subsequent osteosarcoma recurrences is limited to case reports and small series.
*Patients:* 249 consecutive patients with second osteosarcoma recurrences from the COSS trials were evaluated for history, presentation, and outcome.
*Results:* Second recurrences occurred a median of 2.7/0.8 (min-max 0.5/0.1–15.9/9.8) years after the initial biopsy and first relapse, respectively. After a median of 1.0 (0–16.8) years from the diagnosis of second recurrence for all patients and 2.2 (0–16.8) years for 52 survivors, 5 and 10 year actuarial survival rates were 19%/12%. The corresponding rates were 32%/24% for 119 patients with a third surgical CR and 2%/0% for the others ($p < 0.0001$). The length of the second relapse-free interval also correlated with outcome ($p < 0.0001$). 24 patients remained in third CR at last follow-up.
*Conclusions:* A subgroup of patients with second osteosarcoma recurrences achieves long-term survival. A longer second relapse-free interval and a third surgical remission are positive prognostic indicators.

**P075**

**Sixteen children with pleuropulmonary blastoma: Results of the cooperative soft tissue sarcoma group**

S. Kirsch[1], I. B. Brecht[1], T. Dantonello[1], V. Debus[3], C. Int-Veen[1], H. Toomes[2], A. Schuck[6], B. F. Schmidt[1], P. Winkler[2], I. Leuschner[2], D. Harms[4], S. Bielack[4], H. Jurgens[4], T. Spieken[5], T. Klingebiel[7], E. Koscielniak[1], J. Treuner[1]
*([1]CWS Study Group, Department of Pediatric Haematology and Oncology, Olgahospital Stuttgart, Germany, [2]Department of Thoracic Surgery, Klinik Schillerhöhe, Gerlingen, Germany, [3]Department of Radiotherapy, University of Münster, Münster, Germany, [4]Department of Paidopathology, University of Kiel, Kiel, Germany, [5]Department of Pediatric Haematology and Oncology, University of Münster, Münster, Germany, [6]Department of Pediatric Haematology and Oncology, University of Frankfurt, Frankfurt, Germany, [7]Gerhard-Domagle Institut of Pathology, University Munster, Germany)*

*Purpose:* Analysis of data from patients with the extremely rare pleuropulmonary blastoma (PPB) treated within the CWS 81–96 trials.
*Methods:* Evaluation of 16 patients (8 boys and 8 girls) with a median age of 3 years treated between 7/1982 and

10/2000 according to the CWS studies. Median follow up of was 71.67 months (survivors).
*Results:* Initial resection of the tumor was carried out in 14 patients, which resulted in complete excision in 7 cases (3 IRS I, 4 IRS II), but macroscopic tumor residuals (IRS III) in 8 patients. The subsequent chemotherapy consisted of the typical treatment scheme for high risk sarcoma. 4 patients were irradiated with dosages between 18 and 45 Gray and 6 patients underwent a secondary resection. The 5-years overall and event free survival of the collective are 70.1%. Both patients with progressive disease (one of them initial stage IV) died of disease, but 12 of the 14 patients, who reached complete remission did not develop local recurrence and are alive (75%).
*Discussion:* Pleuropulmonary blastoma is a very rare lung malignancy in children. Though prognosis is reported to be very poor, the outcome of patients treated according to the CWS-guidelines shows promising results.

**P076**

**Pulmonary nodule at fluorine-18 deoxyglucose positron emission tomography (FDG-PET) in a child with Ewing's Sarcoma**

[1]E. Kısmet[1], T. Kendirli[1], E. Demirkaya[2], Y. Narin[3], V. Köseoğlu[1]
*([1]Department of Pediatric Oncology, Gülhane Military Medical School of Medicine, Ankara, Turkey, [2]Department of Pediatrics, Gülhane Military Medical School of Medicine, Ankara, Turkey, [3]Department of Nuclear Medicine, Gülhane Military Haydarpaşa Training Hospital, İstanbul, Turkey)*

Ewing's sarcoma is one of the childhood tumors that may metastasize to the lungs. The pulmonary metastasis may be the first presenting sign of the tumor or encountered during chemotherapy or at follow-up. Positron emission tomography (PET) using the glucose analogue F-18-Fluorodeoxyglucose (FDG) is a promising new method for non-invasive differentiation of benign and malignant solitary pulmonary nodules in patients with malignancy. We report a patient with Ewing sarcoma with a pulmonary nodule without abnormal accumulation of FDG and pulmonary fibrosis with false positive accumulation.

A 4-year-old boy who had been treated for Ewing sarcoma had pulmonary nodule considering metastasis at follow-up by computed tomography. FDG-PET scan was negative but there was abnormal accumulation at the other lung where a pulmonary nodule excised before and proven histopathologically benign. Although the nodule that was negative at FDG-PET disappeared in a one-month-period there was no change on CT at the lung where accumulation of FDG was detected. We conclude that FDG-PET is helpful to differentiate malign pulmonary nodules from benign ones but false positive results should be kept in mind especially if there are fibrotic changes secondary to recent pulmonary operation.

**P077**

**Role of caspase 8 in death receptor-mediated apoptosis in Ewing's sarcoma**

U. Kontny, A. Lissat, R. Klein, T. Vraetz, R. Klein, C.M. Niemeyer
*(Division of Pediatric Hematology and Oncology, Department of Pediatrics and Adolescent Medicine, University of Freiburg, Germany)*

*Purpose:* The prognosis for patients with metastatic or relapsed Ewing's sarcoma is poor. Characterizing the pathways leading to cell death in this tumor could lead to the development of new rational therapeutics. In this study we have analyzed the death receptor pathways in Ewing's sarcoma.
*Methods:* Nine Ewing's sarcoma cell lines were incubated with the death ligands Fas ligand or TRAIL. Apoptosis was measured by flowcytometry. Expression of caspases, bcl-2 and IAP-family members was studied by RNA-multitemplate protection assay and immunoblot. Transient and stable transfection of caspase 8 were done using lipofectamine.
*Results:* Seven out of nine Ewing' sarcoma cell lines studied were sensitive to TRAIL-mediated apoptosis. The two TRAIL-resistant cell lines were found to be caspase 8-deficient. Incubation of resistant cell lines with interferon-gamma could restore sensitivity to TRAIL-mediated apoptosis. Interferon-gamma induced expression of caspases 1, 7, 8, bak, and downregulated the expression of surviving. TRAIL-induced apoptosis could be inhibited by a caspase 8-specific inhibitor, however, transfection of caspase 8 into caspase 8-deficient Ewing's sarcoma cells did not restore sensitivity to TRAIL-mediated apoptosis.
*Discussion:* While the expression of caspase 8 is essential for death-receptor mediated apoptosis, the sensitizing effect of interferon-gamma goes beyond the induction of caspase 8-expression.

**P078**

**Outcome of patients with pulmonary metastasis of soft tissue sarcomas following surgical resection**

R. Kopp[1], G. Meimarakis[1], S. Piltz[1], H.O. Steitz[1], R. Issels[2] R. Hatz[1], K.W. Jauch[1]
*([1]Department of Surgery, [2]Department of Oncology, Klinikum Großhadern, LMU Munich, Germany)*

*Introduction:* Pulmonary metastasis are observed in patients with soft tissue sarcomas (STS). Surgical resection of pulmonary metastasis is still the most promising treatment based on the minor response of soft tissue sarcomas following radio- or chemotherapy.
*Patients and methods:* Between 1990 and 2003 we have treated 55 patients (25 female, 32 male; mean age: 48 years) for pulmonary metastasis of soft tissue sarcomas with the intention of curative resection. The following parameters were retrospectively analysed: age, gender, primary treatment, tumor localisation, histopathological type and grading, size and number of metastasis, tumor-free interval, multiple resections and adjuvant chemotherapy.
*Results:* Complete resection of pulmonary metastasis detectable at the time of operation was achieved in 44 patients (80%). Overall-survival after 5 years was 37.1% (follow-up: 27.8 months). Perioperative mortality was 2.6%. Minor complications including wound infection, hematothorax or pneumonia were observed in 5.2%. Survival was associated with evidence of complete tumor resection (43% vs. 18%; $p < 0.004$), size of metastasis (<4 cm) and less invasive operative procedures.
*Conclusions:* Operative treatment of pulmonary metastasis in patients with soft tissue sarcomas is an important therapeutic option associated with low perioperative morbidity and mortality and acceptable survival.

**P079**

**Primary malignant sarcomas of the heart and great vessels – a single center experience**

H. Aebert, B. Kosan, M. Rudert, R. Teichmann, W. Budach, J.T. Hartmann
*(Medizinische Klinik II, Universitätsklinikum Tübingen, Germany)*

Sarcomas of the heart are very rare entities. The most common subtypes included leiomyo- and angiosarcoma.

Prognosis of these patients (pts) is very poor because of locally advanced or metastatic stage at diagnosis.

Between 6/2001 and 10/2003 12 pts with primary sarcoma of the heart or great vessels have been identified in the local sarcoma registry. Median age was 43 years (22–72). The majority of pts have been referred to the University Hospital after initiation of treatment, e.g. surgical incomplete resection. All pts were symptomatic with reduction of vitality, anorexia, dyspnoea, or neurological symptoms at the time of reception. Tumors were located in atrium [($n=5$), left (3)/right (2)], Art. pulmonalis ($n=3$), Aorta ($n=2$), pericardium, left chamber, ($n=1$ each). Six pts presented with localised disease and 5 pts underwent curative intended resection. One pt was considered locally irresectable. Six pts had advanced disease including brain mets (4 pts), lymph nodes involvement (2), lung mets (3) and liver mets (2). Five of these 6 pts underwent palliative (incomplete) resection to relieve the physiologic effects of the tumor on the heart or the arteria. Three out of 5 pts with curative intended surgery have developed local recurrence within 6, 8 and 25 mos despite adjuvant treatment. One single pt with an PNET sarcoma subtype is still alive without relapse after completion of pre- and postoperative chemotherapy and another pt with a paraganglioma is still disease-free after tumor resection only.

Palliative chemotherapy including adriamycin and ifosfamide has been applied in 7 pts and 4 pts responded to treatment (1 PR, 3 SD). Palliative radiation included 3 pts with brain metastases and a single pt with progressive primary tumor. At a median follow up 13 mos (2–28) 2 pts had NED, 3 pts are alive with disease and 7 pts have died due to tumor progression.

In conclusion, pts with primary sarcomas of the heart and the great vessels were of young age, and half of them presented with locally or distant advanced disease. These pts should preferentially be referred to a tertiary Cancer Center immediately after diagnosis without preceding treatment attempts. Although the majority of pts cannot treated curatively due to the delay in diagnosis, an optimized treatment approach including neoadjuvant chemo-/radiotherapy might enhanced the rate of complete resection depending on the histologic subtype and responsiveness to treatment.

### P080

### New pulmonary nodules in patients with malignancies: An retrospective analysis

B. Kosan[1], G. Friedel[2], T. Kyriss[2], H. Toomes[2]
*([1]Department of Thoracic, Cardiac and Vascular Surgery, Eberhard-Karls-University Tuebingen, Germany, [2]Department of Thoracic Surgery, Schillerhoehe Hospital, Gerlingen, Germany)*

*Introduction:* In patients with a malignant disease newly discovered pulmonary nodules are highly suspected of metastases or lung cancer. The purpose of our retrospective study is to determine the proportion of primary and secondary malignant pulmonary lesions.
*Patients:* Between 1995 and 2003 87 patients (33 male, 54 female), mean age 64 years (range 29–78) underwent surgery because of suspected lung metastases in our hospital. The underlying malignancies were breast cancer (25%), colorectal cancer (18%), prostate cancer (11%), renal cancer (8%), melanoma (7%), sarcoma (5%) and others (26%). Seven patients had two and one three malignancies in their medical history. According to oncologic standards surgery was performed in VATS technique in (44%) cases. The remaining patients (56%) underwent thoracotomy.
*Results/discussion:* Histology showed malignancy in 37%. In 26% primary lung cancer was diagnosed, only 11% had metastases and 63% had benign or inflammatory nodules. 65% of the lung cancer cases and 78% with metastases were asymptomatic. The lung lesions were accidental findings due to examinations or follow-up. Lung cancer was associated with colorectal cancer in 5, breast cancer in 4, prostate cancer in 3 patients. No perioperative mortality occurred. We conclude that newly diagnosed pulmonary nodules should be resected and histologically examined.

### P081

### Limb salvage by plastic reconstruction in patients with soft tissue sarcoma of the lower extremity

Th. Kremer[1,2], Ch. Leowardi[1], M. Wente[1], U. Hinz[1], H. Friess[1], G. Germann[2], Ch. Herfarth[1], M.W. Büchler[1], M.H.M. Schwarzbach[1]
*([1]Department of General-, Visceral and Trauma Surgery, University Clinic of Heidelberg, Germany, [2]Departement of Plastic-, Reconstructive-, and Hand Surgery, - Burn Center -, Plastic- & Hand Surgery, University of Heidelberg, BG Trauma Center Ludwigshafen, Germany)*

*Introduction:* Reconstruction of the body surface is a major focus in modern surgery for soft tissue sarcoma (STS). Plastic reconstruction often facilitates radical oncologic resection and conservation of extremity function.
*Methods:* Analysis of prospectivly collected data from adults with STS of the lower limb and plastic reconstruction (1988–2004). Assessment focused on type of reconstruction, tumor manifestation, oncologic outcome and extremity function (MSTS-score). SAS for statistical analysis.
*Results:* 45 (mean age 48 years, 1.5:1 male:female) of 128 patients needed 52 plastic reconstructions after STS-resection. 46% presented with local recurrence, 54% with primary tumors. Histologically liposarcoma and malignant fibrous histiocytoma dominated. Defects were located at groin and thigh (64%), knee (21%) and lower leg (13%). Reconstruction was achieved by pedicled flaps (36%), random pattern flaps (25%), split-thickness grafts (18%) and free microvascular transplantations (6%). 62% received intraoperative radiotherapy. 8% major, 34% minor complications, 27% reoperations and no lethality or major amputation were observed. Local tumor control was 81%. At a mean follow-up of 73 month 49% died. Functionality was high (MSTS-score 70.3%)
*Discussion:* Plastic reconstruction was necessary in 1/3 of patients after limb preserving STS-resection. Long-term functional results and good local tumor control underline the importance of plastic surgery in a multimodality treatment strategy for STS of the lower limb.

### P082

### The relationship of cytogenetics findings with clinical outcomes in Ewing sarcoma

J. Kruseová, E. Stejskalová, R. Kodet, D. Sumerauer, J. Starý
*(Departments of Pediatric Hematology and Oncology and Pathology, Motol Hospital, Prague, Czech Republic)*

*Purpose:* To show the relationship of cytogenetics findings with clinical outcomes in Ewing sarcoma.

We analysed eleven patients. Four of them had a trisomy of chromosome 8 and one a trisomy 12: primary disease 1x scapula (4.5 ml), 2x pelvis (114, 283 ml), 2x chest (423, 1036 ml). One patient had metastatic dissemination in his skeleton and died of disease progression. Others didn't have metastases and achieved CR (median follow-up 43 months). A patient with chest tumours relapsed 13 months after the end of treatment, tumours appearing in his lungs. He is currently undergoing second line treatment. Unbalanced t(1.16) was ascertained in two patients – one had shoulder blade involvement (2578 ml), with generalisation into lungs and upper-clavicular lymph nodes. He had only two copies of the der(16) t(1.16) but no other aberration. He died during treatment of disease progression. The other one had primary disease in his femur (1165 ml) with pulmonary metastasis. He is alive with untreatable disease progression. Along with the t(1.16) he had a modal number of 51–56 chromosomes, two markers, a deleted 5p and 22q chromosome and gains of chromosomes 8 12 20 and 21. We found no correlation of trisomy 8 and 12 and clinical outcome, but the presence of the t(1.16) in our patients karyotype predicted refractory disease.

### P083

**Comparison of treatment concepts for extraosseous Ewing Tumours (EET) within consecutive trials of two GPOH cooperative study groups**

R. Ladenstein[1], U. Pötschger[1], H. Jürgens[2], M. Paulussen[2], E. Koscielniak[3], J. Treuner[3]
*(The GPOH Cooperative Soft Tissue Sarcoma and Ewing Tumour Study Group, [1]St. Anna Kinderspital Wien, Austria, [2]Universitätskinderklinik Münster, Germany, [3]Olgahospital, Stuttgart, Germany)*

*Purpose:* Comparison of strategies in EET patients (pts) treated within two GPOH Cooperative Study Groups (CSG).
*Patients/methods:* 236 pts with EET are evaluable: 113 pts in CWS and 123 pts in ETS studies. The median age at diagnosis is 15 years (6 months–58 yrs); 133 pts are males. Primary sites were extremities in 62 pts and central sites in 174 pts. Chemotherapy was VACA/CE-VAIA(VIDE) based in both CSG. 85 pts had initial surgery (Sx), 56 pts later Sx. Radiotherapy (median dose 45 Gy) was given in 141 pts (in 87 pts in addition to Sx).
*Results:* The 5 year overall survival rate (OS) of 236 patients is 60% (EFS 49%). OS in non-metastatic disease (NMD) is 70% and 33% with metastases at diagnosis (60 pts) ($p < 0.001$). Age significantly influenced OS rate: pts <14 yrs (103 pts) 70%, 14–18 yrs (54 pts) 57%, >18 yrs (79 pts) 47%. Best outcome in NMD was observed in studies CWS91 (OS 84%) and EuroEwing99 (OS 75%) followed by CWS-concepts (study 96: OS 71% and studies 81/86: 69%) and earlier ETS-concepts (studies 91/92: OS 67% and studies 81/85/86: OS 38%)
*Conclusions:* Induction treatment intensity (EuroEwing99) and early local control measures (CWS concepts) appear equally important.

### P084

**Nephrotoxicity of platinum derivatives in paediatric sarcoma patients**

T. Langer[1], W. Stöhr[1], M. Paulides[1], S. Bielack[2], H. Jürgens[3], J. Treuner[4], R. Rossi[5], J.D. Beck[1]
*([1]LESS Centre, University Hospital for Children and Adolescents, Department of Paediatric Oncology, Erlangen, Germany, [2]Osteosarcoma Trial Centre, University Children's Hospital Muenster, Department of Paediatric Haematology and Oncology, Muenster, Germany, Ewing's Sarcoma Trial Centre, University Children's Hospital Muenster, [3]Department of Paediatric Haematology and Oncology, Muenster, Germany, [4]Cooperative Soft Tissue Sarcoma Study, Olgahospital, Department of Paediatric Oncology, Stuttgart, Germany, [5]Hospital for Children and Adolescents, Klinikum Neukölln, Berlin, Germany)*

*Background:* Platinum derivatives are known for their nephrotoxicity. They can impair, to a different degree, glomerular function and induce renal magnesium loss. There are only a few prospective studies with repeated measurements following cessation of antineoplastic therapy in children and adolescents.
*Patients and methods:* 651 sarcoma patients were investigated prospectively for nephrotoxicity in a multicentre trial. The mean period of follow-up was $2.1 \pm 1$ years. Renal function was examined by testing plasma magnesium, plasma creatinine and GFR estimated by the Schwartz formula. We evaluated the incidence, dependencies and the course of impairments.
*Results:* We found no platinum-induced impairments of glomerular function. Frequency of hypomagnesaemia after cisplatin therapy was 12.9% (95%-CI: 7.4–20.4%) and after carboplatin therapy 15.6% (95%-CI: 5.3–32.8%), in comparison with 5.1% (95%-CI: 2.4–9.5%) in patients without any treatment with platinum derivatives. In all patients the frequency of impairments decreased with further follow-up, but the mild decrease of plasma magnesium in platinum-treated patients persisted throughout the study period.
*Conclusion:* Nephrotoxicity after treatment with cisplatin and carboplatin was mild in our study. Further studies have to show if hypomagnesaemia is permanent and if it will result in any clinically relevant impairment.

### P085

**Response rate of fibrosarcoma cells to cytotoxic drugs on the expression level correlates to the therapeutic response rate of fibrosarcomas and is mediated by regulation of apoptotic pathways**

M. Lehnhardt[1], L. Klein-Hitpass[2], C. Kuhnen[3], H.H. Homann[1], A. Daigeler[1], H.U. Steinau[1], L. Steinstraesser[1], O. Müller[4]
*([1]Department of Plastic Surgery, Burn Center, Hand surgery, Sarcoma Reference Center BG University Hospital Bergmannsheil, Ruhr University Bochum, Germany, [2]Institute of Cell Biology (Tumor Research), IFZ, University of Essen, Germany, [3]Institute of Pathology, BG University Hospital Bergmannsheil, Ruhr University Bochum, Germany, [4]Tumor Genetics Group, Max-Planck-Institut für molekulare Physiologie, Dortmund, Germany)*

*Background:* Because of the high resistance rate of fibrosarcomas against cytotoxic agents clinical chemotherapy of these tumors is not established.
*Methods:* HT1080 fibrosarcoma cells were exposed to the chemotherapeutic agents doxorubicin, actinomycin D or vincristine. Gene expression patterns were analyzed by microarray analysis and validated by quantitative real-time PCR.
*Results:* The analysis of the microarray data resulted in 3.309 (actinomycin D), 1.019 (doxo) and 134 (vincristine) probe sets that showed significant expression changes. For the RNA synthesis blocker actinomycin D, 99.4% of all differentially expressed probe sets were down-regulated. In comparison, probe sets down-regulated by doxo comprised only 37.4% of all genes effected by this agent. Closer analysis of the differentially regulated genes revealed that doxo induced cell death of HT1080 cells mainly by regulating the abundance of factors mediating the mitochondrial apoptosis pathway.
*Conclusions:* The response rates on the gene expression level, i.e. the number of genes regulated by the drugs correlate to the clinical effectiveness of the drugs. Doxo seems to exert its cytotoxic mechanism by regulating genes, which are involved in several different apoptosis regulating pathways. The exact knowledge of the genes affected by the drugs will help to understand the diverse modes of soft tissue sarcoma cell death in response to cytotoxic therapies.

### P086

**Feasibility of chemosensitivity testing in soft tissue sarcomas**

M. Lehnhardt, A. Daigeler, L. Steinstraesser, H.H. Homann, H.U. Steinau
*(Department of Plastic Surgery, Burn Center, Handsurgery, Sarcoma Reference Center BG University Hospital Bergmannsheil, Ruhr University, Bochum, Germany)*

*Background:* The role of chemotherapy in soft tissue sarcomas is not clearly defined and is largely restricted to clinical trials.
*Methods:* The objective of this study was to gain additional information about the chemosensitivity of soft tissue sarcomas to seven 7 different chemotherapy agents as single drugs and 4 combinations. Therefore we used an established ATP based in-vitro testing system and examined 50 soft tissue sarcomas. Chemosensitivity was assessed using a luciferin-luciferase-based luminescence assay providing individual chemosensitivity indices for each agent tested.
*Results:* The sensitivity varied widely according to the histological subtypes. The tumors state of cellular dedifferentiation played a crucial role for the efficiency of the chemotherapeutic agents. The sensitivity also depended on the presentation of the sarcoma as a primary or recurrent tumor. The highest sensitivity was demonstrated for actinomycin D. The combination of actinomycin D and ifosfamide yielded a high sensitivity in 76%. Doxorubicin as a mono-therapy or in combination with ifosfamide achieved high sensitivity in 70% and 72%, respectively.
*Conclusions:* Chemosensitivity testing is feasible in soft tissue sarcomas. It can be used to create sensitivity and resistance profiles of established and new cytostatic agents and their combinations in soft tissue sarcomas.

### P087

**Allocation of soft tissue sarcoma patients of extremities and wall: 10 years overview**

S. Leinung, P. Wuttke, M. Schönfelder, P. Würl
*(Surgical Clinic, University of Leipzig, Germany)*

*Patients and methods:* Over a 10-year period, the patients referred to us with a soft tissue tumor (suspicios of soft tissue sarcoma only) of the extremities and wall of the trunk were analyzed retrospectively. The aim of the present study was to investigate the differential diagnoses, the number of incompletely operated soft tissue sarcoma (STS), and local recurrences.
*Results:* A total of 490 patients with an soft tissue tumor (STT) were referred to our department, and of these patients 55% were diagnosed with an STS. In addition to STS, the differential diagnoses for STT included 2% lymphomas, 18% isolated carcinoma metastases, 18% benign mesenchymal tumors, 5% inflammatory processes, and 2% old hematomas. Only 45% of the STS had not undergone previous surgery. Of these, 15% had been incompletely resected, while 39% of the STS patients were admitted with a local recurrence.
*Discussion:* In view of the numerous differential diagnoses of an STT, both the possibility of an STS and also carcinoma manifestations in the soft tissues should receive more attention. With the aim of reducing the relatively high number of STS re-resections and local recurrences, the treatment of patients with suspicious STT should be reserved for a specialized center.

### P088

**Gastrointestinal stromal tumours: Results of surgical therapy**

C. Leowardi, U. Hinz, M.N. Wente, T. Kremer, G. Mechtersheimer, H. Friess, C. Herfarth, M.W. Büchler, M.H.M. Schwarzbach
*(Department of Surgery and Institute of Pathology, University of Heidelberg, Germany)*

*Purpose:* Surgical treatment is the standard therapy for resectable gastrointestinal stromal tumours (GIST). The aim of this study was to analyze the results of surgical therapy under consideration of histopathological reclassification of this tumour entity.
*Patients/methods:* Observational study from a single center during a representative period. All patients who underwent surgery for primary or recurrent GIST between 1992 and 2004 were included. Immunohistochemical reclassification was performed. Clinical data were gathered prospectively in a computerized registry and subjected to univariate and multivariate analyses together with the follow-up results.
*Results:* With a median age of 57 years, 49 male and 36 female patients ($n = 85$) were referred for 71 primary tumors and 14 recurrences. Primary tumours were mainly located in the stomach ($n = 41$) and in the small bowel ($n = 18$). According to Fletcher-classification high-risk tumours dominated (53%), only in these tumours recurrent disease was observed after surgery (19%). Complete resection could be achieved in 84.5%. With a median follow-up of 50 months, 5-year-survival-rates of patients with complete resected tumours (91.5%) and patients with primary tumours at first presentation (84.5%) were significantly better than those of patients with incomplete resected tumours and those who presented with recurrent disease ($p < 0.0001$, $p = 0.0004$).
*Discussion:* Complete surgical resection is of decisive prognostic relevance and represents a potentially curative treatment for the majority of patients with resectable GIST.

### P089

**Malignant vascular tumors: Clinical presentation, surgical therapy and long-term prognosis**

C. Leowardi[1], U. Hinz[2], M.N. Wente[1], T. Kremer[1], G. Mechtersheimer[3], F. Willeke[1], H. Friess[1], C. Herfarth[1], M.W. Büchler[1], M.H.M. Schwarzbach[1]
*([1]Department of Surgery, [2]Unit for Documentation and Statistics, [3]Institute of Pathology, University of Heidelberg, Heidelberg, Germany)*

*Purpose:* Clinical knowledge of malignant vascular tumors (MVT) in adults is limited. The aim here was to analyze clinical presentation, surgical therapy, and prognosis.
*Patients/methods:* Observational study from a single center. Patients with primary or recurrent MVTs between 1988 and 2004 were included.
*Results:* Of 568 sarcoma patients 43 presented with a MVT (7%). With a median age of 53 years, 24 males and 19 females were referred for 30 primary tumors and 13 recurrences. Tumors were diagnosed in the extremities ($n = 16$), trunk ($n = 3$), abdomen ($n = 15$), retroperitoneum ($n = 7$), and thyroid gland ($n = 2$). Subgrouping revealed 22 angiosarcomas (51.2%), 9 malignant hemangiopericytomas (20.9%), 8 malignant epiheloid hemangioendotheliomas (18.6%), and 4 lymphangiosarcomas

(9.3%). Median overall survival was 21.4 months with 2-, 5-, and 10- year overall survival rates of 41.5%, 38.3%, and 18.8%, respectively. Patients with tumors of the extremities and trunk and patients without metastasis at presentation had a better prognosis than those with abdominal or retroperitoneal tumors ($p = 0.0122$) and patients with metastasis at presentation ($p = 0.0187$).
*Discussion:* MVTs are rare, presenting with various clinical symptoms and an ubiquitous occurrence. Their clinical course is aggressive and thus prognosis is poor. Complete surgical resection represents a potentially curative treatment, especially for tumors of the extremities and trunk.

**P090**

**Survival and prognostic factors in patients with pulmonary metastases from soft tissue sarcomas**

K.A. Gawad, L. Liebl, F. Elson, J. Kaifi., E. Yekebas, J.R. Izbicki
*(Department of General-, Visceral- and Thoracic Surgery, University Medical Center Hamburg-Eppendorf, Germany)*

*Background/aim:* Few studies on the role of pulmonary metastasectomy in patients with soft tissue sarcomas (STS) are currently available. The small number of such studies is mainly noncontemporaneous and predate a more radical approach including metastasectomy in cases of multiple metastases as well as repeat resection. The aim of our study was to evaluate the overall prognosis after such surgery and to identify clinically relevant prognostic factors.
*Patients/methods:* Of 475 STS patients prospectively followed in our database 48 patients underwent 67 metastasectomies. Further studies were limited to such patients with at least 3-years follow up.
*Results:* 26 patients (12 female/14 male) underwent 37 metastasectomies between 1990 and 2002. 7 (27%) of those patients underwent at least one repeat resection for recurrent metastases. 19 were more than 2 cm whereas 18 were less than 2 cm in diameter. Size of metastases, extent of resection and extension of disease (solitary or multiple metastases) were identified as possible independent prognostic factors. The data suggest tumor size as one relevant prognostic factor: Patients with a maximum metastasis size of 2 cm or less with a longer disease free interval.
*Conclusion:* Detailed subgroup analysis may isolate patients with particular benefit of resection of pulmonary metastasis.

**P091**

**Prognostic relevance of FDG-PET in patients with malignant peripheral nerve sheath tumors (MPNST) and neurofibromatosis type-1 (NF-1)**

K.A. Gawad, L. Liebl, W. Brenner, R. Buchert, A. von Daimling, C. Hagel, R.E. Friedrich, V.F. Mautner
*(Departments of General-, Visceral- and Thoracic Surgery, Nuclear Medicine and Mund-, Kiefer-, Gesichtschirurgie, University Medical Center Hamburg-Eppendorf, Germany)*

The aim of this study was to assess the potential of FDG-PET for risk assessment and outcome prediction in NF1 patients with MPNST.
*Methods:* F-18-fluorodeoxyglucose-PET imaging was performed in 17 patients with NF-1 and MPNST prior to therapy. The mean follow-up time was 18 (2–53) months. SUV was calculated for each tumor and correlated to tumor grade and patient outcome.
*Results:* 10 of 17 patients died within 11 (4–14) months. 3 patients had an SUV <3 (2.1–2.8; tumor grade II each). None of these patients developed metastases during follow-up (31–53 months). 14 patients had an SUV >3, 4 of which had a tumor grade II (SUV 4.0–11.0). 3 of these 4 died, 1 patient is still alive (follow-up: 2 months). The remaining 10 patients had a tumor grade III (SUV 3.2–11.6). 7 of these 10 patients died, 3 are still alive (follow-up: 10, 20 and 41 months). In Kaplan-Meier analysis, patients with an SUV >3 had a significantly ($p = 0.034$) shorter mean survival time of 15 compared to 45 months in patients with an SUV <3. Tumor grading did not reveal differences in survival time between grades ($p = 0.249$).
*Conclusion:* Pretherapeutic tumor SUV obtained by FDG-PET was a significant parameter for risk assessment and prediction of survival in NF1 patients with MPNST.

**P092**

**Chemotherapy in alveolar soft part sarcoma: Single institution experience and review of the literature**

T. Lindner, D. Pink, P.-U. Tunn, P.C. Thuss-Patience, A. Kretzschmar, P. Reichardt
*(Department of Hematology and Oncology, Charité Campus Buch, HELIOS-Klinikum Berlin-Buch, Berlin, Germany)*

*Background:* Alveolar soft part sarcoma (ASPS) is a rare tumour. Published series about treatment and outcome are scarce. Conclusive data about response to chemotherapy are not available.
*Results:* From our registry containing 830 patients, we identified 9 patients with ASPS. From the literature 100 cases of adult patients and 15 children with sufficient data about chemotherapy were identified. Response to 1st-line chemotherapy in 124 patients was: CR 2%, PR 4%, SD 44%, PD 49%. 316 patients with stage IV disease were evaluable for the metastatic sites. The incidence of brain metastases is 30.7% (97/316). Brain metastases were detected at a median interval of 37 months (range 0–396 months) after primary diagnosis. The median survival after diagnosis of brain metastases is 19 months. The median survival for patients with metastases disease treated by chemotherapy is 45+ months.
*Conclusions:* ASPS shows a high incidence of brain metastases, at least 3 times higher than that of other soft tissue sarcomas. Chemotherapeutic regimens well established for the treatment of soft tissue sarcomas lack efficacy in ASPS. Staging of metastatic ASPS should routinely include imaging of the brain. ASPS should not be treated with chemotherapy outside of controlled clinical trials.

**P093**

**Geldanamycin induces growth arrest and apoptosis of embryonal and alveolar rhabdomyosarcoma cells**

M. Majka, E. Lesko, M.Z. Ratajczak
*(Department of Transplantation, Jagiellonian University Medical College, Cracow, Poland)*

Geldanamycin (GA) is a new potential drug that has been shown in various cell systems to inhibit tumour cells proliferation, survival and motility by binding to various proteins including heat shock proteins that play critical role in tumor cells growth.

In this study we looked at the GA effect on rhabdomyosarcoma (RMS) cell lines. In our study we used embryonal (ERMS) and alveoral (ARMS) cell lines. We assessed effect of GA on RMS cells proliferation, apoptosis, cell cycle and motility.

We found that GA had an anti-proliferative effect on RMS cells by inducing their apoptosis and causing cell cycle arrest. The ERMS cells were more susceptible to GA treatment.

RMS often gives distant metastasis to various organs including bone marrow. RMS cells respond to HGF with increase motility and chemotaxis. We tested if GA could inhibit HGF-induced chemotaxis of RMS cells. We found that GA was able to inhibit strongly chemotaxis of RMS cells toward HGF gradient in dose dependent manner.

New therapies are still needed in RMS disease. Our data shows that GA could be a potential new drug in RMS treatment. It blocks RMS proliferation, decreases its survival and what is very important inhibits chemotaxis of tumour cells.

### P094

### TNM staging in Ewing tumors: A recommendation from the (EI)CESS/EE 99 studies

D. Manner, C. Liebscher, A. Hunold, M. Paulussen, H. Juergens
*(University Children's Hospital, Department of Ped. Hematology and Oncology, Münster, Germany)*

*Background:* Tumor size and metastases are known risk factors in Ewing tumors. Adequate staging is essential to stratify treatment intensity, but TNM staging is not established.
*Patients and methods:* The validity of TNM staging was tested based on tumor volume (T1 $\leq$200 ml; T2 >200 ml $\leq$500 ml; T3, >500 ml), the presence or absence of lymph node metastases (N0, N1), and distant metastases (M0, no metastases, M1 lung/pleura metastases; M1a $\leq$5 nodules; M1b >5 lesions; M2 bone metastases, M2a, 1 lesion; M2b >1 lesion and/or microscopic bone marrow contamination; M3 multi-system metastases). 1371 patients from consecutive studies (1981–2003) were analyzed.
*Results:* Ten-year event-free survival (EFS) was 0.46. EFS in patients without metastases (T1-3N0M0) was 0.61 compared to 0.32 in metastatic patients (T1-3N0,1M1-3), $p = 0.0001$. In non-metastatic patients, tumor volume discriminated EFS: T1:0.67; T2:0.50; T3:0.44, $p = 0.0001$. Lymph node metastases correlated to unfavorable prognosis (N0:0.54, Nn1:0.28, $p = 0.0008$). The difference in EFS between pulmonary, skeletal and multi-system dissemination was significant: M1:0.47, M2:0.29; M3:0.17, $p = 0.0001$. The discrimination of M1 subgroups (M1a/M1b) was of prognostic relevance ($p = 0.0313$); M2 subgroups (M2a/M2b) discriminated outcome less clearly ($p = 0.0657$).
*Conclusion:* TNM staging is appropriate in Ewing tumors and should be incorporated in future trials.

### P095

### IGF1R pathway blockage: A new strategy in ewing tumour (ET) treatment?

A.S. Martins[1], C. Mackintosh[1], D. Herrero Martín[1], T. Hernández[2], E. de Alava[2]
*([1]Laboratory of Molecular Pathology, [2]Tumor Bank, Centro de Investigación del Cáncer, Universidad de Salamanca, Salamanca, Spain)*

ET cell survival and proliferation depends on several major autocrine loops. Its blockade is a promising therapeutic approach. We analyzed the in vitro impact of IGF1R pathway blockade by ADW742, a specific inhibitor of this receptor, on ET cell line proliferation, apoptosis, cell cycle, pathway phosphorylation, colony formation and motility, alone and in combination with Vincristine (VCR) or Doxorubicin (DXR).

IGF1R expression, constitutive, and ligand-inducible phosphorylation of IGF1R were detected in 4/4 ET cell lines, indicating a functional pathway. Combined treatments showed a reduction of the proliferative rate ranging 50–85%, and isobolograms showed synergy. Apoptotic rates reached nn%-80%. Interestingly, IC50 of phosphorylation of AKT plummeted below 0.25 μM in combined treatments (5 μM with ADW742 alone), suggesting that this molecule could be an interesting surrogate marker of tumor response to therapy. Combined therapy strongly inhibits colony formation and mobility of ET cells.

Inhibition of ET cell proliferation by ADW742 is mediated through blockade of IGF1R signaling. A significant reduction of tumor cell growth and increase in apoptosis, mainly by blocking both IGF1R-AKT/MAPK pathways was seen, supporting a potential role for ADW742 in combined therapies of ET, and the use of p-AKT as an early marker of response to treatment.

### P096

### Phase II study of dose-intensive chemotherapy with adriamycin and ifosfamide followed by high-dose ICE with PSCT in locally advanced soft tissue sarcomas

F. Mayer[1], W. Brugger[2], H.G. Mergenthaler[3], H. Aebert[1], R. Teichmann[1], M. Rudert[1], P.M. de Zwart[1], W. Budach[1], L. Kanz[1], J.T. Hartmann[1]
*([1]Medical Center and Interdisciplinary Sarcoma Center, University of Tuebingen, Germany, [2]Klinikum, Hematology and Oncology, Villingen-Schwenningen, Germany, [3]Katharinenhospital, Hematology/Oncology, Stuttgart, Germany)*

*Background:* To determine whether dose-intensive neoadjuvant chemotherapy is a feasible and effective approach in patients (pts) with unresectable soft tissue sarcoma (STS).
*Patients/methods:* Treatment consisted of 3 cycles of adriamycin (75 mg/sqm) on day 1 and ifosfamide 12 g/m$^2$ as 72 h continuous infusion (AI) followed by one cycle of high-dose chemotherapy (HD-ICE), etoposide 500 mg/sqm, carboplatin 500 mg/sqm and ifosfamide 4 g/m$^2$, applied on day −4 through −2, and retransfusion of PBSC on day 0. Presence of distant metastases deemed resectable was allowed.
*Results:* Between 11/97–2/00 and 4/01–6/04 20 pts, median age 45 (21–62) years, with high grade STS and different locations (retroperitoneum, trunk, extremity) have entered into this prospective trial, either with primary locally advanced STS at primary diagnosis (65%) or with local recurrences (35%). Four pts had additional single resectable metastases in the lung (3) or the liver (1). Fifteen pts (79%) have completed all 3 planned AI cycles and attained a disease stabilisation/minor (47%) or partial remission (32%); one pt is still ongoing. The other 4 pts revealed disease progression after the first ($n = 1$) or second AI cycle ($n = 3$). Two pts did not receive HD-ICE because of ineffective apharesis and grade III neurotoxicity. HD-VIC was well tolerated without dose reduction in the remaining 10 pts (2 pts still ongoing). Non-haematological grade III toxicity consisted of emesis in 2 pts (22%), and mucositis as well as neurotoxicity in one pt each (11%). After PSCT leucocytes and thrombocytes counts recovered on day 10 and 12, respectively. Tumor assessment revealed one additional PR following HD-ICE. After completion of AI+HD-ICE 3 pts without significant tumor shrinkage underwent preoperative radiation according to protocol. In total, 11 of 17 currently evaluable pts (65%) were allocated to surgery. R0 resection was performed in 9 of 11 pts (82%). In one pt a lung metastasis has been removed simultaneously. After a median follow up time of

18+ mos (range, 4–72+) the 1-yr PFS rate is 26% and the 1-yr survival rate 76%. Depending on the resection status median survival is 38 or 18 mos ($p < 0.01$).
*Conclusion:* Neoadjuvant AI plus HD-ICE with autologous PSCT is a feasible regimen with an acceptable toxicity profile in pts with locally advanced STS. R0 resection as the primary end point was realised in 65% of the pts.

### P097

**Metastatic rhabdomyosarcoma report: SIOP trial MMT 98**

H.P. McDowell[1], A. Foot[2], C. Bergeron[3], M. Jenney[4], M. Stevens[2], O. Oberlin[5], C. Ellershaw[6], D. Machin[6]
*([1]RLC NHS Trust, Liverpool, UK, [2]Royal Hospital for Children, Bristol, UK, [3]Centre Leon Berard, Lyon, France, [4]Llandough Hospital, Cardiff, UK, [5]Institut Gustave Roussy, Villejuif, France, [6]UKCCSG Centre, Leicester, UK)*

The SIOP metastatic sarcoma protocol MMT98 ran from July 1998 until November 2004. Patient stratification was standard risk, (SR) <10 yrs old, no bone marrow and/or no bone involvement, and poor risk (PR), >10 yrs old or with marrow and/or bone involvement. SR patients received a 6 drug regime for 27 weeks with local treatment, followed by 9 courses of low dose vincristine, actinomycin D and cyclophosphamide (VAC). PR patients entered a window study of either carboplatin or doxorubincin followed by high dose sequential monotherapy using cyclophosphamide, etoposide and carboplatin, local treatment and then VAC × 9. Centres unable to undertake high dose monotherapy administered SR treatment.

There were 38 SR, 74 PR patients, 28 on the SR protocol, 46 on the PR protocol. Median follow up SR is 13.89 mths, 2 year EFS 54.05%, OS 63.43%, for PR is 11.40 months, 2 year EFS 4.26% OS 37.69%. PR patients on the SR protocol, 2 year EFS 42.11%, OS 51.07% vs. 11.98% and 30.33% for the PR protocol.

This initial analysis shows survival comparative to other studies for the SR patients. Use of high dose monotherapy has failed to provide a beneficial effect for PR patients.

### P098

**Effectiveness of ICE-CAV in high risk Ewing sarcoma family tumors: A single institution study**

G.M. Milano, R. Cozza, L. De Sio, I. Ilari, A. Castellano, P. Fidani, A. Serra, A. Jenkner, C. Dominici, C. De Laurentis, A. Donfrancesco
*(Bambino Gesù Children's Hospital IRCCS, Rome, Italy)*

*Purpose:* Ewing's sarcoma (ES) and extraosseous Ewing's sarcoma/PNET (EES) share histopatologic characteristics, constituting the Ewing's sarcoma family. Risk factors are volume >200 ml, site, bilateral pulmonary metastasis, EWS-FLI transcript and bone marrow involvement.
*Methods:* ICE regimen was active and well tolerated in phase I/II studies, both in Ewing/PNET. The induction regimen consisted of 2 ICE (Ifosfamide 1800 mg/m$^2$/d × 5 days. Carboplatin 400 mg/m$^2$/d × 2 days, Etoposide 100 mg/m$^2$/d × 5 days), 2 CAV (Cyclophosphamide 1500 mg/m$^2$/d × 2 days plus Doxorubicin 75 mg/m$^2$ and Vincristine 1.5 mg/m$^2$ continuous infusion over 72 hrs); courses were repeated every 21–28 days, G-CSF was administered.
*Patients:* Eleven ES and 8 EES, median age 107 months, were enrolled. 5/19 had bilateral lung metastasis and 3/19 bone marrow involvement. 38 courses of ICE-CAV were administered. Grade 3–4 hematological toxicity occurred in all ICE courses.
*Results:* Objective response-rate (CR + PR + MR) after ICE-CAV was 94.7% (3CR, 15 PR, 1PD). 17/19 pts completed protocol (total 7 courses): after a median follow up of 23 months (range 6–91) 13 pts are DF, 1 AWD after relapse and 5 DOD. Over 90% CR rate and 63% 5 years OS seem promising and we plan to enrol additional patients.

### P099

**Profile of children with bone and soft tissue sarcoma admitted with febrile neutropenia**

R. Mittal, H. Mottl, J. Nemec
*(Department of Medical Oncology, Kuwait Cancer Control Centre, Kuwait)*

*Objectives:* To study the incidence and outcome of children who were admitted with febrile neutropenia after receiving chemotherapy for bone and soft tissue sarcomas.
*Methodology:* The clinical data of all the cases of bone and soft tissue sarcomas, who were admitted with febrile neutropenia (from 1.1.2003 to 31.12.2004) was recorded in a pre formed Performa.
*Results:* A total of 10 patients of bone and soft tissue sarcomas received 80 courses of various chemotherapies during the study period. Various chemotherapy protocols (mainly European) were used according to the primary lesion. Fifteen of the 20 episodes were recorded in children with diagnosis of ES/PNET. Prophylactic growth factors were used in all except one patient. The median day of onset of fever was day 9 (range 5–15), Median days for ANC recovery was 5 days (range 3–8), and for platelets 7 days (range 5–10). Fever was the only presenting symptom in majority of the case. No microbial positivity was recoded in any case. Empirical antibiotic given was Fortum/ amikacin, which was changed to Tazobactum/amikacin from April 2003. In only 4 episodes second line antibiotics were used. Antifungal (ampho-B) were used in 2 episodes. All patients recovered without any significant morbidity. There was no fatal episode, and none of the patient required admission to ICU.
*Conclusions:* There is high incidence of febrile neutropenia in children who receive chemotherapy for sarcomas. But all the incidences can be safely managed by using a standard protocol without any serious consequences.

### P100

**Needle biopsy vs. open biopsy: Is it useful in soft tissue and bone tumors?**

E. Moessinger[1], F. Laenger[2], E. Hesse[1], L. Bastian[1]
*([1]Klinik für Unfallchirurgie, Medizinische Hochschule Hannover, Hannover, Germany, [2]Pathologisches Institut, Medizinische Hochschule Hannover, Hannover, Germany)*

The aim of this retrospective study was to evaluate the results of pathological findings in patients with soft tissue and bone tumors, who were first treated with needle biopsy.

Between January 2002 and December 2003. 100 patients underwent biopsy in our clinic because of soft tissue or bone tumors.

56 bone tumors, including secondary bone tumors, and 44 soft tissue tumors were treated with biopsy during their diagnostic algorhythm. Needle biopsy (NB) was performed in 70 patients and open biopsy (OB) was performed in 30 patients. The NB group was treated in our outpatient clinic and was operated under local anaesthesia while the OB-group was admitted in our clinic for at least 2 days. There was no significant difference in the results of pathological findings between the NB an OB groups.

Especially in soft tissue tumors, the needle biopsy has become an alternative treatment in the diagnostic algorhythm.

### P101

**It could be difficult to remain blind to molecular diagnosis of EWS-FLI 1 gene rearrangement during the treatment of Ewing Sarcoma/PNETs**

P. Mudry[1], J. Sterba[1], V. Bajciova[1], J. Berkovcova[2], J. Skotakova[3], P. Gal[4], P. Janicek[5]
*([1]Department of Pediatric Oncology, Children's Hospital Brno, Czech Republic, [2]Department of Internal Medicine - Haematooncology, University Hospital Brno, Czech Republic, [3]Department of Pediatric Radiology, Children's Hospital Brno, Czech Republic, [4]Department of Pediatric Surgery, Traumatology and Orthopedics, Children's Hospital Brno, Czech Republic, [5]Department of Orthopedics, St Anna Hospital, Brno, Czech Republic)*

Evaluation for the presence of Ewing sarcoma/PNET (ES/PNET) cells using RT-PCR for EWS-FLI 1 fusion products is possible, however the clinical utility of such studies is still matter of controversy and ongoing randomized clinical trials. We believe, that there are some clinical situations, where the patient could benefit from such information. As an example of positive role of molecular diagnosis we present two ES/PNET patients with complicated clinical course and another two patients with their molecular relapse detection from peripheral blood prior diagnostic imagines. We can document feasibility of EWS-FLI 1 detection in all our specimens – tumor tissue, peripheral blood, bone marrow, all suspicious tissues biopsies, incl. CSF and stem cells. PCR methods could be used for molecular relapse detection, and both confirmation of negativity of surgical specimens, or revealing of positivity in the tissue sample, or minimal residual disease (MRD) detection could be useful for clinicians. Despite the prognostic role of such testing remains to be established, for some patients (e.g. poorly tolerating standard treatments) the use of PCR results could be of benefit to while considering experimental therapies like targeted or antiangiogenic maintenance therapy with acceptable toxicity profile to retain remission and/or quality of life.

### P102

**Plastic-surgical management of patients with sarcoma of the forearm and hand**

M. Müller, G. Germann, B. Bickert, M. Sauerbier
*(Department of Hand-, Plastic and Reconstructive Surgery - Burn Center -, BG Trauma Center Ludwigshafen, Plastic and Hand Surgery of the University of Heidelberg, Germany)*

Radical tumor resection is main therapeutic goal for treatment of sarcomas of the forearm and hand. Reconstructive procedures play a key role in coverage of defects and prevention of problems due to wound infection. If localized within the periphery of the upper limb the vast majority of cases require sophisticated techniques to achieve salvage of forearm and hand.

Besides local cutaneous, muscle and fascia – flaps, pedicled and free microvascular transplantations are used. In special instances ablative surgery may become necessary. The purpose of this study was to evaluate the outcomes of patients who underwent radical resection of sarcomas about forearm/hand. Between 1995 and 2005 15 sarcoma resections were performed. 9 patients were male, 6 female, average age was 52 years. After biopsy 7 fibrous histiocytomas, 2 leiomyosarcomas, 2 synovial cell carcinomas, 2 osteosarcomas, 1 epitheloid sarcoma, 1 chondrosarcoma were diagnosed. 2 patients were treated with primary closure or mesh graft transplantation. 1 patient received a local flap, 6 patients needed free microvascular transplantations to cover the defect. It was impossible to preserve the limb 6 times, 3 patients had tumor recurrence. Our results show the necessity of plastic-surgical reconstruction as an integrative component of modern sarcoma therapy.

### P103

**Results of Spanish Cooperative Protocol SEOP-95 for non metastatic osteosarcoma of the limbs in children**

A. Muñoz, M.S. Maldonado, J. Alfaro, T. Contra, P. Garcia-Miguel, M.J. Antuña, A. Cantalejo, N. Pardo, C. Melero, L. Gros, J. Heras, J. Huguetet, M. Ruiz-Portal, G. Ocete
*(Spanish Society of Pediatric Oncology (SEOP), Servicio de Pediatria. Hospital Ramon y Cajal, Madrid, Spain)*

*Objectives:* To improve survival and the rate of limb salvage procedures, we started a protocol in which from 1995 to 2000, 99 patients with non metastatic osteosarcoma of the limbs were enrolled.
*Methods:* The protocol consisted of preoperative chemotherapy with ifosfamide doxorubicin, cisplatin and high dose methotrexate. After surgery patients received postoperative chemotherapy with the same drugs.
*Results:* Median age was 12 years. Femur (50%) was the most common primary site. Limb salvage procedures were performed in 85% of patients. Good histologic response was observed in 67 cases. There were 7 local relapses (7% ) and 4 toxicity-related deaths. One patient developed a second neoplasia (AML). No significant sequelae were observed. With a follow up of 42 to 118 months (median 83 months) the 5 years event-free-survival is 67%.Survival for good histologic response patients was 73% versus 52% for poor responders ($P = 0.011$).Survival for patients with tumor volume <100 ml was 80% versus 68% for the rest ($P = 0.12$).
*Conclusions:* This protocol led to good oncologic results, with a significant toxicity rate. Our current protocol SEOP-2001 has shortened the post-op chemotherapy in order to reduce treatment-related toxicity.

### P104

**Spanish cooperative study SEOP-EW 95 for the treatment of children with Ewing sarcoma and primitive neuroectodermal tumors of bone**

P. Garcia-Miguel, A. Sastre, A. Alfaro, M.J. Antuña, A.. Cantalejo, T. Contra, J.V. Hernandez, A. Muñoz, N. Pardo, M.J. Torres, M. Ruiz-Portal, J. Heras, G. Ocete, J. Huguet, A. Santos, M.S. Maldonado
*(Spanish Society of Pediatric Oncology (SEOP), Hospital Infantil La Paz, Madrid, Spain)*

*Purpose:* To improve survival of children with Ewing sarcoma and PNET.
*Methods:* From 1995 to 2001, 132 patients were included. Non axial tumors received preoperative and postoperative chemotherapy with Vincristine, Adriamycin, Ifosfamide and Actinomycin if tumor volume <100 ml, plus etoposide for >100 ml tumors. Axial tumors received same chemotherapy, irradiation and/or surgery for primary and high-dose Busulfan–Melphalan. For metastatic patients treatment was as above plus irradiation of metastases.

*Results:* There were114 Ewing and 18 PNET: Median age was 10.5 years; 51% of patients had pelvic or metastatic/multicentric tumors. EFS at 63 months is 56%. Pelvic primary tumors had a EFS of 17% versus 63% for the rest ($P = 0.001$). Patients with good response to preoperative chemotherapy had an EFS of 79% versus 49% for the rest ($P = 0.002$). Patients with lung metastases had an EFS of 63% versus a 17% for multicentric ($P = 0.001$). There were 2 secondary neoplasias (1 ALL and 1 AML).
*Conclusions:* In our series, with an elevated rate of high risk patients, this protocol was found to be effective in disease control and survival.

### P107

**"SOS Desmoide" A French patient group for desmoid tumor promoting research, supporting patients**

M. Podevin[1], L. Mignot[2], M. Lemarchand[1], E. Taugourdeau[1], P.H. Cottu[3], O. Oberlin[4]
*([1]SOS Desmoide Association, [2]Hopital Foch, Suresnes, France, [3]Clinique des diaconesses, Paris, France, [4]Hopital Pitié-Salpétrière, Paris, France, [5]Institut Gustave Roussy, Villejuif, France)*

"SOS desmoide" is an association created in 1998 by a patient in partnership with a doctor with several aims:

- To break the loneliness of the patients and their family.
- To enhance exchange networks between patients, between doctors, between patients and doctors, and between doctors and researchers.
- To sort and synthesize existing publications.
- To promote research.

Since that date, the association has grown up and has now 250 members of whom 60 are patients – adults and children.

"SOS desmoide" has set up a website for the patients and the doctors (http://www.sos-desmoide.asso.fr/).

It has edited information booklets for patients and doctors and is editing a newspaper.

It has launched a tumor bank to collect tumor samples that will be used for research, with the strong wish to remain in close contact with the researchers.

It has set up supportive group discussions.

The association is in relationship with many patients and doctors from France and are trying to promote a network of doctors in France who would join their efforts to increase their expertise on this rare disease.

As the association had also contact with patients from the entire world, it would like to make itself known and to promote similar associations in other countries.

### P108

**Desmoid tumors in childhood: Place of radiation therapy and chemotherapy**

C. Oudot[1], P. Marie[2], D. Orbach[3], G. Raimondo[1], M.J. Terrier Lacombe[1], O. Oberlin[1]
*([1]Institut Gustave Roussy, Villejuif, France, [2]Hôpital Trousseau, Paris, France, [3]Institut Curie, Paris, France)*

*Purpose:* Retrospective study to define the efficacy and places of surgery, radiation radio therapy and chemotherapy.
*Patients:* Between 1982 and 2003, 71 children were treated in 3 French hospitals.
*Results:* There was a male predominance (41 boys/30 girls). Median age at diagnosis was 6.7 years [0–16]. Localizations were various: head and neck (32%), limbs (30%). Only 3 had intra-abdominal localization (4%). Three cases are associated with Gardner's Syndrome.

The first line of treatment was surgery in 56/71 cases (79%) and chemotherapy in 10 cases (14%) because of unresectable lesions. When surgery was performed first, free margins were described in only 8.5%.

Local radiotherapy was given in 22 cases after surgical resection. Persistent complete response has been obtained in 12 cases (55%).

36 children received a total of 60 various chemotherapy lines (either vinblastin + methotrexate, or actinomycin based regimens) at diagnosis or for relapse. Complete and partial responses was obtained after 21/60 (35%). 8 patients have presented a spontaneous regression.
*Conclusions:* Despite heterogeneous treatments, encouraging results have been observed after chemotherapy or radiation therapy. Prospective studies according to European recommendations will give us additional data on therapy and epidemiology of this rare disease.

### P109

**Ewing's sarcoma of the pelvis: 62 patients of the French Society of Pediatric Oncology (SFOP)**

E. Mascard[2], P. Wicart[2], C. Carrie[1], J. Dubousset[2], O. Oberlin[2]
*([1]Hospital Saint Vincent de Paul, Paris, France, [2]Institut Gustave Roussy, Villejuif, France)*

We aimed to evaluate the role of surgery and/or radiation therapy in pelvic Ewing's sarcoma.

Sixty-two patients were treated, from 1984 to 1993, according to three successive chemotherapy protocols of the SFOP. There were 35 females and 27 males, aged 5 to 28 years. Sixteen patients had metastases at diagnosis. The modality of local treatment was not randomized. Surgery was performed alone in 18 cases, surgery plus radiation therapy in 15 cases, and radiation therapy alone in 25 cases. Four patients had no local treatment. High-dose chemotherapy with stem cell transplantation was used in 14 patients.

The results were retrospectively assessed at mean 6.6 years follow-up: 29 patients had no evidence of disease, 6 had an evolutive disease, and 27 were deceased. Two patients developed a radio-induced sarcoma. Overall survival was $55 \pm 6\%$ at five years FU and $53 \pm 7\%$ at 10 year FU. There was no significant difference in survival or local control related to the chemotherapy protocols. There was a significant improvement in overall survival (68% vs. 43%, $p = 0.007$) after surgery compared with radiation therapy alone. Only two patients with metastases at diagnosis had no evidence of disease at last F.U.

There was a significant improvement in pelvic Ewing sarcoma outcome, with chemotherapy, and surgical treatment when feasible. The prognosis of such lesions was, in our experience, not so worse from other locations.

### P110

**Alveolar soft part sarcoma: A difficult and rare tumour in oncology**

J. Vermeulen, P. Freneaux, S. Helfre, V. Laurence, F. Doz, H. Pacquement, J. Michon, D. Orbach
*(Institut Curie, Paris, France)*

*Aim:* To retrospectively review the presentation and outcome of 5 patients treated between 1990 and 2004 for alveolar soft part sarcoma (ASPS) in our institution.
*Patients:* Median age was 9 years [6–36]. Primary was limbs (3 pts), orbital (1 pt) or non parameningeal head and neck

(1 pt). Histological diagnosis was initially wrong in 4 pts. 2/2 tumor had a TFE3 expression and TFE3/ASPL transcript was present in 1/1 case.

First treatment was inadequate surgery (4 pts) or biopsy (1 pt). IRS groups distribution was: IRS-I 1 pt, IRS-II 2 pts, IRS-III 1 pt, IRS-IV 1 pt (pulmonary). Complete remission (CR) was achieved with surgery alone 1 pt, surgery with chemotherapy and radiotherapy 2 pts. The 2 children with evaluable tumor were progressive after chemotherapy. One child was lost of follow up (FU) during treatment with progressive disease. One child is in late second CR after a surgery of a distant solitary pulmonary metastasis. At end of FU, 3 children are in CR (at 2 months–3 and 9 years).

*Conclusions:* 1. ASPS is a very rare tumor; 2. Local therapy should be maximum; 3. Histological diagnosis is difficult and may be helped by the TFE3 nuclear expression and the presence of TFE3/ASPL fusion transcript. 4. Long term metastasis may be curable with surgery only.

**P111**

**Role of MLL4 in sarcomagenesis**

M.J. O'sullivan, N. Melnyk, P.H.B. Sorensen
*(University of British Columbia, Vancouver, BC, Canada)*

We identified a novel non-constitutional chromosomal translocation, t(17;19)(p13;q13), occurring as solitary karyotypic abnormality in an intracranial, extra-axial undifferentiated sarcoma presenting in a 12-year-old girl. Two-colour FISH analysis using overlapping BACs, showed localization of the 19q13.12 breakpoint to the region harbouring the MLL4 gene.

This is the first tumor showing translocation involving MLL4, and only sarcoma with a translocation involving an MLL gene family member. Although there has been no prior report of involvement of the MLL4 gene in chromosomal translocation, the closely related MLL gene is frequently involved in translocations in leukemias, with adverse prognostic implications.

Aberrations of 19q13, the region harboring MLL4, are common in undifferentiated sarcomas and osteosarcomas. FISH analysis of an additional 47 cases of undifferentiated sarcomas and osteosarcomas obtained from CHTN using overlapping BACs showed only fused signals, suggesting that none of these cases bears a translocation involving MLL4. Ten cases showed amplification of MLL4, 17 showed 2–4 signals, possibly indicating duplication or polyploidy. The remaining 10 cases were normal. This is provocative in light of the identification of partial tandem duplication of MLL in many hematopoietic neoplasms lacking translocation of MLL.

We hypothesise that MLL4 plays a role in sarcomagenesis, analogous to the role of MLL in leukemogenesis.

**P112**

**Neoadjuvant chemotherapy for osteogenic sarcoma: Results of a single institute from Turkey**

A. Ozkan, T. Celkan, H. Apak, S. Ocak, M. Hız, S. Dervişoglu, F. Dincbas, S. Karaman, I. Yildiz
*(Department of Pediatric Oncology, Cerrahpasa Medical Faculty, University of Istanbul, Turkey)*

*Purpose:* The aim of this study is to describe the outcome of neoadjuvant chemotherapy for osteogenic sarcoma.

*Patients and methods:* Between November 1990 and November 2004, 27 patients with osteosarcoma were treated according to a protocol of neoadjuvant chemotherapy with Cooperative Osteosarcoma Study Group (COSS) or combination of cisplatin and doxorubicin. The median age at diagnosis was 12 years (range, 5–16 years). A total of 25 (92.6%) primary tumors were located in an extremity, whereas 2 (7.4%) occurred in the axial skeleton. Surgery was limb salvage in 17 patients and amputation in 3 patients. The proportion showing a good histopathological response (>90% tumour necrosis) to preoperative chemotherapy was about 55%.

*Results/discussion:* The 5-year overall survival was significantly related to the degree of histologic response to preoperative chemotherapy (81.82% for good responders versus 55.56% for poor responders). 20 (74%) patients had localized disease, while 7 (26%) had metastases at diagnosis. In 6 patients (22%), no surgical treatment was performed. Thirteen patients (48.1%) received radiotherapy (3 patients for tumor control of unresectable primaries).The mean follow-up of all patients was 75 months (range 2–117 months). The 5-year event-free survival (EFS) and overall survival (OS) rates were 52.58 and 57.52% respectively.

**P113**

**All-trans retinoic acid, interferon-α and zoledronic acid in the treatment of a patient with resistant metastatic osteosarcoma**

A. Ozkan
*(Department Of Pediatric Oncology, Cerrahpasa Medical Faculty, University of Istanbul, Turkey)*

*Purpose:* Alternative approach in the treatment of patients with resistant metastatic osteosarcoma.

*Patient and methods:* The patient, a girl 14 years of age, was first admitted to the hospital for right knee and left jaw pain and mass. A high grade osteoblastic osteogenic sarcoma of the right distal femur and left mandibula' was diagnosed after an excisional biopsy. Bilateral micronodular lung metastases were present at diagnosis.

The patient was initially treated according to the Cooperative Osteosarcoma Study Group (COSS 96) protocol for osteogenic sarcoma. We detected tumour progression on the 10th and 18th weeks of protocol. Despite the administration of second line (high dose ifosfamide + etoposide $\times$ 2) and third line (doxorubicin + cisplatin $\times$ 2) of chemotherapy, combined local radiotherapy, which couldn't achieve the control of the tumour. The patient was then treated with a combination of oral ATRA (90 mg/m$^2$ for 3 days per week), subcutaneous IFN$\alpha$ (3 $\times$ 106 U/m$^2$ 5 days per week), intravenous infusion zoledronic acid (4 mg per months) and CAM (complementary and alternative medicine: diet, vitamine, melatonin, antioxidan therapy, mistletoe, green tea, curcuma). Treatment was well tolerated, and the patient is in stable disease for 8 months.

*Results/discussion:* ATRA/interferon $\alpha$/zoledronic acid treatment may be considered as an alternative approach in the treatment of patients with metastatic osteosarcoma who have disease that is resistant to conventional chemotherapy

**P114**

**Ewing's sarcoma: Results of a single institute from Turkey**

A. Ozkan, T. Celkan, H. Apak, S. Ocak, M. Hız, S. Dervişoglu, A. Canpolat, S. Karaman, I. Yildiz
*(Department of Pediatric Oncology, Cerrahpasa Medical Faculty, University of Istanbul, Turkey)*

*Purpose:* The aim of this study is to describe the outcome of therapy for Ewing's sarcoma.

*Patients and methods:* Data on 25 patients with Ewing's sarcoma of bone treated at a single institution between January 1983 and January 2003 were retrospectively considered (median age, 9.0 years; range, 3.0–15.0 years). All patients received primary treatment with chemotherapy and surgery and or radiotherapy as local modality treatment.

*Results/discussion:* At diagnosis, 16 patients had local-regional disease, and 9 had distant metastases. The mean follow-up of all patients was 50 months (range 4–108 months). The 4-year event-free survival (EFS) and overall survival (OS) rates were 24.64 and 24.89% respectively.

The 4-year overall survival (OS) rate for patients with local-regional disease is 42.86%; the 4-year OS rate for patients with distant metastases is 0%. All patients with distant metastases had progressive disease during therapy, and 9 patients with local-regional disease experienced disease progression during therapy. Despite significant progress with the use of intensive multiagent chemotherapy and local control measures, a significant proportion of our patients die of disease progression.

### P115

**Malignant degeneration to osteosarcoma in a patient with heterotopic ossiffication**

C. Pacheco, M.D. Albalá, M. Torres, A. Benítez, M.C. Pérez, J. García, J.M. Latre
*(Department of Nuclear Medicine, Reina Sofía Hospital, Córdoba, Spain)*

Heterotopic ossification (HO) is an extra-osseous non neoplastic growth of new bone. The clinical impact of HO depends on the clinical setting, location and extent of disease. The treatment is generally conservative. Malignant degeneration to osteosarcoma has been reported but is extremely rare.

We present a 56 years old woman with an HO at the right calf since November-1990. That lesion presented a rapid enlargement and significant pain during last months.

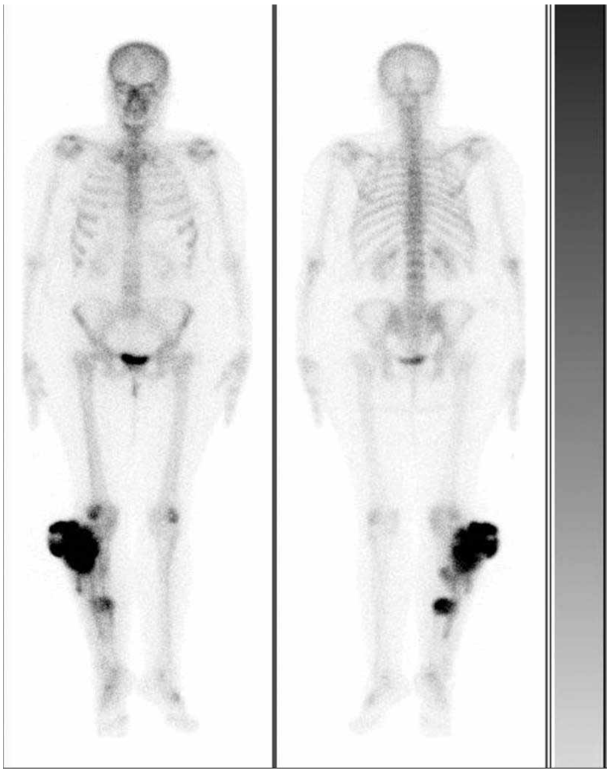

X-rays planar showed an increase in soft tissue mass respect to the previous. MRI demonstrated a large image with heterogeneous high T1 and T2 signal and heterogeneous gadolinium enhancement.

Three-phases-bone-scan showed an increased activity in soft tissues, due to hyperemia and ossification. Delayed scan showed increase uptake of affected adjacent bones, without another pathologic uptakes justified by metastases.

The final diagnostic confirmed by biopsy was well-differenced osteosarcoma with rapid growth of its cartilaginous component, and the patient was put to amputation of the leg with definitive treatment.

The contribution of the bone-scan in this case resides in the visualization of the lesions' hyperemia in the early phases, the demonstration of the bone involvement in delayed images and the importance of discarding bone metastases in one unique study.

### P116

**No increased cancer risks in relatives of children with Ewing's sarcoma: A population-based family study**

D. Pang, R. Alston, A. Kelsey, J.M. Birch
*(University of Manchester & Cancer Research UK, Manchester, UK)*

*Purpose:* To determine whether there are increased risks of cancer among relatives of children with Ewing's sarcoma, to assess the possible role of cancer predisposition.

*Patients and methods:* All children under 15 years old included in the Manchester Children Tumour Registry between 1 January 1954 and 31 December 2001 with histologically confirmed Ewing's sarcomas were eligible. Families of cases were interviewed. Reported cancers in relatives were verified from medical records. Standardised incidence ratios (SIR) were estimated from the age, sex, and calendar year specific national cancer incidence rates. *P*-values were calculated assuming occurrence of cancer follows a Poisson distribution.

*Results:* The study has sufficient statistical power (99%) to detect a 2 fold increased risk. There is no overall cancer excess in relatives of cases ($O = 65$, SIR=0.8, $p > 0.05$). There are no discernible excesses in any specific common cancers in relatives, including breast, CNS, lung, colorectal, stomach, cervical, melanoma, prostate and bladder (all SIRs <1.5, all *P*-values >0.05).

*Conclusion:* The study does not support a general role of cancer predisposition in Ewing's families. However the results do not rule out a genetic role in certain rare families. Further studies on subgroups of Ewing's incorporating molecular analyses would be helpful to clarify this.

### P117

**Correlation between histology and PAX/FKHR fusion status in alveolar rhabdomyosarcoma**

D.M. Parham[1], S. Qualman[2], L. Teot[3], F.G. Barr[4], W.H. Meyer[5]
*(Soft Tissue Sarcoma Committee of the Children's Oncology Group: [1]Departments of Pathology and Pediatrics, Arkansas Children's Hospital and Arkansas for Medical Sciences, Little Rock, AR, USA, [2]Children's Hospital, Columbus, OH, USA, [3]Children's Hospital of Pittsburgh, Pittsburgh, PA, USA, [4]University of Pennsylvania, Philadelphia, PA, USA, [5]Children's Hospital of Oklahoma, Oklahoma City, OK, USA)*

At the molecular level, alveolar rhabdomyosarcoma (ARMS) is characterized by three mutually exclusive PAX/FKHR conditions: PAX3/FKHR fusion (present in 65% of cases), PAX7/FKHR fusion (present in 20%), and PAX/FKHR fusion negativity (present in 15%). The possibility of morphologic variation among these molecular subtypes has not been investigated. We undertook a blinded retrospective study of 65 cases of ARMS (16 PAX fusion negative, 36 PAX3/FKHR positive, and 13 PAX7/FKHR by routine RT-PCR). We evaluated cytohistologic parameters such as microcyst formation, solid foci, differentiation, giant cell formation, anaplasia, nuclear grade, mitotis/karyorrhexis index, rosette formation, geographic necrosis, stroma production, and the presence of foci resembling embryonal rhabdomyosarcoma. We analysed the results using a simple chi-square formula. Of these features, only lack of cyst formation reached significance ($p = 0.00014$), with 7 of 16 PAX negative cases lacking this feature, compared to 0 of 36 PAX3/FKHR cases and 2/13 PAX7/FKHR cases. More investigation is indicated, but these preliminary results indicate that in general, only lack of cyst formation in ARMS would suggest the absence of a PAX fusion.

### P118

**A comparison of epirubicin- and doxorubicin-induced cardiomyopathy in paediatric soft-tissue sarcoma patients**

M. Paulides[1], W. Stöhr[1], I.B. Brecht[2], A. Kremers[1], J. Treuner[2], E. Koscielniak[2], T. Langer[1], J.D. Beck[1]
*([1]LESS Centre, University Hospital for Children and Adolescents, Department of Paediatric Oncology, Erlangen, Germany, [2]Cooperative Soft Tissue Sarcoma Study, Olgahospital, Department of Paediatric Oncology, Stuttgart, Germany)*

*Background:* Epirubicin-induced cardiomyopathy has been studied almost exclusively in adult cancer patients. The aim of our study was to investigate epirubicin-induced cardiotoxicity in comparison with doxorubicin in children and adolescents.
*Patients and methods:* 172 soft tissue sarcoma patients (mean age at diagnosis: 8.3 years), treated with epirubicin (median cumulative dose: 450 mg/m$^2$) or doxorubicin (median cumulative dose: 240 mg/m$^2$) within the high risk group of the CWS-96 study, were examined in a prospective multicentre study. Heart function was analysed by echocardiography, measuring left-ventricular fractional shortening. The median follow-up was 27.7 months.
*Results:* The cumulative incidence of clinically manifest cardiomyopathy was 0% (0/60; 95%-CI: 0–6.0%) in patients treated with epirubicin, and 0.9% (1/108; 95%-CI: 0–5.1%) in patients treated with doxorubicin. Three patients showed subclinical cardiomyopathy. There was no difference in fractional shortening between the two treatment arms.
*Conclusion:* Cardiotoxicity was low in this study. For the short term, cardiotoxicity seems to be only a small problem in epirubicin-treated patients, if applied as in this study.

### P119

**Ewing tumor with extrapulmonary metastases: Changes in survival between 1981 and 1999. Data from the GPOH (EI)CESS Group**

M. Paulussen, N. Weddeling, C. Liebscher, A. Hunold, D. Manner, H. Juergens
*(GPOH (EI)CESS Group, Dept of Pediatric Hematology/Oncology, University Children's Hospital, Muenster, Germany)*

*Background:* Outcome of patients with extrapulmonary metastatic Ewing tumor is commonly regarded fatal. Here, outcome of patients registered to three consecutive co-operative GPOH Ewing tumor treatment studies is analyzed.
*Patients:* Patients were treated within CESS81 ($n = 20$), CESS86 ($n = 62$), and EICESS92 ($n = 164$). Prescribed treatment consisted of alkylator- and doxorubicin based chemotherapy with local therapy of the primary tumor and of metastases, where possible. In EICESS92, some patients received high-dose therapy with stem cell rescue in addition.
*Results:* Four-year survival was 0.10 in CESS81, 0.31 in CESS86, and 0.32 in EICESS92. The improvement from CESS81 to CESS86 was significant ($p = 0.0152$). There was no significant further improvement from CESS86 to EICESS92.
*Conclusions:* Since CESS81, the prognosis of patients with extrapulmonary metastatic Ewing tumor has increased to more than 25% of patients surviving. This outcome, however, is still unsatisfactory and new treatment strategies are warranted in this patient group.

### P120

**Epitheloid sarcoma: Single institution experience with focus on chemotherapy**

D. Pink, P.-U. Tunn, T. Lindner, P.C. Thuss-Patience, A. Kretzschmar, P. Reichardt
*(Department of Hematology and Oncology, Charité Campus Buch, HELIOS-Klinikum Berlin-Buch, Berlin, Germany)*

*Background:* Epitheloid sarcoma (ES) is a very rare tumor of children and young adults.
*Results:* No publications on chemotherapy of ES beyond case reports were identified in PubMed. From our registry of 830 patients, we identified 18 patients with ES (2 female, 16 male). Median age at primary diagnosis was 31 years (range 18–56) with 9/18 patients under 30 years. 6/17 patients developed a local recurrence, 13/17 metastases. 4 patients had metastases at primary diagnosis. Metastases occurred in lymph nodes (8/13), lungs (11/13), skin (5/13), bone (2/13), muscles (1/13) and brain (1/13). 9 patients were treated with first-line chemotherapy in advanced disease: 6/9 Epi/Ifo (3xSD, 3xPD), 1/8 Doxo/Ifo (1xSD), 2/8 Doxo mono (2xSD). 2nd-line chemotherapy was used in 5 patients: 2/5 patients HD-Ifo (1xSD, 1xPD), 1/5 Doxo/DTIC (1xPD), 2/5 Gem/Doce (1xPR, too early). 3rd-line-therapy: 1/2 Gem/Cis (1xSD), 1/2 Gem/Doce (1xSD). 14/17 patients are alive at a median follow-up of 19 months.
*Conclusions:* Standard chemotherapy seems to be ineffective (no responses to doxorubicin-containing therapies in 9 patients, no responses in 7 patients treated with high-dose ifosfamide). In contrast, gemcitabine-containing therapies showed promising activity.

### P121

**Chemotherapy in patients with high-grade or dedifferentiated chondrosarcomas: Single institution experience**

D. Pink, P.-U. Tunn, T. Lindner, P.C. Thuss-Patience, A. Kretzschmar, P. Reichardt
*(Department of Hematology and Oncology, Charité Campus Buch, HELIOS-Klinikum Berlin-Buch, Berlin, Germany)*

*Background:* Chondrosarcoma (CS) is a rare neoplasm, but 2nd most common malignant bone tumor (10–20% of cases). 40% of CS are low-grade ("1/2", 1), 60% high-grade (2, 3, dediff. CS) [dedifferentiated CS: 10%]. The 5-year OS for low-grade CS is 90%, for high-grade (2, 3) 40–50% and for dedifferentiated CS 10–20%. Data on chemotherapy in metastatic or unresectable CS are rare.
*Results:* Only 11 publications containing original data on chemotherapy in CS were identified in PubMed. Some responses to chemotherapies including ifosfamide, doxorubicin, and cisplatin were reported. In dedifferentiated CS chemotherapy seemed to improve the prognosis of the patients. In our institution 19 patients with CS were treated with chemotherapy. 4/19 patients responded to chemotherapy (CR and PR; 20% RR). Most patients were treated with high-dose ifosfamide, antracyclines and cisplatin. 7/19 are alive; median OS was 36 months from first-diagnosis (5+ to 125). 17 patients had metastatic disease, 5 are alive; median OS was 20 months from diagnosis of metastatic disease (3+ to 91).
*Conclusions:* In high-grade and dedifferentiated CS chemotherapy with HD ifosfamide and antracyclines or cisplatin and antracyclines seems to be effective with response rates comparable to soft tissue sarcomas. A prospective multicenter trial should be performed.

**P122**

**Soft tissue sarcomas in children: 12 years data from a single pediatric oncology center in Poland**

K. Połczyńska[1], J. Stefanowicz[1], D. Sierota[1], E. Drożyńska[1], E. Bień[1], B. Kaczorowska-Hać[1], T. Stachowicz-Stencel[1], A. Szołkiewicz[1], M. Hennig[1], A. Balcerska[1], P. Czauderna[2], L. Komasara[2], E. Iżycka-Świeszewska[3]
*([1]Department of Pediatrics, Hematology, Oncology and Endocrinology, [2]Department of Pediatric Surgery, [3]Department of Pathomorphology, University of Gdańsk, Poland)*

588 children with malignancies were treated in Department of Pediatric, Hematology, Oncology and Endocrinology in Gdańsk between 1992 and 2004. In that group 62 (10.5%) patients suffered from soft tissue sarcomas. The aim of the study was to describe tumour histology, localization and clinical stage, treatment protocols and results of therapy.

RMS was diagnosed in 33, non-RMS in 26 and RMS-like tumours in 5 children. There were 35 girls and 27 boys in age between 3 months and 23 years at the diagnosis.

Favourable tumour localization was found in 17, unfavourable in 45 cases. Most of patients were in III and IV (55%, 18%) clinical stage of disease.

Children were treated according to CWS-91, 96, 2002 and other (IRS) protocols.

R0 primary tumour resection was performed in 7, R1 in 15 and R2 in 40 patients (11%, 24%, 65%). Relapse was observed in 19 (31%) patients. 27 children (44%) died between 2 and 60 months from diagnosis – 3 of them due to sepsis.

In 9 patients (16%) permanent consequences of treatment are noticed. The mutilating surgery was done in 8 children.

Results of soft tissue sarcomas treatment are still not satisfactory. Significantly high percentage of cases with advanced stage of disease was found.

**P123**

**Soft tissue sarcomas located in extremities: Review of 13 cases**

K. Połczyńska[1], J. Stefanowicz[1], D. Sierota[1], E. Drożyńska[1], E. Bień[1], T. Stachowicz-Stencel[1], A. Szołkiewicz[1], A. Balcerska[1], P. Czauderna[2], L. Komasara[2], E. Iżycka-Świeszewska[3], M. Dubaniewicz[4]
*([1]Department of Pediatrics, Hematology, Oncology & Endocrinology, University of Gdańsk, Poland, [2]Department of Pediatric Surgery, Gdańsk, Poland, [3]Department of Pathomorphology, Gdańsk, Poland, [4]Department of Radiology, Gdańsk, Poland)*

*Introduction:* Sarcomas having seats in extremities are associated with a worse prognosis. This opinion caused us to review results of therapy in patients treated in our department between 1992 and 2004.
*Objective:* To describe clinical presentation, used therapy and outcome.
*Patients:* In 13 of 62 children with sarcomas tumour was located in extremities (21%). In this group were 6 girls and 7 boys in age from 3 months to 12 years.

RMS were recognized in 8 children (RMA–5, RME–3), non-RMS in 3 (MFH–2, TT–1), and RMS-like in 1 child. Advanced stages of disease were noted in 62% patients (III–4, IV–4). The treatment was carried according to CWS-91, 96, 2002 and other (IRS) protocols.
*Results:* 7 children are alive, 5 in complete remission with time of observation 3–10 years from the end of therapy. 6 patients died (46%): 5 of disease's progression. 1 child died of treatment complications – sepsis. Relapse was observed in 8 children. Among them 2 are in complete remission for more than 5 years, 2 are still treated and 4 patient died during 7–35 months from diagnosis.

**P124**

**Soft tissue sarcomas in children with neurofibromatosis type 1: Four cases treated in a single oncological institution**

E. Bien,[1] A. Szolkiewicz[1], T. Stachowicz-Stencel[1], K. Polczynska[1], D. Sierota[1], E. Drozynska[1], G. Gulida[2], P. Czauderna[3], W. Kosiak[4], A. Balcerska[1]
*([1]Department of Paediatrics, Haematology, Oncology and Endocrinology, Medical University of Gdansk, Poland, [2]Department of Anatomopathology, Medical University of Gdansk, Poland, [3]Department of Paediatric Surgery, Medical University of Gdansk, Poland, [4]Department of Paediatric Nephrology, Medical University of Gdansk, Poland)*

Patients with neurofibromatosis type 1 (NF1) are predisposed to developing malignant neoplasms, including soft tissue sarcomas (STS), mainly of neural origin. Herein we report on 4 cases of STS occurring in children with NF1, treated in a single oncological institution. Material and methods: F/M: 3/1, age: 8mo–14years. Histopathology: RME-2, MPNST-1, MTT-1. Primary tumour was localized in minor pelvis in 3/4 children and left forearm in 1. All patients presented with advanced, unresectable tumours and received CWS-91, CWS-96 or CWS-2002 protocols for STS.

Both patients with RME entered CR. A girl with forearm tumour developed lung metastases 1.5 year post-therapy and died of progression despite complete excision and magachemotherapy with autologous PBSCT. 8-month-old boy with bladder RME died in CR of neutropenia-related sepsis. Patients with MPNST and MTT of minor pelvis did not respond to therapy. 14-year-old girl with huge unresectable tumour died of progression. Another patient underwent mutilating MTT resection with uterus and bladder. She is disease-free 6 years post-therapy – the only one among presented patients.
*Conclusions:* Neural and non-neural STS in NF1 children seem to display worse prognosis than appearing as sporadic cases. Children with NF1 should remain under detailed control of oncologist.

**P126**

**Clinical characteristics of sarcomas of head and neck in children**

S. Postovsky[1], M. Weyl Ben Arush[1], D. Fliss[3], L. Ardikian[2]
*([1]Pediatric Hematology Oncology Department, [2]Maxillofacial Surgery Department, Rambam Medical Center, Haifa, Israel,*

[3]*Otolaryngology Department, Sourasky Medical Center, Tel Aviv, Israel)*

*Background:* Limited information is available about clinical behavior and management of pediatric head and neck sarcomas (PHNS).
*Aim of study:* To characterize presentation, treatment and outcome of PHNS.
*Patients:* Fifteen children, 6 boys and 9 girls, median age 9.6 years (range, 3 months–17 years) were evaluated. Diagnoses were as follows: Rhabdomyosarcoma – 5 patients (pts), osteosarcoma – 3 pts, Ewing's sarcoma – 2 pts, mesenchymal chordoma, rhabdoid tumor, malignant peripheral nerve sheath tumor, aggressive fibromatosis – one pt each.
*Results:* Swelling was presenting sign in 9 pts. Other symptoms were related to pressure, obstruction or bleeding. The mean duration of symptoms before diagnosis was 6 months (range, 4 days–18 months). The tumor involved adjacent structures in 6 of 15 pts. There were no metastases at diagnosis. Treatment consisted of surgery (S) – 2 pts, S + radiotherapy (RT) – 1 pt, S + chemotherapy (CT) – 1 pt, CT + RT + S – 11 pts. With median FU of 38 months, 3 pts – DOD, 10 pts – NED, two pts – AWD. Three pts developed distant metastases (two – died).
*Conclusions:* PHNS are characterized by relatively low risk for developing metastatic disease (20%). In 40% of pts, there was extension to adjacent structures. The completeness of surgical resection of PHNS is the main prognostic factor.

### P127

**Survival of metastatic disease in children with bone sarcomas and soft tissue sarcomas**

S. Postovsky, B. Moaed, R. Elhasid, A. Ben Barak, A. Khalil, I. Zaidman, M. Haimi, M. Weyl Ben Arush
*(Pediatric Oncology/Hematology Department, Meyer Children's Hospital, Rambam Medical Center, Haifa, Israel)*

*Background:* Metastatic disease (MD) is the main cause of death in children with sarcoma.
*Objectives:* To evaluate the influence of MD on survival among children wit bone (BS) and soft tissue sarcomas (STS).
*Patients and methods:* Medical charts of twenty-six pts were evaluated. There were 14 boys and 12 girls with median age of 13.5 years (range, 1–19 years). Fifteen pts were with BS (Ewing's sarcoma – 8 pts and osteogenic sarcoma – 7 pts) and 11 pts – various STS. Seven pts developed bone metastases (5 – isolated and 2 – combined with lungs) and 19 – metastases in other than bone location (lung – 15 pts isolated or in combination with other locations).
*Results:* With median follow-up of 33.6 months, 19 pts are DOD, 5 pts – NED, 2 pts – AWD. Median survival after diagnosis of MD was 13.6 months for BS pts and 27.0 months – for STS ($p = 0.049$). Median survival did not differ between group with bone MD (24.1 months) and group with metastases in other locations (17.3 months), $p = \text{NS}$.
*Conclusions:* 19% of sarcoma pts with metastases are currently NED. Survival depends on histological diagnosis but not on location of metastases in studied cohort of pts. More efficient methods of treatment of MD are required.

### P128

**Radiological pseudo-progression of primary pulmonary metastases in osteosarcoma: A case report**

J.C. Potratz[1], S. Bielack[1], T.M. Bernhardt[2], H. Bürger[3], G. Gosheger[4], M. Semik[5], H. Jürgens[1]
*([1]Departments of Pediatric Hematology and Oncology, [2]Clinical Radiology, [3]Pathology, [4]Orthopaedics, [5]Thoracic and Cardiovascular Surgery, University of Münster, Münster, Germany)*

Metastatic progression during chemotherapy is generally interpreted as chemotherapy failure associated with a dismal outlook. We report a 15-year-old girl with conventional osteosarcoma of the left distal femur. Initial chest CT scans showed two lesions in the right lung suspicious of primary pulmonary metastases. The patient received induction chemotherapy with doxorubicin, high-dose methotrexate, cisplatin and ifosfamide. Chest CT scans 9 weeks after the initial CT revealed a marked increase of bipulmonal sclerotic lesions in size and number, which had to be interpreted as progressive disease radiologically.

Surgery of the primary tumor was performed as scheduled in week 10. Histologic analyses revealed a good response (<10% viable tumor). Following week 13, staged bilateral thoracotomies were performed. A total of 20 histologically confirmed metastases were removed from both lungs, all void of viable tumor cells. Thus, a complete surgical remission was achieved, which is still maintained after 14 months of follow-up.

This case demonstrates that radiological "pseudo"-progression of sclerotic pulmonary osteosarcoma metastases during early chemotherapy need not necessarily indicate true disease progression, but might sometimes be explained by reactive sclerosis of pre-existing metastases not evident on initial imaging studies. This must be kept in mind when deciding about further treatment.

### P129

**Autologous peripheral blood stem cell transplant in paediatric patients with poor prognosis solid tumors solid tumors in a developing country: A pilot study**

S. Poyiadjis, L. Wainwright, G. Naidu, D. Mackinnon
*(Paediatric Haematology-Oncology Unit, Chris Hani-Baragwanath Hospital, University of the Witwatersrand, Soweto, Johannesburg, South Africa)*

*Introduction:* Cyropreserved autologous Peripheral Blood Stem Cells (PBSC) have been used by our unit for transplant in children with poor prognosis solid tumours since 1999.
*Method:* Twelve patients underwent PBSC harvest with a view to be transplanted in combination with high dose chemotherapy. Seven patients never progressed to transplantation due to failure of the initial "window" treatment.

Five patients had their PBSC transplant: four patients with Rhabdomyosarcoma (RMS) and one with recurrent Wilms tumour. The protocols used were the ones recommended by the UKCCSG.
*Results:* One patient with RMS died six months after transplant from progression of the disease. Three patients with RMS and one with Wilms tumour are alive: 8, 23, 46, 52 months after completion of therapy.

The patient who died still had macroscopic disease when entering the Sequential High Dose Monotherapy programme, unlike the other four survivors.

The harvest of the PBSC, the CD34 yield and engraftment were all satisfactory.
*Discussion:* Paediatric poor prognosis advanced solid tumours in developing countries can be successfully treated with aggressive therapy in combination with autologous PBSC transplant. Macroscopic disease at the time of transplantation is a poor factor.

### P130

**Rotationplasty: Is there still a role?**

A. Puri, C. Anchan, M.G. Agarwal, Y. Panchawagh
*(Department of Orthopaedic Oncology, Tata Memorial*

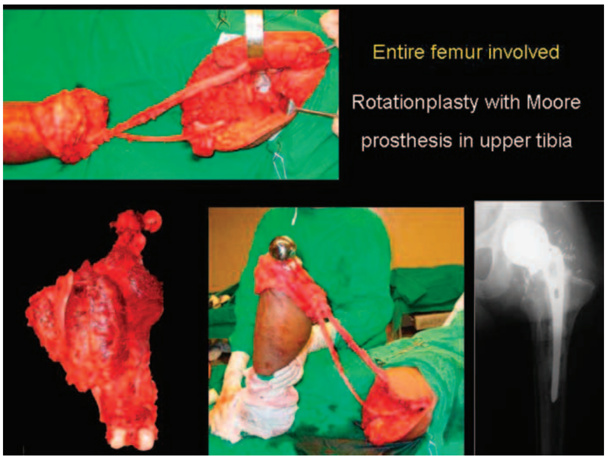

*Hospital, Mumbai, India)*

Due to the incidence of large tumors at index presentation precluding conventional limb salvage and the high cost of expandable prosthesis for limb salvage in the paediatric population, rotationplasty continues to be an often used procedure in the developing world.

35 patients underwent rotationplasty at our institute from January 2000 to September 2004. The age ranged from 6 to 25 years. Twenty nine patients had osteosarcoma, 5 had Ewing's sarcoma and 1 patient underwent rotationplasty for failed megaprosthesis. The involved bone included lower end femur in 28 cases, upper femur in 1 case, upper tibia in 4 cases and entire femur in 2 cases.

Two of these patients underwent an amputation following post operative vascular compromise. Other complications included vascular congestion in 2 cases which recovered after exploration, partially recovered nerve palsy in 1, wound infection in 2, local recurrence in 1 and non union requiring bone grafting in 1 case.

Functional evaluation was documented in 25 patients with a follow up ranging from 3 months to 56 months. Using the MSTS scoring system the score was 25 or greater in 22 of 25 patients.

In appropriately indicated cases rotationplasty is an option with excellent oncologic and functional results.

**P131**

**Renal sarcomas in the intergroup rhabdomyosarcoma group experience: 1972–1999**

B. Raney, J. Anderson, S. Qualman, M. Wharam, E. Wiener, W. Meyer
*(The Children's Oncology Group, Arcadia, CA, USA, Division of Pediatrics at the UT MD Anderson Cancer Center, Houston, TX, USA)*

*Purpose:* To ascertain clinical characteristics, treatment, and outcome of IRSG patients with renal sarcomas.
*Patients/methods:* The IRSG database includes newly diagnosed patients <21 years old with rhabdomyosarcoma [RMS] or undifferentiated sarcoma [UDS].
*Results:* Five of the 4514 IRSG patients [0.1%] were diagnosed with renal embryonal RMS [$N=4$] or UDS [$N=1$]. Patient ages ranged from 2 to 12 years; four were female. Tumor diameters ranged from 8 to 15 centimeters. One patient underwent complete removal of localized tumor [Group I]; 1 patient had localized gross residual tumor [Group III], and 3 had distant metastases in lungs and bone [Group IV] at diagnosis. All patients received vincristine, dactinomycin, and cyclophosphamide; 2 also received topotecan. The 4 patients with residual disease received radiotherapy. All 5 patients achieved a complete response, but the 3 with Group IV disease recurred in lung [$N=2$] or brain [$N=1$] and died within 13 months. The Group III patient died of *Aspergillus* pneumonia. Only the Group I patient survives, continuously disease-free, at 17+ years.
*Conclusions:* Renal sarcomas enrolled on IRSG protocols were large and often presented with advanced disease. Removing all gross tumor at diagnosis may be the best approach to curing these patients.

**P132**

**Outcome of residual structures present at the end of therapy in parameningeal rhabdomyosarcoma**

J. Robertson, C. Int-Veen, T. Dantonello, I.B. Brecht, S. Kirsch, E. Koscielniak, J. Treuner
*(CWS-Study Group, Olgahospital, Stuttgart, Germany)*

*Introduction:* Parameningeal rhabdomyosarcomas are difficult to treat surgically due to the proximity of vital structures. Following radio- and chemotherapy, residual structures often persist at the end of treatment, making it difficult to rule out the presence of small residual tumors.
*Patients:* 141 patients registered with the German Soft Tissue Trials (CWS) between 1991 and 1996 who had a pm-RMS were evaluated. Inclusion criteria were: Intention to treat, alveolar or embryonal histology, treatment in Germany, diagnosis by December 31, 2001, under 22 years of age at the time of diagnosis and Stage II or III.
*Results:* 35 (25%) patients had an unclear residual structure at the end of treatment. 37 (26%) had a residual tumor, 32 (23%) no residual structure and in 24 (17%) patients, the residual structure was described as inflammation or scar tissue. There was no information on 12 patients, and one patient was lost. 7 (20%) patients with an unclear residual structure had a relapse. 5 (14%) of the patients with a residual tumor relapsed. Of the patients without a residual structure, 7 (22%) relapsed. 13 (54%) patients with a residual structure described as inflammation or scar tissue had a relapse.
*Discussion:* Unclear residual structures do not have a significantly higher risk of relapse compared to no residual structure, residual tumors have similar rates of relapse if full remission has been achieved, and residual structures described as inflammation or scar tissue have an unusually high risk of relapse.

**P134**

**Adamantinoma: A new histological variant**

N. Little, B. Rogers, J. Pringle, S.R. Cannon
*(Royal National Orthopaedic Hospital, UK)*

Adamantinoma is a rare low-grade malignant epithelial bone tumour constituting up to 0.5% of all malignant bone tumours. An expansile, osteolytic mid-diaphyseal tibial lesion was found in a 12 year-old girl. An initial histological diagnosis of basaloid-type adamantinoma was made. Following excision, further histology demonstrated basaloid cells and acellular matrix focally surrounded by osteoclast giant cells with calcium deposits, features consistent with pilomatrixoma. Several histological variants of adamantinoma have been documented; this case details a previously unreported histological adamantinoma variant – pilomatrixoma-adamantinoma.

**P135**

**Rb-Loss: A genetic event in high grade chondrosarcoma with low relevance to clinical practice**

M. Röpke, B. Meyer, R. Schneider-Stock

*(Department of Orthopedic Surgery and Department of Pathology, Otto-von-Guericke-Universtiät Magdeburg, Germany)*

Loss of function of the human retinoblastoma gene (Rb) is a frequent genetic abnormality in human malignancies and causes a disturbance of cell cycle and loss of normal proliferation and differentiation. We studied loss of heterozygosity (LOH) of the Rb gene in 31 formalin-fixed, paraffin-embedded cartilaginous tumors using polymerase chain reaction. Both components of DD-chondrosarcoma, the low grade component and the anaplastic component, were separated by a microdissection approach. The genetic data were correlated with the expression of the Rb protein examined by Rb-immunohistochemistry. We found Rb-LOH in one chondrosarcoma grade 3, and in the anaplastic component in 7 of 8 cases of DD-chondrosarcoma (89% of all high grade chondrosarcomas). All benign cartilaginous tumors (four enchondromas and two chondroblastomas), low grade chondrosarcomas and low grade tumor components of DD-chondrosarcomas were negative regarding Rb-LOH. We concluded that Rb-LOH predominantly occurs in highly malignant chondrosarcoma. However, it is not a marker for identifying low grade tumors with a tendency towards progression or local recurrence. This was confirmed by the evaluation of the complete clinical histories and long-term follow-up data which was available in 13 cases. Radical or strictly wide surgery will still act as the guarantor for the successful treatment of chondrosarcoma.

### P136

**Plakoglobin expression depends on DNA methylation and acetylation in alveolar rhabdomyosarcoma**

T. Gastaldi[1], P. Bonvini[1], F, Sartori[1], A. Marrone[2], A. Iolascon[2], A. Rosolen[1]
*([1]Clinica di Onceomatologia Pediatrica, Azienda Ospedaliera-Università di Padova, Padova, Italy, [2]CEINGE, Università di Napoli, Napoli, Italy)*

Plakoglobin and β-catenin are pivotal components of cell-cell adherent junctions. β-catenin over-expression induces cell proliferation and tumor formation whereas high levels of plakoglobin suppress cell proliferation and tumorigenicity.

β-catenin and plakoglobin expression was studied in 5 alveolar (ARMS) and 4 embryonal (ERMS) rhabdomyosarcoma (RMS) cell lines and 11 RMS biopsies by Western-blot. β-catenin was homogeneously expressed in all samples, whereas plakoglobin was absent or barely detectable in ARMS. Semi-quantitative RT-PCR analysis confirmed this expression pattern. Cell fractionation analyses demonstrated that both catenins localized in the cytoplasm and in the nucleus.

Because aberrant methylation and/or acetylation of promoter regions may silence gene expression, the effects of the DNA demethylating agent 5-Aza-2′-deoxycytidine (5AzadC) and of the histone deacetylase inhibitor Trichostatin-A (TSA) were studied in RMS lines. We showed a partial dose-dependent restoration of plakoglobin expression in 5AzadC treated ARMS cells, whereas TSA-treatment caused a time-dependent increase in plakoglobin expression. β-catenin was not affected by either 5AzadC or TSA. We observed a synergistic increase of plakoglobin expression in ARMS cells treated with both agents.

The methylation status of promoter-associated CpG islands was also determined in ARMS and ERMS.

Our results may explain some of the biological and clinical differences between ARMS and ERMS and represent the basis for a wider functional characterization of catenins in RMS and other solid tumors of childhood.

### P137

**Optimization of a real-time RT-PCR assay for minimal residual disease studies in rhabdomyosarcoma**

F. Sartori, R. Alaggio, G. Albiero, L. Mussolin, A. Rosolen
*(Clinica di Oncoematologia Pediatrica, Azienda Ospedaliera-Università di Padova, Padova, Italy)*

Real time quantitative reverse transcription polymerase chain reaction (RQ-PCR) is a powerful tool for measurements of gene expression for diagnostic and prognostic purposes in several malignancies. To this aim the establishment of uniform methods for normalization of RNA input is relevant.

In this study we searched for the most useful endogenous control genes for normalization of the measurements, based on their lowest variability among bone marrow (BM) aspirates from different RMS patients.

We analyzed the RNA expression of 11 potential endogenous control genes. The sensitivity of the method was assessed by limiting dilutions of alveolar RMS tumor cells RH30 in hematopoietic cells. Subsequently, we studied the expression of the RMS markers MyoD1 and Myogenin in 10 non-RMS cell lines and 5 BM aspirates from non-RMS patients in order to define their specificity.

The GUS and HPRT genes exhibited the lowest variability among the BM specimens. MyoD1 and myogenin sensitivity ranged from 10–5 to 10–6 and they were never detected in specimens other than RMS.

Preliminary results from an ongoing study of 25 RMS patients confirmed the potentials and advantages of the RQ-PCR assay compared to standard RT-PCR.

QR-PCR appear a favorable option for the analysis of specific transcripts for the study of minimal residual disease in children with RMS.

### P138

**Comparative analysis of molecular and histological features in Ewing family tumors of childhood**

R. Alaggio, F. Sartori, S. Bitetti, V. Ninfo, G. Bisogno, A. Rosolen
*(Istituto di Anatomia Patologica e Clinica di Oncoematologia Pediatrica, Azienda Ospedaliera-Università di Padova, Padova, Italy)*

The Ewing family of tumors (EFT) harbors specific EWS/ETS gene rearrangements.

We investigated the morphologic and immunohistochemical features of 47 children with initial diagnosis of EFT and determined possible correlations between histology and molecular characteristics.

Chimeric transcripts, growth pattern (nested/solid), stroma (absent/abundant fibrosis) and cytology (small or epithelioid cells) were evaluated.

43 cases were EWS/ETS positive: They included 2 cases of ectomesenchymoma previously diagnosed as rhabdomyosarcomas. Other 4 cases were negative for any transcript: 2 were considered PNETs, 1 synovial sarcoma and 1 undifferentiated sarcoma.

Of the 41 Ewing/PNETs EWS-FLI1 fusion type-1 transcript was detected in 21 cases, type-2 in 11, other EWS-FLI1 variants in 6 and EWS-ERG in 3. Solid growth pattern (31/41 cases), and absent stroma (24/41 cases) were not associated with specific transcripts. Epithelioid cells and atypical PNET features were more frequent in tumors carrying transcripts other than type-1/2 (prevalence of 80 and 75%). These characteristics were associated with weak CD99 positivity (60%). No differences were detected in neural marker expression.

Morphology alone allowed a correct diagnosis in 95% of cases. Detection of specific transcripts appeared critical in the differential diagnosis. Uncommon variant chimeric transcripts were associated more frequently with "atypical PNET" features and weak CD99 expression.

### P139

**Gene expression profilino identifies potential relevant genes in alveolar rhabdomyosarcoma pathogenesis and discriminates PAX3-FKHR positive and negative tumors**

C. De Pittà[1], L. Tombolan[1], G. Albiero[1], F. Sartori[2], C. Romualdi[1], M. Carli[2], G. Lanfranchi[1], A. Rosolen[1]
*([1]CRIBI Biotechnology Centre, University of Padova, Padova, Italy, [2]Clinica di Oncoematologia Pediatrica, Azienda Ospedaliera-Università di Padova, Padova, Italy)*

Some morphological and biochemical characteristics are common to alveolar (ARMS) and embryonal (ERMS) rhabdomyosarcoma (RMS) and suggest that RMS arises from a disturbance in the normal skeletal muscle development.

To study the pathogenesis of ARMS we used the expression profiling technology with cDNA microarrays on 5 PAX3-FKHR positive and 5 negative ARMS.

Gene expression profiles allowed to successfully distinguish PAX3-FKHR positive and negative tumors and to discuss the role of some genes that might be biologically relevant for the two ARMS subtypes (RAC1, CFL1, CCND1). Interestingly, the majority of ARMS examined (9/10) could be correctly classified in two clinical groups based on only 11 discriminating genes. We confirmed the predictive value of this small set of genes analyzing blindly 4 additional ARMS biopsies. All 4 patients were successfully classified, with 100% accuracy.

Transcription profiles of tumor samples and normal fetal skeletal muscle were also compared. 171 differentially expressed genes were found in all of the 10 ARMS. Of them, 53 (31%) were over-expressed and 118 (69%) under-expressed. The functional analysis of altered genes led to the identification of a group of transcripts (BCOR, DDX5, LGALS1, BIN1) that may be relevant in the tumorigenic processes leading a normal muscle cell to become a malignant rhabdomyoblast.

### P140

**Response of progressive fibromatosis to therapy with liposomal doxorubicin**

J. Rossler[1], G. Wehl[1], J.E. Otten[2], N. Boehm[3], M. Uhl[4], U. Kontny[1], C.M. Niemeyer[1]
*([1]Division of Pediatric Hematology and Oncology, [2]Department of Pediatrics, [3]Department of Oral and Maxillofacial Surgery, [4]Insitute of Pathology, [5]Section of Pediatric Radiology, [6]Department of Radiology, University of Freiburg, Germany)*

*Introduction:* Patients with progressive, unresectable fibromatosis may suffer from high morbidity. Various chemotherapeutic regimens have been tried in these patients with limited success. Here, we report on the successful use of pegylated liposomal doxorubicin in the treatment of 4 patients with unresectable fibromatosis in unfavorable localizations.
*Case reports:* Three children and one adult with progressive fibromatosis were treated with three-weekly cycles of chemotherapy with liposomal doxorubicin (dose range 20 mg/m$^2$ to 50 mg/m$^2$ per day every 21 days). Tumors were located at the nasal cavity, fossa infratemporalis, oral cavity, abdomen and fossa supraclavicularis, and were surgically unresectable. Three of the four patients had been heavily pretreated with various chemotherapeutic agents. Objective tumor response was monitored by magnetic resonance imaging and possible cardiotoxicity by echocardiography at regular intervals. A tumor response was obtained in all four patients. All patients showed normal cardiac function after completion of chemotherapy as evaluated by left ventricular shortening fraction. Following chemotherapy cycles severe neutropenia was not observed.
*Conclusions:* Pegylated liposomal doxorubicin is a therapeutic option in patients with progressive unresectable fibromatosis in unfavorable localizations.

### P141

**"Other" localisations of rhabdomyosarcoma in children in the PPGGL materials**

A. Rybczyńska, B. Kazanowska, A. Reich, A. Balcerska, W. Balwierz, J. Bodalski A. Chybicka, A. Dłużniewska, E. Adamkiewicz-Drożyńska, K. Kątski, J. Kijowski, J. Kowalczyk, A. Kurylak, M. Krawczuk–Rybak, E. Solarz, K. Stefańska, D. Stencel, B. Szewczyk, J. Wachowiak, M. Wieczorek, W. Woźniak, M. Wysocki
*(Paediatric Haematology and Oncology Departments: Poznań, Białystok, Bydgoszcz, Chorzów, Gdańsk, Kraków, Lublin, Łódz, Warszawa, Wroclaw, Poland)*

The aim of the research was an analysis of RMS occurring in children in so called "other" localisation. The object of the analysis was children aged one month to seventeen years and six months (median age 11 years 5 months). The most common localisation were: chest wall ($n=11$, 22.5%), pelvis ($n=10$, 20.4%), abdominal cavity ($n=8$, 16.3%), vertebral canal ($n=4$, 8.2%), retroperitoneal space ($n=3$, 6.12%), mediastinum ($n=2$, 4.1%). The most common clinical stages were: III ($n=22$, 44.9%), IV($n=17$, 34.7%). The size of the tumor at the most of the children was bigger than 5 cm. In TNM, T2b changes dominated. In 17 (34.7%) cases metastases were recognised at the moment of diagnosis – in nodes, bones, pleura, lungs and bone marrow. In 12 (24.5%)cases the tumor was recognised as RMS and the rest was diagnosed as non-RMS (PNET mainly). The first step of the treatment was chemotherapy. In 38 (77.6%) cases R2 resection was carried out and pT3b (=27, 55.1) stage was the most common. Remission was diagnosed in 71.4% of children, especially those who under went combined treatment. The treatment has been successfully completed in 38 (77.6%) cases, in 4 (8.2%) progression was diagnosed (local progression in 3 cases). 13 children (26.5%), died mainly because of progression ($n=6$) and return of the illness ($n=3$). Localisation: Abdominal cavity ($n=3$), chest wall ($n=3$), vertebral canal ($n=1$), pelvis ($n=1$). The most common were non-RMS tumors. Period of observation: 5 months to 6 years and 9 months.
*Conclusions:* 1. The patients with so called "other" localisation of RMS are a diver group. 2. Further detailed analysis is needed.

### P142

**Stratified single and double autologous bone marrow trasplantation for Ewing's sarcoma family of tumors**

A. Sawada, M. Inoue, M. Yasui, T. Hamazaki, T. Kishimoto, I. Saitoh, T. Nakano, M. Koyama, Y. Takeshita, A. Sakata, T. Okamura, N. Sakata, K. Yagi, K. Kawa

*(Pediatric Hematology/Oncology, Osaka Medical Center and and Research Institute for Maternal and Child Health)*

*Purpose:* We explored the efficacy and safety of single (for standard-risk) and double (for high-risk) autologous BMT for Ewing's sarcoma family of tumors (ESFT).
*Patient and methods:* Eight patients (3 localized, 4 metastatic and one relapsed tumors) were treated in our institute from 1993 to 1999. All but one were treated at first with 3–5 courses of window chemotherapy which consisted of cyclophosphamide, pirarubicin, cisplatin, and vincristine or etoposide. The patients with primary tumor of pelvis or bone were treated with 1st BMT, and then the primary sites were irradiated 30 Gy. In other patients, the primary tumors were resected and irradiated (if residual diseases were presented), and then treated with 1st BMT. The patients without favorable clinical setting (completely resected primary tumor and no distant metastasis) underwent 2nd BMT.
*Results:* Among 8 patients, five (two localized, two metastatic and one relapsed diseases) are alive well for 5–12 years, and three (one localized and two metastatic diseases) died.
*Discussion:* Chemo-resistance is the only adverse prognostic factor, and we otherwise observed no late relapse. Thus, autologous BMT appear to be feasible and effective for ESFT. The latter patients may need other novel therapeutic modality in combination with high-dose chemotherapy.

### P143

**Long term survival (more than 10 years) of patients with osteosarcoma and multiple synchronous metastases**

T.O. Schneider[1], S. Bielack[2], B. Bode-Lesniewska[3], B. Kempf-Bielack[2], A.R. von Hochstetter[4], H.P. Honegger[5], J. Streuli[1], G.U. Exner[1]
*([1]Orthopädische Universitätsklinik Balgrist, Zürich, Switzerland, [2]Pädiatrische Hämatologie und Onkologie, Klinik und Poliklinik für Kinder- und Jugendmedizin, Universitätsklinikum Münster, Germany, [3]Institut für klinische Pathologie, Departement Pathologie, Universitätsspital Zürich, Switzerland, [4]Pathologieinstitut Enge, Zürich, Switzerland, [5]Institut für medizinische Onkologie und Hämatologie, Stadtspital Triemli, Zürich, Switzerland)*

*Rationale:* Few patients with osteosarcoma (OSA) and multiple synchronous metastases survive longer than 10 years after first diagnosis. We present data of long term survivors.
*Materials and methods:* 9 OSA patients with metastases at 3 sites (at least 2 histologically proven) ore more were identified among COSS registry surviving more than 10 years. Median age was 20.2 (range 8–45) years. Primary tumor sites were the femur (7) and pelvis (2). All patients had synchroneous pulmonary metastases, 4 patients had additional skeletal metastases (rib, vertebra, femur, pelvis). All patients had intensive chemotherapy according to COSS protocols, surgery when required and repeat lung metastasectomies
*Results:* Two patients were lost to f/u after 10 years without disease manifestation. Complete remission was achieved in four patients with an average of 16.2 years without relapse. One patient with multiple synchronous lung metastases is alive with disease at 15.6 years. One patient with multiple synchronous pulmonary metastases and multiple skeletal metastases lives at 10.1 years with stable disease, but free of symptoms. One patient died 14.4 years after diagnosis from lung disease.
*Conclusion:* The data presented point to the fact that some patients with extensive disease may survive for long time if they undergo adequate treatment. Initially, treatment in most of these patients was considered only palliative.

### P144

**Radiotherapy in Ewing tumors of the vertebrae: Treatment results and local relapse analysis of the CESS 81/86 and EICESS 92 trials**

A. Schuck[1], S. Ahrens[2], I. von Schorlemer[1], M. Kuhlen[2], M. Paulussen[2], A. Hunold[2], G. Gosheger[3], W. Winkelmann[3], J. Dunst[4], N. Willich[1], H. Jürgens[2]
*([1]University Hospital of Muenster, Germany, Departments of Radiotherapy, [2]Pediatric Oncology and Hematology, [3]Orthopedics, Muenster, Germany, [4]University Hospital of Halle, Department of Radiotherapy, Halle, Germany)*

*Purpose:* Treatment results in patients with Ewing tumors of the vertebrae enrolled in the CESS 81, 86, and EICESS 92 trials were analyzed with special emphasis on radiation-associated factors.
*Methods:* A retrospective analysis was performed on 116 patients with primary tumors of the cervical, thoracic, or lumbar vertebrae treated between 1981 and 1999. Furthermore, a relapse analysis was done on those patients who underwent radiotherapy and subsequently had a local recurrence.
*Results:* 64.6% of the patients received definitive radiotherapy, 27.5% of patients had surgery and radiotherapy. Only 4 patients underwent definitive surgery. 27 patients presented with metastases at diagnosis. 22.4% of the total group developed local relapses. Among the subgroup with definitive radiotherapy local recurrence was seen in 17 of 75 patients (22.6%). Event-free survival and survival at 5 years were 47% and 58%. Of 14 evaluable local relapses following radiotherapy, 13 were in-field. No correlation between radiation dose and local control could be found.
*Discussion:* Local control is poor in vertebral Ewing tumors. The results following definitive radiotherapy in vertebral tumors are comparable to those of other tumor sites when definitive radiotherapy is given. Nearly all local relapses following radiotherapy are in-field.

### P145

**Radiotherapy in adult type soft tissue sarcoma (sts): Results of the CWS trials**

A. Schuck[1], C. Int-Veen[2], S. Kirsch[2], T. Dantonello[2], I. Brecht[2], B. Schmidt[3], E. Koscielniak[2], J. Treuner[2]
*([1]Radiotherapy Department, University Hospital Muenster, Germany, [2]Department of Pediatric Oncology/Hematology, Olgahospital Stuttgart, Germany, [3]Radiotherapy Department, Katharinenhospital, Stuttgart, Germany)*

*Purpose:* The results of patients with adult type sts registered in the CWS trials are analyzed.
*Methods:* All patients with "adult type" sts registered between 6/1981 and 12/2002 in the CWS trials were included in the analysis. Treatment consisted of local therapy and in some cases chemotherapy. The median follow up of surviving patients was 51 months.
*Results:* There were 274 patients with localized disease. In IRS group I, 2 of 14 patients (14%) with radiotherapy relapsed locally and 8 of 50 patients (16%) without. In IRS group II, 8 of 33 patients (24%) with radiotherapy relapsed locally and 8 of 18 patients (44%) without (difference not significant). In IRS group III, 51 patients received second surgery. 7 of the 25 patients (28%) with additional radiotherapy and 1 of 16 patients without additional radiotherapy (6%) relapsed locally. Patients with RT-doses >45 Gy experienced less local failures.
*Discussion:* Radiotherapy is essential in adult type STS IRS group II and IRS group III without oncological radical secondary surgery. It is still open questions whether patients with primary or secondary resection with free margins profit from additional RT.

### P146

**Complete recovery from severe methotrexate intoxication after high-dose methotrexate therapy for sarcoma by early use of carboxypeptidase G2**

S. Schwartz[1], L. Fischer[1], A. Korfel[1], K. Jahnke[1], T.R. Auton[2], E. Thiel[1]
*([1]Medizinische Klinik III, Charité – Campus Benjamin Franklin, Berlin; Germany, [2]Protherics PLC, Runcorn, Cheshire, UK)*

*Purpose:* Accumulation of methotrexate (MTX) after high-dose MTX (HD-MTX) therapy is a life-threatening complication. Carboxypeptidase G2 (CPG2) rapidly cleaves MTX into non-toxic metabolites. Few data exist on the efficacy of CPG2 in patients (pts) with excessive MTX accumulation.
*Patients/methods:* We have identified 4 sarcoma pts treated with CPG2 for very severe MTX-intoxication in a nationwide survey.
*Results:* Four pts (20–35 years; 2 osteosarcoma, 2 chondrosarcoma) with renal failure (serum creatinine: 308–362 μmol/l) and serum MTX levels of 567, 304, 158 and 292 μmol/l were registered 19, 40, 48 and 48 hours after start of HD-MTX (5464–12 005 mg/m$^2$, 4 hour infusion), respectively. Risk factors were overweight ($n=2$), suboptimal hydration ($n=2$), low urine pH ($n=2$), preexisting renal insufficiency ($n=2$), relevant comedication ($n=2$) and urine flow obstruction ($n=1$). CPG2 (15–35 units/kg/body weight) was given as a single intravenous injection 28–61 hours after start of HD-MTX and tolerated in all pts without side effects. Serum creatinine levels returned to normal in all pts by days 23–43 after CPG2 treatment.
*Discussion:* CPG2 is effective and well tolerated in patients with excessive MTX-intoxication and facilitates complete recovery from renal failure. Future studies might evaluate the potential role of CPG2 as a standard rescue agent in high-risk pts.

### P147

**Radiotherapy in axial osteosarcomas**

R. Schwarz[1], M. Kevric[2], J.-N.Machatschek[2], S. Flege[2], S. Bielack[2]
*([1]Department of Radiation Oncology, University-Hospital Hamburg-Eppendorf, Hamburg, Germany, [2]COSS, Department of Pediatric Hematology and Oncology, University Children's Hospital Muenster, Germany)*

*Purpose:* Analysis of the Cooperative Osteosarcoma Study Group (COSS) experience with radiotherapy of axial osteosarcoma and review of the literature.
*Patients/methods:* 45 patients with biopsy-proven high-grade axial osteosarcomas of different sites (pelvis 32, cranial 9, others 4)and three with metastases in axial sites who had received radiotherapy at some time during their treatment course were identified from the COSS database. Median age was 21 (5–63) years. All patients received chemotherapy in accordance to COSS protocols. Radiotherapy was decided individually. Indications were unresectable primary tumor (24), preoperative (4), postoperative treatment (4), local recurrence (10), and metastases (6). Different modalities were used: photontherapy (31), photons plus high-dose samarium-153-EDTMP (9), and others (8). Median dose in curative treatment was 60 Gy (30–75), in palliative treatment 50 Gy (30–60).
*Results/discussion:* Median follow-up was 1.7 (0.1–9) years. 27 patients died. 16 of 29 patients with curative treatment and 5/19 patients with palliative treatment were alive. In 16 patients tumors were locally controlled or showed no further local progression. Prognosis of axial osteosarcoma remains poor in spite of multimodality treatment. Surgery remains the standard for local therapy. High-dose radiotherapy in addition to chemotherapy and surgery may contribute to treatment in selected patients.

### P148

**Clinical results in the treatment of primary and locally recurrent retroperitoneal soft-tissue sarcomas**

M.H.M. Schwarzbach[1], S. Cardona[2], U. Hinz[3], F. Willeke[1], G. Mechtersheimer[4], C. Herfarth[1], M.W. Buechler[1], T. Lehnert[2]
*([1]Department of Surgery, [2]Division of Surgical Oncology, [3]Unit for Documentation and Statistics, [4]Institute of Pathology [5]Department of Surgery, Mannheim University Clinic, University of Heidelberg, Heidelberg, Germany)*

*Purpose:* The impact of local control on prognosis in primary (PT) and locally recurrent (LR) retroperitoneal soft-tissue sarcoma.
*Patients and methods:* 110 adult patients underwent surgery (1988–2003). Results were analyzed for prospectively gathered clinico-pathological data. Kaplan-Meier estimations and Cox regression analyses were performed.
*Results:* Resectability rate was 90%, being comparable for PT ($n=71$) and LR ($n=39$). Morbidity, mortality, blood loss, and operation time were comparable for PT and LR. Follow-up was 89 months. Local 2-, 3- and 5-year control rates after complete resection of PT were 66%, 66%, and 59% compared to 23%, 19%, and 9% for LR ($P<0.001$). The mean number of operations differed: PT (1.4) versus LR (2.4) ($P=0.0047$). The 5-year survival after complete resection were 51% for PT and 43% for LR ($P=0.39$). The 5-year survival were 65%, 29%, and 0% for complete/incomplete resection and exploration ($P<0.001$). High-grade and blood loss show a poorer prognosis.
*Discussion:* Comparable resectability, morbidity, mortality, blood loss, operation time, and hospitalization were observed for surgery of PT and LR retroperitoneal sarcomas. Repetitive surgery leads to survival rates after resection of LR as observed in PT. Survival improves with radicality of local resection.

### P149

**Analysis peri-operative factors in sarcoma surgery**

M.N. Wente[1], M.H.M. Schwarzbach[1], U. Hinz[1], C. Leowardi[1], G. Mechtersheimer[3], J. Debus[4], G. Egerer[5], H. Friess[1], M.W. Büchler[1]
*([1]Department of Surgery, [2]Unit for Documentation and Statistics, [3]Institute of Pathology, [4]Department of Radiology, [5]Department of Oncology, University of Heidelberg, Heidelberg, Germany)*

*Purpose:* Few data are available about STS patient population and peri-operative parameters of a specialized surgical unit.
*Patients and methods:* Prospectively data of surgically treated STS patients from 10/2001 to 10/2004.
*Results:* 159 patients underwent 179 operations (median age: 60.2 years). Visceral STS (VIS) (35.7%), retroperitoneal STS (RET) (31.3%), and extremity STS (EXT) (27.3%) dominated. GIST were seen in 65.7% of VIS, liposarcoma in 47.6% of the RET, and either malignant fibrous histiocytoma (30%) or liposarcoma (23.3%) of the EXT. Recurrence was treated in about half of the RET and one third of EXT. VIS were mainly primary disease. Operation time was 240 min, 210 min, 120 min for RET, VIS, and EXT. Blood loss was 500 ml, 210 and 50 for RET, VIS and EXT operations. Morbidity was 26.6% for VIS, 29.1% for RET and 34.7% for EXT. Lethality was 8.9%, 2.0, and 1.6 for RET, EXT and VIS operations. Recurrence was resected with acceptable peri-operative parameters and morbidity.
*Conclusion:* STS surgery has to be differentiated for VIS, RET, and EXT tumors. Recurrence is often treated in RET and EXT, while primary tumors predominate as in VIS. Tumor type and peri-operative factors vary with location. Monocentrical data are useful for the peri-operative management.

**P150**

**High-dose ifosfamide in relapsed or progressive Ewing's sarcoma**

B.M. Seddon, A.M. McTiernan, M.P. Michelagnoli, S. Gabbie, S. Daw, J.S. Whelan
*(London Bone and Soft Tissue Sarcoma Service, UCL Hospitals, London, UK)*

*Purpose:* To determine the response rate and toxicity to high dose ifosfamide (HDI) in patients with relapsed or refractory Ewing's Tumours.
*Patients and methods:* Between 1993 and 2003, 28 patients (15 males) were treated with HDI 12–18 g/m$^2$ (median 15 g/m$^2$) as part of multi component treatment strategies. Eleven patients were treated following disease progression during first line treatment, and 17 for relapsed disease. All patients had previously received ifosfamide. Twenty-five patients proceeded to further treatment.
*Results:* A total of 57 cycles were given, median 2 (range, 1–4) per patient. The principle toxicity, evaluable in 26 patients, was haematological, with 20 patients (77%) requiring admission for febrile neutropenia. Platelet and blood transfusions were required in 27% and 62% of cycles respectively. Ifosfamide-induced encephalopathy was observed in 8 patients, severe in 2. Objective response rate, measurable in 21 patients, was 19%, with 2 complete responses and 2 partial responses. Fifteen patients (54%) were judged to derive clinical benefit, (objective response, stable disease, or reduction of non-measurable disease). Median overall survival from the start of HDI was 10 months (95% Confidence Interval, 7–13).
*Conclusions:* HDI has activity in patients with relapsed or refractory Ewing's tumours who have been pre-treated with standard dose ifosfamide.

**P151**

**Imatinib is not an alternative treatment for uterine leiomyosarcomas**

C. Serrano[1], C. Mackintosh[1], D. Herrero[1], A.S. Martins[1], T. Hernández[2], J. Pérez-Fontán[2], A. Orfao[3], E. Serrano[4], A. Bullón[4], M. Abad[4], E. de Alava[1]
*([1]Laboratory of Molecular Pathology, [2]Tumor Bank, [3]Flow Cytometry Lab, Centro de Investigación del Cáncer, Universidad de Salamanca, Salamanca, Spain, [4]Department of Pathology, Hospital Clínico Universitario de Salamanca, Salamanca, Spain)*

*Background:* Several studies reporting that KIT is expressed in uterine leiomyosarcomas (UL), suggest a chance for a treatment with tyrosine-kinase inhibitors. Nevertheless, these drugs are effective only when KIT signalling pathway is constitutively active.
*Design:* Immunohistochemistry on a Tissue-microarray including 18 UL was performed with a KIT antibody used in GIST international clinical trials (Dako A4502). Western Blot (WB) with KIT and phospho 719Tyr-KIT, WB for antityrosine residues after immunoprecipitation with KIT, and flow cytometric studies were performed on 5 frozen tissue samples corresponding to 4 UL and 1 GIST. Exons 11 and 9 of c-kit and exon 12 of PDGFRA were sequenced in all cases.
*Results:* Only mast cells were immunoreactive in UL, whereas neoplastic cells lacked KIT immunoreactivity. Accordingly, flow cytometric studies showed that KIT immunoreactive cells in UL had features corresponding to mast cells. UL samples showed very faint KIT bands in WB, but lacked KIT activation, as assessed by WB for phospho 719Tyr-KIT, and WB for antityrosine residues after immunoprecipitation with KIT. No KIT or PDGFRA mutations were found.
*Conclusions:* Our results indicate that KIT is not involved in UL pathogenesis, and is not likely a target for anti-tyrosine-kinase drug therapy in UL.

**P152**

**Impact of whole-body MR and FDG-PET on staging and assessment of therapy response in patients with Sarcoma: Initial results of a prospective study**

C. Furth[1,2], B. Spors[2], J. Ruf[2], T. Voelker[1,2], D. Misch[1], B. Stöver[3], M. Gutberlet[2], R. Felix[2], G. Henze[1], H. Amthauer[2]
*([1]Department for Pediatric Oncology, [2]Clinic of Radiology, Nuclear Medicine and Radiation Oncology, [3]Clinic of Radiology, Department of Pediatric Radiology, Campus Virchow-Klinikum, Charité – Universitätsmedizin Berlin, Germany)*

*Purpose:* An adequate assessment of metastases is crucial for the initial staging and the consecutive therapeutic regimen but also for a reliable evaluation of response to therapy. We compared the value of whole body MR and FDG-PET for staging and therapy assessment.
*Patients/methods:* In this prospective pilotstudy we report on 5 pts (3f, 2 m; mean age: 10.7) with metastasized sarcoma who, apart from conventional staging by bone scan, x-ray and CT were also examined before and after chemotherapy both by FDG-PET and whole-body MR using STIR and T1-weighted (±contrast media) sequences. Histology and/or clinical/imaging follow-up served as a gold standard.
*Results/discussion:* Detection of nodal and visceral involvement correlated well with conventional imaging modalities ($n=4/5$ pts) with exception of mediastinal and intrapumonal metastases ($n=2$ pts). Bone and bone marrow lesions ($n=2/5$ pts) were detected with the highest accuracy using FDG-PET, followed by MRI and bone-scan. Therapy response was best predicted with PET. Both whole body examinations might therefore be a great asset to stage-adjusted therapy regimens, ultimatively also influencing patient outcome.

**P153**

**Osteosarcoma extrasceletale as second malignant disease after Wilm's tumor treatment case presentation**

J. Stefanowicz[1], D. Sierota[1], A. Balcerska[1], Z. Śledziński[2], K. Jaśkiewicz[3], E. Iżycka-Świeszewska[3], K. Połczyńska[1], S. Hać[2].
*([1]Department of Pediatrics, Hematology, Oncology & Endocrinology, Medical University of Gdańsk, Poland,*

[2]*Department of General Endocrine and Transplant Surgery, Medical University of Gdańsk, Poland,* [3]*Department of Pathology Medical University of Gdańsk, Poland)*

Secondary neoplasms are a rare but difficult problem in Wilms tumor treatment. The secondary neoplasm usually decrease dramatically the overall treatment outcome.

Authors presented a 23 years old male who underwent successful Wilms tumor treatment at age of 4 (III° grade, mix type). Patient underwent chemotherapy, left nephrectomy and radiotherapy. At age 13 this patient was operated on because of pancreatic head benign teratoma – local tumor excision was performed. At age 23 he suffered from pancreatic head osteosarcoma extra-sceletale. Patient underwent pancreatoduodenectomy. He died 6 months after the operation because of rapid local and distant osteosarcoma progress.

Patients who underwent extensive anti-cancer therapy are immuno-compromised and predisposed to second malignant disease development. In young patients radio-therapy may develop the malignant disease. Thus it seems reasonable to focus attention on early secondary neoplasm detection as well as in primary disease recurrence.

**P154**

**Sarcomas as second malignant neoplasms after solid tumours in children: One center experience**

J. Stefanowicz, K. Połczyńska, D. Sierota, B. Kaczorowska-Hać, E. Drożyńska, A. Balcerska
*(Department of Pediatrics, Hematology, Oncology and Endocrinology, Medical University of Gdańsk, Poland)*

The second neoplasm occurrence is serious complication of oncologic treatment reducing the probability of patient's survival. Risk of second and next malignancy in children cured of neoplastic disease predominates about 15–20 times risk of cancer appearance in general children population.

Among patients treated in our department between 1992 and 2004 second neoplasm was recognized in 13 cases. In 4 children there were sarcomas: Skeletal and extraskeletal osteosarcoma in 2 cases, chondrosarcoma in 1 case and undifferentiated sarcoma in 1 patient.

Wilms tumour was the previous neoplasm in 3 children and retinoblastoma was observed in 1 child. Second tumour occurred from 7 to 19 years after the first disease. Patients' age in time of diagnosis was 9–23 years. 3 children died between 6 months and 2 years from diagnosis. 1 male patient is alive, cured of undifferentiated sarcoma post retinoblastoma.

There was evident relationship between radiotherapy and second neoplasm in 3 patients. It is difficult to univocally define factors predisposing to second malignant tumours. The role of genetic agents is considered however chemotherapy and radiotherapy are thought to be the main causes of second malignancies.

**P155**

**Neurofibromatosis type 1 as a risk factor of malignancies in childhood. Prophylactic procedures in patients from one institution in Poland**

D. Sierota, J. Stefanowicz, K. Połczyńska, J. Wierzba, E. Bień, T. Stachowicz-Stencel, A. Szołkiewicz, E. Drożyńska, A. Balcerska
*(Department of Pediatrics, Hematology, Oncology & Endocrinology, University of Gdańsk, Poland)*

Neurofibromatosis type 1 (NF1) is quite frequent disorder of autosomal-dominant pattern.

The average incidence is about 1 per 3000 births. In patients with NF1 malignancies such as soft tissue sarcoma or leukemia are noted.

There are 61 children with NF1 suspicion observed in our department. Each child is carefully controlled in every 6 months as a outpatient. Pediatric, neurological and ophtalmological examination is performed during first visit and in case of any symptoms. Number of Lisch nodules, field of vision, visually evoked potential, audiogram and dermatological evaluation of skin abnormalities and also orthopedic examination are performed. In each case of asymptomatic NF1, MRI of brain and medulla is done in every 2 years.

Café-au-lait spots were observed in 61 children, freck-ling of armpits in 40, neurofibromas in 6, Lisch nodules in 1 patient. Secondary symptoms and complications such as mental retardation (5) and epilepsies (3 cases), benign or malignant CNS tumours (8), scoliosis (32) were noted. In 4 patients malignant neoplasms occurred (6.5%): RMS – 2 cases, Triton tumour – 1 case, MPNST – 1 case. 1 child died of disease progression, 1 of treatment complications (sepsis). 2 children are alive.

In our opinion patients with NF1 need permanent and specialist medical care.

**P156**

**Inverse prognostic impact of syt-ssx fusion type in paediatric synovial sarcomas treated according to the German cooperative soft tissue sarcoma study therapy protocol**

S. Stegmaier, I. Leuschner, J. Treuner, E. Koscielniak
*(Olgahospital, Stuttgart, Germany)*

Synovial Sarcomas (SS) are genetically characterized by the chromosomal translocation t(X;18) generating syt-ssx1 or syt-ssx2 gene fusions in more than 95%. Several studies indicated with variable significance that tumours bearing the ssx1 fusion transcripts have a worse outcome than tumours with ssx2 fusion. To assess the prognostic value of syt-ssx fusion type in patients treated in a prospective study, we investigated a group of 46 paediatric (age: 1–25 years) SS patients with syt-ssx positive tumours. All patients were registered in the CWS (German Cooperative Soft Tissue Sarcoma Study) between 1996 and 2002. All patients were treated with adjuvant chemotherapy. Fusion type was determined by RT-nested-PCR on RNA isolated from fresh/frozen or paraffin embedded tumour tissue. Kaplan-Meier analysis indicates significantly better event-free survival for patients whose tumours contain syt-ssx1 ($n = 26$) compared to -ssx2 fusions ($n = 14$) for localized disease ($p = 0.03$ log-rank-test), being at odds with other studies. This is to our knowledge the only prospective treated group with SS, which was analysed for the prognostic relevance of the fusion type. All other published results included retrospectively analysed patients treated according to individual or institutional recommendations. This may be the reason for our contrary findings in comparison to other publications.

**P157**

**Osteosarcoma: Experience with cisplatin and doxorubi-cin in a pediatric institution**

P. Streitenberger, L. Morán, M. Garcia Lombardi, P. Pesce, G. Rey
*(Unidad de Oncología, Hospital de Niños Ricardo Gutierrez, Buenos Aires, Argentina)*

The use of preoperative chemotherapy has been shown to be of direct and significant benefit to the osteosarcoma patient. There is virtually an immediate palliation of symptoms, early treatment of the micrometastasis and local control of the disease. We present our experience and results with cisplatin and doxorubicin.
*Methods:* In the years 1997–2004 twenty six patients with Osteosarcoma were treated. All children were given preoperative chemotherapy one to three cycles of Cisplatin 100 mg/m$^2$/day Day 1 and Doxorubicin 25 mg/m$^2$/day Day 1–3, then radical resection of primary tumor and finally postoperative chemotherapy was given.
*Results:* There were 24 evaluables cases, 21 with localized osteosarcoma, 3 with pulmonary metastasis. The age of the patients ranged between 5 and 17 years, (mean 13 years), half of them were males. The tumor was located in distal femur (69.2%), proximal tibia (23.1%) and others (7.7%). The pathology was conventional type in 88.5%. Surgery limb-salvage was attemped in 13 patients and amputation in 11. The overall survival was 54% and the event free survival was 50%, with mediam follow-up of 30 months (5 to 83 months). The outcome of localized osteosarcoma was 61% of overall survival and 57% of event free survival. All patients with metastasis died.
*Conclusion:* Despite the low number of evaluable cases, the results shown us the efficacy of cisplatin and doxorubicin in the treatment of localized Osteosarcoma. Multicentric studies must be done in order to evaluate the outcome of metastasic patients.

### P158

**Dose- and time-dependent apoptosis of human sarcoma cells by ibandronate and clodronate in vitro**

U. Stumpf[1], S. Goettig[2], M. Schoenherr[1], C. Eberhardt[1], R. Henschler[2], A.A. Kurth[1]
*([1]Department of Orthopaedic Surgery Stiftung Friedrichsheim. University Hospital Frankfurt/Main, Germany, [2]Institut of Transfusion Medicine and Immune Hematology, German Red Cross Blood Center Johann Wolfgang Goethe University Frankfurt/Main, Germany)*

*Introduction:* Bisphosphonates are known to induce apoptosis in different cell types. We tested and compared the effects the bisphosphonates Clodronate (CL) and Ibandronate (IB) could have in a direct way on human sarcoma cells in vitro.
*Methods:* Human osteosarcoma cells (SAOS2 and 791TMHOS) and Ewings's sarcoma cells (RD-ES and TC71) were treated with CL (2 mM, 1 mM-10–7 M) and IB (10–3 M-10–7 M) for 24, 48 and 72 h. Cell apoptosis was evaluated by fluorescent flow cytometric analysis: quantification via FACS and PI staining.
*Results:* CL-treated osteosarcoma-cells show after 72 h incubation with 2 mM 12.6% and 10–3 M 8.7% apoptopic cells. Ewing‘s sarcoma cells were evaluated after 72 h: 2 mM 10–25% apoptosis.

10–3 M IB causes on osteosarcoma cells after 48 h <10% and 72 h 10–25% apoptotic cells, Ewing sarcoma cells incubated with IB showed at 10–3 M and 10–4 M 10–25% and of 10–5 M and 10–6 M <10% of apoptopic cells.
*Conclusions:* Clodronate and Ibandronate have time- and dose-related direct effects on primary bone tumor cells: apoptosis and inhibition of cell proliferation, with Ibandronate having larger effects. The use of Ibandronate may be indicated as an adjuvant to existing chemotherapeutic protocols.

### P159

**Relapse of osteosarcoma in children: Treatment, results and prognostic factors**

V. Svesko[1], Z. Bekic[1], Lj. Poleksic[1], B. Sbutega[2], S. Savic[2], R. Jakovic[3], M. Atanackovic[4]
*([1]Institute for Oncology and Radiology of Serbia, Belgrade; Serbia & Montenegro, [2]Institute for Orthopedic Surgery "Banjica", Belgrade; Serbia & Montenegro, [3]Institute for Pulmonary Disease, Belgrade, Serbia & Montenegro, [4]Institute for Pathology, Belgrade, Serbia & Montenegro)*

*Objective:* The aim of our study was to evaluate results of treatment and analysis of prognostic factors in patients with the relapse of osteosarcoma.
*Patients and methods:* From 1990–2000, we treated 22 children with osteosarcoma relapse, median age 16. All patients received aggressive multimodal therapy (chemotherapy/surgery) in previous treatment for non-metastatic osteosarcoma.

In 19 pts pulmonary metastases were detected (6 solitary), while 3 pts had local relapse of disease. Disease-free interval (DFI) was more than 1 year in 8 pts.

Surgery was performed in 16 pts (thoracotomy 13, amputation 3).

Chemotherapy regimens were administered in all patients: IFO-VP16 (10 pts); HDMth/IFO-VP16 (8 pts); HDMth/Carbo-VP16 (4 pts).
*Results:* During 10–128 months follow-up period (Me = 36 mts) disease-free survival rate was 34.71%.

The most significant prognostic factors, that influenced survival were: presence of a solitary metastases ($p = 0.0188$), local relapse of disease ($p = 0.026$) completeness of resection ($p = 0.039$), DFI longer than 1 year ($p = 0.034$), and the tumor necrosis over 90% in previous treatment ($p = 0.047$).

There were no significant differences in survival in relation to the type of chemotherapy regimen applied.
*Conclusion:* The use of aggressive multimodal therapy (surgery/chemotherapy) and evaluation of prognostic factors are necessary for successful treatment in patients with osteosarcoma relapse.

### P160

**Lung metastases in patients with osteosarcoma: Radiological examination and the reality**

A. Szafrański, M. Rychłowska-Pruszyńska, W. Woźniak
*(The Clinic of Oncological Surgery in Children and Adolescents, Institute of Mother and Child, Warsaw, Poland)*

In the Institute of Mother and the Child, 382 osteosarcoma patients aged between 3 and 26 years were treated between 1985 and 2003. 117 patients had lung metastases. Treatment started with chemotherapy. After obtaining the stabilization or the partial regress of changes, metastases were removed by surgery. Patients with lung metastases which were treated by comprehensive treatment during the last five years were analysed on-the-job.
*Conclusions:* (1) Profitable is the effacement of metastases in the period of the stabilization after use of chemotherapy. (2) Intervention by surgery ascertains on average about 10% more metastases than are visible under examination by CT scan. (3) Any doubts concerning unspecific changes in visible lungs under CT examination, should be interpreted as disadvantageous to the patient and be verified by histopathological examination. (4) A most exact method of removing of metastatic changes in the lungs seems to be classical thoracotomy. (5) Repeated (2–3 times) excision of metastases seems to be advisable.

The effacement of metastatic focuses makes the obtainment of the many years survival of patients possible. 28% patients obtained five year survivals.

**P161**

**The significance of biopsy in the conservative treatment of bone tumours**

M. Rychłowska Pruszyńska, A. Szafrański, W. Woźniak
*(The Clinic of Oncological Surgery in Children and Adolescents, Institute of Mother and Child, Warsaw, Poland)*

The authors demonstrate the significance of bioptic examination in the diagnosis and treatment of bone tumours. A well conducted biopsy not only aims to allow a diagnosis to be made, but also determines the possible pathways of operational treatment.

The material subject to analysis is 382 cases of patients with malignant bone tumours who were treated at the Institute of Mother and Child within the years 1985–2003. In all the cases, neoplastic material was taken from the patient and examined histopathologically. This analysis includes the surgical technique used, the location from which the material was taken, and the complications that occurred.

The analysis of the material shows that the biopsy should be conducted by the same surgeon responsible for the eventual surgical procedure, which confirms the opinions of other authors interested in surgical treatment of bone tumours.

When performed correctly, a biopsy enables for a saving procedure to be performed, and may limit the number of local recurrences and complications in the form of inflammation.

**P162**

**Rhabdomyosarcoma in children: 10 year experience from a single institution in a developing country**

T. Priya Kumari[1], Dr. Ratheesan[2], V.G. Chellam[3], B. Rajan[2], P. Kusumakumary[1]
*([1]Pediatric Oncology, [2]Radiation Oncology, [3]Pediatric Pathology, Regional Cancer Centre Trivandrum, India)*

*Aim:* Clinicopathological study and survival analysis of Rhabdomyosarcoma (RMS) in children (0–14 yrs).
*Methods:* Retrospective analysis – RMS children treated between January 1992 and December 2001. Grouping by IRS system and survival by KaplanMeier method.
*Results:* 80 children had RMS. Median age 5.5 years. Primary sites were head and neck in 42.5%, extremity in 21.3%, genitourinary in 13.75%. IRS grouping showed Group1 3.8%, Group2 16.5%, Group3 74.7% and Group4 5.1%. 61.25% had embryonal RMS. 63 children were evaluable for treatment outcome. Group1 received chemotherapy (VAC $\times$ 1 year). Gp2 received chemotherapy + local RT. Gp3 and 4 were given VACAdr $\pm$ cisplatin and etoposide + RT. 11 children developed progressive disease. 7 died, 2 had progressive disease and 5 had infections. Overall survival was 76% and disease free survival was 80% at 13 years. Survival by stage was statistically significant ($p$ value = 0.02 by log rank test) Gp1 (100%) Gp2 (78%), Gp3 (77% ) Gp4 (0%). Survival by histology showed better survival for embryonal RMS (85%) compared to alveolar RMS (69%) $p$ value (0.05).
*Conclusion:* In our centre majority of children with RMS presented with advanced disease. An overall survival of 76% at 13 years could be achieved at our centre despite the limited resources available in our developing country.

**P163**

**Therapy of aggressive fibromatosis is still an open question: A series of patients treated at a single institution**

Z. Tomasevic, D. Ristic
*(Institute for Oncology & Radiology of Serbia, Belgrade, Serbia)*

*Background:* In the absence of large, randomized trials, the precise role of systemic therapy in this rare disease is unclear.
*Methods:* We reported a series of patients with inoperable, deep extra-abdominal aggressive fibromatosis, treated with combination of chemo-hormonal therapy. Therapy consisted of six cycles standard CVP regimen, followed by tamoxifen, 20 mg daily.
*Results:* During 1995–2004, nine patients, concomitantly, without selection, were included in this investigation. Median age was 24, with predominantly male sex (6/9). Extremities, was the most frequent localization (5/9). Three patients had bulky disease (over 10 cm). Five patients underwent previous surgery, 3 wide excisions and 2 palliative interventions. Complete remission was observed in one patient, partial remission in four and stabilization of disease in four patients. In category of responders, median duration to onset of response was 10 months (range 4–14); median response duration was 32+ months.
*Conclusions:* Systemic treatment should be considered in patients with aggressive fibromatosis for whom local treatment approaches are primary not possible or have failed.

**P164**

**The different responses to IGF-I stimulation for myogenin induction and cell cycle progression between human alveolar and embryonal rhabdomyosarcoma cells in comparison with non-tumor myoblasts**

K. Tsuchiya, H. Hosoi, T. Sugimoto
*(Department of Pediatrics, Kyoto Prefectural University of Medicine, Kyoto, Japan)*

*Purpose:* Elucidation of specific abnormalities on the signaling pathway in rhabdomyosarcoma (RMS) for developing new therapeutic agent(s) in the future treatment.
*Materials:* Rh30 and KP-RMS-MS human alveolar rhabdomyosarcoma (ARMS) cell lines, and Rh30mTOR-rr cell line stably expressing rapamycin-resistant mTOR mutants (mTOR-rr; S2035I), and RD and KP-RMS-KH human embryonal RMS (ERMS) cell lines.
*Methods:* Western blotting analysis to see myogenin induction and the signaling downstream insulin-like growth factor receptor (IGF-IR). FACS analysis for the cell cycle progression. (Results) In the two ARMS cell lines, IGF-I response for myogenin induction were well preserved. Rapamycin inhibited myogenin induction, but not in Rh30mTOR-rr cells, suggesting that the signal goes mainly through IGF-IR/mTOR pathway. IGF-I, however, did not cause G1 arrest as observed in non-tumor myoblast C2C12 cells, bur rather stimulated the cell cycle progression. On the contrary, in the two ERMS cell lines, IGF-I did not show any influence upon myogenin induction, G1 arrest or cell cycle progression.
*Discussion:* These differences on responses to IGF-I among human ARMS, ERMS and non-tumor myoblast cells may give a clue to further understanding of their different oncogenesis and clinical behaviors, and to discovery of new possible therapeutic target(s).

**P165**

**Free vascularized bilateral fibula graft: Biological reconstruction following resection of malignant bone tumors of the lower-extremity**

P.-U. Tunn, K.T. Moesta

*(Clinic for Surgery and Surgical Oncology, Charité Campus Buch, Berlin, Germany)*

*Purpose:* We present the free vascularized bilateral fibula graft as a method for reconstruction of long bones after resection of malignant tumors.
*Patients and methods:* Between 11/2000 and 08/2003 five patients (2 female, 3 male; average age: 15.2 years) received a biological reconstruction following resection of a malignant bone tumor (Ewing sarcoma: $n = 4$, osteosarcoma: $n = 1$) using a free vascularized fibula graft and plate fixation. Tumors were located in the femur ($n = 3$) and tibia ($n = 2$). Defects of median 16.4 cm (11.5–23 cm) had to be bridged. All Patients had multimodal treatment according to the EURO-E.W.I.N.G or COSS-96 protocol respectively. The median observation period was 52 months.
*Results:* R0-resection was achieved in all cases, none of the patients had local recurrence. Radiographic signs of osseous remodelling could be detected after two months. Full weight bearing on the affected leg was permitted after 6–10 months. Complications occurred in 3 patients (haemorrhage: $n = 1$, pseudarthrosis: $n = 1$, fracture: $n = 1$). None of the complications led to failure of the reconstruction or to amputation. MSTS ranking was very good in 3 and good in 2 patients.
*Discussion:* Biological reconstruction of osseous defects is always desirable if possible. Good functional results can be obtained by using free vascularized bilateral fibula grafts in the lower extremity.

**P167**

**Ewing's sarcoma family of tumors and spinal cord compression: The Pinda protocols experience**

M. Villarroel[1], J. Quintana[1], J. Tordecilla[2], C. Salgado[3], M. Reyes[4], A. Becker[5], P. Zolezzi[6], Y. Rayo[7], L. Neira[8]
*([1]The Programa Infantil de Drogas Antineoplásicas (PINDA) Ministry of Health, Chile: Hospital Luis Calvo Mackenna, Santiago, [2]Hospital Roberto del Río, Santiago, [3]Hospital Exequiel González Cortés, Santiago, [4]Radiotherapy, Instituto Nacional del Cáncer, Santiago, [5]Hospital Sótero del Río, Santiago, [6]Hospital Clínico, Valdivia, [7]Hospital Van Buren, Valparaíso, [8]Hospital Gustavo Fricke, Viña del Mar)*

*Objectives:* Report the experience of 35 patients with spinal cord compression, among 161 patients treated for Ewing's sarcoma family of tumors according to 3 national protocols (PINDA 87, 92, 98).
*Material/methods:* Fourteen males, median age 9 years; 16 patients had vertebral, 19 paravertebral tumors. Primary sites: Cervical 6, thoracic 8, lumbar 13, sacral 8. Pain and neurologic deficit was present in all patients. Twenty-six patients had localized disease, 22 had tumors >8 cm, 15 were PNET. All patients received chemotherapy.
*Results:* Laminectomy in 26 patients, 22 with neurological recovery. Seven patients received radiotherapy only, 5 are disease-free. Twenty-three received RT median dose 45 Gy. Five patients didn't receive RT (2 complete resection, 2 pogressive tumors, 1 refused treatment), all relapsed.

Median follow-up 102 months; 5-year overall survival and EFS, for localized tumors: 51.6% and 66.1%, (13 disease-free); 25% and 30% for metastasic tumors. Relapses: local 7, distant 2, both 4. Five patients progressed, 1 refused treatment, 1 had a secondary malignancy.
*Conclusions:* Results are comparable to those of other patients wit EFT and were not affected by treatment era, primary site or tumor size. Early surgical debulking and RT as local treatment, minimize neurologic sequelae and improves EFS.

**P168**

**Multimodal therapy for children and adolescents with Ewing's sarcoma family of tumors. Preliminary results of Chilean National Protocol Pinda 98**

M. Villarroel[1], J. Tordecilla[2], C. Salgado[3], M. Reyes[4], A. Becker[5], P. Zolezzi[6], Y. Rayo[7], L. Neira[8], M. Arriagada[9]
*([1]The Programa Infantil de Drogas Antineoplásicas (PINDA) Ministry of Health, Chile: Hospital Luis Calvo Mackenna, Santiago, [2]Hospital Roberto del Río, Santiago, [3]Hospital Exequiel González Cortés, Santiago, [4]Radiotherapy, Instituto Nacional del Cáncer, Santiago, [5]Hospital Sótero del Río, Santiago, [6]Hospital Clínico, Valdivia, [7]Hospital Van Buren, Valparaíso, [8]Hospital Gustavo Fricke, Viña del Mar)*

*Objectives:* Evaluate a pilot trial, (high dose vincristine, doxorrubicin, cyclophosphamide, and ifosfamide, etoposide), in the treatment of Ewing's sarcoma and primitive neuroectodermal tumor of bone or soft tissue. Estimate 5-year overall survival and event-free survival, improve local control, identify prognostic factors and define toxicity.
*Methods:* Fifty-four eligible patients were enrolled April 1998–December 2003; 46 completed chemotherapy. Local control: Irradiation to the primary tumor 55.6 Gy, complete surgical excision, or both.
*Results:* Thirty-three male patients, median age 10.5 years, 38 had localized tumors, 23 had tumors >8 cm. Eight patients had soft tissue tumors, 21 PNET. Twenty-one of 32 patients with localized tumors, remain disease free, median 44 months. Relapse: Local in 5 cases, metastasic in 1 and both in 4. Nine patients died of tumor progression, 1 of a secondary malignancy. At 5 years, OS was 69.7% and EFS 55.7%. One of 14 patients with metastasic disease, remains disease free for 16 months.
*Conclusions:* There was an improval in survival for patients with localized tumors (PINDA 92 EFS 5-years 47%). Relevant prognostic factors: extremities tumors (EFS 5-years 88.9% vs. 38.7%), ES histology (71.5% vs. 37.3% for PNET) and response to induction chemotherapy (complete remission 5-year EFS 85.7% vs. 34.4%).

**P169**

**Value of FDG-PET imaging for primary staging of malignant pediatric sarcomas within a prospective multicenter study (PET 2003)**

T. Voelker[1,2], D. Misch[1,2], B. Stöver[3], R. Felix[2], H. Amthauer[2], G. Henze[1]
*([1]Department for Pediatric Oncology, [2]Clinic of Radiology, Nuclear Medicine and Radiation Oncology, [3]Clinic of Radiology, Department of Pediatric Radiology, Campus Virchow-Klinikum, Charité – Universitätsmedizin Berlin, Germany)*

*Purpose:* Evaluation of FDG-PET in comparison to conventional imaging modalities (CIM).
*Patients/methods:* In this prospective multicenter study, 20 pt (12f, 8m; mean age: 12.4 y) with a histologically proven, sarcoma (osteosarcoma $n = 5$, Ewing-sarcoma $n = 7$, rhabdomyosarcoma $n = 8$) were examined by FDG-PET for primary staging ($n = 17$) and for detection of recurrence ($n = 3$) resp. The results were compared with CIM (MRI, CT-thorax, bone-scan) within a 10 day interval to the FDG-PET scan.

Histology and clinical/imaging follow-up served as gold standard.
*Results/discussion:* FDG-PET and CIM correctly detected the primary tumor in 17 pts 8/17 pts. had locoregional, distant or mutiple metastases which were correctly indicated by FDG-PET (lung $n=2$, distant $n=4$, multiple $n=5$) 5/8 pts. Concordantly showed metastatic disease in CIM (lung $n=2$, other $n=1$, multiple $n=2$). In contrast to CIM, FDG-PET revealed a more extensive metastatic disease in 7 patients (42%), resulting in upstaging with a consecutive change of therapy. Concerning the detection of local recurrences FDG-PET and CIM were true positive in two, and false negative in 1/3 pts. FDG-PET and CIM are suitable for the staging of primary and recurrent sarcomas, however, due to its apparent higher sensitivity, FDG-PET has a potential impact on further treatment strategy.

### P170

### Retrospective analysis of FDG-PET for staging of malignant childhood sarcomas

T. Voelker[1,2], T. Denecke[2], C. Furth[1,2], D. Misch[1,2], P. Hundsdoerfer[3], R. Felix[2], G. Henze[1], H. Amthauer[2]
*([1]Department for Pediatric Oncology, Clinic of Radiology, [2]Nuclear Medicine and Radiation Oncology, Campus Virchow-Klinikum, Charité – Universitätsmedizin Berlin, Germany, [3]EMBL, European Molecular Biology Laboratory, Heidelberg, Germany)*

*Purpose:* Evaluation of FDG-PET in staging of childhood sarcomas in comparison to conventional imaging modalities (CIM).
*Patients/methods:* 22 pts (12f, 10m; mean age: 11.4 y) with a histologically proven, sarcoma (osteosarcoma $n=12$, Ewing-sarcoma $n=4$, rhabdomyosarcoma $n=6$) were examined by PET for staging ($n=14$) and recurrence detection ($n=8$). The results were compared with CIM (MRI, CT-thorax, bone-scan) within 10 day interval to the PET. Histology and clinical/imaging follow-up served as gold standard.
*Results/discussion:* In 14 pts with sarcomas PET and CIM correctly detected the primary tumor. 4/14 pts showed metastases: Lung $n=1$ (PET neg., CIM pos.), distant $n=3$ (2/3: PET pos., CIM neg. and 1/3: concordantly PET and CIM false pos.)

In 5/8 pts with relapse, restaging by PET and CIM was concordant. In one pt CIM correctly indicated a lung metastasis, undetected by PET. In contrast, PET depicted an additional axillary lymph node manifestation, unnoticed in CIM. In 3/3 pts, a recurrence was correctly excluded by both modalities. PET and CIM are suitable for the staging of sarcomas and the detection of recurrences. However, whereas PET appears to be superior to CIM in the detection of distant metastases, its use for lung metastases is limited.

### P171

### Functional expression of chemokine receptors in childhood osteosarcoma

I. von Luettichau[1,3], M. Notohamiprodjo[1], M. Nathrath[3], M. Kremer, A. Wechselberger[1,3], A. Henger[1], R. Djafarzadeh[1], S. Burdach[3], R. Huss[2], P.J. Nelson[1]
*([1]Medical Policlinic of the Ludwig-Maximilians University, Munich, Germany, [2]Institute of Pathology, Ludwig-Maximilians University, Munich, Germany, [3]Children's Hospital of the Technical University Munich, Germany, Institute of Pathology of the Technical University Munich, Germany)*

Osteosarcoma is the most frequent tumor of the bone in childhood. The major cause of death in osteosarcoma patients is tumour metastasis. Chemokines are small chemotactic proteins initially characterized by their ability to control leukocyte chemotaxis. These proteins have been subsequently shown to regulate proliferation, differentiation, migration and homing of diverse cell types. Chemokines are also thought to be active participants in malignant disease. All chemokines mediate their biological effects through a family of seven-transmembrane G-protein-coupled receptors. The goal of this study was to characterize the functional expression of chemokine receptors on childhood osteosarcoma to identify potential roles for specific chemokines/receptors in the development and progression of tumor metastasis and micrometastasis. We demonstrate functional expression of chemokine receptor CCR1, CCR4, CCR7, CXCR4, CXCR5 and CX3 CR1 on the osteosarcoma cell lines SAOS, HOS and U2-OS using quantitative Taqman analysis, FACS and modified Boyden chamber assays. In parallel, the expression of these receptors was found on 20 pediatric osteosarcoma tumor biopsy specimens. Our data suggest a potential role for chemokines in the biology of childhood osteosarcoma. Insight into mechansisms that underlie metastasis may help facilitate novel therapeutic approaches in future.

### P172

### Identification of specific markers for bone tumor progenitor subtypes

I. von Luettichau[1,3], E. Kremmer[4], J. Myslivietz[4], M. Notohamiprodjo[1], A. Wechselberger[1,3], A. Henger[1], R. Djafarzadeh[1], S. Burdach[3], R. Huss[2], P.J. Nelson[1]
*([1]Medical Policlinic of the Ludwig-Maximilians University, Munich, Germany, [2]Institute of Pathology, Ludwig-Maximilians University, Munich, Germany, [3]Children's Hospital of the Technical University Munich, Germany, [4]Institut of Immunology, GSF, Munich, Germany)*

Osteosarcoma is the most common malignant bone tumor in children and adolescents. Several subtypes of osteosarcoma can be identified based on morphology. Currently we lack a clear understanding of the intrinsic properties that distinguish one subtype from another, and how these differ in their response to similar conditions in vitro and in vivo. Mesenchymal stem cells have a multilineage differentiation potential and are characterized by lack of the hematopoetic markers CD45, CD14, CD11 and CD34, but express CD29, CD73 (SH3), CD105 (SH2), CD166. To date, no individual specific marker adequately identifies these cells. The goal of this study is the development of methods for identifying, isolating and characterizing specific precursor populations at intermediate stages of differentiation of osteocytes, and their relationship to tumor-generating cells. To this end, SV40 large T antigen was used to immortalize human mesenchymal stem cells. These cells were then used as immunogen in rat to generate a panel of monoclonal antibodies directed against surface proteins of the progenitor cells. These antibodies are largely negative for peripheral blood cells, but positive for primary human mesenchymal stem cells. They show a dramatic variability in positivity on the osteosarcoma cell lines U2-OS, HOS,

SAOS, MG63 and CRL and some have been adapted for use in immunohistochemistry.

### P173

### Pediatric Soft Tissue Sarcomas (STS) in Iran

P. Vossough[1], M. Alebouyeh[2], Kh. Arjmandi[3], M. Faranoush[4], Sh. Ansari[3], Gh. Bahoush[3], E. Shahgholi[5], A.A. Hedayatiasl[6]
*([1]Department of Pediatric Hematology and Oncology, Iran University of Medical sciences, Aliasghar Children's Hospital, Iran, [2]Department of Pediatric Hematology and Oncology, Shahid Beheshti University of Medical Sciences, Shohada Hospital, Iran, [3]Department of Pediatric Hematology and Oncology, Iran University of Medical Sciences, Aliasghar Children's Hospital, Iran, [4]Department of Pediatric Hematology and Oncology, Semnan University of Medical Sciences, Amir Al Momenin Children's Hospital, Iran, [5]Department of Pediatric Hematology and Oncology, Tehran University of Medical Sciences, Bahrami Children's Hospital, Iran)*

*Background:* STSs account for 7% of all childhood tumors. Our survey was an epidemiologic study on soft tissue sarcoma in a single institution.
*Methods and materials:* In a cross sectional analytic study, the patients were evaluated for age, sex, histologic type of cancer, stage, primary site of tumor, chemotherapy protocol and outcome.
*Results:* STSs were the fifth most frequent malignancies amongst patients of this center (253 cases). The rate was 4.7% and about 85% of them were rhabdomyosarcoma. Others types were malignant fibrous histiocytoma 3.9%, angiosarcoma 3.13%, fibro sarcoma 2.34% and undifferentiated sarcoma 1.56%. M/F ratio were 1.37. The average age of children was 5.3 ± 0.6 yrs old. About 60.5% of patients were younger than six years. Most patients were in stage I–II (81%). Embryonal subtype was 75%. Primary site of involvement were 57.98% in head and neck, 6.72% in extremity and 35.29% in trunk. The overall 5 years survival rate was 72%. Relapse occurred in 25% of patients. Chemotherapy protocol included VCR/ACT/CPA/ADR in 32.85%, VCR/ACT 35.42%, VCR/ACT/CPA in 31.73% and IF/VCR.
*Conclusion:* The prognosis of STS varies greatly depending on the age, primary site, tumor size, histologic grade and extent of disease at diagnosis. The most patients in stage I–II had good prognosis.

### P174

### L1 cell adhesion molecule (CD171) expression in gastrointestinal stromal tumors (GIST)

J.T. Kaifi[1], K.A. Gawad[1], A. Strelow[1], E.F. Yekebas[1], R. Wachowiak[1], W. Schaefer[2], M. Schachner-Camartin[3], J.R. Izbicki[1]
*([1]Klinik fuer Allgemein-, Viszeral- und Thoraxchirurgie, [2]Institut fuer Pathologie, [3]Zentrum fuer Molekulare Neurobiologie, Universitaetsklinikum Hamburg-Eppendorf, Martinistrasse 52, 20246 Hamburg, Germany)*

*Background:* After surgical resection the outcome of gastrointestinal stromal tumors (GIST) is hardly predictable. Cell adhesion molecules have an essential function in tumor progression and recently this was demonstrated for L1 cell adhesion molecule (L1CAM; CD171). The aim of our study was to determine expression of L1CAM in GIST and correlate expression with development of metastases.
*Methods:* We retrospectively analyzed L1CAM expression in 55 surgically resected GIST (primary tumors or metastases) by immunohistochemistry on paraffin sections. Staining was performed by peroxidase technique with monoclonal antibody UJ127.11 against L1CAM. Expression results were compared with development of metastases in these patients.
*Results:* L1CAM was detected in 36 (65.5%) of 55 GIST. 23 (41.8%) of 55 patients suffering from GIST developed metastases and among these 13 were positive for L1CAM. $\chi^2$ test did not show a significant association between L1CAM expression and metastasizing GIST ($p = 0.264$ by Fisher's exact test).
*Conclusion:* Our data suggest that L1CAM is expressed in a high number of GIST and could potentially serve as a marker for this tumor. L1CAM expression did not correlate with development of metastases in GIST patients and is consequently not a predictor for metastasizing GIST.

### P175

### Chemosensibilization in patients with unresectable soft tissue and osteogenic sarcomas by focused microwaves achieving intratumoral tissue temperatures of 42 to 44°C

R. Wessalowski[1], D.T. Schneider[1], O. Mils[1], M. Cohnen[2], H. Pape[3], M. Paulussen[4], R. Ladenstein[5], E. Kosicielniak[6], T. Klingebiel[7], J. Engert[8], D. Harms[9], U. Göbel[1]
*([1]Clinic of Pediatric Oncology, Hematology and Immunology, [2]Institute of Diagnostic Radiology, [3]Department of Radiation Oncology, Heinrich Heine University Dusseldorf, [4]Clinic of Pediatric Hematology and Oncology, Westfälische Wilhelms University, Münster, Germany, [5]St. Anna Children's Hospital, Vienna, Austria, [6]Olgahospital, Stuttgart, [7]Children's University Hospital, Frankfurt, [8]Department of Pediatric Surgery, Ruhr-University Bochum, [9]Institute of Paidopathology, University of Kiel, Germany)*

*Background:* Multidisciplinary risk-adapted regimens have achieved remarkable cure rates in sarcoma patients. However, a number of patients still fail therapy due to insufficient local tumor control. We and others have previously shown that the application of heat at temperatures of 40–44°C increases the cytotoxicities of anticancer drugs including N-lost derivatives, cytotoxic antibiotics and platinum analoga. Therefore, we investigated whether thermochemotherapy (TCH) can be utilized to facilitate surgical resection in otherwise nonresectable sarcomas and whether this combination improve local tumor control.
*Patients/methods:* This study enrolled 25 patients (female = 9, male = 16, age 1–59 years), 5 osteogenic, 20 soft tissue sarcomas. Indication: loco-regional relapse ($n = 9$), unresectable tumor ($n = 16$). Site: pelvis (17), abdomen (1), head/neck (2), extremities (4), spine (1).

Hyper-PEI-protocol: 4 × 1800–2000 mg ifosfamide/qm, 4 × 100 mg etoposide/qm, 2 × 40 mg cisplatin/qm plus 2 × hyperthermia (42–44°C, 1 h) or RHT-95-protocol: 1 × 50 mg adriamycin/qm, 4 × 1500 mg ifosfamide/qm, 2 × 100 mg etoposide/qm plus 2 × hyperthermia (42–44°C, 1 h).
*Results:* 141 TCH-courses (279 heat sessions) were applied, 6 patients achieved CR, 12 PR. TCH was followed by surgical resection in 15/25 and/or radiotherapy in 6/25 patients. Outcome: 13NED, 2AWD, 10DOD.
*Conclusion:* TCH shows substantial therapeutic efficacy and facilitates complete tumor resection in 11/15 operated patients. Multimodal treatment including TCH leads to sustained remission in about 50 per cent of patients.

### P176

**The value of positron emission tomography scanning with fluorine-18 deoxyglucose in the detection of local and metastatic disease in childhood soft tissue and bone sarcomas and evaluation of response to chemotherapy**

M. Weyl Ben Arush[1], R. Bar Shalom[2], S. Postovsky[1], Z. Keidar[2], A. Ben Barak[1], R. Elhasid[1], I. Zeidman[1], M. Haimi[1], I. Meller[3], Y. Kollender[3], O. Israel[2]
*([1]Pediatric Hematology Oncology Department, Rambam Medical Center, Haifa, Israel, [2]Nuclear Medicine Department, Rambam Medical Center, Haifa, Isreal, [3]Oncology Orthopedic Unit, Sorasky Medical Center, Tel Aviv, Israel)*

*Aim:* To assess the diagnostic value of positron emission tomography with fluorine-18-deoxyglucose (FDG-PET/CT) in the detection of local, recurrent and metastatic sarcomas (S) in children and evaluation of response to standard chemotherapy.
*Patients/methods:* 26 children, 14 boys, 12 girls, median age 12.7 years. FDG-PET scans were compared to surgical pathology or clinical follow up (FU) for a median of 6 months (5 m–48 m). 8 pts performed FDG-PET at diagnosis, restaging, and detection of recurrence, 11 pts at diagnosis and restaging, 15 pts at restaging and FU. Eleven pts were evaluated for response to chemotherapy.
*Results:* For detection of local disease, FDG-PET had a sensitivity of 0.92, specificity – 0.94 and accuracy – 0.93, detection of non pulmonary metastases: sensitivity – 100, specificity – 0.50, accuracy – 0.70, detection of lung metastases: sensitivity – 0.66, specificity – 0.91, accuracy – 0.86.There were 5 false negative, 5 false positive. FDG uptake values decreased significantly for 6 pts with favorable histological response to chemotherapy.
*Conclusions:* FDG-PET is a sensitive test to detect local disease and non pulmonary distant recurrences of sarcomas. In the future, FDG-PETscanning in childhood sarcomas has to be directed to the clinical implication for detection and treatment evaluation of childhood sarcomas.

### P177

**Differences in survival between study groups detected in an international randomised study: Analysis of Eicess 92**

J. Whelan[1], A. McTiernan[1], C. Weston[2], S. Ahrens[3], R. Grimer[4], I. Lewis[5], C. Douglas[2], A. Cassoni[1], D. Spooner[6], M. Paulussen[3], H. Jurgens[3], A. Craft[7]
*([1]The Meyerstein Institute of Oncology, UCL Hospitals, London, UK, [2]UKCCSG Data Centre, University of Leicester, UK, [3]Department of Pediatric Hematology and Oncology, University Children's Hospital, Muenster, Germany, [4]The Royal Orthopaedic Hospital, Birmingham, UK, [5]Regional Paediatric Oncology Unit, St. James University Hospital, Leeds, UK, [6]Queen Elizabeth II Hospital, Birmingham, UK, [7]Inst. of Child Health, The Royal Victoria Infirmary, Newcastle upon Tyne, UK)*

*Background:* EICESS 92 was a randomised study involving 647 patients with Ewing tumours conducted by two study groups, UKCCSG and GPOH, between 1992–8. Survival at 5 years for all patients was 62.6% (95% CI 58.8–66.5); for GPOH patients 66.5% (95% CI 62–71.1) and for UK patients, 54.3% (95% CI 47.3–61.4, $p = 0.009$).
*Methods:* A detailed analysis of trial data to find cause for survival difference.
*Results:* This could not be accounted for by differences in patient baseline characteristics or differences in delivery of chemotherapy. Differences were evident between the frequencies of local treatment modalities. Surgery or radiotherapy alone was more often used in UK patients. Pre-operative radiotherapy was used often in the GPOH. Local recurrence with or without metastatic recurrence was commoner in the UK (21.4% vs. 7.1%). Survival differed according to local treatment received (surgery alone best, radiotherapy alone worst, $p = 0.0001$). Analyses of subgroups failed to identify a clear causative association between local treatment modality and survival differences between the 2 groups.
*Conclusion:* Differences in outcome can occur between study groups within the same trial. Different approaches to local treatment of Ewing's tumours led to higher local recurrence rates in UK patients and may have been associated with inferior survival.

### P178

**Fibrodysplasia ossificans progressiva in children**

A. Szymborska, T. Klepacka, M. Szeliga, W. Woźniak
*(The Clinic of the Oncological Surgery in Children and Adolescents, Institute of Mother and Child, Warsaw, Poland)*

Fibrodysplasia ossificans progressiva (FOP) is rare genetic disease, heredited by autosomal dominant way.

Characterized by heterotopic forming bone tissue in subcutaneous and muscular tissue. In majority cases it begins in the first decade of life. Growing pain symptoms with oedema of soft tissue and limited joint mobility leads to disability.

Localisation most often in skeletal muscles, muscles of extremities and small muscles of fingers. In diagnosis fundamental role plays the presence of abnormalities of finger bones as halux, short halux, microdactylia and different bone deformations. Histopathological features often suggest osteosarcoma, scleroderm, fibrosarcoma, osteodystrofia Albrighta, neurofibromatosis. Important role in diagnosis seems to have BMP-4.

There are no elaborated standards for clinical treatment in FOP. Also there is no effective method of treatment FOP.

Gen therapy with BMP-4 raises the hope for effective FOP management.

### P179

**Bone tumors in pelvis in children: Limb salvage operations**

W. Woźniak, M. Rychłowska-Pruszyńska, M. Kuczabski, T. Izbicki, T. Walenta, A. Szafrański, K. Bilska, J. Kijowski

In the period of 1985–2003 more than 400 patients with primary bone malignant tumors (osteosarcoma, Ewing sarcoma, chondrosarcoma) were treated in the Institute of Mother and Child in Warsaw. In all of them contemporary methods of complex treatment were applied, including chemotherapy, surgery and/or radiotherapy. Localization of primary neoplasm's focus in the pelvis is less common and the radical surgery is problematic in children.

We present a group of 19 patients in whom following operations were preformed: 2 "classical" hemipelvectomies—excision of pelvis bone with exarticulation of lower extremity; 17 internal hemipelvectomies—excision of pelvis bone sparing the lower extremity; 3 patients, type I—hip bone ala excision above the hip articulation; 2 patient, type II A—excision of the hip bone ala with the hip articulation; 3 patients, type II B—ischiadic and pubic bone excision with the hip articulation; 4 patients, type II C—excision of the hip bone ala with the hip articulation, public and ischiadic bones; 2 patients, type I/III; 3 patients, type III—pubic and ischiadic bones excision under the hip articulation.

Internal hemipelvectomies have been made mainly in last four years. In reconstruction was use bone grafts metal and endoprothesies plates. Ten pas are alive with the follow-up of 20–60 months. The patients have been rehabilitating the salved limb.
*Conclusions:* 1. The internal hemipelvectomy is a less mutilating operational treatment, performed within the complex therapy of malignant bone tumors in children and youth. 2. The internal hemipelvectomy allows the limb salvage and efficacious patient's walk. 3. The internal hemipelvectomy requires a very well prepared operation team, good technical background allowing reconstruction of the resected bones rehabilitation in the postoperational time.

**P180**

**Chemotherapy in osteosarcoma: Results of treatment in own material by the Polish Pediatric Solid Tumor Group**
W. Woźniak, A. Chybicka, J. Bogusławska–Jaworska, J. Bohosiewicz, J. Kowalczyk, P. Kołecki, M. Korzon, M. Liebhart, M. Rybak, M. Wysocki, W. Gołębiowski, B. Kazanowska, J. Kijowski, A. Szafrański, M. Rychłowska, T. Izbicki, M. Kuczabski, J. Węcławek-Tompol, K. Kątski, W. Madziara, M.S. Popadiuk, M. S. Boruczkowski, M. Leda
*(Polish Pediatric Centers for Osteosarcoma Treatment, The Clinic of the Oncological Surgery in Children and Adolescents, Institute of Mother and Child, Warsaw, Poland)*

*Objectives:* In the analysis two chemotherapy regimes: EORTC and the adopted French HD-MTX protocol (HD-MTX + DOXO/IFO +), which are using in the treatment of osteosarcoma patients by Polish Pediatric Solid Tumor Group, were presented.
*Methods:* According to results of treatment in osteosarcoma patients, which were treated by these two chemotherapy regimens, the primary analysis was made, to compare in the standardized way the efficacy of two osteosarcoma chemotherapy regimens.
*Results:* In the period 1998–2003, 111 patients with osteosarcoma were treated in 6 Polish Pediatric Oncological Centers. 56 patients were treated by EORTC protocol, 24 by the adopted French HD-MTX protocol (HD-MTX + DOXO/IFO + ETO) and 15 patients by the SFOP/different protocols. After neoadjuvant chemotherapy the surgical treatment was performed. Limb salvage surgery was performed in 41 from 56 patients which were treated by EORTC protocol, in 20 from 24 treated by SFOP protocol.

52 patients are alive from the group treated by EORTC protocol. In the group which were treated by SFOP protocol, 28 alive, in the group treated by SFOP/different protocol, 11 alive from 15 patients, with mean follow-up 60 months.

**P181**

**Clear cell sarcoma of soft tissues: An unexpected diagnosis**
C.S. Wynne[1], E.D. Gent[2], I.E. Moore[3], J. Sastry[1]
*([1]Department of Paediatric Oncology, Southampton General Hospital, Southampton, UK, [2]Department of Orthopaedic Surgery, Southampton General Hospital, Southampton, UK, [3]Department of Histopathology, Southampton General Hospital, Southampton, UK)*

Clear cell sarcoma is a rare soft tissue neoplasm whose clinical behaviour and outcome has not been previously characterized in children. We report a case of clear cell sarcoma of soft tissues, affecting the great toe.
*Patients/methods:* A four-year-old boy presented with pain in a swelling overlying the terminal interphalangeal joint of the great toe. This lesion had been present at birth and had grown slowly, with no bleeding or colour change. Examination showed a discoloured firm swelling measuring $1 \times 1.5$ centimetre, over the proximal part of the hallux. No other abnormality was detected.
*Results:* An ultrasound scan was suggestive of a well-encapsulated haemangioma. After an initial observation period of six months, a decision to excise the lesion was taken in view of pain and difficulty wearing shoe, even though size did not change significantly. Microscopically, it was multinodular, intersecting and infiltrating tendinous tissue, extending to the excision margins. Spindled cells and multinucleated giant cells were present with melanin deposition, mild pleomorphism and mitoses. On staining, the cells were positive for vimentin, HMB45, NSE, S100 and negative for MNF 116, CD68, CD1a, ASMA and desmin. CD34 highlighted vasculature and giant cells were negative with Factor 13a. Ki67 showed mild proliferation. The diagnosis was Clear Cell Sarcoma of soft tissues with residual microscopic disease, categorised as stage 2 (IRS grouping). There was no evidence of metastatic spread on bone scan and CT of chest and abdomen. There was no evidence of loco-regional spread on MRI scan of the lower limb and inguinal region. In view of the residual microscopic disease, amputation of the great toe was undertaken to obtain clear margins. The functional outcome was satisfactory.
*Discussion:* Clear Cell Sarcoma of soft tissues is a rare tumour in children. There are only few reports among children in literature.[1,2] UKCCSG is developing a protocol for the non Rhabdomyosarcoma Soft Tissue Tumours (ESSG-NRSTS). Complete surgical resection with negative margins is the most effective treatment for this disease. Patients with metastatic disease are candidates for multi-institutional chemotherapy trials.

**P182**

**Identification of regulatory proteins critical for T-cell activation in blood of sarcoma patients**
E.M. Zadorin, K.V. Shevchenko
*(Central Research Laboratory Centre, Kiev, Ukraine)*

The activation of T cells, mediated by the T-cell receptor (TCR), activates a battery of specific membrane-associated, cytosolic and nuclear proteins. Identifying the signalling proteins downstream of TCR activation will help us to understand the regulation of immune responses and will contribute to developing therapeutic agents that target immune regulation. In an effort to identify novel signalling molecules specific for T-cell activation we undertook a large-scale dominant effector genetic screen using retroviral technology. We discovered molecules not previously known to have functions in this pathway, including a novel protein with a RING domain (found in a class of ubiquitin ligases; we call this protein TRAC-1), transmembrane molecules (EDG1, IL-10R$\alpha$ and integrin $\alpha$2), cytoplasmic enzymes and adaptors (PAK2, A-Raf-1, TCPTP, Grb7, SH2-B and GG2-1), and cytoskeletal molecules (moesin and vimentin). Furthermore, using truncated Lck, PLC$\gamma$1, EDG1 and PAK2 mutants as examples, we showed that these dominant immune-regulatory molecules interfere with IL-2 production in oncology patients primary lymphocytes. This study identified important signal regulators in T-cell activation. It also demonstrated a highly efficient strategy for discovering many components of signal transduction pathways and validating them in treatment of oncology patients.

**P183**

**Oxidative stress regulates the expression and activity of transcription factor activator protein-1 in rat sarcoma models**

K.V. Shevchenko, E.M. Zadorin
*(NBS, Kiev, Ukraine)*

The transcription factor activator protein-1 (AP-1), composed of the Fos and Jun families of proto-oncogenes, is induced in response to extracellular signals as part of an immediate-early gene response. We hypothesize that teratogens such as oxidative stress induce AP-1 activity in the rat conceptus and that this AP-1 response may either trigger abnormal development or protect the embryo against insult. To test this hypothesis, the AP-1 response was assessed in whole embryos in culture. There was a significant elevation in the oxidized to reduced glutathione ratio in the embryo and yolk sac within 0.25 hr of the initiation of culture, peaking at 0.5 hr; this is indicative of heightened oxidative stress. Thus, there are tissue-specific differences in the duration of the AP-1 response in the conceptus. Addition of the antioxidants catalase and superoxide dismutase, but not vitamin E, prevented the rise in the oxidized to reduced glutathione ratio and also inhibited the induction of AP-1 mRNAs and DNA-binding activity. The AP-1 response to oxidative stress may determine how the conceptus responds to sarcoma. These results can be used in medicine.

**P184**

**Reasons for medical facility visits for teenage & younger European children with sarcomas attending Barretstown Therapeutic Camp: A four year comparative study for quality of life**

E. Kinsella[1], T. Dignan[1], F. Breatnach[1], P. Zeltzer[2]
*([1]Barretstown Gang Camp, Dublin, Barretstown, Ballymore, Ireland, [2]Shilysca Inc. Los Angeles, USA)*

*Purpose:* Assess medical facility visits (MFV) for children with sarcomas (S)
*Methods:* Prospective collection of needs/events over 28, 10-day camps (1998–2001). Campers: (a) 7–13 yrs, (b) >14–17 yrs. Teen Camp. Staff = 2 volunteer physicians, 3–4 nurses/75 campers. Collected data were demographics, reason for visit (disease-related?).
*Results:* 2246 children ages 7–18, 26 countries, were supported by 836 staff. Median age = 12; M/F = 1.3/1. Most common diagnoses: Leukemia = 30%, lymphoma = 15%, sarcoma = 13% (283); other = 42%. Subgroups: Ewing-S = 100; osteo-S = 94; rhabdomyo-S = 48; S = 13, soft tissue-S = 12; synovial-S = 4; other = 12. Age = 13 + 3. Countries of origin had different distributions than other cancers: Spain, Hungary = highest. Age distributions differed. Total of 3378 MFV for campers and 1785 for staff. Of 283 S pts, 119 were available to assess visit frequency (yrs 2000–2001); S children made 227 visits (range 0–10) x = 1.9. M/F visit freq = 1.0; other diseases = 1.5. Most frequent MFV were misc. complaints (50%), pain, headache, cuts/abdominal pain. 31% MFV were disease/treatment related vs. 10% for other cancers.
*Conclusions:* (1) With appropriate medical and staff support, Barretstown demonstrated a high safety profile for S pts. (2) Most medical interventions were related to normal camp issues: bee sting, activity-related injury, etc; 69% MFV were not disease-related. (3) Staff account for 35% total visits.

**P185**

**The EpSSG RMS2005: A randomized trial for localised rhabdomyosarcoma**

G. Bisogno, C. Bergeron, M. Jenney, E. Koscielniak, S. Gallego, C. Rechnitzer, B. Kazanowska, A. Schuck, A. Kelsey, V. Ninfo, G.L. De Salvo, A. Ferrari, J. Treuner, M. Carli, O. Oberlin, M. Stevens
*(EpSSG)*

*Introduction:* RMS2005 is the first protocol launched by the European paediatric Soft tissue sarcoma Study Group (EpSSG). It addresses the treatment of young people (<21 years) with non-metastatic rhabdomyosarcoma.

The protocol comprises a randomised trial for "high risk patients" and observational studies for patients categorized in other risk groups.
*Methods:* The analyses of previous European studies has identified a High Risk Group: patients in IRS group II or III, with favourable pathology but unfavourable site and tumour size or age; patients in IRS Group I, II or III with favourable pathology, site, size and age but with nodal involvement; all patients with unfavourable histology except alveolar N1.

One aim of the study is to improve the outcome of these patients by implementing two novel strategies: (1) the intensification of initial chemotherapy adding anthracyclines to the standard IVA regimen, (2) the adoption of a low dose maintenance treatment after 1st line chemotherapy.
*Treatment:* Patients will be randomized on day 0 to receive IVA (ifosfamide, vincristine, actinomycin D) vs. IVADo (IVA + Doxorubicin) over the initial 4 cycles. This is followed by 5 IVA cycles for all patients. This combination has been tested in a pilot study and judged feasible. After the standard 9 cycles of chemotherapy patients in complete remission will be randomised to stop treatment or receive maintenance chemotherapy comprising low dose cyclophosphamide and vinorelbine for 6 months.

Surgery will depend on initial IRS group staging. All patients will receive radiotherapy with doses ranging between 36 Gy and 50.4 Gy depending on histology, resection margins and tumour response.
*Conclusions:* Increasing European collaboration should allow the exploration of better treatment strategies for children with RMS through randomised studies.

**P186**

**Skip metastases in osteosarcoma. Experience of the cooperative osteosarcoma study group**

L. Kager[1], A. Zoubek[1], S. Lang[2], G.U. Exner[3], R. Maas[4], C. Franzius[5], B. Kempf-Bielack[6], H. Jürgens[6], H. Gadner[1], S. Bielack[6]
*([1]COSS Study Group, St. Anna Children's Hospital, Vienna, [2]Department of Pathology, University Hospital Vienna, Austria, [3]Department of Orthopedics, University Hospital Zürich, Switzerland, [4]Department of Radiology, Hamburg, [5]Department of Nuclear Medicine, University Hospital Muenster, [6]Department of Pediatric Hematology and Oncology, University Children's Hospital Muenster, Muenster, Germany)*

*Purpose:* Detection of "Skip" metastases (SM) herein defined as second, smaller foci of OS occurring in the same bone or as second lesion(s) of OS on the opposing side of a joint is rare in patients with high grade osteosarcoma (OS). The prognosis for OS patients presenting with SM is reported to be catastrophic.
*Patients and methods:* The authors surveyed the medical records of 1765 consecutive collected patients with newly diagnosed high-grade OS of bone registered in the neo-adjuvant COSS studies and identified 24 patients (1.4%) with unequivocally proven SM (radiographically by progression in 1 and histologically in 23). All 24 patients were treated by an aggressive surgical approach coupled with polychemotherapy. Demographic, diagnostic, tumor, and treatment related variables; response and survival data were analyzed.
*Results:* SM were identified preoperatively in 11/24 (46%) patients by bone scan, 8/22 (36%) by plain X-ray, 5/11 (45%) by CT, and 15/19 (79%) by MRI. A complete surgical resection of all clinically detectable tumor sites had been achieved in 22/24 patients (92%) during front line therapy. With a median follow-up of 4.4 years (7 years for survivors), 12 patients (50%) were alive, all in continuous complete surgical remission. Prognosis was significantly better in the 16 patients with SM occurring within the same bone of the primary, when compared to the 8 patients with transarticular SM (5-year survival 62%, SE = 12% vs. 25%, SE = 15%).
*Conclusion:* SMs are rare in osteosarcoma and preoperative detection relies on appropriate diagnostic imaging. Aggressive multimodal therapy holds the promise to achieve cure, especially in those patients in whom SM occur within the same bone of the primary lesion.

### P187

**The role of liposomal anthracyclines added to ifosfamide in the treatment of advanced soft tissue sarcomas**

J.M. Siehl, U. Keilholz, A. Schmittel, G. Hütter, H. Szelenyi, E. Thiel
*(Medizinische Klinik III, Charité Universitätsmedizin Berlin, Campus Benjamin Franklin, 12200 Berlin, Germany)*

Actually the standard chemotherapy for advanced soft-tissue sarcomas is a combination of doxorubicin and ifosfamide. For both drugs there is a dose-response relationship. The main problem, however, is a high rate of toxicities.

Liposomal encapsulation of anti-cancer drugs is a strategy pursued to reduce toxicity and improve tumour uptake. Liposomal anthracyclines are far less cardiotoxic as the conventional formulations and they might accumulate at the site of the tumour. So far there are little data on the efficacy of liposomal anthracyclines in advanced soft-tissue sarcomas when used as single agents. The role of liposomal anthracyclines in combination with ifosfamide is yet to be determined.

We combined in a phase II study liposomal daunorubicine with ifosfamide in the treatment of advanced soft tissue sarcoma. 40 patients were enrolled, 35 of them were treated first line. In another 4 patients we combined liposomal doxorubicine with ifosfamide.

The toxicity of these combinations was tolerable. The response rate was 31% with a median overall survival of 14 months.

Therefore the combination of liposomal anthracyclines with ifosfamide turned out to be a safe and effective regimen in the treatment of advanced soft tissue sarcoma. Further evaluation should be performed.

### P188

**Osteosarcoma treatment in children**

V. Kobys, G. Klymnyuk, T. Tarasova, O. Balytskaya, E. Shayda, N. Repina, A. Dedkov
*(Institute of Oncology of the Academy of Medical Sciences of Ukraine, (Kiev, Ukraine))*

*Introduction:* Improvement osteosarcoma treatment results in children still remains an actual problem despite achieved survival rate within 70–80%. The application of preoperative chemotherapy correction we consider to be promising for the improvement of chemotherapy efficiency.
*Material and methods:* The chemotherapy scheme with individual matching of preoperative therapy for osteosarcoma local forms was developed and approved at the Institute of Oncology from 1999. 28 patients were treated with this scheme. The purpose of this work is to analyze and summarize treatment results of this patients' group. The middle age was 12.5 years. The following drugs were used: Methotrexate 12 g/m$^2$, cysplatin 40 mg/m$^2$ 1–3 day, doxorubicin 25 mg/m$^2$ 1–3 day. Holoxane was used when inefficiency or low efficiency of previous drug combinations was fixed by complex examinations: Extremity X-ray, CT, Doppler US.
*Results:* According to pathohistology of the operating material pathomorphism of III–IV stage (after Havos) was in 17 patients (60%). 22 patients (79%) are alive from 16 months to 5 years. Survival cumulative index during the period of 24 months is 79.2% ($p < 0.05$). Thus, the developed treatment protocol of osteosarcoma in children using individual matching of preoperative therapy demonstrated high efficiency, but small quantity of patients and limited terms of observation require further investigations.

